# *N-*Aminopyridinium reagents as traceless activating groups in the synthesis of *N*-Aryl aziridines

**DOI:** 10.1038/s41467-022-31032-w

**Published:** 2022-06-10

**Authors:** Hao Tan, Samya Samanta, Asim Maity, Pritam Roychowdhury, David C. Powers

**Affiliations:** Department of Chemistry, Texas A&M University, College Station, TX 77843 USA

**Keywords:** Synthetic chemistry methodology, Homogeneous catalysis

## Abstract

*N-*functionalized aziridines, which are both useful intermediates and important synthetic targets, can be envisioned as arising from the addition of nitrenes (*i.e*., NR fragments) to olefinic substrates. The exceptional reactivity of most nitrenes, in particular with respect to unimolecular decomposition, prevents general application of nitrene-transfer to the synthesis of *N*-functionalized aziridines. Here we demonstrate *N*-aryl aziridine synthesis via 1) olefin aziridination with *N*-aminopyridinium reagents to afford *N*-pyridinium aziridines followed by 2) Ni-catalyzed C–N cross-coupling of the *N*-pyridinium aziridines with aryl boronic acids. The *N*-pyridinium aziridine intermediates also participate in ring-opening chemistry with a variety of nucleophiles to afford 1,2-aminofunctionalization products. Mechanistic investigations indicate aziridine cross-coupling proceeds via a noncanonical mechanism involving initial aziridine opening promoted by the bromide counterion of the Ni catalyst, C–N cross-coupling, and finally aziridine reclosure. Together, these results provide new opportunities to achieve selective incorporation of generic aryl nitrene equivalents in organic molecules.

## Abstract (web summary)

Aziridines are useful intermediates, present in important synthetic targets. Here, the authors show a strategy for the synthesis of N-aryl aziridines based on N-aminopyridinium reagents followed by Ni-catalyzed C–N cross-coupling of N-pyridinium aziridines with aryl boronic acids.

## Introduction

**Fig. 1:**
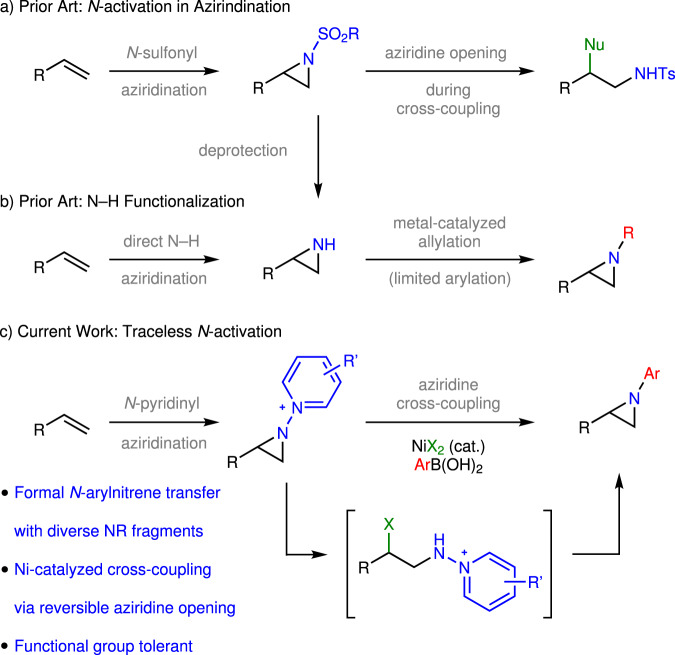
N-Activation strategies for aziridination. **a** Nitrene transfer to olefins provides access to aziridines but often requires the utilization of sulfonyl groups to activate the nitrogen. **b** N–H aziridines can be accessed directly from olefins and metal-catalyzed allylation methods enable functionalization of the N–H valence. **c** Here we advance *N*-pyridinium aziridines as platforms for C–N cross coupling to provide access to *N*-substituted aziridines.

Aziridines, which are three-membered nitrogen-containing heterocycles, are attractive synthetic intermediates en route to 1,2-aminofunctionalization products and are present in various naturally occurring alkaloids and pharmacologically active compounds^1–4^. Retrosynthetically, aziridines can be envisioned as arising from the combination of a nitrene equivalent with an olefinic substrate. In practice, aziridination via nitrene transfer is severely limited by the promiscuous reactivity of unstabilized nitrenes^5^: For example, attempts to access *N*-phenylaziridines from phenylnitrene (generated by thermolysis or photolysis of phenyl azide) result in polymeric tars instead of the desired aziridine^6^. Since Evans’s report of Cu-catalyzed olefin aziridination^7^, myriad transition metal-catalyzed methods have been developed for nitrene transfer to olefins (Fig. 1a)^8–10^. Metal-catalyzed nitrene transfer catalysis typically requires electron-withdrawing groups, such as *N*-sulfonyl substituents, to activate the nitrogen equivalent for transfer^11–13^; there are limited reports of metal-catalyzed nitrene transfer from aryl azide precursors^14, 15^. The resulting *N*-protected aziridines can be challenging to utilize in downstream *N*-functionalization chemistry. For example, exposure of *N*-sulfonyl aziridines to metal-catalyzed cross-coupling conditions typically results in aziridine opening, not *N*-functionalization^16–24^. N–H aziridines can be accessed by either deprotection of *N*-protected aziridines^25–27^ or by direct synthesis of olefinic precursors (Fig. 1b)^28–32^. While derivatization of the N–H valence can provide access to some *N-*functionalization products, arylation of these compounds is not broadly developed^33–37^.

*N-*aminopyridinium reagents represent a burgeoning class of bifunctional reagents^38^ which combine a nucleophilic *N*-amino group with a low-lying pyridinium-centered LUMO that enables access to N-centered radicals via reductive N–N cleavage (LUMO = lowest unoccupied molecular orbital)^39^. In the context of amination chemistry, *N*-sulfonylaminopyridiniums have been utilized in photoredox-promoted olefin difunctionalization^40–47^ and aromatic C–H amination reactions^48–54^. These reactions also rely of the presence of the electron withdrawing *N-*sulfonyl substituents to stabilize incipient *N*-centered radicals. Broad application of *N*-aminopyridiniums as bifunctional reagents in amination chemistry is stymied by the limited methods currently available to prepare *N*-functionalized aminopyridiniums, which are accessed by either addition of hydrazines to pyrylium salts or by sulfonylation of *N*-aminopyridiniums^55, 56^.

In this work, we describe the first example of olefin aziridination with *N*-aminopyridinium reagents (Fig. 1c). Inspired by the Ni-catalyzed C–C coupling chemistry of *N*-alkylpyridinium electrophiles pioneered by Watson^57–60^ and others^61–66^, we demonstrate that the resulting *N*-pyridinium aziridines are competent electrophiles for C–N bond-forming cross-coupling with aryl boronic acids to afford *N*-aryl aziridines. Analogous chemistry is unknown for *N*-tosyl or *N*-phthalyl aziridines. Moreover, in contrast to classical methods for functionalization of N–H aziridines based on *N-*centered nucleophilicity, the described protocol leverages *N-*centered electrophilicity to provide access to the products of formal aryl nitrene transfer to olefinic substrates. Initial mechanistic experiments suggest that the cross-coupling proceeds via a non-canonical mechanism involving halide-promoted ring opening, C–N bond-forming cross coupling, and aziridine reclosure.

## Results

**Fig. 2:**
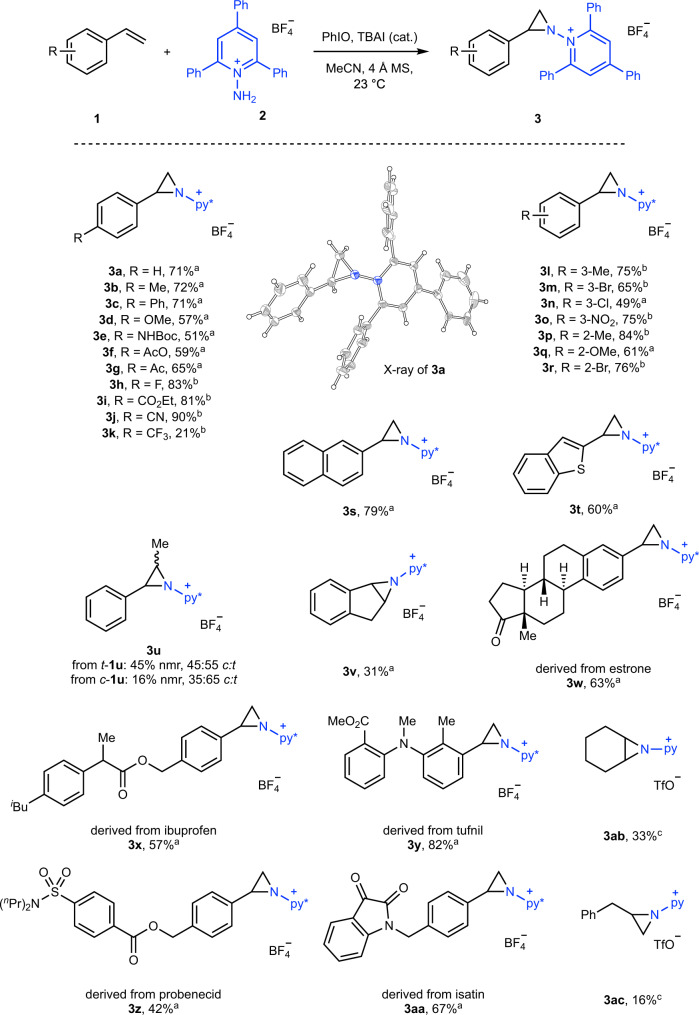
Iodide-catalyzed olefin aziridination. Condition **a** **1** (1.0 equiv), **2** (1.0 equiv), PhIO (1.0 equiv), TBAI (5 mol%); condition **b** **1** (1.0 equiv), **2** (1.6 equiv), PhIO (1.6 equiv), TBAI (20 mol%); condition **c** **1** (1.0 equiv), *N*-aminopyridinium triflate (1.0 equiv), PhIO (1.0 equiv), TBAI (5 mol%). X-ray structure of **3a** presented as displacement ellipsoid plot (50% probability) with BF_4_^–^ counterion removed for clarity. py* = *N*-2,4,6-triphenylpyridinium, py = *N*-pyridinium.

We began the development of a formal nitrene transfer sequence by developing robust conditions for olefin aziridination with *N*-aminopyridinium reagents as nitrogen sources. Combination of styrene and *N*-aminopyridinium triflate in the presence of iodobenzene diacetate (PhI(OAc)_2_) and MgO resulted in 1-(2-phenylaziridin-1-yl)pyridin-1-ium in 64% yield. Aziridination could also be accomplished using *N*-amino-2,4,6-triphenylpyridinium tetrafluoroborate (**2**) as the nitrogen source under these conditions (42% yield of the corresponding pyridinium aziridine (**3a**)). During subsequent studies of C–N cross coupling (vide infra), the triphenyl derivative was found to provide superior results and thus we optimized the aziridination reaction with compound **2** as the pyridinium source. Examination of the impact of various catalysts, solvents, reaction temperatures, and additives (see Supplementary Information Section C.1 for details) identified optimized aziridination conditions based on iodide catalysis in the presence of 4 Å molecular sieves, which affords aziridine **3a** in 71% yield (Fig. 2).

An array of 4-substituted vinyl arenes participate in aziridination with the optimized conditions: hydrocarbyl substituents (**3b** and **3c**), electron-donating alkoxy and Boc-protected amines (**3d** and **3e**), as well as various electron-withdrawing substituents (**3f**–**3k**) are all well tolerated. Both *meta*- and *ortho*-substituents (**3l**–**3o** and **3p**–**3r**, respectively) are compatible with the developed aziridination conditions and 2-vinylnaphthalene and 2-vinylbenzothiophene undergoes aziridination to **3s** and **3t** in 79% and 60% yield, respectively. For electron-neutral and electron-rich substrates, 5 mol% [TBA]I is utilized; for electron deficient substrates we increased the catalyst loading to 20 mol% to achieve efficient aziridination. Consistent with previous reports of iodide-catalyzed aziridination that are proposed to proceed via radical mechanisms, functionalization of 1,2-disubstituted olefins is not stereospecific^12^: aziridination of *trans*-β-methylstyrene (*trans*-**1u**) affords a 45:55 *cis*:*trans* mixture of **3u**; aziridination of *cis*-**1u** affords a 35:65 *cis*:*trans* mixture of **3u**. For geometrically constrained 1,2-disubstituted olefins, such as indene (**1v**), aziridination provides a single diastereomer (e.g., **3v** in 31% yield). The developed conditions are effective for the aziridination on more complex substrates, including **3w**–**3aa**, which are derived from pharmaceutically relevant estrone, ibuprofen, tufnil, probenecid, and isatin. Application to aliphatic olefins is available, albeit in lower efficiency: Aziridination of cyclohexene and allylbenzene with *N*-aminopyridinium triflate afforded the corresponding aziridines **3ab** and **3ac** in 33% and 16% yield, respectively.

**Fig. 3:**
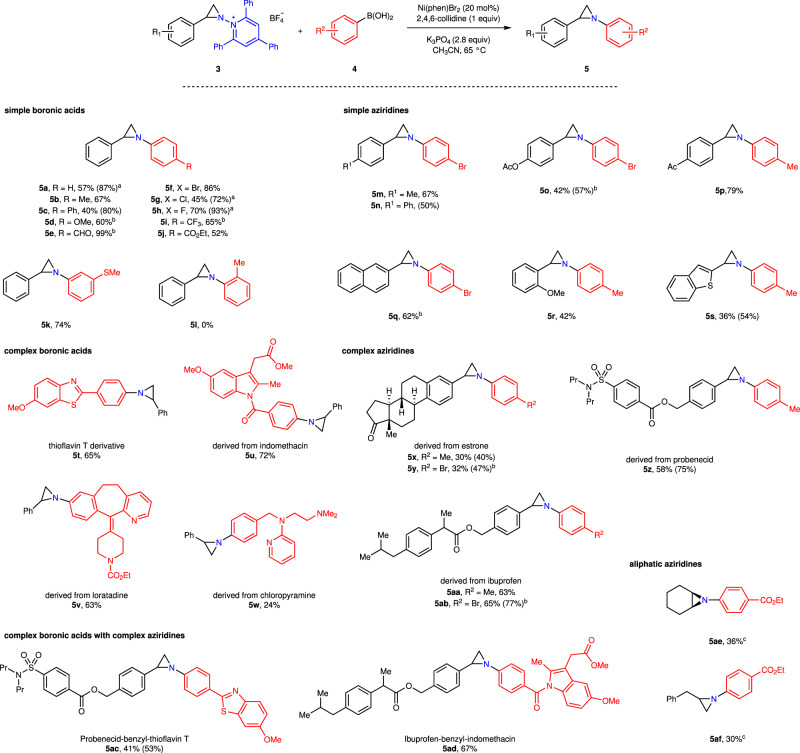
Ni-catalyzed cross-coupling of *N*-pyridinium aziridines 3. Cross-coupling of **3** with aryl boronic acids provides access to *N*-arylaziridines, which are the products of formal transfer of aryl nitrenes to olefins. **a** These reactions were carried out with 10 mol% Ni(phen)Br_2_. **b** For these substrates, K_2_CO_3_ was used in place of K_3_PO_4_. **c** Prepared from the unsubstituted *N*-pyridinium aziridine (i.e. **3aa** and **3ab**). Yields reported are based on isolated products (based on ^1^H NMR integration). Phen = 1,10-phenanthroline.

With conditions in hand to efficiently access *N*-pyridinium aziridines, we turned our attention to engaging these species as electrophiles in C–N coupling reactions. We envisioned a C–N cross coupling of *N*-pyridinium aziridines would (1) provide access to the products of formal nitrene transfer to olefins, (2) provide a rare example of an aziridine cross-coupling in which the aziridine ring remains intact, and (3) represent the first application of pyridinium electrophiles in C–N cross-coupling chemistry. We initiated our investigations by examining potential Ni-catalyzed cross coupling of *N*-pyridinium aziridine electrophiles with appropriate organometallic nucleophiles (i.e., Grignard reagents, organolithiums, organostannanes, and boronic acids). We identified that treatment of *N*-pyridinium aziridine **3a** with tolyl boroxine and NiCl_2_(dme) in MeCN afforded *N*-arylaziridine **5b** in 36% yield. The coupling efficiency is extremely sensitive to the Ni(II) counter anion: Under identical conditions, NiCl_2_ provided **5b** in 36% yield while NiBr_2_ afforded **5b** in 60% yield. Ni(OAc)_2_, Ni(acac)_2_, and NiSO_4_ salts were completely ineffective. Optimization of the cross-coupling reaction (see Supplementary Information Section C.2 for details) ultimately identified the use of NiBr_2_(phen) as catalyst in the presence of K_3_PO_4_ and 2,4,6-collidine provided *N*-tolylaziridine **5b** in 79% yield (Fig. 3). The catalyst loading could be reduced to 10 mol% without significant loss of yield, but further reduction to 5 mol% resulted in substantial reduction in reaction efficiency. For comparison, attempted cross-coupling reactions with *N*-phthalyl or *N*-sulfonyl aziridines under our optimized conditions or with N–H aziridines using C–N cross-coupling conditions described in the literature were unsuccessful (see Supplementary Information sections C.8 and C.9)^33, 34^. We also examined potential Ni-catalyzed cross-electrophile-coupling and found inferior results: Combination of **3a** with 4-bromotoluene in the presence of NiCl_2_ (30 mol%), 1,10-phenanthroline (30 mol%), and either Mn or Zn dust as terminal reductant afforded the product **5b** at 50% and 11% NMR yield, respectively (see Table S7)^60^.

The developed cross-coupling conditions enable cross-coupling with simple arylboronic acids substituted in the 4-position (**5a**–**5j**) and in the 3-position (**5k**), but substitution in the 2-position (**5l**) and alkyl boronic acids (i.e., *n*-butylboronic acid) were not tolerated. Notably, electron-neutral and electron-rich aryl groups can be incorporated in the *N*-arylaziridine efficiently, which represent specific challenges in direct nitrene transfer strategies^11, 15^. Similarly, various substitutions of the pyridinium aziridine coupling partner were also tolerated (**5m**–**5s**). The developed aziridination reaction is compatible with both complex boronic acids, such as those derived from thioflavin T (**5t**), indomethacin (**5u**), loratadine (**5v**), and chloropyramine (**5w**), and with complex pyridinium aziridine partners, such as those derived from estrone (**5x**–**5y**), probenecid (**5z**), and ibuprofen (**5aa**–**5ab**). Fragment coupling reactions in which both complex boronic acids and complex pyridinium aziridine partners are directly linked via an aziridine ring were efficient (**5ac**–**5ad**). Coupling of aziridines derived from aliphatic olefins is less efficient than coupling of vinyl arene-derived aziridines: Cross-coupling of **3ab** and **3ac** with (4-(ethoxycarbonyl)phenyl)boronic acid (**4j**) afforded aziridines **5ae** and **5af** in 36% and 30% yield, respectively.

**Fig. 4:**
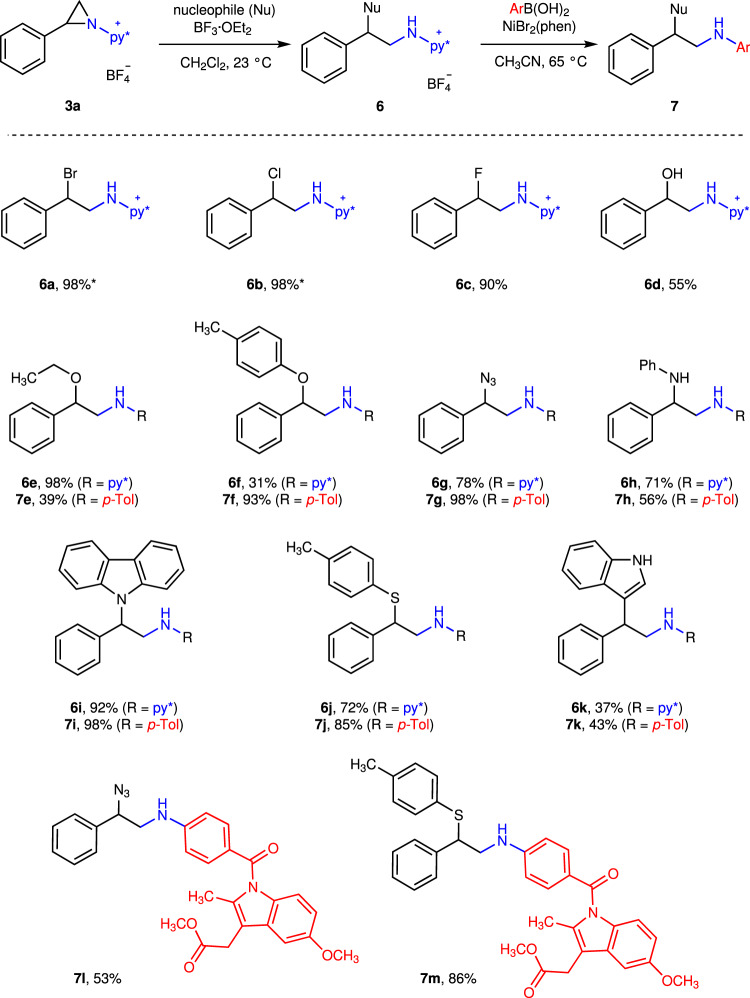
Derivatization of 3a via sequential ring-opening and cross-coupling. Nucleophilic opening of aziridine **3a** provides access to 1,2-aminofunctionalization products **6**. Ni-catalyzed cross-coupling of compounds **6** provides the opportunity to elaborate the resulting acyclic *N*-aminopyridinium derivatives to generate anilines **7**. *Yields determined by ^1^H NMR due to instability of these compounds towards intramolecular elimination.

In addition to direct C–N coupling with boronic acids, the developed *N*-pyridinium aziridines participate in ring-opening chemistry to access 1,2-difunctionalization products (Fig. 4). Exposure of *N*-pyridinium aziridine **3a** to halide sources (i.e. [TBA]Br, [TBA]Cl, or pyridine·HF) or H_2_O in the presence of BF_3_·OEt_2_ resulted in opening of the aziridine to afford 1,2-haloamine derivatives **6a**–**6c** or 1,2-hydroxyamine **6d**. Attempts to isolate **6a** and **6b** resulted in low isolated yields due to aziridine reclosure to *N*-pyridinium aziridine **3a** (vide infra). A variety of other oxygen-, nitrogen-, and sulfur-based nucleophiles participate in aziridine opening to afford isolable aminopyridinium derivatives **6e**–**6j**. Indole can serve as a carbon nucleophile to provide **6k** in 37% yield. These ring-opened compounds could be isolated as analytically pure materials and participate in efficient Ni-catalyzed cross coupling to generate 1,2-aminofunctionalized compounds **7e**–**7k** (the products of *p*-tolylboronic acid coupling), respectively. The ring-opened product **6g** and **6j** also participated in cross coupling with more complex boronic acids, as highlighted by the synthesis of **7l** and **7m**, which are derived from cross-coupling of ring-opened compounds with the boronic acid derived from indomethacin.

**Fig. 5:**
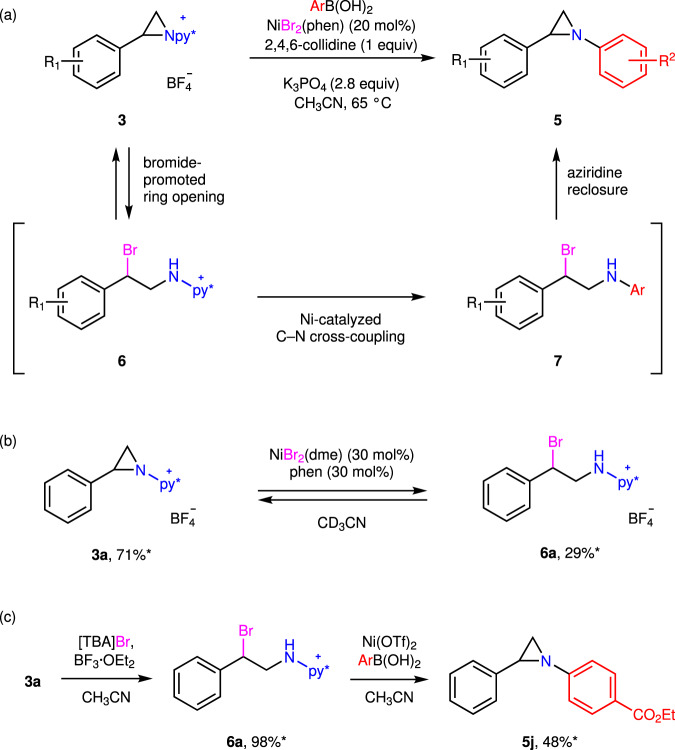
Summary of available mechanistic data for the cross-coupling of 3a. **a** Reversible halide-promoted aziridine opening, cross coupling, and aziridine reclosure are proposed to mediate C–N cross coupling of *N*-pyridinium aziridines. Consistent with this mechanism. **b** NiBr_2_ reacts with *N*-pyridinium aziridine **3a** to generate ring-opened **6a** and **c** exposure of ring-opened **6a** to Ni(OTf)_2_ or Ni(BF_4_)_2_ affords arylated aziridine **5j**. *Yields determined by ^1^H NMR spectroscopy.

Metal-catalyzed cross-coupling of aziridine often results in ring opening products^16–24^. In contrast, we observed Ni-catalyzed C–N coupling to generate *N*-arylaziridines in which the aziridine ring is conserved in the product. To better understand this unusual reaction outcome, we were interested in evaluating the mechanism of C–N bond-forming chemistry that leaves the three-membered aziridine ring intact. These investigations were guided by (1) a desire to understand the bromide-specific activity noted in our original catalyst optimization studies and (2) the observation that while treatment of **3a** with [TBA]Br and BF_3_·OEt_2_ resulted in ring opening, attempts to isolate the resulting benzyl bromide **6a** resulted in re-isolation of **3a**, which suggested that ring-opening with bromide is reversible. This observation suggested the possibility that aziridine opening, followed by cross-coupling of an open-chain aminopyridinium intermediates, and finally aziridine reclosure may be operative (Fig. 5a). Consistent with this hypothesis, treatment of aziridine **3a** with NiBr_2_(dme) (with or without added phenanthroline) results in the observation of ring-opened compound **6a** by ^1^H NMR (Fig. 5b). While BF_3_·OEt_2_ was required for ring opening with [TBA]Br, spontaneous ring-opening is observed upon addition of NiBr_2_, which suggest that under these conditions Ni^2+^ is serves as a cooperative Lewis acid activator and bromide source for aziridine opening. Finally, exposure of a sample of compound **6a** to Ni(OTf)_2_ or Ni(BF_4_)_2_ and *p-*ethoxylcarbonylphenylboronic acid results in the formation of *N*-arylaziridine **5j**, which demonstrates the viability of cross-coupling and aziridine reclosure (Fig. 5c). For further discussion of the impact of added bromide on cross-coupling efficiency, see Section C.7 of the Supplementary Information.

## Discussion

In summary, we report a strategy for the synthesis of *N-*aryl aziridines, which are the formal products of aryl nitrene addition to olefins. This method overcomes the inherent instability of free nitrene fragments by harnessing *N*-pyridinium aziridine intermediates that participate in Ni-catalyzed C–N cross-coupling. By decoupling the aziridination from installation of the *N*-substituent, this strategy overcomes the common requirement for difficult-to-remove *N*-substituents in aziridination chemistry. The observed C–N cross-coupling chemistry contrasts the typical reactivity pattern of *N*-sulfonylaziridine cross-coupling, which typically participate in ring-opening C–N activation, by taking advantage of a unique reversible ring opening/reclosure mechanism. These studies not only provide strategies to access products of formal nitrene transfer to olefins but significantly expand the synthetic scope of nitrene transfer by demonstrating *N-*aminopyridinium to be a bifunctional amination reagent.

## Methods

### General procedure for olefin aziridination

In an N_2_-filled dry box, a 20-mL scintillation vial was charged with 1-amino-2,4,6-triphenylpyridin-1-ium tetrafluoroborate (**2**, 82.0 mg, 0.200 mmol, 1.00 equiv), tetrabutylammonium iodide (3.7 mg, 0.010 mmol, 5 mol%), 4 Å molecular sieves, vinyl arene (**1**, 0.200 mmol, 1.00 equiv), and iodosylbenzene (44.0 mg, 0.200 mmol, 1.00 equiv). Acetonitrile (1.0 mL) was added, the vial was removed from the dry box, and the reaction mixture was stirred for 18 h. The reaction mixture was filtered through a pad of Celite and concentrated in vacuo. The residue was purified by silica gel flash chromatography (2:1 ethyl acetate:hexanes) to afford compound **3**.

### General procedure for cross-coupling of pyridinium aziridines

A 20-mL scintillation vial was charged with Ni(Phen)Br_2_ (20 mol%), base (2.8 equiv), aryl boronic acid (**4**, 2.4 equiv), pyridinium aziridine (**3**, 1 equiv) and a magnetic stir bar. In an N_2_ filled dry box, a solution of 2,4,6-collidine in acetonitrile (0.08 M, 1.0 equiv) was added to the scintillation vial with the rest of the reaction components. The reaction vial was heated at 65 °C for 36 h. After cooling to 23 °C, the reaction mixture was transferred to a centrifuge tube and centrifuged at 3220×*g* (6000 rpm) for 10 min. The supernatant was decanted. The residue was washed with CH_2_Cl_2_ and the combined supernatants were concentrated under reduced pressure and the crude mixture was purified as indicated in the Supplementary Information to afford the aziridine compound **5**.

### General procedure for nucleophilic opening of pyridinium aziridines

In an N_2_-filled dry box, a 20-mL scintillation vial was charged with 2,4,6-triphenyl-1-(2-phenylaziridin-1-yl)pyridinium tetrafluoroborate (**3**, 300 mg, 0.586 mmol, 1.00 equiv), BF_3_·OEt_2_ (0.075 mL, 0.59 mmol, 1.0 equiv), and CH_2_Cl_2_ (5 mL). A 25-mL Schlenk tube is charged with the appropriate nucleophile (0.703 mmol, 1.20 equiv). The CH_2_Cl_2_ solution of **3** and BF_3_·OEt_2_ was added to the Schlenk flask that contained the nucleophile. The resulting reaction mixture was allowed to stir at indicated temperature for indicated time. Solvent was removed under reduced pressure and the residue was purified by SiO_2_ gel chromatography (eluent 2:1 ethyl acetate:hexane) to afford the compound **6**.

### General procedure for cross-coupling of N-pyridinium amines **6**

In an N_2_-filled dry box, a 20-mL scintillation vial was charged with compound **6** (0.100 mmol, 1.00 equiv) and CH_3_CN (1 mL). A separate 20-mL was charged with NiBr_2_(dme) (3.1 mg, 0.010 mmol, 10 mol%), 1,10-phenanthroline (2.4 mg, 0.014 mmol, 0.14 equiv), K_3_PO_4_ (53.1 mg, 0.250 mmol, 2.50 equiv), *p*-toluyl boronic acid (20.4 mg, 0.150 mmol, 1.50 equiv). The CH_3_CN solution of **6** was added to the vial containing NiBr_2_·DME. The reaction mixture was stirred at 65 °C for 18 h. The reaction mixture was cooled to 23 °C. The reaction mixture was centrifuged at 3220×*g* (6000 rpm) for 10 min and the supernatant was decanted. Solvent was removed under reduced pressure and the residue was purified by alumina gel column chromatography to obtain compound **7**.

## Supplementary information

Supplementary Information

## Data Availability

All data generated in this study are provided in the Supplementary Information. Crystallography data for the structure reported in this Article have been deposited at the Cambridge Crystallographic Data Centre, under deposition number CCDC 2159504 (**3a**). Copies of the data can be obtained free of charge via https://www.ccdc.cam.ac.uk/structures/.
