# Supplementary material for: N-Aminopyridinium reagents as traceless activating groups in the synthesis of N-Aryl aziridines
Source: Nat Commun. 2022 Jun 10;13:3341. doi: 10.1038/s41467-022-31032-w (PMC9187731; doi:10.1038/s41467-022-31032-w)
Supplement: Supplementary file 1 — Supplementary Information [file 41467_2022_31032_MOESM1_ESM.pdf]

Supplementary Information

N-Aminopyridinium Reagents as Traceless Activating Groups in the Synthesis of *N*-Aryl Aziridines

Hao Tan, Samya Samanta, Asim Maity, Pritam Roychowdhury, and David C. Powers*

[a]*Department of Chemistry, Texas A&M University, College Station, TX 77843, USA*

Email: powers@chem.tamu.edu

Table of Contents

I. Supplementary Methods **S3**
A. General Considerations S3
A.1 Materials S3
A.2 Characterization Details S3
B. Synthesis and Characterization S5
B.1 Synthesis of 1-Aminopyridinium Salts S5
B.2 Preparation of Vinyl Arenes S7
B.3 Preparation of Ni(II) catalysts S9
B.4 Preparation of Aryl Boronic Acids S10
B.5 Vinyl Arene Aziridination S11
B.6 Cross-Coupling of Pyridinium Aziridines S21
B.7 Nucleophilic Opening of Pyridinium Aziridines S32
B.8 Cross-Coupling of *N*-Pyridinium Amines S37
II. Supplementary Discussion **S41**
C. Additional Data S41
C.1 Optimization of Vinyl Arene Aziridination S41
C.2 Optimization of Pyridinium Aziridine Cross-Couplings S45
C.3 Stereochemistry of Aziridination with *trans*- and *cis*-β-Methylstyrene S51
C.4 Observation of Pyridinium Aziridine Ring-Opening under Cross-Coupling Conditions S53
C.5 Ring-Closing and Cross-Coupling with NiX_2 S54
C.6 Attempts of Aliphatic Alkene Aziridination using *N*-Aminopyridinium Reagents S55
C.7 Cross-Coupling Reactions using 10 mol% of Nickel Catalyst S56
C.8 Failed Cross-Coupling of 2-(2-Phenylaziridin-1-yl)isoindoline-1,3-dione and 2-Phenyl-1-tosylaziridine S58
C.9 Cross-Coupling of 2-Phenylaziridine S60
C.10 Attempts to Observe Intermediates in Iodide-Catalyzed Aziridination S62
D. X-Ray Diffraction Data S64
E. NMR Spectra for New Compounds S66
III. Supplementary References **S209**

I. Supplementary Methods
A. General Considerations

A.1 Materials All chemicals and solvents were obtained as ACS reagent grade and used as received. Styrene, tetrafluoroboric acid etherate, and 2,4,6-collidine were acquired from BeanTown Chemical (BTC). 4-Fluorostyrene, 4-acetoxystyrene, 2-bromostyrene, 4-tolueneboronic acid, 4-chlorophenylboronic acid, 4-fluorophenylboronic acid, 4-trifluoromethylphenylboronic acid 4-ethoxycarbonylphenylboronic acid, and 2-methylphenylboronic acid were purchased from Matrix Scientific. Isatin, 4-trifluoromethylstyrene, 3-bromostyrene, indene, trifluoroboron etherate, *p*-thiocresol, hydrogen fluoride pyridine, 2-(4-isobutylphenyl)propanoic acid (Ibuprofen), 4-(dipropylsulfamoyl)benzoic acid (Probenecid) and *p*-biphenylboronic acid were acquired from Oakwood. Hexanes, ethyl acetate, dichloromethane, potassium phosphate, tetrabutylammonium iodide, anhydrous nickel(II) bromide, nickel(II) bromide dimethoxyethane complex, nickel(II) acetate tetrahydrate, 1,10-phenantroline, 4-methylstyrene, 4-bromophenylboronic acid, 3-(methylthio)phenylboronic acid, *p*-cresol, tetrabutylammonium bromide, tetrabutylammonium azide, carbazole, and chalcone were obtained from Sigma Aldrich. 4-Formylphenylboronic acid, 2-((3-chloro-2-methylphenyl)amino)benzoic acid (Tufnil), and loratadine were acquired from Ambeed. 4-Vinylaniline, 4-vinylbenzoic acid, 4-vinylbenzyl chloride, tetrabutylammonium chloride, and phenylboronic acid were acquired from TCI. 4-Methoxyphenylboronic acid and methyltriphenylphosphonium bromide were acquired from Chem Impex. 3-Nitrostyrene and *trans*-β-methylstyrene were acquired from Acros. Acetophenone, 2-aminopyridine, hydrazine monohydrate, aniline, *p*-methylbenzylamine, and benzyl mercaptan were acquired from Alfa Aesar. Anhydrous sodium sulfate and anhydrous potassium carbonate were obtained from VWR. Acetonitrile and methanol were obtained from Fischer Scientific. *cis*-β-Methylstyrene was acquired from ChemCruz Chemicals. Chloropyramine hydrochloride was acquired from Thermo Scientific. Dry dichloromethane and acetonitrile (purchased from Fisher scientific, HPLC grade) were obtained from a drying column and stored over activated 4 Å molecular sieves.[1] NMR solvents were purchased from Cambridge Isotope Laboratories and were used as received. All reactions were carried out under ambient atmosphere unless otherwise noted. *tert*-Butyl (4-vinylphenyl)carbamate (**1e**),[2] 1-(4-vinylphenyl)ethan-1-one (**1g**),[3] ethyl 4-vinylbenzoate (**1i**),[4] (8R,9S,13S,14S)-13-methyl-3-vinyl-6,7,8,9,11,12,13,14,15,16-decahydro-17H-cyclopenta[a]phenanthren-17-one (**1w**),[5] 4-vinylbenzyl 2-(4-isobutylphenyl)propanoate (**1x**),[6] methyl 2-(methyl(2-methyl-3-vinylphenyl)amino)benzoate (**1y**),[7] 4-vinylbenzyl 4-(*N*,*N*-dipropylsulfamoyl)benzoate (**1z**),[6] and (4-(6-methoxybenzo[d]thiazol-2-yl)phenyl)boronic acid[8] were prepared according to literature procedures.

A.2 Characterization Details ^{1}H and ^{13}C NMR spectral acquisitions were recorded on an Inova 500 FT NMR (Varian), a VNMRS 500 FT NMR (Varian), or an Acsend™ 400 NMR (Bruker) and were referenced against residual proteo solvent signals: $CDCl_3$ (7.26 ppm, ^{1}H; 77.16 ppm, ^{13}C) and acetonitrile-d_3 (1.94 ppm, ^{1}H). ^{1}H NMR data are reported as follows: chemical shift (δ, ppm), (multiplicity: s (singlet), d (doublet), t (triplet), m (multiplet), br (broad), integration). ^{13}C NMR data are reported as follows: chemical shift (δ, ppm). Mass

spectrometry data were recorded on either Orbitrap Fusion™ Tribrid™ Mass Spectrometer or Q Exactive™ Focus Hybrid Quadrupole-Orbitrap™ Mass Spectrometer from ThermoFisher Scientific. Preparative HPLC was carried out by Agilent 1260 Infinity II Preparative LC System, Agilent 1290 II Preparative Open-Bed Fraction Collector, using an Agilent Prep-C18 column (50 × 50 mm, 5 μm pore, part# 446905-502). HPLC grade acetonitrile was purchased from Sigma Aldrich and was used as the eluent. Experimental details of crystallization are included in the synthetic procedures for the relevant compounds. A Bruker APEX 2 Duo X-ray (three-circle) diffractometer was used for crystal screening, unit cell determination, and data collection for the X-ray crystal structures of **3a**. Crystal suitable for X-ray diffraction were mounted on a MiTeGen dual-thickness micro-mount and placed under a cold N2 stream (Oxford). The X-ray radiation employed was generated from a Mo sealed X-ray tube (Kα = 0.70173 Å with a potential of 40 kV and a current of 40 mA). Bruker AXS APEX II software was used for data collection and reduction. Absorption corrections were applied using the 4 program SADABS. A solution was obtained using XT/XS in APEX2 and refined in Olex2.[9-11] Hydrogen atoms were placed in idealized positions and were set riding on the respective parent atoms. All non- hydrogen atoms were refined with anisotropic thermal parameters. The structure was refined (weighted least squares refinement on F2) to convergence.[11]

B. Synthesis and Characterization

B.1 Synthesis of 1-Aminopyridinium Salts

Synthesis of 2,4,6-triphenyl-*N*-aminopyridinium tetrafluoroborate (2)

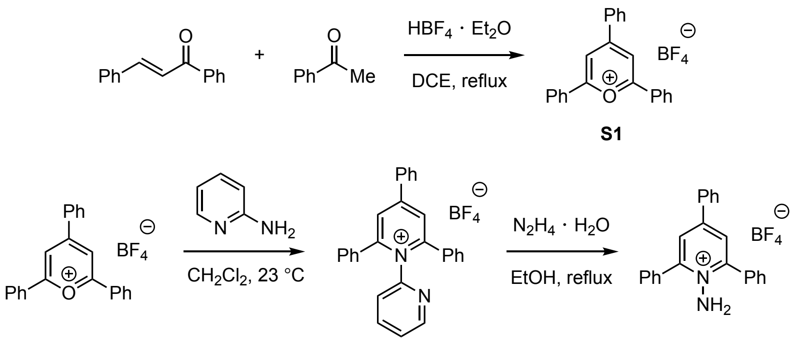

S1 **S2** **2**

2,4,6-Triphenylpyrylium tetrafluoroborate (**S1**) was synthesized according to the following literature procedure.[12] A 2-L round bottom flask was charged with chalcone (68.8 g, 33.2 mmol, 2.00 equiv), acetophenone (20.0 g, 16.6 mmol, 1.00 equiv), and dichloroethane (DCE, 120 mL). Tetrafluoroboric acid etherate (46 mL, 33.2 mmol, 2.00 equiv) was added dropwise using an addition funnel. The reaction mixture was heated to reflux for 4 h. The reaction mixture was cooled to 23 °C before diethyl ether was added and the crude mixture was filtered. The obtained yellow solid was washed with diethyl ether (50 × 5 mL) and dried under vacuum to yield 45.0 g of compound **S1** (68% yield). ^{1}H NMR (499 MHz, CD_3CN) δ 8.72 (s, 2H), 8.46–8.40 (m, 4H), 8.33–8.28 (m, 2H), 7.90–7.84 (m, 3H), 7.81–7.75 (m, 6H).

1-Amino-2,4,6-triphenylpyridin-1-ium tetrafluoroborate (**2**) was according to the following literature procedure.[13] To a suspension of 2,4,6-triphenylpyrylium tetrafluoroborate (10.0 g, 25.3 mmol, 1.00 equiv) in dichloromethane (253 mL, 0.100 M) was added 2-aminopyridine (3.57 g, 38.0 mmol, 1.50 equiv). The reaction was stirred at 23 °C for 12 h before solvent was removed under reduced pressure. Addition of Et_2O to the residue resulted in crystallization of 11.6 g of **S2** (97% yield). ^{1}H NMR (400 MHz, $CDCl_3$) δ 8.23 (ddd, *J* = 4.9, 1.9, 0.8 Hz, 1H), 8.11 (s, 2H), 7.96–7.87 (m, 2H), 7.80–7.73 (m, 1H), 7.65–7.47 (m, 8H), 7.38–7.24 (m, 6H), 7.12 (ddd, *J* = 7.5, 4.8, 1.0 Hz, 1H).

To a suspension of **S2** (9.99 g, 21.2 mmol, 1.00 equiv) in ethanol (70 mL) was added hydrazine monohydrate (5.36 g, 106 mmol, 5.00 equiv). The reaction is heated under reflux for 4 h. The reaction mixture was cooled to 23 °C before a small amount of diethyl ether (1.0 mL) was added. The reaction mixture was cooled to −20 °C at which the target product crystallized. The obtained solid was recrystallized from CH_2Cl_2 and Et_2O and then dried under vacuum at 70 °C to afford 5.81 g of the title compound (67% yield). ^{1}H NMR (400 MHz, $CDCl_3$) δ 7.85 (s, 2H), 7.86–7.76 (m, 4H), 7.77–7.70 (m, 2H), 7.66–7.57 (m, 6H), 7.60–7.50 (m, 3H), 6.20 (s, 2H).

Synthesis of *N*-aminopyridinium triflate (*S*3)

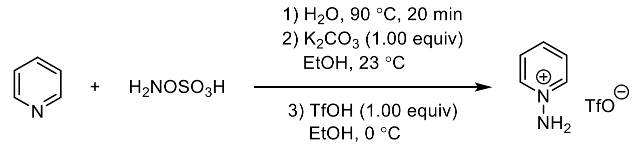

N-Aminopyridinium triflate (**S3**) was prepared according to the literature procedure.[14] To a freshly prepared solution of hydroxylamine-*O*-sulfonic acid (11.3 g, 10.0 mmol, 1.00 equiv) in 64 mL of H_2O in a 250-mL flask was added pyridine (24.0 mL, 30.0 mmol, 1.00 equiv). The reaction was heated at 90 °C for 20 minutes and cooled to 23 °C, followed by addition of potassium carbonate (13.8 g, 10.0 mmol, 1.00 equiv). The mixture was concentrated under reduced pressure and 120 mL of ethanol was added. The resulting suspension was filtered and TfOH (15.0 g, 8.80 mL, 10.0 mmol, 1.00 equiv) was added dropwise at 0 °C. The solution was concentrated under reduced pressure and crystalized at -20 °C. The precipitated was filtered and dried under vacuum to give the title compound **S3** as an off-white solid (16.5 g, 67% yield). ^{1}H NMR (400 MHz, CD_3CN) δ 8.57 (d, J = 6.2 Hz, 2H), 8.28 (t, J = 7.7 Hz, 1H), 7.92 (t, J = 7.3 Hz, 2H), 7.11 (s, 2H). ^{19}F NMR (377 MHz, CD_3CN) δ –79.1.

B.2 Preparation of Vinyl Arenes

General Procedure A[15]

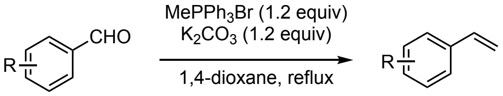

4-Vinylbenzonitrile (**1j**), 3-methylstyrene (**1l**), and 2-methylstyrene (**1p**) were synthesized according to the following procedure. Characterization data were well-matched to those reported in literature.

A 50-mL round bottom flask was charged with methyltriphenylphosphonium bromide (3.57 g, 10.0 mmol, 1.20 equiv), potassium carbonate (1.80 g, 13.0 mmol, 1.57 equiv), the appropriate benzaldehyde (8.30 mmol, 1.00 equiv), and 1,4-dioxane (10 mL). The reaction mixture was heated to reflux for 24 h. The reaction mixture was cooled to 23 °C, filtered, and the filtrate was concentrated under reduced pressure. The residue purified by silica gel column chromatography using 100% hexanes as the eluent to afford the corresponding styrene.

General Procedure B[16]

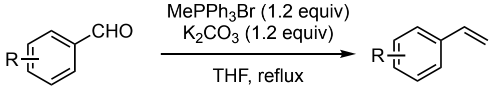

4-Vinylanisole (**1d**) and 2-vinylnaphthalene (**1s**) were synthesized according to the following procedure. Characterization data were well-matched to those reported in literature.

A 50-mL Schlenk flask was charged with methyltriphenylphosphonium bromide (2.70 g, 7.69 mmol, 1.20 equiv), potassium carbonate (1.30 g, 9.60 mmol, 1.50 equiv) and the corresponding benzaldehyde (6.40 mmol, 1.00 equiv) under N_2. Anhydrous tetrahydrofuran (15 mL) was added and the reaction mixture was heated at reflux for 48 h. The reaction was cooled to 23 °C, filtered, and the filtrate was concentrated under reduced pressure. The residue was purified by silica gel column chromatography using 100% hexanes as the eluent to give the corresponding styrene.

Gerenal Procedure C[17]

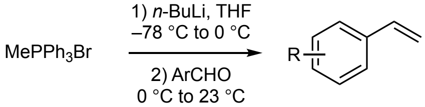

4-Vinyl-1,1'-biphenyl (**1c**), 3-chlorostyrene (**1n**), 2-vinylanisole (**1q**), and 2-vinylbenzo[b]thiophene (**1t**) were synthesized according to the following procedure. Characterization data were well-matched to those reported in literature.

A 50-mL Schlenk flask was charged with methyltriphenylphosphonium bromide (1.70 g, 4.75 mmol, 1.20 equiv) and tetrahydrofuran (35 mL). *n*-Butyl lithium (2.5 M, 4.40 mmol, 1.8 mL, 1.10 equiv) was added dropwise at –78 °C under N_2. The reaction mixture was warmed to 0 °C at which temperature it was stirred for 2h. The appropriate benzaldehyde was then added dropwise at 0 °C.[1] The reaction was allowed to stir at 23 °C overnight. Saturated aqueous NH_4Cl (35 mL) was added. The mixture was extracted with ethyl acetate (20 × 3 mL), washed with brine, dried over anhydrous sodium sulfate, and concentrated under reduced pressure. The residue was purified by silica gel column chromatography using 100% hexanes or 5% ethyl acetate in hexanes as the eluent to give the corresponding styrene.

Synthesis of 1-(4-vinylbenzyl)indoline-2,3-dione (1aa)

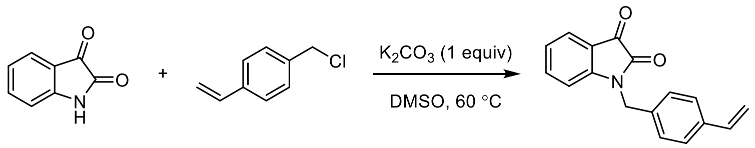

1aa

A 50-mL round bottom flask was charged with indoline-2,3-dione (13.6 mmol, 2.00 g, 1.00 equiv), potassium carbonate (13.6 mmol, 1.88 g, 1.00 equiv), and DMSO (45 mL). 1-(Chloromethyl)-4-vinylbenzene (4.20 g, 27.5 mmol, 2.02 equiv) was added and the reaction mixture was heated at 60 °C for 24 h. The reaction was cooled to 23 °C before water (40 mL) and EtOAc (40 mL) were added. The organic layer was washed with distilled water (30 mL × 5) and then brine (30 mL), dried over anhydrous sodium sulfate, and concentrated under reduced pressure. The residue was purified by silica gel column chromatography using 10% EtOAc in hexanes as the eluent to yield a red solid (2.69 g, 75% yield). ^{1}H NMR (400 MHz, $CDCl_3$) δ 7.60 (d, *J* = 7.6 Hz, 1H), 7.47 (td, *J* = 7.8, 1.4 Hz, 1H), 7.38 (d, *J* = 8.2 Hz, 2H), 7.29 (d, *J* = 8.1 Hz, 2H), 7.08 (t, *J* = 7.5 Hz, 1H), 6.77 (d, *J* = 7.9 Hz, 1H), 6.68 (dd, *J* = 17.6, 10.9 Hz, 1H), 5.73 (d, *J* = 17.6 Hz, 1H), 5.25 (d, *J* = 10.8 Hz, 1H), 4.91 (s, 2H). ^{13}C NMR (101 MHz, $CDCl_3$) δ 183.3, 158.4, 150.8, 138.4, 137.7, 136.2, 134.0, 127.8, 127.0, 125.5, 124.0, 117.8, 114.7, 111.1, 43.9. HRMS-ESI$^+$ (m/z): [M+1]+ calcd. for C17H14NO2+, 264.1019; found, 264.1019.

[1] If the benzaldehyde is a solid, it was added to the reaction mixture as a solution in tetrahydrofuran.

B.3 Preparation of Nickel(II) Catalysts

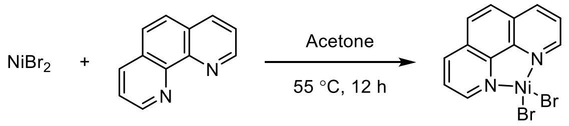

Ni(Phen)Br_2 was prepared according to the following modification of literature methods.[18] A 20-mL scintillation vial was charged with anhydrous nickel(II) bromide (218 mg, 1.00 mmol, 1.00 equiv) and acetone (2 mL). 1,10-Phenanthroline (180 mg, 1.00 mmol, 1.00 equiv) was dissolved in acetone (4 mL) and added to the reaction mixture. The reaction mixture was heated at 55 °C for 12 h. The reaction was cooled to 23 °C before the resulting green suspension was concentrated under reduced pressure. The residue was dried under vacuum at 65 °C to yield a blue-green powder (392 mg, 98% yield). ^{1}H NMR (400 MHz, DMSO) δ 49.6 (s, 2H), 45.7 (s, 2H), 25.2 (s, 2H), 23.5 (s, 2H), 18.9–19.2, (m, 4H). These spectral data are well-matched to those reported in the literature.[19]

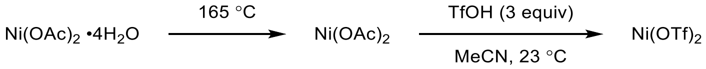

Ni(OTf)$_2$ was prepared according to the following modification of literature methods.[20] Nickel(II) acetate tetrahydrate was first heated under air at 165 °C for overnight to afford anhydrous nickel(II) acetate. A 100-mL Schlenk flask was charged with anhydrous nickel(II) acetate (354 mg, 2.00 mmol, 1.00 equiv) and acetonitrile (25 mL) under N_2. Trifluoromethanesulfonic acid (900 mg, 6.00 mmol, 3.00 equiv) was added via syringe dropwise. The reaction was stirred at 23 °C for 2 h to give a blue solution. The reaction was concentrated to ~6 mL under reduced pressure. Diethyl ether (20 mL) was added and blue solid precipitated. The liquid was decanted and the solid was washed with diethyl ether and hexanes. The solid was dried under vacuum at 70 °C to yield a light green powder (454 mg, 64% yield).

B.4 Preparation of Aryl boronic acids[2]

General Procedure[21]

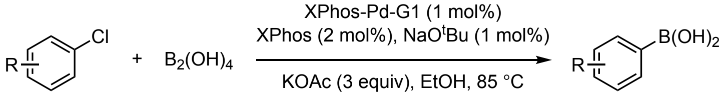

Boronic acids **4a** (derivatized from indomethacin methyl ester)[21] and **4b** (derived from loratadine)[21] were synthesized according to the procedure above and characterization data matched those reported in literature.

A 50-mL sealed tube was charged with the appropriate aryl chloride (2.75 mmol, 1.00 equiv), hypodiboric acid (370 mg, 4.13 mmol, 1.50 equiv), XPhos-Pd-G1 (20.3 mg, 0.00275 mmol, 1 mol%), XPhos (26.2 mg, 0.0550 mmol, 2 mol%), sodium *tert*-butoxide (2.6 mg, 0.0550 mmol, 2 mol%), and potassium acetate (810 mg, 8.25 mmol, 3.00 equiv). Ethanol (15 mL) was added under N_2. The reaction was heated at 85 °C for 3 h. After cooling to 23 °C, the reaction mixture was filtered through a pad of celite and the filtrate was concentrated under reduced pressure. The residue was dissolved in 5 mL of CH_2Cl_2 and the solution was added dropwise to a 250-mL beaker containing 100 mL of hexane. The resulting precipitate was isolated by filtraton and dried under vacuum to yield the respective boronic acids.

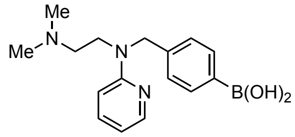

4c

(4-(((2-(dimethylamino)ethyl)(pyridin-2-yl)amino)methyl)phenyl)boronic acid (**4c**). Obtained from chloropyramine (840 mg, 2.90 mmol) according to the general procedure and obtained as a white solid (367 mg, 42% yield). ^1H NMR (400 MHz, DMSO-d_6) δ 8.11–8.05 (m, 1H), 7.96 (s, 2H), 7.75–7.68 (m, 2H), 7.43 (ddd, *J* = 8.9, 7.1, 2.0 Hz, 1H), 7.17 (d, *J* = 7.8 Hz, 2H), 6.57–6.49 (m, 2H), 4.74 (s, 2H), 3.62 (t, *J* = 7.1 Hz, 2H), 2.45 (t, *J* = 7.0 Hz, 2H), 2.19 (s, 6H). ^{13}C NMR (101 MHz, DMSO) δ 157.7, 147.6, 141.0, 137.3, 134.3, 125.8, 111.6, 105.7, 56.5, 51.2, 45.9, 45.4. HRMS-ESI$^+$ (m/z): [M+1]+ calcd. for C16H23BN3O2+, 300.1878; found, 300.1878.

---

[2] Simple boronic acids acquired from commercial sources contains varied amount of the corresponding acid anhydrides. 4-Toluene boronic acid acquired from Matrix, contained ~90% of *p*-tolylboroxine indicated by ^1H NMR spectroscopy. Heating a sample at 120 °C under air for 48 h resulted in a sample of the boroxine containing less than 5% boronic acid. Other boronic acids were used as received.

B.5 Vinyl Arene Aziridination

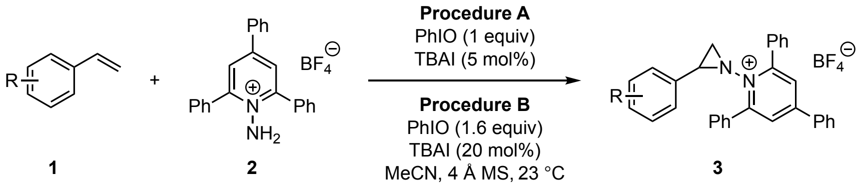

Procedure A In an N_2 filled dry box, a 20-mL scintillation vial was charged with 1-amino-2,4,6-triphenylpyridin-1-ium tetrafluoroborate (**2**, 82.0 mg, 0.200 mmol, 1.00 equiv), tetrabutylammonium iodide (3.7 mg, 0.010 mmol, 5 mol%), 4 Å molecular sieves, vinyl arene (**1**, 0.200 mmol, 1.00 equiv),[3] and iodosylbenzene (44.0 mg, 0.200 mmol, 1.00 equiv). Acetonitrile (1.0 mL) was added, the vial was removed from the dry box, and the reaction mixture was stirred for 18 h. The reaction mixture was filtered through a pad of Celite and concentrated in vacuo. The residue was purified by silica gel flash chromatography (2:1 ethyl acetate:hexanes) to afford the title compound.

Procedure B In an N_2 filled dry box, a 20-mL scintillation vial was charged with 1-amino-2,4,6-triphenylpyridin-1-ium tetrafluoroborate (**2**, 131 mg, 0.320 mmol, 1.60 equiv), tetrabutylammonium iodide (14.8 mg, 0.0400 mmol, 20 mol%), 4 Å molecular sieves, vinyl arene (**1**, 0.200 mmol, 1.00 equiv, if is a solid), and iodosylbenzene (70.4 mg, 0.320 mmol, 1.60 equiv).[3] Acetonitrile (1.0 mL) was added, the vial was removed from the dry box, and the reaction mixture was stirred for 18 h. The reaction mixture was filtered through a pad of Celite and concentrated in vacuo. The residue was purified by silica gel flash chromatography (2:1 ethyl acetate:hexanes) to afford the title compound.

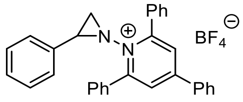

3a

*2,4,6-triphenyl-1-(2-phenylaziridin-1-yl)pyridin-1-ium tetrafluoroborate (**3a**)*. Prepared via Procedure A from styrene (**1a**) and obtained as a pale-yellow powder (72.8 mg, 71% yield). The crystalline sample used for the diffraction experiment was obtained via pentane diffusion into an EtOAc/DCM (1:1) solution. ^{1}H NMR (400 MHz, CD_3CN) δ 8.14 (s, 2H), 8.04–7.92 (m, 5H), 7.68–7.25 (m, 9H), 7.23–7.17 (m, 1H), 7.12 (t, *J* = 7.5 Hz, 2H), 6.72–6.57 (m, 2H), 3.52 (dd, *J* = 8.4, 5.7 Hz, 1H), 2.69 (dd, *J* = 8.4, 3.1 Hz, 1H), 2.36 (dd, *J* = 5.7, 3.1 Hz, 1H). ^{13}C NMR (101 MHz, CD_3CN) δ 154.2, 154.0, 134.9, 134.8, 132.8, 132.6, 132.4, 130.8, 130.6, 130.0, 129.1, 128.8, 127.1, 126.4, 54.5, 49.0. ^{19}F NMR (376 MHz, CD_3CN) δ –151.92, –151.97. HRMS-ESI$^+$ (m/z): [M]+ calcd. for C31H25N2+, 425.2012; found, 425.2007.

[3] If the vinyl arene is a solid, it is added to the reaction mixture neat. If the vinyl arene is a liquid, it is added to the reaction mixture as a solution in CH_3CN.

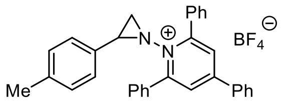

3b

*2,4,6-triphenyl-1-(2-(p-tolyl)aziridin-1-yl)pyridin-1-ium tetrafluoroborate (**3b**)*. Prepared via Procedure A from 4-methylstyrene (**1b**) and obtained as a yellow powder (75.8 mg, 72% yield). ^{1}H NMR (400 MHz, CD_3CN) δ 8.14 (s, 2H), 8.07–7.85 (m, 6H), 7.73–7.18 (m, 9H), 6.99–6.90 (m, 2H), 6.60–6.48 (m, 2H), 3.48 (dd, *J* = 8.4, 5.8 Hz, 1H), 2.65 (dd, *J* = 8.4, 3.1 Hz, 1H), 2.34 (dd, *J* = 5.8, 3.1 Hz, 1H), 2.26 (s, 3H). ^{13}C NMR (101 MHz, CD_3CN) δ 154.2, 153.9, 139.1, 134.9, 132.8, 132.6, 132.5, 131.7, 130.8, 130.6, 130.0, 129.4, 129.1, 127.1, 126.4, 54.5, 48.9, 21.1. ^{19}F NMR (377 MHz, CD_3CN) δ –151.98, –152.03. HRMS-ESI^{+} (m/z): [M]+ calcd. for C32H27N2+, 439.2169; found, 439.2166.

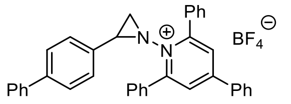

3c

*1-(2-([1,1'-biphenyl]-4-yl)aziridin-1-yl)-2,4,6-triphenylpyridin-1-ium tetrafluoroborate (**3c**)*. Prepared via Procedure A from 4-vinyl-1,1'-biphenyl (**1c**) and obtained as a yellow powder (83.6 mg, 71% yield). ^{1}H NMR (400 MHz, CD_3CN) δ 8.15 (s, 2H), 8.07–7.95 (m, 6H), 7.72–7.32 (m, 16H), 6.73 (d, *J* = 8.3 Hz, 2H), 3.55 (dd, *J* = 8.4, 5.7 Hz, 1H), 2.71 (dd, *J* = 8.4, 3.1 Hz, 1H), 2.43 (dd, *J* = 5.8, 3.1 Hz, 1H). ^{13}C NMR (101 MHz, CD_3CN) δ 154.2, 154.0, 141.7, 141.2, 134.9, 134.0, 132.9, 132.6, 132.5, 130.8, 130.6, 130.1, 129.8, 129.1, 128.5, 127.8, 127.3, 127.1, 127.0, 54.4, 48.9. ^{19}F NMR (377 MHz, CD_3CN) δ –151.94, –152.00. HRMS-ESI^{+} (m/z): [M] +calcd. for C37H29N2+, 501.2325; found, 501.2319.

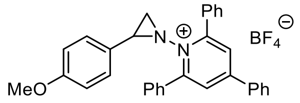

3d

*1-(2-(4-methoxyphenyl)aziridin-1-yl)-2,4,6-triphenylpyridin-1-ium tetrafluoroborate (**3d**)*. Prepared via Procedure A from 4-vinylanisole (**1d**) and obtained as a yellow powder (61.8 mg, 57% yield). ^{1}H NMR (400 MHz, CD_3CN) δ 8.14 (s, 2H), 8.04–7.88 (m, 6H), 7.72–7.35 (m, 9H), 6.67 (d, *J* = 8.8 Hz, 2H), 6.55 (d, *J* = 8.7 Hz, 2H), 3.74 (s, 3H), 3.48 (dd, *J* = 8.4, 5.8 Hz, 1H), 2.64 (dd, *J* = 8.4, 3.1 Hz, 1H), 2.35 (dd, *J* = 5.9, 3.2 Hz, 1H). ^{13}C NMR (101 MHz, CD_3CN) δ 160.7, 154.2, 153.9, 134.9, 132.9, 132.7, 132.5, 130.8, 130.6, 130.0, 129.1, 127.8, 127.1, 126.6, 114.2, 55.9, 54.5, 48.6. ^{19}F NMR (377 MHz, CD_3CN) δ –152.15, -152.21. HRMS-ESI^{+} (m/z): [M]+ calcd. for C32H27N2O+, 455.2118; found, 455.2113.

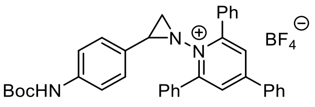

3e

*1-(2-(4-((tert-butoxycarbonyl)amino)phenyl)aziridin-1-yl)-2,4,6-triphenylpyridin-1-ium tetrafluoroborate (**3e**)*. Prepared via Procedure A from tert-butyl (4-vinylphenyl)carbamate (**1e**) and obtained as a yellow powder (64.0 mg, 51% yield). ^{1}H NMR (400 MHz, CD_3CN) δ 8.14 (s, 2H), 7.98 (ddd, *J* = 19.0, 7.6, 2.3 Hz, 6H), 7.69–7.35 (m, 10H), 7.19–7.12 (m, 2H), 6.58–6.51 (m, 2H), 3.46 (dd, *J* = 8.4, 5.8 Hz, 1H), 2.63 (dd, *J* = 8.4, 3.1 Hz, 1H), 2.33 (dd, *J* = 5.8, 3.1 Hz, 1H), 1.49 (s, 9H). ^{13}C NMR (101 MHz, CD_3CN) δ 154.2, 153.9, 153.7, 140.3, 134.9, 132.9, 132.6, 132.5, 130.8, 130.6, 130.2, 130.0, 129.1, 128.6, 127.1, 127.1, 80.6, 54.5, 48.7, 28.4. ^{19}F NMR (376 MHz, CD_3CN) δ –152.30, –152.35. HRMS-ESI^{+} (m/z): [M]+ calcd. for C36H34N3O2+, 540.2646; found, 540.2646.

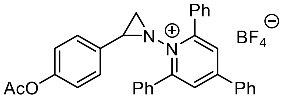

3f

*1-(2-(4-acetoxyphenyl)aziridin-1-yl)-2,4,6-triphenylpyridin-1-ium tetrafluoroborate (**3f**)*. Prepared via Procedure A from 4-vinylphenyl acetate (**1f**) and obtained as a yellow powder (67.5 mg, 59% yield). ^{1}H NMR (400 MHz, CD_3CN) δ 8.16 (s, 2H), 8.05–7.82 (m, 6H), 7.71–7.31 (m, 9H), 6.89–6.81 (m, 2H), 6.73–6.65 (m, 2H), 3.50 (dd, *J* = 8.4, 5.7 Hz, 1H), 2.68 (dd, *J* = 8.4, 3.1 Hz, 1H), 2.40 (dd, *J* = 5.7, 3.1 Hz, 1H), 2.23 (s, 3H). ^{13}C NMR (101 MHz, CD_3CN) δ 170.4, 154.2, 154.1, 151.8, 134.9, 132.9, 132.6, 132.5, 132.4, 130.8, 130.6, 130.1, 129.1, 127.6, 127.1, 122.3, 54.0, 49.0, 21.2. ^{19}F NMR (376 MHz, CD_3CN) δ –152.00, –152.06. HRMS-ESI^{+} (m/z): [M]+ calcd. for C33H27N2O2+, 483.2067; found, 483.2069.

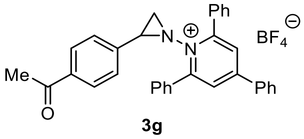

3g

*1-(2-(4-acetylphenyl)aziridin-1-yl)-2,4,6-triphenylpyridin-1-ium tetrafluoroborate (**3g**)*. Prepared via Procedure A from 1-(4-vinylphenyl)ethan-1-one (**1g**) and obtained as a yellow powder (72.1 mg, 65% yield). ^{1}H NMR (400 MHz, CD_3CN) δ 8.15 (s, 2H), 7.99 (m, 6H), 7.77–6.99 (m, 11H), 6.83–6.77 (m, 2H), 3.57 (dd, *J* = 8.4, 5.7 Hz, 1H), 2.75 (dd, *J* = 8.4, 3.0 Hz, 1H), 2.53 (s, 3H), 2.42 (dd, *J* = 5.7, 3.0 Hz, 1H). ^{13}C NMR (101 MHz, CD_3CN) δ 198.5, 154.1, 154.1, 140.0, 137.7, 134.8, 132.8, 132.5, 132.4, 130.7, 130.6, 130.0, 129.1, 128.6, 127.1, 126.6, 53.9, 49.3, 26.9. ^{19}F NMR (377 MHz, CD_3CN) δ –152.30, –152.35. HRMS-ESI^{+} (m/z): [M]+ calcd. for C33H27N2O+, 467.2118; found, 467.2110.

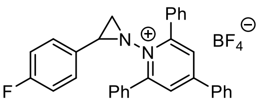

3h

*1-(2-(4-fluorophenyl)aziridin-1-yl)-2,4,6-triphenylpyridin-1-ium tetrafluoroborate (**3h**)*. Prepared via Procedure B from 4-fluorostyrene (**1h**) and obtained as a brown powder (88.0 mg, 83% yield). ^{1}H NMR (400 MHz, CD_3CN) δ 8.14 (s, 2H), 8.04–7.88 (m, 6H), 7.67–7.22 (m, 9H), 6.92–6.81 (m, 2H), 6.73–6.61 (m, 2H), 3.53 (dd, *J* = 8.4, 5.7 Hz, 1H), 2.69 (dd, *J* = 8.4, 3.1 Hz, 1H), 2.35 (dd, *J* = 5.7, 3.1 Hz, 1H). ^{13}C NMR (101 MHz, CD_3CN) δ 163.5 (d, $^{1}J_{C\text{–}F}$ = 244 Hz), 154.2, 154.1, 134.9, 132.9, 132.5, 132.5, 131.0, 130.8, 130.6, 130.0, 129.1, 128.5 (d, $^{3}J_{C\text{–}F}$ = 9 Hz), 127.1, 115.6 (d $^{2}J_{C\text{–}F}$ = 22 Hz), 53.8, 48.9. ^{19}F NMR (377 MHz, CD_3CN) δ –115.20, –152.37, –152.42. HRMS-ESI^{+} (m/z): [M]+ calcd. for C31H24FN2+, 443.1918; found, 443.1912.

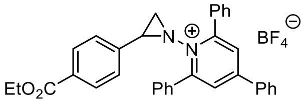

3i

*1-(2-(4-(ethoxycarbonyl)phenyl)aziridin-1-yl)-2,4,6-triphenylpyridin-1-ium tetrafluoroborate (**3i**)*. Prepared via Procedure B from ethyl 4-vinylbenzoate (**1i**) and obtained as a brown powder (94.7 mg, 81% yield). ^{1}H NMR (400 MHz, CD_3CN) δ 8.16 (s, 2H), 8.05–7.88 (m, 6H), 7.78–7.70 (m, 2H), 7.68–7.27 (m, 9H), 6.83–6.76 (m, 2H), 4.32 (q, *J* = 7.1 Hz, 2H), 3.57 (dd, *J* = 8.4, 5.7 Hz, 1H), 2.74 (dd, *J* = 8.4, 3.0 Hz, 1H), 2.40 (dd, *J* = 5.7, 3.1 Hz, 1H), 1.35 (t, *J* = 7.1 Hz, 3H). ^{13}C NMR (101 MHz, CD_3CN) δ 166.4, 153.9, 153.8, 139.7, 134.5, 132.6, 132.2, 132.1, 130.9, 130.5, 130.3, 129.7, 129.3, 128.8, 126.8, 126.3, 61.5, 53.6, 49.1, 14.2. ^{19}F NMR (377 MHz, CD_3CN) δ –152.46, –152.52. HRMS-ESI^{+} (m/z): [M]+ calcd. for, C34H29N2O2+, 497.2224; found, 497.2223.

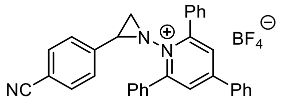

3j

*1-(2-(4-cyanophenyl)aziridin-1-yl)-2,4,6-triphenylpyridin-1-ium tetrafluoroborate (**3j**)*. Prepared via Procedure B from 4-vinylbenzonitrile (**1j**) and obtained as a brown powder (96.7 mg, 90% yield). ^{1}H NMR (400 MHz, CD_3CN) δ 8.17 (s, 2H), 8.06–7.79 (m, 6H), 7.67–7.37 (m, 11H), 6.89–6.82 (m, 2H), 3.54 (dd, *J* = 8.1, 5.9 Hz, 1H), 2.75 (dd, *J* = 8.4, 3.1 Hz, 1H), 2.43 (dd, *J* = 5.7, 3.2 Hz, 1H). ^{13}C NMR (101 MHz, CD_3CN) δ 154.3, 154.2, 140.4, 134.8, 133.0, 132.6, 132.6, 132.4, 130.8, 130.6, 130.1, 129.1, 127.4, 127.2, 119.4, 112.4, 53.6, 49.4. ^{19}F NMR (377 MHz, CD_3CN) δ –151.98, –152.03. HRMS-ESI^{+} (m/z): [M]+ calcd. for C32H24N3+, 450.1965; found, 450.1955.

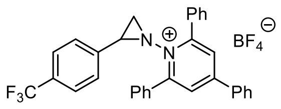

3k

*2,4,6-triphenyl-1-(2-(4-(trifluoromethyl)phenyl)aziridin-1-yl)pyridin-1-ium tetrafluoroborate (**3k**)*. Prepared via Procedure B from 4-(trifluoromethyl)styrene (**1k**) and obtained as a brown powder (24.4 mg, 21% yield). ^{1}H NMR (400 MHz, CD_3CN) δ 8.17 (s, 2H), 8.05–7.85 (m, 6H), 7.76–7.05 (m, 11H), 6.91–6.85 (m, 2H), 3.57 (dd, J = 8.4, 5.6 Hz, 1H), 2.75 (dd, J = 8.4, 3.1 Hz, 1H), 2.44 (dd, J = 5.7, 3.1 Hz, 1H). ^{13}C NMR (101 MHz, CD_3CN) δ 154.2, 154.1, 139.48, 139.47, 134.8, 132.8, 132.4, 130.8, 130.5, 130.4 (q, $^2J_{C-F}$ = 31.9 Hz), 130.0, 129.1, 127.2, 127.1, 125.6 (q, $^3J_{C-F}$ = 3.8 Hz), 125,2 (q, $^1J_{C-F}$ = 271 Hz), 53.6, 49.2. ^{19}F NMR (376 MHz, CD_3CN) δ –63.14, –152.02, –152.07. HRMS-ESI$^+$ (m/z): [M]+ calcd. for C32H24F3N2+, 493.1886; found, 493.1874.

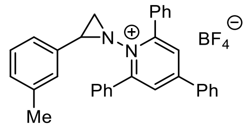

3l

*2,4,6-triphenyl-1-(2-(m-tolyl)aziridin-1-yl)pyridin-1-ium tetrafluoroborate (**3l**)*. Prepared via Procedure B from 3-methylstyrene (**1l**) and obtained as a brown powder (78.9 mg, 75% yield). ^{1}H NMR (400 MHz, CD_3CN) δ 8.13 (s, 2H), 8.07–7.91 (m, 6H), 7.70–7.21 (m, 9H), 7.04–6.98 (m, 2H), 6.50–6.44 (m, 1H), 6.39 (s, 1H), 3.47 (dd, J = 8.4, 5.8 Hz, 1H), 2.67 (dd, J = 8.4, 3.1 Hz, 1H), 2.37 (dd, J = 5.8, 3.1 Hz, 1H), 2.21 (s, 3H). ^{13}C NMR (101 MHz, CD_3CN) δ 154.2, 154.0, 138.4, 134.9, 134.6, 132.8, 132.6, 132.4, 130.8, 130.6, 130.0, 129.8, 129.1, 128.7, 127.7, 127.1, 123.0, 54.6, 48.6, 21.0. ^{19}F NMR (376 MHz, CD_3CN) δ –151.75, –151.81. HRMS-ESI$^+$ (m/z): [M]+ calcd. for C32H27N2+, 439.2169; found, 439.2161.

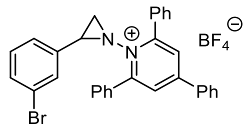

3m

*1-(2-(3-bromophenyl)aziridin-1-yl)-2,4,6-triphenylpyridin-1-ium tetrafluoroborate (**3m**)*. Prepared via Procedure B from 3-bromostyrene (**1m**) and obtained as a brown powder (76.8 mg, 65% yield). ^{1}H NMR (400 MHz, CD_3CN) δ 8.16 (s, 2H), 8.05–7.89 (m, 6H), 7.69–7.40 (m, 9H), 7.36 (ddd, J = 8.0, 2.0, 1.0 Hz, 1H), 7.06 (t, J = 7.9 Hz, 1H), 6.80 (t, J = 1.9 Hz, 1H), 6.71–6.64 (m, 1H), 3.46 (dd, J = 8.4, 5.7 Hz, 1H), 2.69 (dd, J = 8.4, 3.2 Hz, 1H), 2.42 (dd, J = 5.7, 3.2 Hz, 1H). ^{13}C NMR (101 MHz, CD_3CN) δ 154.2, 137.3, 134.8, 132.9, 132.6, 132.4, 132.1, 130.8, 130.7, 130.6, 130.0, 129.7, 129.1, 127.1, 125.2, 122.3, 53.7, 48.8. ^{19}F NMR (377 MHz, CD_3CN) δ –152.27, –152.33. HRMS-ESI$^+$ (m/z): [M]+ calcd. for C31H24BrN2+, 503.1117; found, 503.1109.

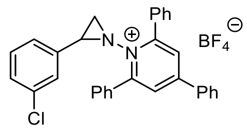

3n

*1-(2-(3-chlorophenyl)aziridin-1-yl)-2,4,6-triphenylpyridin-1-ium tetrafluoroborate (**3n**)*. Prepared via Procedure A from 3-chlorostyrene (**1n**) and obtained as a yellow powder (53.6 mg, 49% yield). ^{1}H NMR (400 MHz, CD_3CN) δ 8.15 (s, 2H), 7.98 (m, 6H), 7.71–7.28 (m, 9H), 7.21 (ddd, *J* = 8.0, 2.1, 1.1 Hz, 1H), 7.11 (t, *J* = 7.8 Hz, 1H), 6.68–6.59 (m, 2H), 3.49 (dd, *J* = 8.4, 5.7 Hz, 1H), 2.70 (dd, *J* = 8.4, 3.1 Hz, 1H), 2.41 (dd, *J* = 5.7, 3.1 Hz, 1H). ^{13}C NMR (101 MHz, CD_3CN) δ 154.2, 154.1, 137.2, 134.8, 134.2, 132.9, 132.5, 132.4, 130.8, 130.6, 130.4, 130.0, 129.1, 129.1, 127.1, 126.7, 124.8, 53.7, 48.8. ^{19}F NMR (377 MHz, CD_3CN) δ –152.33, –152.38. HRMS-ESI^{+} (m/z): [M]+ calcd. for C31H24ClN2+, 459.1623; found, 459.1614.

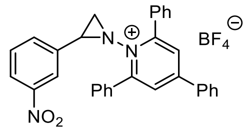

3o

*1-(2-(3-nitrophenyl)aziridin-1-yl)-2,4,6-triphenylpyridin-1-ium tetrafluoroborate (**3o**)*. Prepared via Procedure B from 3-nitrostyrene (**1o**) and obtained as a light-brown powder (83.5 mg, 75% yield). ^{1}H NMR (400 MHz, CD_3CN) δ 7.91 (s, 2H), 7.81–7.55 (m, 7H), 7.46–7.01 (m, 11H), 6.87 (d, *J* = 7.7 Hz, 1H), 3.39 (dd, *J* = 8.4, 5.7 Hz, 1H), 2.52 (dd, *J* = 8.4, 3.2 Hz, 1H), 2.25 (dd, *J* = 5.7, 3.3 Hz, 1H). ^{13}C NMR (101 MHz, CD_3CN) δ 154.3, 154.1, 148.6, 137.1, 134.8, 132.9, 132.6, 132.5, 132.4, 130.8, 130.6, 130.2, 130.1, 129.1, 127.2, 124.0, 121.7, 53.4, 49.0. ^{19}F NMR (377 MHz, CD_3CN) δ –151.98, –152.03. HRMS-ESI^{+} (m/z): [M]+ calcd. for C31H24N3O2+, 470.1863; found, 470.1852.

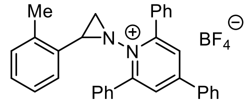

3p

*2,4,6-triphenyl-1-(2-(o-tolyl)aziridin-1-yl)pyridin-1-ium tetrafluoroborate (**3p**)*. Prepared via Procedure B from 2-methylstyrene (**1p**) and obtained as a brown powder (88.4 mg, 84% yield). ^{1}H NMR (400 MHz, CD_3CN) δ 8.15 (s, 2H), 8.06–7.91 (m, 6H), 7.69–7.28 (m, 9H), 7.07 (m, 2H), 6.96–6.85 (m, 2H), 3.77 (dd, *J* = 8.4, 5.7 Hz, 1H), 2.72 (dd, *J* = 8.4, 2.7 Hz, 1H), 2.15 (dd, *J* = 5.7, 2.7 Hz, 1H), 1.85 (s, 3H). ^{13}C NMR (101 MHz, CD_3CN) δ 154.2, 153.9, 136.4, 134.9, 133.4, 132.8, 132.5, 132.2, 130.7, 130.6, 130.4, 129.9, 129.1, 128.5, 127.1, 126.7, 124.4, 51.4, 50.1, 18.6. ^{19}F NMR (377 MHz, CD_3CN) δ –151.93, –151.98. HRMS-ESI^{+} (m/z): [M]+ calcd. for C32H27N2+, 439.2169; found, 439.2164.

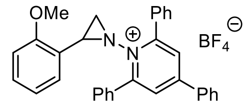

3q

*1-(2-(2-methoxyphenyl)aziridin-1-yl)-2,4,6-triphenylpyridin-1-ium tetrafluoroborate (**3q**)*. Prepared via Procedure A from 2-vinylanisole (**1q**) and obtained as a yellow powder (66.2

mg, 61% yield). ^{1}H NMR (400 MHz, CD_3CN) δ 8.13 (s, 2H), 8.04–7.85 (m, 6H), 7.70–7.23 (m, 9H), 7.15 (ddd, *J* = 8.3, 6.9, 2.2 Hz, 1H), 6.89–6.77 (m, 2H), 6.65 (d, *J* = 8.3 Hz, 1H), 3.96 (dd, *J* = 8.5, 5.9 Hz, 1H), 3.54 (s, 3H), 2.69 (dd, *J* = 8.5, 2.8 Hz, 1H), 2.28–2.23 (m, 1H). ^{13}C NMR (101 MHz, CD_3CN) δ 158.0, 154.2, 153.9, 134.9, 132.8, 132.4, 132.2, 130.7, 130.6, 130.0, 129.7, 129.1, 127.1, 125.5, 122.8, 121.0, 110.9, 55.7, 49.3, 48.8. ^{19}F NMR (376 MHz, CD_3CN) δ –152.37, –152.42. HRMS-ESI$^+$ (m/z): [M]+ calcd. for C32H27N2O+, 455.2118; found, 455.2110.

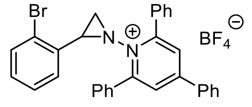

3r

*1-(2-(2-bromophenyl)aziridin-1-yl)-2,4,6-triphenylpyridin-1-ium tetrafluoroborate (**3r**).* Prepared via Procedure B from 2-bromostyrene (**1r**) and obtained as a brown powder (89.9 mg, 76% yield). ^{1}H NMR (400 MHz,CD_3CN) δ 8.17 (s, 2H), 8.05–7.90 (m, 6H), 7.71–7.36 (m, 9H), 7.32–7.23 (m, 2H), 7.14–7.07 (m, 1H), 7.01 (dd, *J* = 7.7, 1.7 Hz, 1H), 3.98 (dd, *J* = 8.4, 5.6 Hz, 1H), 2.75 (dd, *J* = 8.4, 2.8 Hz, 1H), 2.22 (dd, *J* = 5.6, 2.8 Hz, 1H). ^{13}C NMR (101 MHz, CD_3CN) δ 154.3, 154.2, 134.8, 134.4, 132.9, 132.9, 132.3, 132.3, 130.7, 130.6, 130.6, 130.1, 129.1, 128.4, 127.2, 126.8, 123.0, 53.6, 49.9. ^{19}F NMR (377 MHz, CD_3CN) δ –152.14, –152.19. HRMS-ESI$^+$ (m/z): [M]+ calcd. for C31H24BrN2+, 503.1117; found, 503.1116.

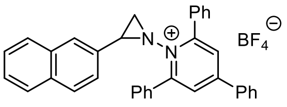

3s

*1-(2-(naphthalen-2-yl)aziridin-1-yl)-2,4,6-triphenylpyridin-1-ium tetrafluoroborate (**3s**).* Prepared via Procedure A from 2-vinylnaphthalene (**1s**) and obtained as a yellow powder (89.0 mg, 79% yield). ^{1}H NMR (400 MHz, CD_3CN) δ 8.14 (s, 2H), 8.08–7.96 (m, 6H), 7.85–7.77 (m, 1H), 7.72–7.16 (m, 13H), 7.08 (d, *J* = 1.8 Hz, 1H), 6.82 (dd, *J* = 8.5, 1.8 Hz, 1H), 3.68 (dd, *J* = 8.4, 5.8 Hz, 1H), 2.73 (dd, *J* = 8.4, 3.2 Hz, 1H), 2.47 (dd, *J* = 5.8, 3.1 Hz, 1H). ^{13}C NMR (101 MHz, CD_3CN) δ 154.2, 154.0, 134.9, 134.0, 133.4, 132.8, 132.6, 132.4, 132.1, 130.8, 130.6, 130.0, 129.1, 128.6, 128.5, 128.4, 127.3, 127.2, 127.1, 126.8, 123.1, 54.8, 48.6. ^{19}F NMR (376 MHz, CD_3CN) δ –151.94, –152.00. HRMS-ESI$^+$ (m/z): [M]+ calcd. for C35H27N2+, 475.2169; found, 475.2159.

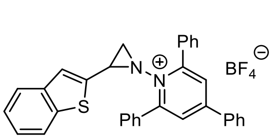

3t

*1-(2-(benzo[b]thiophen-2-yl)aziridin-1-yl)-2,4,6-triphenylpyridin-1-ium tetrafluoroborate (**3t**).* Prepared via Procedure A from 2-vinylbenzo[b]thiophene (**1t**) and obtained as a yellow powder (68.2 mg, 60% yield). ^{1}H NMR (400 MHz, CD_3CN) δ 7.86 (s, 2H), 7.76–7.67 (m, 6H), 7.58–7.46 (m, 1H), 7.42–7.08 (m, 10H), 7.08–6.99 (m, 2H), 6.26 (s, 1H), 3.69 (dd, *J* = 8.3, 5.7 Hz, 1H), 2.51 (dd, *J* = 8.3, 3.3 Hz, 1H), 2.12 (dd, *J* = 5.7, 3.3 Hz, 1H). ^{13}C NMR (101 MHz, CD_3CN) δ 154.3, 154.1, 139.8, 139.5, 138.6, 134.8, 132.9, 132.6, 132.5, 130.8, 130.6, 130.1, 129.1,

127.2, 125.7, 125.5, 124.9, 124.3, 123.1, 52.0, 49.7. ^{19}F NMR (376 MHz, CD_3CN) δ –152.04, –152.09. HRMS-ESI$^+$ (m/z): [M]+ calcd. for C33H25N2S+, 481.1773; found, 481.1727.

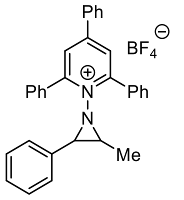

cis-**3u**

*1-(2-methyl-3-phenylaziridin-1-yl)-2,4,6-triphenylpyridin-1-ium tetrafluoroborate (cis-**3u**)*. Prepared via Procedure A from *trans*-β-methylstyrene. Only the *cis*-isomer was isolated in analytical purity and obtained as a yellow solid (21.2 mg, 20% yield). ^{1}H NMR (400 MHz, CD_3CN) δ 8.12 (s, 2H), 8.04–7.85 (m, 6H), 7.68–7.35 (m, 9H), 7.22–7.09 (m, 3H), 6.88–6.81 (m, 2H), 3.66 (d, *J* = 8.7 Hz, 1H), 2.96 (dq, *J* = 8.7, 5.9 Hz, 1H), 0.18 (d, *J* = 6.0 Hz, 3H).

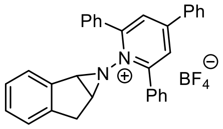

3v

*1-(6,6a-dihydroindeno[1,2-b]azirin-1(1aH)-yl)-2,4,6-triphenylpyridin-1-ium tetrafluoroborate (**3v**).* Prepared via Procedure A from indene on 2.0 mmol scale and obtained as a brown powder (321.7 mg, 31% yield). ^{1}H NMR (400 MHz, CD_3CN) δ 8.17 (s, 2H), 8.06 – 7.91 (m, 6H), 7.85 – 7.71 (m, 6H), 7.68 – 7.60 (m, 3H), 7.21 (td, *J* = 7.5, 1.2 Hz, 1H), 7.13 – 7.09 (m, 1H), 7.02 (td, *J* = 7.5, 1.0 Hz, 1H), 6.11 (d, *J* = 7.5 Hz, 1H), 4.06 (d, *J* = 6.3 Hz, 1H), 3.56 (dd, *J* = 6.0, 4.8 Hz, 1H), 2.69 (dd, *J* = 18.1, 4.9 Hz, 1H), 2.47 (d, *J* = 18.3 Hz, 1H). ^{13}C NMR (101 MHz, CD_3CN) δ 154.7, 154.2, 144.9, 137.15, 134.9, 133.4, 132.9, 132.8, 130.6, 130.6, 130.5, 130.2, 129.1, 127.4, 127.2, 126.6, 126.0, 63.0, 58.7, 34.6. ^{19}F NMR (377 MHz, CD_3CN) δ -151.7, -151.7. HRMS-ESI$^+$ (m/z): [M]+ calcd. for C32H25N2+, 437.2012; found, 437.2006.

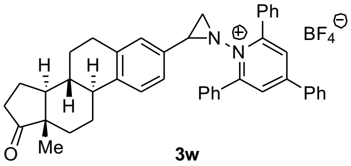

3w

*1-(2-((8R,9S,13S,14S)-13-methyl-17-oxo-7,8,9,11,12,13,14,15,16,17-decahydro-6H-cyclopenta[a]phenanthren-3-yl)aziridin-1-yl)-2,4,6-triphenylpyridin-1-ium tetrafluoroborate (**3w**)*. Prepared via Procedure A from (8R,9S,13S,14S)-13-methyl-3-vinyl-6,7,8,9,11,12,13,14,15,16-decahydro-17H-cyclopenta[a]phenanthren-17-one (**1w**, 1.05 mmol) on 1.05 mmol scale and obtained as a yellow powder (453.3 mg, 63% yield). ^{1}H NMR (400 MHz, CD_3CN) δ 8.15 (s, 2H), 8.08–7.78 (m, 6H), 7.75–7.14 (m, 9H), 7.07 (d, *J* = 8.1 Hz, 1H), 6.44 (d, *J* = 8.1 Hz, 1H), 6.23 (s, 1H), 3.48–3.33 (m, 1H), 2.71 (m, 2H), 2.62 (dd, *J* = 8.4, 3.2 Hz, 1H), 2.52–2.30 (m, 3H), 2.23 (d, *J* = 9.5 Hz, 1H), 2.12–1.97 (m, 4H), 1.86 (dt, *J* = 9.2, 2.8 Hz, 1H), 1.71–1.30 (m, 6H), 0.90 (d, *J* = 13.1 Hz, 3H). ^{13}C NMR (101 MHz, CD_3CN) δ 220.9, 154.2, 153.9, 141.2, 137.0, 134.9, 132.8, 132.6, 132.5, 131.6, 130.8, 130.6, 130.0, 129.1, 128.0, 127.1, 125.8, 122.9, 54.7, 51.1, 48.6, 48.3, 45.0, 38.9, 36.2, 32.4, 29.7, 27.0, 26.5, 22.1, 14.2.^{19}F

NMR (377 MHz, CD_3CN) δ –151.89, –151.95. HRMS-ESI+ (m/z): [M]+ calcd. for C43H41N2O+, 601.3213; found, [M+] = 601.3220.

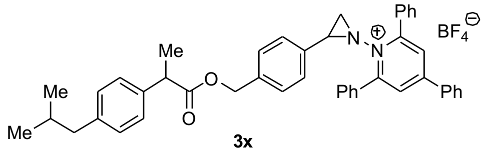

3x

*1-(2-(4-(((2-(4-isobutylphenyl)propanoyl)oxy)methyl)phenyl)aziridin-1-yl)-2,4,6-triphenylpyridin-1-ium tetrafluoroborate (**3x**)*. Prepared via Procedure A from 4-vinylbenzyl 2-(4-isobutylphenyl)propanoate (**1x**, 1.90 mmol) on 1.9 mmol scale and obtained as an orange powder (785.7 mg, 57% yield). ^{1}H NMR (400 MHz, CD_3CN) δ 8.15 (s, 2H), 8.07–7.79 (m, 6H), 7.71–7.27 (m, 9H), 7.21–7.24 (m, 2H), 7.18–7.10 (m, 2H), 6.95 (dd, *J* = 8.3, 1.7 Hz, 2H), 6.58 (dd, *J* = 8.3, 2.2 Hz, 2H), 5.16–4.86 (m, 2H), 3.79 (q, *J* = 7.1 Hz, 1H), 3.44 (dd, *J* = 8.4, 5.8 Hz, 1H), 2.66 (dd, *J* = 8.4, 3.1 Hz, 1H), 2.47 (dd, *J* = 7.2, 2.8 Hz, 1H), 2.36 (ddd, *J* = 5.8, 3.1, 1.3 Hz, 1H), 1.85 (dtd, *J* = 13.6, 6.8, 2.2 Hz, 1H), 1.46 (d, *J* = 7.2 Hz, 3H), 0.89 (dd, *J* = 6.6, 2.0 Hz, 3H). ^{13}C NMR (101 MHz, CD_3CN) δ 175.0, 154.2, 154.1, 141.6, 139.1, 137.7, 134.9, 134.5, 132.9, 132.5, 132.5, 130.8, 130.6, 130.3, 130.0, 129.1, 128.2, 128.1, 128.0, 127.1, 126.5, 66.38, 66.36, 54.3, 49.0, 45.8, 45.7, 45.4, 31.0, 22.5, 18.8, 18.7. ^{19}F NMR (376 MHz, CD_3CN) δ –151.85, –151.91. HRMS-ESI+ (m/z): [M]+ calcd. for C45H43N2O2+, 643.3391; found, 643.3318.

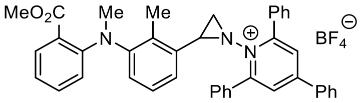

3y

*1-(2-(3-((2-(methoxycarbonyl)phenyl)(methyl)amino)-2-methylphenyl)aziridin-1-yl)-2,4,6-triphenylpyridin-1-ium tetrafluoroborate (**3y**)*. Prepared via Procedure A from methyl 2-(methyl(2-methyl-3-vinylphenyl)amino)benzoate (**1y**, 0.30 mmol) on 0.3 mmol scale and obtained as a brown powder (154.2 mg, 82% yield). ^{1}H NMR (400 MHz, CD_3CN) δ 8.17 (s, 2H), 8.03–7.90 (m, 6H), 7.70–7.36 (m, 11H), 7.33 (dd, *J* = 8.0, 1.7 Hz, 1H), 7.02 (t, *J* = 7.9 Hz, 1H), 6.94–6.84 (m, 2H), 6.77 (d, *J* = 7.8 Hz, 1H), 6.70 (d, *J* = 7.8 Hz, 1H), 3.76 (dd, *J* = 8.4, 5.8 Hz, 1H), 3.19 (s, 3H), 3.00 (s, 3H), 2.72 (dd, *J* = 8.3, 2.5 Hz, 1H), 2.20 (dd, *J* = 5.7, 2.7 Hz, 1H), 1.76 (s, 3H). ^{13}C NMR (126 MHz, $CDCl_3$) δ 169.1, 154.2, 152.9, 149.1, 148.9, 134.9, 134.9, 133.7, 132.1, 131.8, 131.6, 131.2, 129.8, 129.7, 129.2, 128.2, 127.7, 127.5, 127.4, 126.3, 124.7, 122.9, 120.0, 119.7, 118.1, 51.5, 51.1, 49.8, 41.6, 13.6. ^{19}F NMR (377 MHz, CD_3CN) δ –151.95, –152.00. HRMS-ESI+ (m/z): [M]+ calcd. for C41H36N3O2+, 602.2802; found, 602.2804.

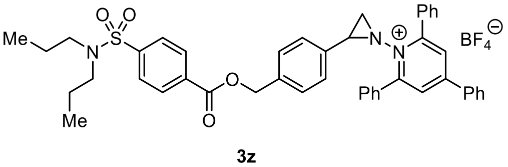

3z

*1-(2-(4-(((4-(N,N-dipropylsulfamoyl)benzoyl)oxy)methyl)phenyl)aziridin-1-yl)-2,4,6-triphenylpyridin-1-ium tetrafluoroborate (**3z**)*. Prepared via Procedure A from 4-vinylbenzyl 4-(N,N-dipropylsulfamoyl)benzoate (**1z**, 1.6 mmol) on 1.6 mmol scale and obtained as a yellow powder (547.9 mg, 42% yield). ^{1}H NMR (400 MHz, CD_3CN) δ 8.21–8.13 (m, 4H), 8.05–7.83 (m, 8H), 7.71–7.28 (m, 9H), 7.23 (d, *J* = 8.1 Hz, 2H), 6.70 (d, *J* = 8.2 Hz, 2H), 5.30 (s, 2H),

3.50 (dtd, J = 8.3, 4.0, 1.9 Hz, 1H), 3.17–3.02 (m, 4H), 2.69 (dt, J = 8.4, 3.2 Hz, 1H), 2.41 (ddd, J = 6.1, 3.2, 1.5 Hz, 1H), 1.51 (h, J = 7.4 Hz, 4H), 0.83 (t, J = 7.4 Hz, 6H). ^{13}C NMR (101 MHz, CD_3CN) δ 165.8, 154.2, 154.1, 145.3, 137.2, 135.0, 134.9, 134.5, 132.9, 132.5, 131.1, 130.8, 130.6, 130.0, 129.1, 128.8, 128.2, 127.1, 126.7, 67.6, 54.3, 50.7, 49.0, 22.6, 11.3. ^{19}F NMR (377 MHz, CD_3CN) δ –151.93, –151.99. HRMS-ESI$^+$ (m/z): [M]+ calcd. for C45H44N3O4S+, 722.3047; found, 722.3045.

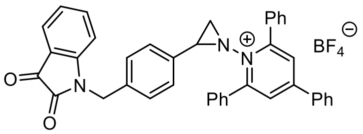

3aa

*1-(2-(4-((2,3-dioxoindolin-1-yl)methyl)phenyl)aziridin-1-yl)-2,4,6-triphenylpyridin-1-ium tetrafluoroborate (**3aa**)*. Prepared via Procedure A from 1-(4-vinylbenzyl)indoline-2,3-dione (**1aa**)and obtained as a red powder (90.0 mg, 67% yield). ^1H NMR (400 MHz, CD_3CN) δ 8.12 (s, 2H), 8.05–7.83 (m, 6H), 7.72–7.20 (m, 11H), 7.17 (t, J = 7.6 Hz, 1H), 7.12 (d, J = 8.0 Hz, 2H), 6.86 (d, J = 8.0 Hz, 2H), 4.90–4.74 (m, 2H), 3.48 (dd, J = 8.4, 5.8 Hz, 1H), 2.69 (dd, J = 8.4, 3.1 Hz, 1H), 2.37 (dd, J = 5.8, 3.1 Hz, 1H). ^{13}C NMR (101 MHz, CD_3CN) δ 184.6, 159.3, 154.0, 151.6, 139.1, 136.4, 134.8, 134.5, 132.8, 132.5, 132.4, 130.7, 130.6, 130.5, 130.0, 129.1, 127.8, 127.0, 126.9, 125.5, 124.7, 118.7, 112.2, 54.2, 48.9, 43.9. ^{19}F NMR (376 MHz, CD_3CN) δ –151.56, –151.61. HRMS-ESI$^+$ (m/z): [M]+ calcd. for C40H30N3O2+, 584.2333; found, 584.2335.

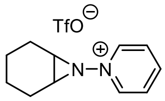

3ab

*1-(7-azabicyclo[4.1.0]heptan-7-yl)pyridin-1-ium trifluoromethanesulfonate (**3ab**)*. Prepared via Procedure A from cyclohexene and 1-aminopyridinium triflate (**S3**) on 0.8 mmol scale, purified using 10% methanol in dichloromethane and obtained as an orange oil (84.3 mg, 33% yield). ^1H NMR (400 MHz, CD_3CN) δ 8.84 – 8.80 (m, 2H), 8.33 – 8.26 (m, 1H), 7.98 – 7.86 (m, 2H), 3.42 – 3.27 (m, 2H), 2.23 – 2.13 (m, 2H), 2.09 – 2.00 (m, 2H), 1.44 – 1.31 (m, 4H). ^{13}C NMR (101 MHz, CD_3CN) δ 143.7, 141.2, 129.2, 47.4, 23.3, 20.2. ^{19}F NMR (377 MHz, CD_3CN) δ -79.3. HRMS-ESI$^+$ (m/z): [M]+ calcd. for C11H15N2+, 175.1230; found, 175.1229.

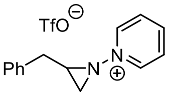

3ac

*1-(2-benzylaziridin-1-yl)pyridin-1-ium trifluoromethanesulfonate (**3ac**).* Prepared via Procedure A from allylbenzene and 1-aminopyridinium triflate (**S3**) on 1.2 mmol scale, purified using 10% methanol in dichloromethane and obtained as a brown oil (67.6 mg, 16% yield). ^1H NMR (400 MHz, CD_3CN) δ 8.62–8.55 (m, 2H), 8.29 (tt, J = 7.7, 1.2 Hz, 1H), 7.92–7.86 (m, 2H), 7.44–7.32 (m, 5H), 3.36 (dq, J = 8.3, 6.2 Hz, 1H), 3.15–3.02 (m, 3H), 2.87 (dd, J = 5.7, 2.9 Hz, 1H). ^{13}C NMR (101 MHz, CD_3CN) δ 144.1, 141.0, 137.8, 130.1, 129.8, 129.3, 128.1, 48.6, 40.9, 37.3. ^{19}F NMR (377 MHz, CD_3CN) δ -79.3. HRMS-ESI$^+$ (m/z): [M]+ calcd. for C14H15N2+, 211.1230; found, 211.1225.

B.6 Cross-Coupling of Pyridinium Aziridines

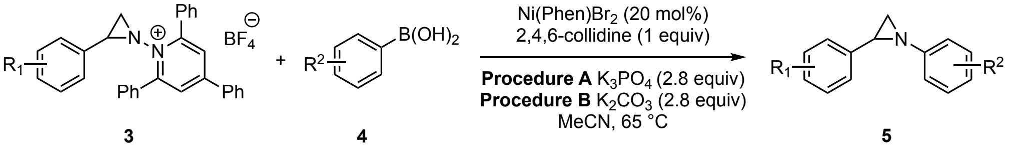

Procedure A A 20-mL scintillation vial was charged with Ni(Phen)Br_2 (20 mol%), potassium phosphate (2.8 equiv), aryl boronic acid (**4**, 2.4 equiv), pyridinium aziridine (**3**, 1 equiv) and a magnetic stir bar[4]. In an N_2 filled dry box, a solution of 2,4,6-collidine in acetonitrile (0.08 M, 1.0 equiv) was added to the scintillation vial with the rest of the reaction components. The reaction vial was heated at 65 °C for 36 h. After cooling to 23 °C, the reaction mixture was transferred to a centrifuge tube and centrifuged at 3220 g (6000 rpm) for 10 min. The supernatent was decanted. The residue was washed with CH_2Cl_2 and the combined supernatents were concentrated under reduced pressure and the crude mixture was purified as indicated below to afford the title compound.

Procedure B A 20-mL scintillation vial was charged with Ni(Phen)Br_2 (20 mol%), potassium carbonate (2.8 equiv), aryl boronic acid (**4**, 2.4 equiv), pyridinium aziridine (**3**, 1 equiv) and a magnetic stir bar. In an N_2 fill dry box, a solution of 2,4,6-collidine in acetonitrile (0.08 M, 1.0 equiv) was added to the reaction. The reaction was heated at 65 °C for 36 h. After colling to 23 °C, the reaction mixture was transferred to a centrifuge tube and centrifuged at 3220 g (6000) rpm for 10 minutes. The supernatent was decanted. The residue was washed with CH_2Cl_2 and the combined supernatents were concentrated under reduced pressure and the crude mixture was purified as indicated below to afford the title compound.

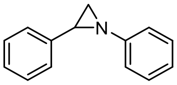

5a

*1,2-diphenylaziridine (**5a**)*. Prepared via Procedure A from **3a** on 0.16 mmol scale (based on **3a**) and obtained as a colorless oil (17.8 mg, 57% yield). Purified by alumina flash column chromatography (0.1% to 1% diethyl ether in hexane). ^{1}H NMR (400 MHz, $CDCl_3$) δ 7.30–7.20 (m, 4H), 7.20–7.09 (m, 3H), 6.96–6.92 (m, 2H), 6.87 (m, 1H), 2.99 (dd, *J* = 6.4, 3.3 Hz, 1H), 2.35 (dd, *J* = 6.4, 1.2 Hz, 1H), 2.29 (dd, *J* = 3.4, 1.2 Hz, 1H). ^{13}C NMR (101 MHz, $CDCl_3$) δ 154.7, 139.6, 129.2, 128.6, 127.5, 126.3, 122.7, 120.7, 41.7, 37.7. These spectral data are well-matched to those reported in the literature.[22]

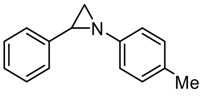

5b

*2-phenyl-1-(p-tolyl)aziridine (**5b**)*. Prepared via Procedure A from **3a** on 0.08 mmol scale (based on **3a**) and obtained as a yellow oil (12.6 mg, 67% yield). Purified by alumina flash column chromatography (0.1% to 1% diethyl ether in hexane). ^{1}H NMR (400 MHz, $CDCl_3$) δ 7.43–7.33 (m, 4H), 7.31–7.27 (m, 1H), 7.06 (d, *J* = 8.2 Hz, 2H), 6.95 (d, *J* = 8.3 Hz, 2H), 3.05

[4] All reaction components were weighed out under air and carried into an N_2 filled dry box.

(dd, *J* = 6.4, 3.3 Hz, 1H), 2.43 (dd, *J* = 6.4, 1.2 Hz, 1H), 2.38 (dd, *J* = 3.3, 1.2 Hz, 1H). These spectral data are well-matched to those reported in the literature.[22]

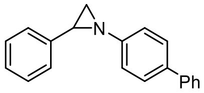

5c

*1-([1,1'-biphenyl]-4-yl)-2-phenylaziridine (**5c**)*.Prepared via Procedure A from **3a** on 0.16 mmol scale (based on **3a**) and obtained as a white solid (17.4 mg, 40% yield). Purified by C_{18} HPLC using 100% acetonitrile at 40 mL/min flow rate. ^{1}H NMR (400 MHz, $CDCl_3$) δ 7.60–7.56 (m, 2H), 7.54–7.49 (m, 2H), 7.47–7.37 (m, 6H), 7.36–7.30 (m, 2H), 7.18–7.11 (m, 2H), 3.17 (dd, *J* = 6.4, 3.3 Hz, 1H), 2.52 (dd, *J* = 6.4, 1.2 Hz, 1H), 2.46 (dd, *J* = 3.3, 1.2 Hz, 1H). ^{13}C NMR (101 MHz, $CDCl_3$) δ 154.1, 141.0, 139.5, 135.7, 128.9, 128.8, 128.6, 127.9, 127.5, 126.9, 126.4, 121.1, 41.9, 37.9. HRMS-APCI (m/z): [M+1]+ calcd. for C20H18N+, 272.1434; found, 272.1431.

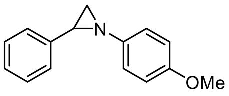

5d

*1-(4-methoxyphenyl)-2-phenylaziridine (**5d**)*. Prepared via Procedure B from **3a** on 0.08 mmol scale (based on **3a**) and obtained as a colorless oil (10.8 mg, 60% yield). Purified by alumina flash column chromatography (1% to 2% diethyl ether in hexane). ^{1}H NMR (400 MHz, $CDCl_3$) δ 7.42–7.32 (m, 4H), 7.31–7.27 (m, 1H), 6.98 (d, *J* = 8.9 Hz, 2H), 6.80 (d, *J* = 8.9 Hz, 2H), 3.77 (s, 3H), 3.03 (dd, *J* = 6.5, 3.4 Hz, 1H), 2.41 (dd, *J* = 6.4, 1.2 Hz, 1H), 2.37 (dd, *J* = 3.5, 1.2 Hz, 1H). These spectral data are well-matched to those reported in the literature.[22]

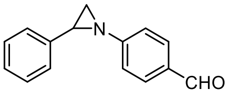

5e

*4-(2-phenylaziridin-1-yl)benzaldehyde (**5e**)*. Prepared via Procedure B from **3a** on 0.08 mmol scale (based on **3a**) and obtained as a white solid (17.9 mg, >99% yield). Purified by alumina flash column chromatography (1% to 5% ethyl acetate in hexane). ^{1}H NMR (400 MHz, CD_3CN) δ 9.86 (s, 1H), 7.86–7.72 (m, 2H), 7.44–7.33 (m, 4H), 7.32–7.29 (ddt, *J* = 6.4, 5.2, 2.6 Hz, 1H), 7.23–7.11 (m, 2H), 3.30 (dd, *J* = 6.4, 3.4 Hz, 1H), 2.58 (d, *J* = 6.4 Hz, 1H), 2.51 (dd, *J* = 3.4 Hz, 1H). ^{13}C NMR (101 MHz, CD_3CN) δ 192.0, 161.4, 139.9, 132.4, 131.9, 129.5, 128.5, 127.2, 121.8, 42.3, 38.2. HRMS-APCI (m/z): [M+1]+ calcd. for C15H14NO+, 224.1070; found, 224.1066.

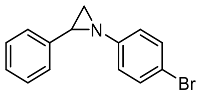

5f

*1-(4-bromophenyl)-2-phenylaziridine (**5f**)*. Prepared via Procedure B from **3a** on 0.08 mmol scale (based on **3a**) and obtained as a white solid (18.9 mg, 86% yield). Purified by alumina flash column chromatography (0.1% to 1% diethyl ether in hexane). ^{1}H NMR (400 MHz,

CDCl3) δ 7.40–7.32 (m, 6H), 7.32–7.27 (m, 1H), 6.96–6.79 (m, 2H), 3.08 (dd, J = 6.4, 3.4 Hz, 1H), 2.52–2.33 (m, 2H). ^{13}C NMR (101 MHz, $CDCl_3$) δ 153.8, 139.0, 132.0, 128.7, 127.6, 126.3, 122.5, 115.1, 41.9, 37.8. HRMS-ESI$^+$ (m/z): [M+1]+ calcd. for C14H13BrN+, 306.0448, 308.0468; found, 306.0485, 308.0464. These spectral data are well-matched to those reported in the literature.[22]

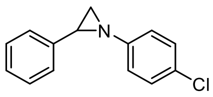

5g

*1-(4-chlorophenyl)-2-phenylaziridine (**5g**)*. Prepared via Procedure A from **3a** on 0.16 mmol scale (based on **3a**) and obtained as a colorless oil (16.4 mg, 45% yield). Purified by alumina flash column chromatography (0.1% to 1% diethyl ether in hexane). ^{1}H NMR (400 MHz, $CDCl_3$) δ 7.36 (d, J = 4.3 Hz, 4H), 7.36–7.27 (m, 1H), 7.23–7.17 (m, 2H), 7.01–6.94 (m, 2H), 3.08 (dd, J = 6.5, 3.4 Hz, 1H), 2.49–2.36 (m, 2H). ^{13}C NMR (101 MHz, $CDCl_3$) δ 153.1, 138.9, 129.0, 128.5, 127.5, 127.4, 126.2, 121.8, 41.8, 37.7. These spectral data are well-matched to those reported in the literature.[22]

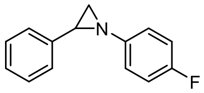

5h

*1-(4-fluorophenyl)-2-phenylaziridine (**5h**)*. Prepared via Procedure A from **3a** on 0.08 mmol scale (based on **3a**) and obtained as a colorless oil (11.7 mg, 69% yield). Purified by alumina flash column chromatography (0.1% to 1% diethyl ether in hexane). ^{1}H NMR (400 MHz, $CDCl_3$) δ 7.41–7.33 (m, 4H), 7.33–7.27 (m, 1H), 7.04–6.89 (m, 4H), 3.06 (dd, J = 6.5, 3.4 Hz, 1H), 2.43 (dd, J = 6.5, 1.1 Hz, 1H), 2.40 (dd, J = 3.3, 1.1 Hz, 1H). ^{13}C NMR (101 MHz, $CDCl_3$) δ 158.7 (d, $^1J_{C-F}$ = 240 Hz), 150.6 (d, $^4J_{C-F}$ = 2.6 Hz), 139.3, 128.6, 127.6, 126.3, 121.7 (d, $^3J_{C-F}$ = 8.0 Hz), 115.6 (d, $^2J_{C-F}$ = 23 Hz), 41.9, 37.8. ^{19}F NMR (376 MHz, $CDCl_3$) δ –121.37. These spectral data are well-matched to those reported in the literature.[22]

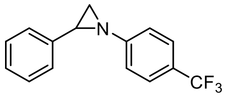

5i

*2-phenyl-1-(4-(trifluoromethyl)phenyl)aziridine (**5i**)*. Prepared via Procedure B from **3a** on 0.08 mmol scale (based on **3a**) and obtained as a colorless oil (13.6 mg, 65% yield). Purified by alumina flash column chromatography (0.1% to 1% diethyl ether in hexane). ^{1}H NMR (400 MHz, $CDCl_3$) δ 7.56–7.47 (m, 2H), 7.42–7.29 (m, 5H), 7.15–7.03 (m, 2H), 3.16 (dd, J = 6.4, 3.4 Hz, 1H), 2.50 (dd, J = 6.4, 1.1 Hz, 1H), 2.48 (dd, J = 3.4, 1.1 Hz, 1H). ^{13}C NMR (101 MHz, $CDCl_3$) δ 157.7, 138.7, 128.7, 127.8, 126.4 (q, $^3J_{C-F}$ = 3.8 Hz), 126.3, 124.7 (q, $^2J_{C-F}$ = 33 Hz),124.5 (q, $^1J_{C-F}$ = 270 Hz), 123.2, 41.8, 37.8. ^{19}F NMR (377 MHz, $CDCl_3$) δ –61.74. HRMS-APCI (m/z): [M+1]+ calcd. for C15H13F3N+, 264.0995; found, 264.0990. These spectral data are well-matched to those reported in the literature.[22]

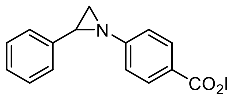

5j

*ethyl 4-(2-phenylaziridin-1-yl)benzoate (**5j**)*. Prepared via Procedure A from **3a** on 0.16 mmol scale (based on **3a**). Purified by alumina flash column chromatography and obtained as a colorless oil (22.2 mg, 52% yield). ^{1}H NMR (400 MHz, $CDCl_3$) δ 7.98–7.93 (m, 2H), 7.39–7.28 (m, 5H), 7.10–7.01 (m, 2H), 4.35 (q, J = 7.1 Hz, 2H), 3.17 (dd, J = 6.4, 3.3 Hz, 1H), 2.51 (dd, J = 6.4, 1.2 Hz, 1H), 2.48 (dd, J = 3.3, 1.2 Hz, 1H), 1.38 (t, J = 7.1 Hz, 3H). These spectral data are well-matched to those reported in the literature.[22]

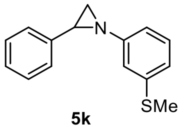

5k

*1-(3-(methylthio)phenyl)-2-phenylaziridine (**5k**)*. Prepared via Procedure A from **3a** on 0.16 mmol scale (based on **3a**) and obtained as a colorless oil (28.6 mg, 74% yield). Purified by C_{18} HPLC using 100% acetonitrile at 40 mL/min flow rate. ^{1}H NMR (400 MHz, CD_3CN) δ 7.41–7.34 (m, 4H), 7.33–7.26 (m, 1H), 7.17 (t, J = 7.9 Hz, 1H), 6.92 (t, J = 2.0 Hz, 1H), 6.87 (ddd, J = 7.8, 1.9, 1.0 Hz, 1H), 6.80 (ddd, J = 8.0, 2.1, 1.0 Hz, 1H), 3.16 (dd, J = 6.5, 3.4 Hz, 1H), 2.47 (dd, J = 6.5, 0.9 Hz, 1H), 2.45 (s, 3H), 2.38 (dd, J = 3.4, 1.0 Hz, 1H). ^{13}C NMR (101 MHz, CD_3CN) δ 156.4, 140.6, 140.4, 130.4, 129.4, 128.2, 127.2, 121.0, 118.7, 118.2, 42.1, 38.1, 15.4. HRMS-APCI (m/z): [M+1]+ calcd. for C15H16NS+, 242.0998; found, 242.0999.

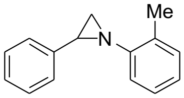

5l

Synthesis of aziridine **5l** was attempted by reacting pyridinium aziridine **3a** with o-tolylboronic acid (as well as (2,4-dimethylphenyl)boronic acid) via both Procedure A and Procedure B. None of the combinations gave the desired aziridine product **5l** detectable by the ^{1}H NMR spectrum of the crude mixture.

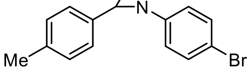

5m

*1-(4-bromophenyl)-2-(p-tolyl)aziridine (**5m**)*. Prepared via Procedure B from **3b** on 0.16 mmol scale (based on **3b**) and obtained as a white solid (30.9 mg, 67% yield). Purified by alumina flash column chromatography (0.1% to 1% diethyl ether in hexane). ^{1}H NMR (400 MHz, $CDCl_3$) δ 7.37–7.29 (m, 2H), 7.28–7.24 (m, 2H), 7.20–7.12 (m, 2H), 6.97–6.85 (m, 2H), 3.04 (dd, J = 6.4, 3.4 Hz, 1H), 2.43–2.36 (m, 2H), 2.35 (s, 3H). ^{13}C NMR (101 MHz, $CDCl_3$) δ 153.9, 137.4, 136.0, 132.0, 129.3, 126.2, 122.4, 115.0, 41.8, 37.7, 21.3. HRMS-APCI (m/z): [M+1]+ calcd. for C15H15BrN+, 320.0645, 322.0624; found, 320.0646, 322.0625.

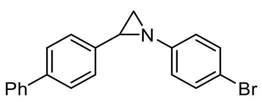

5n

*2-([1,1'-biphenyl]-4-yl)-1-(4-bromophenyl)aziridine (**5n**)*. Prepared via Procedure B from **3c** on 0.16 mmol scale (based on **3c**). The title compound was purified by C_{18} HPLC using 100% acetonitrile at 40 mL/min flow rate, which did not give a baseline separation and obtained as a mixture containing 23% of 2,4,6-triphenylpyridine. Spectroscopic data were extracted by comparing the spectra of the mixture with those of 2,4,6-triphenylpyridine, and the yield was determined to be 50% by integration of the ^{1}H NMR spectrum. ^{1}H NMR (400 MHz, $CDCl_3$) δ 7.64–7.58 (m, 4H), 7.46–7.43 (m, 4H), 7.39–7.34 (m, 3H), 6.98–6.92 (m, 2H), 3.12 (dd, *J* = 6.4, 3.4 Hz, 1H), 2.48–2.45 (m, 2H). ^{13}C NMR (101 MHz, $CDCl_3$) δ 153.7, 140.9, 140.6, 138.1, 132.1, 128.9, 127.5, 127.4, 127.2, 126.7, 122.5, 115.2, 41.7, 37.9. HRMS-APCI (m/z): [M+1]+ calcd. for C20H17BrN+, 350.0539, 352.0518; found, 350.0530, 352.0508.

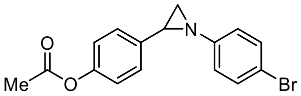

5o

*4-(1-(4-bromophenyl)aziridin-2-yl)phenyl acetate (**5o**)*. Prepared via Procedure B from **3f** on 0.16 mmol scale (based on **3f**) and obtained as a colorless oil (21.5 mg, 42% yield). Purified by alumina flash column chromatography (1% to 5% ethyl acetate in hexanes). ^{1}H NMR (400 MHz, $CDCl_3$) δ 7.42–7.31 (m, 4H), 7.14–7.05 (m, 2H), 6.98–6.83 (m, 2H), 3.06 (dd, *J* = 6.4, 3.3 Hz, 1H), 2.42 (dd, *J* = 6.4, 1.1 Hz, 1H), 2.38 (dd, *J* = 3.3, 1.1 Hz, 1H), 2.31 (s, 3H). ^{13}C NMR (101 MHz, $CDCl_3$) δ 169.7, 153.6, 150.2, 136.7, 132.1, 127.3, 122.4, 121.8, 115.2, 41.4, 37.9, 21.3. HRMS-APCI (m/z): [M+1]+ calcd. for C16H15BrNO2+, 332.0281, 334.0260; found, 332.0279, 334.0256.

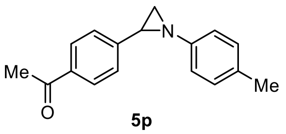

5p

*1-(4-(1-(p-tolyl)aziridin-2-yl)phenyl)ethan-1-one (**5p**)*. Prepared via Procedure A from **3g** on 0.16 mmol scale (based on **3g**) and obtained as a white solid (31.9 mg, 79% yield). Purified by alumina flash column chromatography (1% to 5% ethyl acetate in hexane). ^{1}H NMR (400 MHz, $CDCl_3$) δ 8.07–7.89 (m, 2H), 7.57–7.42 (m, 2H), 7.11–7.00 (m, 2H), 7.00–6.90 (m, 2H), 3.09 (dd, *J* = 6.4, 3.2 Hz, 1H), 2.61 (s, 3H), 2.49 (dd, *J* = 6.5, 1.3 Hz, 1H), 2.38 (dd, *J* = 3.3, 1.3 Hz, 1H), 2.30 (s, 3H). ^{13}C NMR (101 MHz, $CDCl_3$) δ 197.8, 151.7, 145.4, 136.3, 132.3, 129.7, 128.7, 126.4, 120.4, 41.4, 38.3, 26.7, 20.8. HRMS-APCI (m/z): [M+1]+ calcd. for C17H18NO+, 252.1383; found, 252.1382.

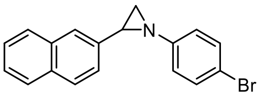

5q

*1-(4-bromophenyl)-2-(naphthalen-2-yl)aziridine (**5q**)*. Prepared via Procedure B from **3s** on 0.16 mmol scale (based on **3s**) and obtained as a colorless oil (32.0 mg, 62% yield). Purified

by C_{18} HPLC using 100% acetonitrile at 40 mL/min flow rate. ^{1}H NMR (400 MHz, $CDCl_3$) δ 7.91–7.74 (m, 4H), 7.52–7.42 (m, 3H), 7.39–7.34 (m, 2H), 6.99–6.94 (m, 2H), 3.24 (dd, *J* = 6.1, 3.7 Hz, 1H), 2.56–2.45 (m, 2H). ^{13}C NMR (101 MHz, $CDCl_3$) δ 153.8, 136.5, 133.5, 133.1, 132.1, 128.5, 127.9, 127.8, 126.5, 126.0, 125.3, 124.1, 122.5, 115.2, 42.2, 38.0. HRMS-APCI (m/z): [M+1]+ calcd. for C18H15BrN+, 324.0382, 326.0362; found, 324.0381, 326.0359.

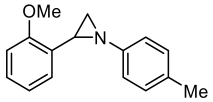

5r

*2-(2-methoxyphenyl)-1-(p-tolyl)aziridine (**5r**)*. Prepared via Procedure B from **3q** on 0.16 mmol scale (based on **3q**) and obtained as a yellow oil (16.3 mg, 43% yield). Purified by C_{18} HPLC using 100% acetonitrile at 40 mL/min flow rate. ^{1}H NMR (400 MHz, $CDCl_3$) δ 7.34 (dd, *J* = 7.5, 1.7 Hz, 1H), 7.26 (dd, *J* = 15.6, 1.8 Hz, 1H), 7.06 (d, *J* = 8.3 Hz, 2H), 7.03–6.99 (m, 2H), 6.96 (td, *J* = 7.4, 1.0 Hz, 1H), 6.90 (dd, *J* = 8.2, 1.1 Hz, 1H), 3.90 (s, 3H), 3.40 (dd, *J* = 6.5, 3.5 Hz, 1H), 2.40 (dd, *J* = 6.5, 1.3 Hz, 1H), 2.37 (dd, *J* = 3.5, 1.3 Hz, 1H), 2.29 (s, 3H). ^{13}C NMR (101 MHz, $CDCl_3$) δ 158.3, 152.7, 131.8, 129.6, 128.3, 127.9, 126.9, 120.8, 120.7, 110.2, 55.6, 37.1, 36.7, 20.9. HRMS-APCI (m/z): [M+1] calcd. for C16H18NO+, 240.1383; found, 240.1385.

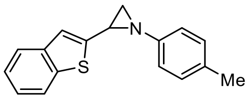

5s

*2-(benzo[b]thiophen-2-yl)-1-(p-tolyl)aziridine (**5s**)*. Prepared via Procedure A on 0.16 mmol scale (based on **3t**) and obtained as a yellow oil (15.3 mg, 36% yield). Purified by C_{18} HPLC using 100% acetonitrile at 40 mL/min flow rate. ^{1}H NMR (400 MHz, $CDCl_3$) δ 7.83–7.77 (m, 1H), 7.75–7.67 (m, 1H), 7.38–7.25 (m, 3H), 7.11–7.05 (m, 2H), 7.01–6.96 (m, 2H), 3.36 (dd, *J* = 6.2, 3.2 Hz, 1H), 2.56 (dd, *J* = 3.2, 1.1 Hz, 1H), 2.52 (dd, *J* = 6.3, 1.1 Hz, 1H), 2.30 (s, 3H). ^{13}C NMR (101 MHz, $CDCl_3$) δ 151.4, 145.3, 140.1, 139.3, 132.5, 129.8, 124.4, 124.1, 123.2, 122.5, 121.0, 120.5, 38.7, 38.5, 20.9. HRMS-APCI (m/z): [M+1]+ calcd. for C17H16NS+, 266.0998; found, 266.0998.

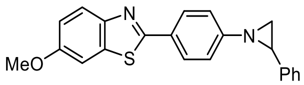

5t

*6-methoxy-2-(4-(2-phenylaziridin-1-yl)phenyl)benzo[d]thiazole (**5t**)*. Prepared via Procedure A from **3a** on 0.08 mmol scale (based on **3a**) and obtained as a pale-yellow oil (37.2 mg, 65% yield). Purified by alumina flash column chromatography (5% to 20% ethyl acetate in hexane). ^{1}H NMR (400 MHz, $CDCl_3$) δ 8.00–7.86 (m, 3H), 7.43–7.35 (m, 4H), 7.35–7.29 (m, 2H), 7.16– 7.10 (m, 2H), 7.07 (dd, *J* = 8.9, 2.6 Hz, 1H), 3.18 (dd, *J* = 6.4, 3.3 Hz, 1H), 2.53 (dd, *J* = 6.4, 1.2 Hz, 1H), 2.48 (dd, *J* = 3.3, 1.2 Hz, 1H). ^{13}C NMR (101 MHz, $CDCl_3$) δ 165.5, 157.7, 156.9, 148.9, 139.0, 136.3, 128.7, 128.4, 128.3, 127.7, 126.3, 123.5, 121.1, 115.5, 104.4, 55.9, 41.9, 37.9. HRMS-APCI (m/z): [M+1]+ calcd. for C22H19N2OS+, 359.1213; found, 359.1211.

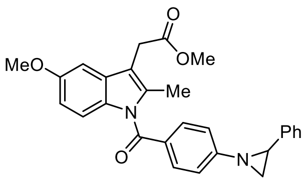

5u

*methyl 2-(5-methoxy-2-methyl-1-(4-(2-phenylaziridin-1-yl)benzoyl)-1H-indol-3-yl)acetate (**5u**)*. Prepared via Procedure A from **3a** and **4a** on 0.16 mmol scale (based on **3a**) and obtained as a white solid (52.5 mg, 72% yield). Purified by alumina flash column chromatography (3% to 10% ethyl acetate in hexane). ^{1}H NMR (400 MHz, $CDCl_3$) δ 7.70–7.60 (m, 2H), 7.42–7.29 (m, 5H), 7.14–7.08 (m, 2H), 6.96 (d, *J* = 2.6 Hz, 1H), 6.91 (d, *J* = 8.9 Hz, 1H), 6.66 (dd, *J* = 9.0, 2.6 Hz, 1H), 3.84 (s, 3H), 3.70 (s, 3H), 3.68 (s, 2H), 3.24 (dd, *J* = 6.4, 3.3 Hz, 1H), 2.57 (dd, *J* = 6.4, 1.1 Hz, 1H), 2.53 (dd, *J* = 3.4, 1.1 Hz, 1H), 2.40 (s, 3H). ^{13}C NMR (101 MHz, $CDCl_3$) δ 171.7, 169.0, 159.2, 155.9, 138.5, 136.3, 131.7, 131.2, 130.5, 129.3, 128.7, 127.8, 126.3, 120.7, 115.0, 111.8, 111.6, 101.1, 55.9, 52.2, 41.9, 37.9, 30.4, 13.2. HRMS-APCI (m/z): [M+1]+ calcd. for C28H27N2O4+, 455.1965; found, 455.1962.

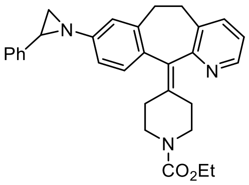

5v

*ethyl 4-(8-(2-phenylaziridin-1-yl)-5,6-dihydro-11H-benzo[5,6]cyclohepta[1,2-b]pyridin-11-ylidene)piperidine-1-carboxylate (**5v**)*. Prepared via Procedure A from **3a** and **4b** on 0.11 mmol scale (based on **3a**) and obtained as a yellow oil (32.1 mg, 63% yield). Purified by alumina flash column chromatography (3% to 10% ethyl acetate in hexane). ^{1}H NMR (400 MHz, CD_3CN) δ 7.93 (dd, *J* = 4.8, 1.7 Hz, 1H), 7.15–7.07 (m, 1H), 7.02–6.85 (m, 5H), 6.72 (ddd, *J* = 7.7, 4.8, 1.2 Hz, 1H), 6.59 (dd, *J* = 8.1, 2.2 Hz, 1H), 6.47 (d, *J* = 2.3 Hz, 1H), 6.42 (dd, *J* = 8.1, 2.4 Hz, 1H), 3.68 (qd, *J* = 7.1, 0.9 Hz, 2H), 3.36–3.18 (m, 2H), 3.03–2.90 (m, 2H), 2.69 (dd, *J* = 6.4, 3.3 Hz, 1H), 2.49–2.31 (m, 2H), 2.06–1.86 (m, 5H), 1.83–1.75 (m, 1H), 0.82 (td, *J* = 7.1, 1.0 Hz, 3H).[5] ^{13}C NMR (101 MHz, CD_3CN) δ 159.5, 159.4, 156.1, 155.0, 147.3, 140.7, 139.69, 139.67, 137.92, 137.90, 136.72, 136.70, 135.8, 134.8, 134.2, 134.1, 130.9, 129.4, 128.2, 127.1, 123.1, 122.3, 122.2, 118.9, 118.8, 61.8, 45.7, 45.6, 42.14, 42.08, 38.1, 38.0, 32.6, 31.9, 31.8, 31.4, 31.3, 15.0.[5] HRMS-APCI (m/z): [M+1]+ calcd. for C30H32N3O2+, 466.2489; found, 466.2485.

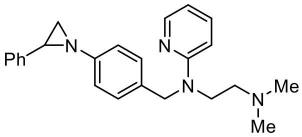

5w

*N[1],N[1]-dimethyl-N[2]-(4-(2-phenylaziridin-1-yl)benzyl)-N[2]-(pyridin-2-yl)ethane-1,2-diamine (**5w**)*. Prepared via Procedure A from **3a** and **4c** on 0.14 mmol scale (based on **3a**) and

[5] The complexity of the spectra data is due to the presence of interconverting rotamers of the carbamate in **5v**.

obtained as a colorless oil (12.6 mg, 24% yield). Purified by alumina column chromatography (1% to 10% ethyl acetate in hexane). ^{1}H NMR (400 MHz, Chloroform-*d*) δ 8.16 (ddd, *J* = 4.9, 2.0, 0.9 Hz, 1H), 7.41–7.33 (m, 5H), 7.31–7.27 (m, 1H), 7.15–7.10 (m, 2H), 7.00–6.95 (m, 2H), 6.53 (ddd, *J* = 7.1, 4.9, 0.8 Hz, 1H), 6.45 (d, *J* = 8.7 Hz, 1H), 4.71 (s, 2H), 3.66–3.59 (m, 2H), 3.07 (dd, *J* = 6.4, 3.3 Hz, 1H), 2.52–2.46 (m, 2H), 2.43 (dd, *J* = 6.4, 1.2 Hz, 1H), 2.38 (dd, *J* = 3.3, 1.2 Hz, 1H), 2.26 (s, 6H). ^{13}C NMR (101 MHz, $CDCl_3$) δ 158.3, 153.6, 148.2, 139.6, 137.3, 133.0, 128.6, 127.9, 127.4, 126.3, 120.8, 111.9, 105.9, 56.9, 51.5, 46.7, 46.0, 41.8, 37.8. HRMS-APCI (m/z): [M+1]+ calcd. for C24H29N4+, 373.2387; found, 373.2384.

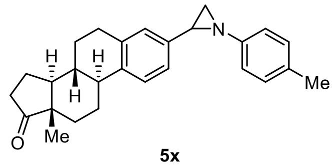

5x

*(8R,9S,13S,14S)-13-methyl-3-(1-(p-tolyl)aziridin-2-yl)-6,7,8,9,11,12,13,14,15,16-decahydro-17H-cyclopenta[a]phenanthren-17-one (**5x**)*. Prepared via Procedure A from **3v** on 0.16 mmol scale (based on **3w**) and obtained as a white solid (18.7 mg, 30% yield). Purified by alumina flash column chromatography (3% to 10% ethyl acetate in hexane). ^{1}H NMR (400 MHz, Chloroform-*d*) δ 7.30 (d, *J* = 8.5 Hz, 1H), 7.18 (d, *J* = 8.1 Hz, 1H), 7.13 (s, 1H), 7.08–7.02 (m, 2H), 6.97–6.90 (m, 2H), 3.00 (ddd, *J* = 6.5, 3.4, 1.5 Hz, 1H), 2.94 (dd, *J* = 9.1, 4.2 Hz, 2H), 2.57–2.46 (m, 1H), 2.49–2.40 (m, 1H), 2.40 (dt, *J* = 6.5, 1.2 Hz, 1H), 2.39–2.30 (m, 2H), 2.29 (s, 3H), 2.20–1.93 (m, 4H), 1.71–1.40 (m, 6H), 0.92 (s, 3H). ^{13}C NMR (101 MHz, $CDCl_3$) δ 221.0, 152.3, 139.0, 137.2, 136.9, 132.0, 129.7, 126.7, 125.6, 123.9, 120.5, 50.7, 48.1, 44.5, 41.5, 38.3, 37.7, 36.0, 31.7, 29.5, 26.6, 25.9, 21.7, 20.8, 14.0. HRMS-APCI (m/z): [M+1]+ calcd. for C27H32NO+, 386.2478; found, 386.2482.

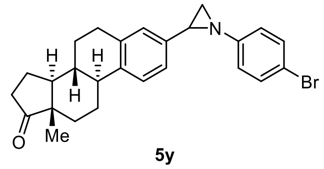

5y

*(8R,9S,13S,14S)-3-(1-(4-bromophenyl)aziridin-2-yl)-13-methyl-6,7,8,9,11,12,13,14,15,16-decahydro-17H-cyclopenta[a]phenanthren-17-one (**5y**)*. Prepared via Procedure B from **3v** on 0.16 mmol scale (based on **3w**) and obtained as a pale-yellow solid (23.4 mg, 32% yield). Purified by alumina flash column chromatography (3% to 10% ethyl acetate in hexane). ^{1}H NMR (400 MHz, $CDCl_3$) δ 7.37–7.32 (m, 2H), 7.30 (d, *J* = 8.1 Hz, 1H), 7.16 (d, *J* = 7.9 Hz, 1H), 7.10 (s, 1H), 6.95–6.89 (m, 2H), 3.06–2.99 (m, 1H), 2.93 (dd, *J* = 9.2, 4.3 Hz, 2H), 2.51 (dd, *J* = 18.8, 8.6 Hz, 1H), 2.46–2.38 (m, 3H), 2.37–2.27 (m, 1H), 2.19–1.94 (m, 4H), 1.68–1.40 (m, 6H), 0.92 (s, 3H). ^{13}C NMR (126 MHz, $cdcl_3$) δ 220.9, 153.9, 139.3, 137.0, 136.5, 132.0, 126.7, 125.7, 123.9, 122.4, 115.1, 50.7, 48.1, 47.6, 44.5, 41.7, 38.3, 37.8, 36.0, 31.7, 26.6, 25.9, 21.7, 14.0. HRMS-APCI (m/z): [M+1]+ calcd. for C26H29BrNO+, 482.1689, 484.1669; found, 482.1690, 484.1666.

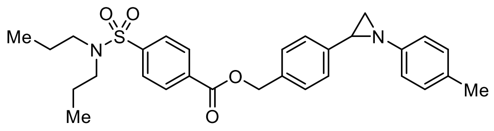

5z

*4-(1-(p-tolyl)aziridin-2-yl)benzyl 4-(N,N-dipropylsulfamoyl)benzoate (**5z**)*. Prepared via Procedure A from **3z** on 0.1 mmol scale (based on **3z**) and obtained as a yellow oil (29.5 mg, 58% yield). Purified by alumina flash column chromatography (5% to 20% ethyl acetate in hexane). ^{1}H NMR (400 MHz, $CDCl_3$) δ 8.25–8.15 (m, 2H), 7.93–7.83 (m, 2H), 7.52–7.37 (m, 4H), 7.10–7.02 (m, 2H), 6.97–6.90 (m, 2H), 5.39 (s, 2H), 3.19–2.97 (m, 5H), 2.44 (dd, *J* = 6.4, 1.3 Hz, 1H), 2.37 (dd, *J* = 3.3, 1.2 Hz, 1H), 2.29 (s, 3H), 1.60–1.47 (m, 4H), 0.86 (t, *J* = 7.4 Hz, 6H). ^{13}C NMR (101 MHz, $CDCl_3$) δ 165.3, 152.0, 144.5, 140.3, 134.6, 133.6, 132.2, 130.5, 129.7, 128.8, 127.2, 126.7, 120.5, 67.3, 50.0, 41.5, 37.9, 22.1, 20.8, 11.3. HRMS-APCI (m/z): [M+1]+ calcd. for C29H35N2O4S+, 507.2312; found, 507.2318.

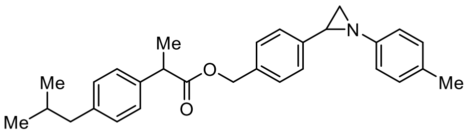

5aa

*4-(1-(p-tolyl)aziridin-2-yl)benzyl 2-(4-isobutylphenyl)propanoate (**5aa**)*. Prepared via Procedure A from **3x** on 0.12 mmol scale (based on **3x**) and obtained as a yellow oil (32.4 mg, 63% yield). Purified by alumina flash column chromatography (1% to 5% ethyl acetate in hexane). ^{1}H NMR (400 MHz, $CDCl_3$) δ 7.28 (d, *J* = 8.2 Hz, 2H), 7.20–7.15 (m, 4H), 7.09–6.99 (m, 4H), 6.93–6.86 (m, 2H), 5.23–4.87 (m, 2H), 3.71 (q, *J* = 7.2 Hz, 1H), 2.98 (dd, *J* = 6.4, 3.3 Hz, 1H), 2.41 (d, *J* = 7.2 Hz, 2H), 2.37 (dd, *J* = 6.5, 1.2 Hz, 1H), 2.30 (dd, *J* = 3.3, 1.2 Hz, 1H), 2.25 (s, 2H), 1.81 (dp, *J* = 13.6, 6.8 Hz, 1H), 1.47 (d, *J* = 7.2 Hz, 3H), 0.86 (d, *J* = 6.6 Hz, 6H). ^{13}C NMR (101 MHz, $CDCl_3$) δ 174.8, 152.1, 140.7, 139.6, 137.7, 135.2, 132.1, 129.7, 129.5, 128.1, 127.4, 126.4, 120.5, 66.3, 45.3, 45.2, 41.5, 37.8, 30.3, 22.5, 20.8, 18.6, 14.6. HRMS-APCI (m/z): [M+1]+ calcd. for C29H34NO2+, 428.2584; found, 428.2584.

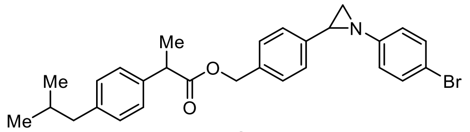

5ab

*4-(1-(4-bromophenyl)aziridin-2-yl)benzyl 2-(4-isobutylphenyl)propanoate (**5ab**)*. Prepared via Procedure B from **3x** on 0.12 mmol scale (based on **3x**) and obtained as a colorless oil (38.7 mg, 65% yield). Purified by alumina column chromatography (1% to 3% ethyl acetate in hexane). ^{1}H NMR (400 MHz, $CDCl_3$) δ 7.37–7.33 (m, 2H), 7.32–7.29 (m, 2H), 7.25–7.19 (m, 4H), 7.13–7.06 (m, 2H), 6.94–6.88 (m, 2H), 5.17–5.04 (m, 2H), 3.76 (q, *J* = 7.2 Hz, 1H), 3.06 (dd, *J* = 6.5, 3.3 Hz, 1H), 2.46 (d, *J* = 7.2 Hz, 2H), 2.43 (dd, *J* = 6.4, 1.2 Hz, 1H), 2.39 (dd, *J* = 3.4, 1.1 Hz, 1H), 1.86 (dp, *J* = 13.6, 6.8 Hz, 1H), 1.52 (d, *J* = 7.2 Hz, 3H), 0.91 (d, *J* = 6.6 Hz, 6H). ^{13}C NMR (101 MHz, $CDCl_3$) δ 174.6, 153.6, 140.7, 138.9, 137.7, 135.5, 132.0, 129.4, 128.2, 127.4, 126.4, 122.4, 115.2, 66.2, 45.3, 45.2, 41.6, 37.8, 30.3, 22.5, 18.5. HRMS-APCI (m/z): [M+1]+ calcd. for C28H31BrNO2+, 492.1553, 494.1512; found, 492.1533, 494.1509.

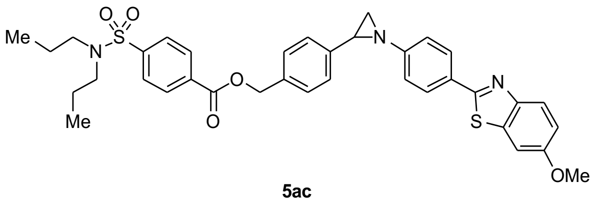

5ac

*4-(1-(4-(6-methoxybenzo[d]thiazol-2-yl)phenyl)aziridin-2-yl)benzyl 4-(N,N-dipropylsulfamoyl)benzoate (**5ac**)*. Prepared via Procedure A from **3z** on 0.12 mmol scale (based on **3z**) and obtained as a yellow solid (32.3 mg, 41% yield). Purified by alumina column chromatography (5% to 20% ethyl acetate in hexane). ^{1}H NMR (400 MHz, $CDCl_3$) δ 8.22–8.15 (m, 2H), 7.97–7.83 (m, 5H), 7.51–7.39 (m, 4H), 7.34 (d, *J* = 2.5 Hz, 1H), 7.14–7.10 (m, 2H), 7.07 (dd, *J* = 8.9, 2.6 Hz, 1H), 5.40 (s, 2H), 3.89 (s, 3H), 3.19 (dd, *J* = 6.4, 3.3 Hz, 1H), 3.14–3.05 (m, 4H), 2.55 (dd, *J* = 6.3, 1.2 Hz, 1H), 2.47 (dd, *J* = 3.3, 1.2 Hz, 1H), 1.61–1.47 (m, 4H), 0.86 (t, *J* = 7.4 Hz, 6H). ^{13}C NMR (101 MHz, $CDCl_3$) δ 165.5, 165.2, 157.7, 156.7, 148.9, 144.6, 139.6, 136.3, 135.0, 133.5, 130.5, 128.9, 128.5, 128.4, 127.2, 126.7, 123.5, 121.1, 115.6, 104.4, 67.3, 56.0, 50.0, 41.6, 38.0, 22.1, 11.3. HRMS-APCI (m/z): [M+1]+ calcd. for C36H38N3O5S2+, 656.2247; found, 656.2253.

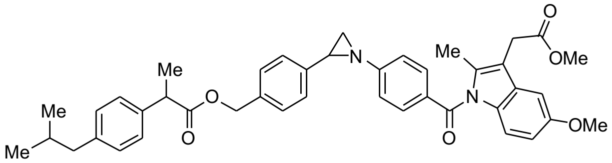

5ad

*4-(1-(4-(5-methoxy-3-(2-methoxy-2-oxoethyl)-2-methyl-1H-indole-1-carbonyl)phenyl)aziridin-2-yl)benzyl 2-(4-isobutylphenyl)propanoate (**5ad**)*. Prepared via Procedure A from **3x** and **4a** on 0.12 mmol scale (based on **3x**) and obtained as a yellow solid (53.9, 67% yield). Purified by alumina column chromatography (5% to 20% ethyl acetate in hexane). ^{1}H NMR (400 MHz,$CDCl_3$) δ 7.70–7.61 (m, 2H), 7.33 (d, *J* = 8.2 Hz, 2H), 7.27–7.19 (m, 4H), 7.11–7.07 (m, 4H), 6.97 (d, *J* = 2.5 Hz, 1H), 6.91 (d, *J* = 9.0 Hz, 1H), 6.66 (dd, *J* = 9.0, 2.5 Hz, 1H), 5.18–5.06 (m, 2H), 3.84 (s, 3H), 3.76 (q, *J* = 7.2 Hz, 1H), 3.71 (s, 3H), 3.68 (s, 2H), 3.21 (dd, *J* = 6.4, 3.3 Hz, 1H), 2.56 (d, *J* = 6.4 Hz, 1H), 2.49 (d, *J* = 3.4 Hz, 1H), 2.46 (d, *J* = 7.2 Hz, 2H), 2.40 (s, 3H), 1.86 (dp, *J* = 13.5, 6.7 Hz, 1H), 1.52 (d, *J* = 7.2 Hz, 3H), 0.90 (d, *J* = 6.6 Hz, 6H). ^{13}C NMR (101 MHz, $CDCl_3$) δ 174.7, 171.6, 169.0, 159.0, 155.9, 140.7, 138.4, 137.7, 136.3, 135.8, 131.8, 131.2, 130.5, 129.5, 129.4, 128.2, 127.4, 126.4, 120.7, 115.0, 111.8, 111.6, 101.1, 66.1, 55.9, 52.2, 45.3, 45.2, 41.6, 37.9, 30.3, 22.5, 18.5, 13.2. HRMS-APCI (m/z): [M+1]+ calcd. for C42H45N2O6+, 673.3272; found, 673.3274.

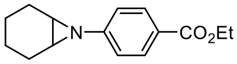

5ae

*ethyl 4-(7-azabicyclo[4.1.0]heptan-7-yl)benzoate (**5ae**)*. Prepared via Procedure A from **3ab** on 0.13 mmol scale (based on **3ab**) and obtained as a colorless oil (11.5 mg, 36% yield). Purified by alumina column chromatography (3% to 5% ethyl acetate in hexane). ^{1}H NMR (400 MHz, $CDCl_3$) δ 7.90 (d, *J* = 8.7 Hz, 2H), 6.97 (d, *J* = 8.6 Hz, 2H), 4.33 (q, *J* = 7.1 Hz, 2H), 2.41–2.32 (m, 2H), 2.05 (ddd, *J* = 13.3, 7.5, 5.5 Hz, 2H), 1.99–1.84 (m, 2H), 1.55–1.44 (m, 2H),

1.37 (t, *J* = 7.1 Hz, 3H), 1.34–1.25 (m, 2H). ^{13}C NMR (101 MHz, $CDCl_3$) δ 166.7, 160.3, 130.9, 124.0, 120.2, 60.7, 39.1, 24.6, 20.4, 14.5. HRMS-APCI (m/z): [M+1]+ calcd. for C15H20NO2+, 246.1489; found, 246.1481.

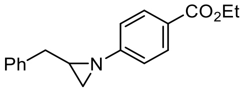

5af

*ethyl 4-(2-benzylaziridin-1-yl)benzoate (**5af**).* Prepared via Procedure A from **3ac** on 0.09 mmol scale (based on **3ac**) and obtained as a colorless oil (7.6 mg, 30% yield). Purified by C_{18} HPLC using 100% acetonitrile at 40 mL/min flow rate. ^{1}H NMR (400 MHz, $CDCl_3$) δ 7.84 (d, *J* = 8.6 Hz, 2H), 7.40–7.27 (m, 5H), 6.77 (d, *J* = 8.6 Hz, 2H), 4.31 (q, *J* = 7.1 Hz, 2H), 2.96 (dd, *J* = 14.1, 5.0 Hz, 1H), 2.82 (dd, *J* = 14.1, 7.4 Hz, 1H), 2.37 (dddd, *J* = 7.3, 6.0, 5.0, 3.4 Hz, 1H), 2.31 (d, *J* = 3.5 Hz, 1H), 2.17 (d, *J* = 6.0 Hz, 1H), 1.35 (t, *J* = 7.1 Hz, 3H). ^{13}C NMR (101 MHz, $CDCl_3$) δ 166.6, 159.1, 139.1, 130.9, 129.1, 128.8, 126.8, 124.4, 120.3, 60.7, 41.7, 39.6, 34.2, 14.5. HRMS-APCI (m/z): [M+1]+ calcd. for C18H20NO2+, 282.1489; found, 282.1477.

B.7 Nucleophilic Opening of Pyridinium Aziridines

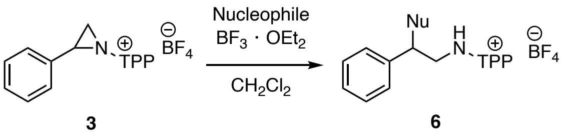

Procedure A The following procedure was carried out in an N_2-filled dry box. A 20-mL scintillation vial was charged with 2,4,6-triphenyl-1-(2-phenylaziridin-1-yl)pyridinium tetrafluoroborate (**3**, 300 mg, 0.586 mmol, 1.00 equiv), BF_3·OEt_2 (0.075 mL, 0.59 mmol, 1.0 equiv), and CH_2Cl_2 (5 mL). A 25-mL Schlenk tube is charged with the appropriate nucleophile (0.703 mmol, 1.20 equiv). The CH_2Cl_2 solution of **3** and BF_3·OEt_2 was added to the Schlenk flask that contained the nucleophile. The resulting reaction mixture was allowed to stir at 23 °C for 16 h. Solvent was removed under reduced pressure and the residue was purified by SiO_2 gel chromatography (eluent 2:1 ethyl acetate:hexane) to afford the title compound.

Procedure B The following procedure was carried out in an N_2-filled dry box. A 20-mL scintillation vial was charged with 2,4,6-triphenyl-1-(2-phenylaziridin-1-yl)pyridinium tetrafluoroborate (**3**, 300 mg, 0.586 mmol, 1.00 equiv), BF_3·OEt_2 (0.075 mL, 0.586 mmol, 1.00 equiv), and CH_2Cl_2 (5 mL). A 25-mL Schlenk tube is charged with the appropriate nucleophile (0.703 mmol, 1.20 equiv). The CH_2Cl_2 solution of **3** and BF_3·OEt_2 was added to the Schlenk flask that contained the nucleophile. The resulting reaction mixture was allowed to stir at 41 °C for 48 h. Solvent was removed under reduced pressure and the residue was purified by SiO_2 gel chromatography (eluent 2:1 ethyl acetate:hexane) to afford the title compound.

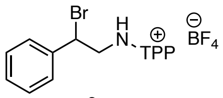

6a

*1-((2-bromo-2-phenylethyl)amino)-2,4,6-triphenylpyridin-1-ium tetrafluoroborate (**6a**).* Prepared via Procedure B from tetrabutylammonium bromide as nucleophile and obtained in 98% NMR yield. ^{1}H NMR (400 MHz, $CDCl_3$): δ 7.88 (s, 2H), 7.85–7.83 (m, 4H), 7.77–7.75 (m, 2H), 7.60–7.51 (m, 9H), 7.14 (dt, *J* = 12.8, 6.6 Hz, 3H), 6.87 (d, *J* = 7.0 Hz, 2H), 6.42 (t, *J* = 7.2 Hz, 1H)4.34 (dd, *J* = 8.0, 4.8 Hz, 1H), 3.37–3.25 (m, 2H). ^{19}F NMR (376 MHz, $CDCl_3$): δ –78.3. HRMS-APCI (m/z): [M]+ calcd. for C31H26BrN2+, 505.1274, 507.1253; found, 505.1256, 507.1235. Attempts to isolate **6a** by column chromatography led to recovery of 2,4,6-triphenyl-1-(2-phenylaziridin-1-yl)pyridinium tetrafluoroborate **3a**.

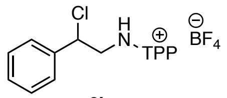

6b

*1-((2-chloro-2-phenylethyl)amino)-2,4,6-triphenylpyridin-1-ium tetrafluoroborate (**6b**).* Prepared via Procedure B from tetrabutylammonium chloride as nucleophile and obtained in 98% NMR yield. ^{1}H NMR (400 MHz, $CDCl_3$): δ 7.94 (s, 2H), 7.89–7.86 (m, 4H), 7.81–7.79 (m, 2H), 7.56 (dd, *J* = 22.6, 6.9 Hz, 9H), 7.21–7.14 (m, 3H), 6.90–6.88 (m, 2H), 6.56 (t, *J* = 6.0 Hz, 1H), 4.37–4.33 (m, 1H), 3.24–3.20 (m, 2H). ^{19}F NMR 376 MHz, $CDCl_3$): δ –78.3. HRMS-APCI (m/z): [M]+ calcd. for C31H26ClN2+, 461.1779; found, 461.1762. Attempts to isolate **6b** by column chromatography led to recovery of 2,4,6-triphenyl-1-(2-phenylaziridin-1-yl)pyridinium tetrafluoroborate **3a**.

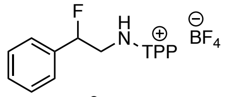

6c

*1-((2-fluoro-2-phenylethyl)amino)-2,4,6-triphenylpyridin-1-ium tetrafluoroborate (**6c**).* A 25-ml Schlenk tube was charged with 2,4,6-triphenyl-1-(2-phenylaziridin-1-yl)pyridinium tetrafluoroborate (**3**, 300 mg, 0.586 mmol, 1.00 equiv) and CH_2Cl_2 (5 mL) under N_2. HF-pyridine was added to the CH_2CL_2 solution of **3a** dropwise at 0 °C and the reaction mixture was allowed to stir at 23 °C for 16 h. Solvent was removed under reduced pressure and the residue was purified by SiO_2 gel chromatography to obtain the product as white solid (281 mg, 90% yield). ^{1}H NMR (400 MHz, $CDCl_3$): δ 7.87–7.90 (m, 4H), 7.89 (s, 2H), 7.76 (dq, *J* = 6.3, 1.9 Hz, 2H), 7.62–7.48 (m, 9H), 7.24–7.16 (m, 3H), 6.85–6.83 (m, 2H), 6.55 (t, *J* = 6.2 Hz, 1H), 4.92 (ddd, *J* = 48.1, 6.2, 4.5 Hz, 1H), 3.11–3.08 (m, 1H), 3.06–3.02 (m, 1H). ^{13}C NMR (100 MHz, $CDCl_3$): δ 156.0, 155.9, 135.7 (d, J_{C-F} = 20 Hz), 134.4, 132.1, 131.6, 130.9, 129.8, 129.6, 129.5, 128.9, 128.7, 128.3, 127.0, 125.1 (d, J_{C-F} = 7.2 Hz), 91.7 (d, J_{C-F} = 170 Hz), 55.7 (d, J_{C-F} = 23 Hz). ^{19}F NMR (376 MHz, $CDCl_3$): δ –152.8 (d, *J* = 20.6 Hz), –184.1 (dt, *J* = 47.2, 24.2 Hz). HRMS-ESI (m/z): [M]+ calcd. for C31H26FN2+, 445.2075; found, 445.2072.

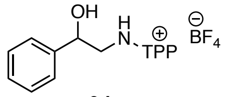

6d

*1-((2-hydroxy-2-phenylethyl)amino)-2,4,6-triphenylpyridinium tetrafluoroborate (**6d**).* Prepared via Procedure A from water as nucleophile and obtained as an off-white solid (171 mg, 55% yield). ^{1}H NMR (400 MHz, $CDCl_3$): δ 7.88 (s, 2H), 7.87–7.84 (m, 4H), 7.76 (dd, *J* = 8.1, 1.5 Hz, 2H), 7.62–7.49 (m, 9H), 7.13–7.11 (m, 3H), 6.84–6.81 (m, 2H), 6.68 (t, *J* = 6.7 Hz, 1H), 4.27 (dd, *J* = 7.4, 3.4 Hz, 1H), 2.92–2.77 (m, 2H), 2.27 (s, 1H). ^{13}C NMR (100 MHz, $CDCl_3$): δ 155.5, 155.4, 140.1, 134.3, 132.1, 131.6, 130.8, 129.8, 129.6, 129.5, 128.5, 128.2, 127.8, 126.8,

125.5, 70.4, 58.2. ^{19}F NMR (376 MHz, $CDCl_3$): δ –152.46, –152.51. HRMS-ESI (m/z): [M]+ calcd. for C31H27N2O+, 443.2118; found, 443.2115.

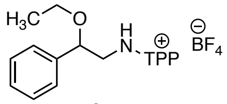

6e

*1-((2-ethoxy-2-phenylethyl)amino)-2,4,6-triphenylpyridinium tetrafluoroborate (**6e**).* Prepared via Procedure A from ethanol as nucleophile and obtained as a yellow solid (320 mg, 98% yield). ^{1}H NMR (400 MHz, $CDCl_3$): δ 8.78 (d, *J* = 6.2 Hz, 2H), 8.46 (bs, 1H), 8.26 (t, *J* = 7.7 Hz, 1H), 7.84 (t, *J* = 7.0 Hz, 2H), 7.43 (d, *J* = 8.3 Hz, 2H), 7.18 (d, *J* = 8.3 Hz, 2H), 4.58 (q, *J* = 6.3 Hz, 1H), 1.63 (d, *J* = 6.6 Hz, 3H). ^{13}C NMR (101 MHz, $CDCl_3$): δ 155.3, 155.2, 138.3, 134.4, 132.0, 131.5, 130.9, 129.8, 129.6, 129.4, 128.6, 128.3, 128.1, 127.0, 126.3, 78.4, 64.1, 57.0, 15.2. ^{19}F NMR (376 MHz, $CDCl_3$): δ –153.21, –153.26. HRMS-APCI (m/z): [M]+ calcd. for C33H31N2O+, 471.2431; found, 471.2424.

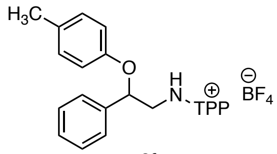

6f

*2,4,6-triphenyl-1-((2-phenyl-2-(p-tolyloxy)ethyl)amino) pyridinium tetrafluoroborate (**6f**).* Prepared via Procedure A from *p*-cresol as nucleophile and obtained as a light-yellow solid (75 mg, 31% yield). ^{1}H NMR (400 MHz, $CDCl_3$): δ 7.92 (s, 2H), 7.85 (dd, *J* = 7.8, 1.6 Hz, 4H), 7.81 (dd, *J* = 7.8, 1.7 Hz, 2H), 7.59–7.53 (m, 9H), 7.17 (t, *J* = 3.3 Hz, 3H), 6.91–6.83 (m, 5H), 6.22 (d, *J* = 8.6 Hz, 2H), 4.71–4.68 (m, 1H), 3.14–3.03 (m, 2H), 2.22 (s, 3H).^{13}C NMR (101 MHz, $CDCl_3$): δ 155.6, 155.4, 154.5, 137.3, 134.6, 132.0, 131.5, 130.9, 130.6, 129.8, 129.7, 129.6, 129.4, 128.8, 128.3, 128.2, 127.1, 125.8, 115.3, 56.8, 20.5. ^{19}F NMR (376 MHz, $CDCl_3$): δ –152.85, –152.90. HRMS-APCI (m/z): [M]+ calcd. for C38H33N2O+, 533.2587; found, 533.2583.

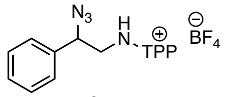

6g

*1-((2-azido-2-phenylethyl)amino)-2,4,6-triphenylpyridinium tetrafluoroborate (**6g**).* Prepared via Procedure A from tetrabutylammonium azide as nucleophile and obtained as an off-white solid (254 mg, 78% yield). ^{1}H NMR (400 MHz, $CDCl_3$): δ 7.89–7.87 (m, 6H), 7.77–7.75 (m, 2H), 7.64–7.48 (m, 9H), 7.24–7.19 (m, 3H), 6.87 (dd, *J* = 7.7, 1.6 Hz, 2H), 6.42 (dd, *J* = 7.6, 6.0 Hz, 1H), 4.03 (dd, *J* = 8.0, 4.5 Hz, 1H), 2.99–2.85 (m, 2H). ^{13}C NMR (100 MHz, $CDCl_3$):

δ 155.9, 155.5, 135.4, 134.4, 132.0, 131.5, 130.8, 129.7, 129.6, 129.5, 129.0, 128.9, 128.3, 127.1, 126.7, 63.3, 55.4. ^{19}F NMR (377 MHz, $CDCl_3$): δ –152.66, –152.71. HRMS-APCI (m/z): [M]+ calcd. for C31H26N5+, 468.2183; found, 468.2192.

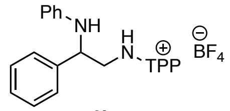

6h

*2,4,6-triphenyl-1-((2-phenyl-2-(phenylamino)ethyl)amino) pyridinium tetrafluoroborate (**6h**)*. Prepared via Procedure B from aniline as nucleophile and obtained as a yellow solid (251 mg, 71% yield). ^{1}H NMR (400 MHz, $CDCl_3$): δ 7.86 (s, 2H), 7.82–7.80 (m, 4H), 7.74 (dd, *J* = 8.2, 1.4 Hz, 2H), 7.61–7.47 (m, 9H), 7.15–7.08 (m, 3H), 6.95 (td, *J* = 8.0, 1.5 Hz, 2H), 6.85–6.82 (m, 2H), 6.58 (t, *J* = 7.3 Hz, 1H), 6.24 (t, *J* = 7.4 Hz, 1H), 6.06 (d, *J* = 7.7 Hz, 2H), 4.01–3.99 (m, 1H), 3.54 (s, 1H), 3.05 (dd, *J* = 7.2, 5.2 Hz, 2H). ^{13}C NMR (100 MHz, $CDCl_3$): δ 155.9, 155.6, 146.2, 138.5, 134.3, 132.1, 131.6, 130.7, 129.8, 129.5, 129.4, 129.1, 129.0, 128.3, 127.7, 127.0, 126.0, 117.7, 113.4, 56.5, 55.1. ^{19}F NMR (376 MHz, $CDCl_3$): δ –152.61, –152.66. HRMS-APCI (m/z): [M]+ calcd. for C37H32N3+, 518.2591; found, 518.2584.

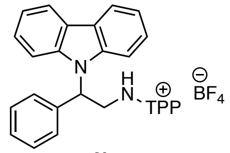

6i

*1-((2-(9H-carbazol-9-yl)-2-phenylethyl)amino)-2,4,6-triphenylp pyridinium tetrafluoroborate (**6i**).* Prepared via Procedure B from carbazole as nucleophile and obtained as light-yellow solid (378 mg, 95% yield). ^{1}H NMR (400 MHz, $CDCl_3$): δ 8.03–8.01 (m, 2H), 7.84 (s, 2H), 7.75–7.73 (m, 2H), 7.64–7.62 (m, 4H), 7.53–7.46 (m, 3H), 7.38–7.34 (m, 2H), 7.29 (d, *J* = 7.7 Hz, 4H), 7.24–7.18 (m, 4H), 7.11 (dq, *J* = 8.3, 2.4 Hz, 3H), 6.79–6.73 (m, 4H), 5.70 (t, *J* = 7.7 Hz, 1H), 5.36 (t, *J* = 7.1 Hz, 1H), 3.97 (t, *J* = 7.5 Hz, 2H). ^{13}C NMR (100 MHz, $CDCl_3$): δ 156.0, 154.7, 139.3, 135.5, 134.4, 131.8, 131.3, 130.0, 129.6, 129.0, 128.8, 128.7, 128.2, 127.8, 127.3, 126.1, 125.9, 123.3, 120.1, 119.5, 110.0, 55.0, 51.9. ^{19}F NMR (376 MHz, $CDCl_3$): δ –152.36, –152.44. HRMS-APCI (m/z): [M]+ calcd. for C43H34N3+, 592.2747; found, 592.2746.

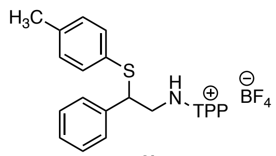

6j

*2,4,6-triphenyl-1-((2-phenyl-2-(p-tolylthio)ethyl)amino)pyridinium tetrafluoroborate (**6j**)*. Prepared via Procedure B from *p*-thiocresol as nucleophile and obtained as light-yellow solid (268 mg, 72% yield). ^{1}H NMR (400 MHz, $CDCl_3$): δ 7.86 (s, 2H), 7.78–7.73 (m, 6H), 7.59–7.48 (m, 9H), 7.14–7.13 (m, 1H), 7.10–7.06 (m, 2H), 6.99 (d, *J* = 7.9 Hz, 2H), 6.88–6.86 (m, 2H), 6.69–6.67 (m, 2H), 6.13–6.09 (m, 1H), 3.46 (dd, *J* = 8.2, 6.0 Hz, 1H), 3.19–3.12 (m, 1H), 3.08–3.01 (m, 1H), 2.33 (s, 3H). ^{13}C NMR (100 MHz, $CDCl_3$): δ 156.0, 155.4, 138.1, 137.2, 134.4, 133.1, 132.1, 131.4, 130.7, 130.0, 129.8, 129.5, 129.4, 129.0, 128.3, 128.0, 127.2, 127.1, 55.5, 51.0, 21.3. ^{19}F NMR (376 MHz, $CDCl_3$): δ –152.72, –152.77. HRMS-APCI (m/z): [M]+ calcd. for C38H33N2S+, 549.2359; found, 549.2350.

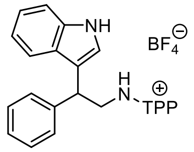

6k

*1-((2-(1H-indol-3-yl)-2-phenylethyl)amino)-2,4,6-triphenylpyridin-1-ium tetrafluoroborate (**6k**).* Prepared via Procedure B from indole as nucleophile and obtained as an light brown solid (159 mg, 37% yield). ^{1}H NMR (400 MHz, $CDCl_3$): δ 8.59 (s, 1H), 7.78 (s, 2H), 7.68–7.66 (m, 6H), 7.56–7.40 (m, 9H), 7.25 (t, *J* = 4.1 Hz, 1H), 7.05–6.94 (m, 4H), 6.82–6.75 (m, 2H), 6.66–6.64 (m, 2H), 6.34 (d, *J* = 2.4 Hz, 1H), 6.14 (dd, *J* = 13.5, 5.9 Hz, 1H), 3.72 (t, *J* = 7.0 Hz, 1H), 3.23 (ddt, *J* = 46.6, 10.3, 7.4 Hz, 2H). ^{13}C NMR (100 MHz, $CDCl_3$): δ 155.2, 154.9, 140.3, 136.5, 134.0, 131.8, 131.5, 130.2, 129.6, 129.2, 128.9, 128.5, 128.0, 127.1, 126.66, 126.51, 125.8, 122.1, 121.6, 118.8, 118.2, 113.6, 111.8, 55.7, 40.5 ^{19}F NMR (377 MHz, $CDCl_3$): δ -152.09, -152.15. HRMS-APCI (m/z): [M]+ calcd. for C39H32N3+, 542.2591; found, 542.2579.

B.8 Cross-Coupling of *N*-Pyridinium Amines

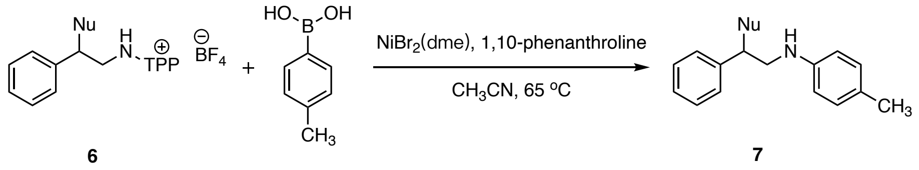

The following procedure was carried out in an N_2-filled dry box. A 20-mL scintillation vial was charged with compound **6** (0.100 mmol, 1.00 equiv) and CH_3CN (1 mL). A separate 20-mL was charged with $NiBr_2$(dme) (3.1 mg, 0.010 mmol, 10 mol%), 1,10-phenanthroline (2.4 mg, 0.014 mmol, 0.14 equiv), K_3PO_4 (53.1 mg, 0.250 mmol, 2.50 equiv), *p*-toluyl boronic acid (20.4 mg, 0.150 mmol, 1.50 equiv). The CH_3CN solution of **6** was added to the vial containing $NiBr_2$·DME. The reaction mixture was stirred at 65 °C for 18 h. The reaction mixture was cooled to 23 °C. The reaction mixture was centrifuged at 3220 g (6000 rpm) for 10 min and the supernatant was decanted. Solvent was removed under reduced pressure and the residue was purified by alumina gel column chromatography to obtain the title compound.

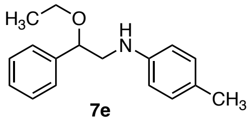

7e

*N-(2-ethoxy-2-phenylethyl)-4-methylaniline (**7e**).* Prepared from 1-((2-ethoxy-2-phenylethyl)amino)-2,4,6-triphenylpyridinium tetrafluoroborate (**6e**) and obtained as a pale-yellow liquid (8.9 mg, 39% yield). The purification was done by alumina gel column chromatography using 95:5 hexanes:ethyl acetate as eluent. ^{1}H NMR (400 MHz, $CDCl_3$): δ 7.40–7.29 (m, 5H), 7.00 (d, *J* = 8.0 Hz, 2H), 6.62 (d, *J* = 8.4 Hz, 2H), 4.50 (dd, *J* = 8.4, 4.5 Hz, 1H), 3.49–3.25 (m, 4H), 2.25 (s, 3H), 1.20 (t, *J* = 7.0 Hz, 3H). ^{13}C NMR (126 MHz, $CDCl_3$): δ 145.6, 140.8, 129.9, 128.7, 128.1, 127.4, 126.8, 114.1, 80.3, 64.6, 51.6, 20.6, 15.5. HRMS-APCI (m/z): [M+1]+ calcd. for C17H22NO+, 256.1696; found, 256.1693.

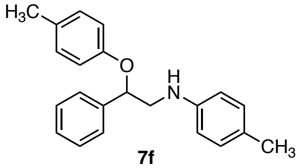

7f

*4-methyl-N-(2-phenyl-2-(p-tolyloxy)ethyl)aniline (**7f**).* Prepared from 2,4,6-triphenyl-1-((2-phenyl-2-(p-tolyloxy)ethyl)amino) pyridinium tetrafluoroborate (**6f**) and obtained as an orange solid (30 mg, 93% yield). The purification was done by alumina gel column chromatography using 95:5 hexanes:ethyl acetate as eluent. ^{1}H NMR (400 MHz, $CDCl_3$): δ 7.40–7.26 (m, 5H), 7.01–6.96 (m, 4H), 6.76–6.73 (m, 2H), 6.61–6.58 (m, 2H), 5.29 (dd, *J* = 7.4, 5.1 Hz, 1H), 4.06 (s, 1H), 3.55–3.46 (m, 2H), 2.25 (s, 3H), 2.22 (s, 3H). ^{13}C NMR (100 MHz,

CDCl3): δ 155.9, 145.5, 139.9, 130.5, 130.0, 129.9, 128.9, 128.1, 127.3, 126.3, 116.0, 113.8, 78.8, 51.6, 20.6, 20.5. HRMS-APCI (m/z): [M+1]+ calcd. for C22H24NO+, 318.1852; found, 318.1855.

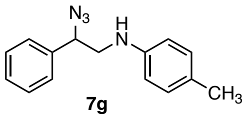

7g

*N-(2-azido-2-phenylethyl)-4-methylaniline (**7g**).* Prepared from 1-((2-azido-2-phenylethyl)amino)-2,4,6-triphenylpyridinium tetrafluoroborate (**6g**) and obtained as a pale-yellow liquid (25 mg, 98% yield). The purification was done by alumina gel column chromatography using 95:5 hexanes:ethyl acetate as eluent. ^{1}H NMR (400 MHz, $CDCl_3$): δ 7.45–7.34 (m, 5H), 7.02 (d, *J* = 8.0 Hz, 2H), 6.60 (d, *J* = 8.4 Hz, 2H), 4.77 (dd, *J* = 8.5, 5.2 Hz, 1H), 3.38 (qd, *J* = 13.4, 6.9 Hz, 2H), 2.26 (s, 3H). ^{13}C NMR (100 MHz, $CDCl_3$): δ 144.7, 137.7, 130.1, 129.2, 128.8, 127.9, 127.2, 113.8, 65.0, 50.2, 20.6. HRMS-APCI (m/z): [M+1]+ calcd. for C15H17N4+, 253.1448; found, 253.1444.

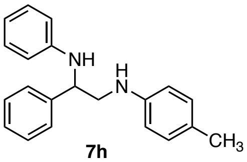

7h

*N^1,1-diphenyl-N^2-(p-tolyl)ethane-1,2-diamine (**7h**).* Prepared from 2,4,6-triphenyl-1-((2-phenyl-2-(phenylamino)ethyl)amino) pyridinium tetrafluoroborate (**6h**) and obtained as an colorless solid (17 mg, 56% yield). The purification was done by alumina gel column chromatography using 95:5 hexanes:ethyl acetate as eluent. ^{1}H NMR (400 MHz, $CDCl_3$): δ 7.41–7.33 (m, 4H), 7.28 (dt, *J* = 7.1, 2.0 Hz, 1H), 7.09 (dd, *J* = 8.6, 7.4 Hz, 2H), 7.03–7.00 (m, 2H), 6.69–6.62 (m, 3H), 6.57–6.54 (m, 2H), 4.62 (dd, *J* = 8.1, 4.8 Hz, 1H), 3.51–3.38 (m, 2H), 2.25 (s, 3H). ^{13}C NMR (100 MHz, $CDCl_3$): δ 147.3, 145.7, 141.6, 130.0, 129.3, 129.0, 127.7, 127.6, 126.6, 117.9, 113.8, 113.6, 57.6, 51.5, 20.5. HRMS-APCI (m/z): [M+1]+ calcd. for C21H23N2+, 303.1856; found, 303.1853.

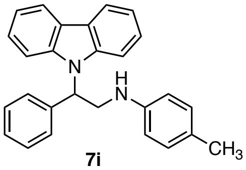

7i

*N-(2-(9H-carbazol-9-yl)-2-phenylethyl)-4-methylaniline (**7i**).* Prepared from 1-((2-(9H-carbazol-9-yl)-2-phenylethyl)amino)-2,4,6-triphenylpyridinium tetrafluoroborate (**6i**) and obtained as a colorless solid (37 mg, 98% yield). The purification was done by alumina gel

column chromatography using 95:5 hexanes:ethyl acetate as eluent. ^{1}H NMR (400 MHz, $CDCl_3$): δ 8.18 (d, *J* = 7.2 Hz), 7.45–7.34 (m, 11H), 7.02 (d, *J* = 8.0 Hz, 2H), 6.60 (d, *J* = 8.4 Hz, 2H), 6..21 (dd, *J* = 9.7, 4.9 Hz, 1H), 4.77 (dd, *J* = 8.5, 5.2 Hz, 1H), 3.38 (qd, *J* = 13.4, 6.9 Hz, 2H), 2.26 (s, 3H). ^{13}C NMR (100 MHz, $CDCl_3$): δ 144.7, 140.3, 138.2, 129.9, 129.0, 127.9, 127.7, 126.8, 125.8, 123.6, 120.4, 119.5, 113.8, 110.5, 56.3, 45.5, 20.5. HRMS-APCI (m/z): [M+1]+ calcd. for C27H25N2+, 377.2012; found, 377.2014.

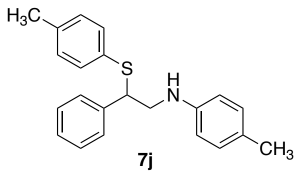

7j

*4-methyl-N-(2-phenyl-2-(p-tolylthio)ethyl)aniline (**7j**).* Prepared from 2,4,6-triphenyl-1-((2-phenyl-2-(p-tolylthio)ethyl)amino)pyridinium tetrafluoroborate (**6j**) and obtained as a pale-yellow liquid (28 mg, 85% yield). The purification was done by alumina gel column chromatography using 95:5 hexanes:ethyl acetate as eluent. ^{1}H NMR (400 MHz, $CDCl_3$): δ 7.33–7.26 (m, 5H), 7.24–7.22 (d, 2H), 7.06 (d, *J* = 7.9 Hz, 2H), 6.97 (d, *J* = 8.0 Hz, 2H), 6.46 (d, *J* = 8.4 Hz, 2H), 4.33 (t, *J* = 7.2 Hz, 1H), 3.63–3.49 (m, 2H), 2.32 (s, 3H), 2.25 (s, 3H). ^{13}C NMR (100 MHz, $CDCl_3$): δ 145.0, 140.2, 138.0, 133.5, 130.2, 129.9, 129.8, 128.8, 128.1, 127.7, 127.4, 113.7, 52.8, 49.1, 21.3, 20.5. HRMS-APCI (m/z): [M+1]+ calcd. for C22H24NS+, 334.1624; found, 334.1618.

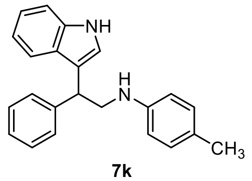

7k

*N-(2-(1H-indol-3-yl)-2-phenylethyl)-4-methylaniline (**7k**).* Prepared from 1-((2-(1H-indol-3-yl)-2-phenylethyl)amino)-2,4,6-triphenylpyridin-1-ium tetrafluoroborate (**6k**) and obtained as a pale-yellow liquid (15 mg, 43% yield). The purification was done by alumina gel column chromatography using hexane: ethyl acetate (5:1) as eluent. ^{1}H NMR (400 MHz, $CDCl_3$): δ 8.05 (s, 1H), 7.45 (d, *J* = 8.0 Hz, 1H), 7.38–7.28 (m, 5H), 7.24–7.16 (m, 2H), 7.10 (d, *J* = 2.4 Hz, 1H), 7.06–7.02 (m, 1H), 7.00–6.98 (m, 2H), 6.55–6.53 (m, 2H), 4.58 (t, *J* = 7.3 Hz, 1H), 3.86 (dd, *J* = 12.0, 7.3 Hz, 1H), 3.69 (t, *J* = 9.8 Hz, 1H), 3.64 (s, 1H), 2.24 (s, 3H). ^{13}C NMR (126 MHz, $CDCl_3$): δ 146.2, 143.2, 137.0, 130.2, 129.0, 128.6, 127.5, 127.21, 127.10, 122.8, 122.0, 120.03, 119.90, 117.8, 113.8, 111.6, 49.8, 42.7, 20.9. HRMS-ESI (m/z): [M+1]+ calcd. for C23H23N2+, 327.1856; found, 327.1846.

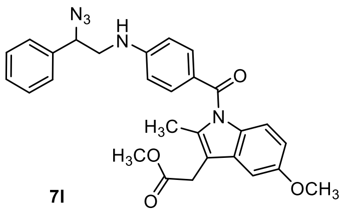

7l

*Methyl 2-(1-(4-((2-azido-2-phenylethyl)amino)benzoyl)-5-methoxy-2-methyl-1H-indol-3-yl)acetate (**7l**).* Prepared from 1-((2-azido-2-phenylethyl)amino)-2,4,6-triphenylpyridinium tetrafluoroborate (**6g**) and obtained as a yellow liquid (26 mg, 53% yield). The purification was done by alumina gel column chromatography using 5:1 hexanes: ethyl acetate as eluent. ^{1}H NMR (400 MHz, $CDCl_3$): δ 7.64–7.60 (m, 2H), 7.46–7.35 (m, 5H), 6.97–6.94 (m, 2H), 6.67 (dd, *J* = 9.0, 2.6 Hz, 1H), 6.63–6.59 (m, 2H), 4.78 (dd, *J* = 8.3, 5.2 Hz, 1H), 4.56 (t, *J* = 5.3 Hz, 1H), 3.84 (s, 3H), 3.70 (s, 3H), 3.69 (s, 2H), 3.54–3.42 (m, 2H), 2.42 (s, 3H). ^{13}C NMR (100 MHz, $CDCl_3$): δ 171.8, 168.9, 155.6, 151.6, 137.0, 136.3, 133.1, 131.4, 130.2, 129.3, 129.2, 127.1, 123.8, 114.8, 112.1, 111.4, 110.9, 101.0, 65.0, 55.9, 52.2, 48.7, 30.4, 13.0. HRMS-APCI (m/z): [M+1]+ calcd. for C28H28N5O4+, 498.2136; found, 498.2133.

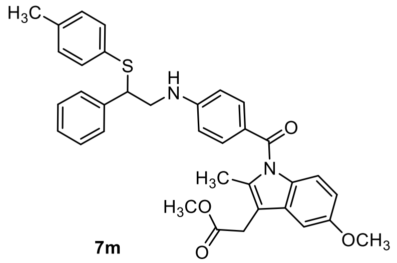

7m

*Methyl 2-(5-methoxy-2-methyl-1-(4-((2-phenyl-2-(p-tolylthio)ethyl)amino)benzoyl)-1H-indol-3-yl)acetate (**7m**).* Prepared from 2,4,6-triphenyl-1-((2-phenyl-2-(p-tolylthio)ethyl)amino)pyridinium tetrafluoroborate (**6j**) and obtained as a yellow liquid (50 mg, 86% yield). The purification was done by alumina gel column chromatography using 5:1 hexanes:ethyl acetate as eluent. ^{1}H NMR (400 MHz, $CDCl_3$): δ 7.56 (d, *J* = 8.7 Hz, 2H), 7.36–7.23 (m, 7H), 7.08 (d, *J* = 7.9 Hz, 2H), 6.97–6.93 (m, 2H), 6.66 (dd, *J* = 9.0, 2.5 Hz, 1H), 6.47–6.44 (m, 2H), 4.51 (t, *J* = 6.0 Hz, 1H), 4.31 (t, *J* = 7.2 Hz, 1H), 3.84 (s, 3H), 3.70 (s, 3H), 3.68 (s, 2H), 3.71–3.57 (m, 2H), 2.42 (s, 3H), 2.31 (s, 3H). ^{13}C NMR (100 MHz, $CDCl_3$): δ 171.8, 169.0, 155.6, 151.7, 139.5, 138.5, 136.3, 133.7, 133.1, 131.4, 130.2, 130.00, 129.8, 129.0, 128.1, 128.0, 123.4, 114.7, 112.1, 111.4, 110.8, 100.9, 55.9, 53.0, 52.2, 47.9, 30.4, 21.3, 12.9. HRMS-APCI (m/z): [M+1]+ calcd. for C35H35N2O4S+, 579.2312; found, 579.2310.

II. Supplementary Discussion

C. Additional Data

C.1. Optimization of Vinyl Arene Aziridination

Optimization tables for Procedure A

Supplementary Table 1. Examination of the Impact of Solvent on Aziridination Efficiency.

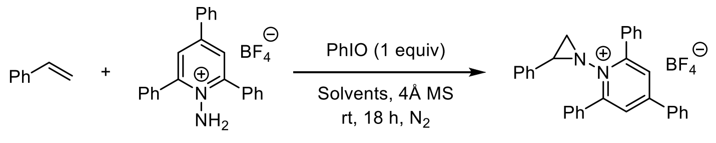

Entry	Solvents	NMR yield
**1**	**MeCN**	**49%**
2	^{t}BuCN	19%
3	^{i}PrOAc	7%
4	PhMe	0
5	PhH	0
6	Propylene carbonate	26%
7	Propylene carbonate (10% in MeCN)	34%

Supplementary Table 2. *Catalyst Screen for Styrene Aziridination Reaction.* Reactions were carried out using 0.2 mmol of styrene, 0.2 mmol of *N*-aminotriphenylpyridinium tetrafluoroborate, and 0.2 mmol of iodosylbenzene with 1 mL of acetonitrile in an N_2 filled dry box. NMR yields were determined by adding 5~10 mg of 1,3,5-trimethoxybenzene as the internal standard to the crude mixture after reaction workup.

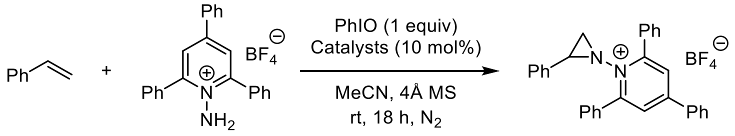

Entry	Catalysts	NMR yield
1	None, under air	49%
2	CuI	53%
3	CuI (30 mol%)	53%
4	$Cu(MeCN)_4PF_6$	16%
5	$Cu(OTf)_2$	20%
6	$Fe(OTf)_2$	0
7	MnTPPCl	4%
8	FeTPPCl	8%
9	$Rh_2(esp)_2$	35%
10	$Rh_2(tfacam)_4$	22%
11	AgOTf	25%
12	TBAI 5 mol%, I_2 10 mol% (under air)	21%
13	CuCl or CuBr	18%
14	CuI, BPhen	64%
15	$Cu(MeCN)_4(PF_6)_2$, TBAI	19%
**16**	**TBAI (5 mol%)**	**73%**
17	KI (5 mol%)	18%
18	CsI (5 mol%)	27%
19	TEAI (5 mol%)	71%
20	Ph_4PI (5 mol%)	36%

Supplementary Table 3. *Screen of Catalyst and Reagent Loading for Aziridination with 3-Nitrostyrene.* Reactions were carried out using 0.2 mmol of 3-nitrostyrene with 1 mL of acetonitrile in an N_2 filled dry box. NMR yields were determined by comparing the product peak against the methylene peak from tetrabutylammonium iodide.

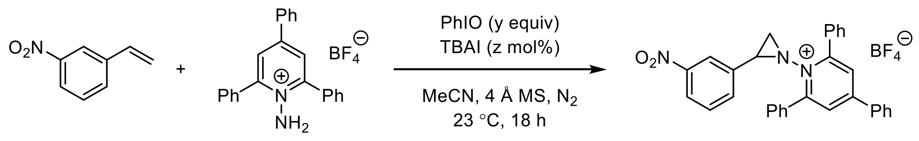

Entry	Py*–NH_2 equiv	PhIO equiv	TBAI loading	Time	NMR yield
0	1	1	5 mol%	18 h	40%
1	1	1.4	5 mol%	48 h	40%
2	1	1	15 mol%	18 h	49%
3	1.4	1.4	15 mol%	18 h	52%
**4**	**1.4**	**1.4**	**21 mol%**	**18 h**	**58%**

TBAI (mol%) / Reagent (eq)	15	20	24	27	30	50
1.4	52%	58%	45%	45%	58%	48%
1.6	61%	**67%**	58%	51%	41%	
1.8	38%	61%	45%			
2.0	42%	39%				

Supplementary Table 4. Head-to-Head NMR Yield Comparison of Procedure A with Procedure B for Styrene Aziridination.

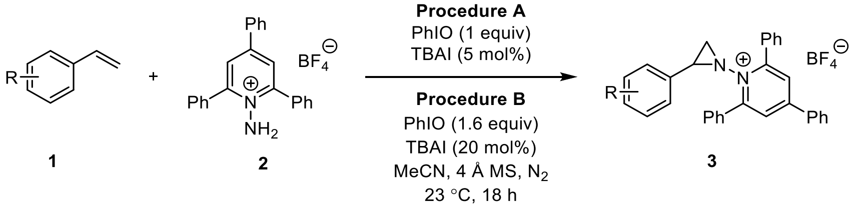

R	Procedure A	Procedure B
H	73%	77%
2-Me	69%	99%
2-Br	61%	82%
2-OMe	74%	70%
3-Me	69%	84%
3-Br	40%	71%
3-Cl	51%	58%
3-NO_2	40%	82%
4-Me	87%	95%
4-F	79%	83%
4-CF_3	32%	72%
4-AcO	62%	97%
4-CO_2Et	58%	87%
4-NHBoc	55%	56%
4-Ph	82%	70%
4-CN	46%	94%
4-OMe	59%	45%
4-Ac	75%	--
Isatin	77%	90%
Benzothiophene	62%	60%
2-Naph	85%	70%

C.2 Optimization of Pyridinium Aziridine Cross-Couplings

Supplementary Table 5. Screen of Catalyst and Ligand Loading.

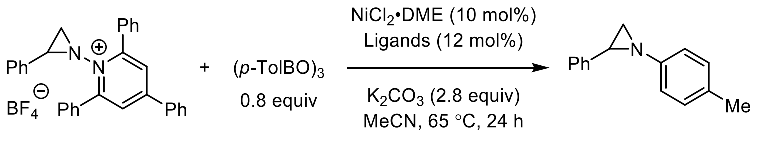

Entry	Catalyst loading	Ligands	NMR yield
1	10 mol%	4,4'-dmbpy, 12 mol%	0
2	10 mol%	1,10-Phen, 12 mol%	5%
3	10 mol%	1,10-Phen, 20 mol%	0
4	20 mol%	1,10-Phen, 20 mol%	36%
**5**	**30 mol%**	**1,10-Phen, 30 mol%**	**67%**
6	30 mol%	BPhen, 32 mol%	0
7	30 mol%	3,4,7,8-Me_4-1,10-Phen, 32 mol%	22%
8	30 mol%	2,9-Me_2-1,10-Phen, 32 mol%	43%
9	30 mol%	terpy, 32 mol%	44%
10	30 mol%	dppf, 32 mol%	29%
11	30 mol%	PCy_3, 32 or 62 mol%	0
12	30 mol%	XPhos, 32 mol%	0

Supplementary Table 6. Investigation of Nucleophiles in Transmetalation.

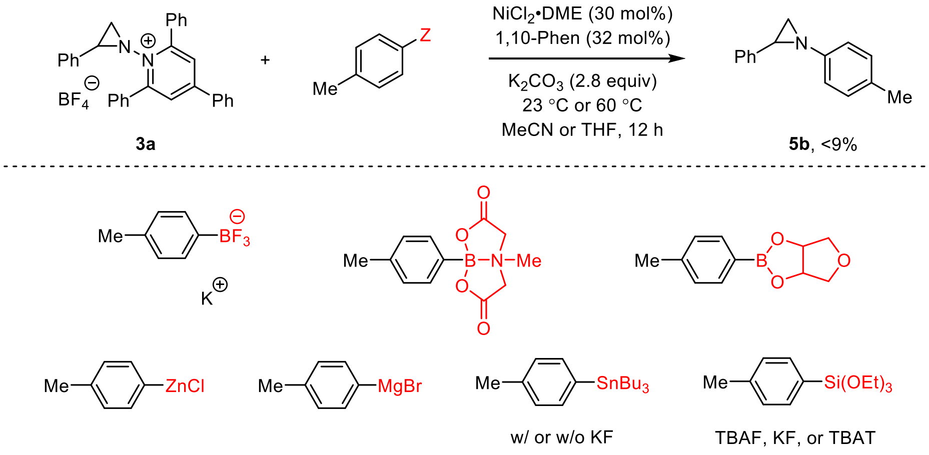

Supplementary Table 7. Investigation of reductive cross-electrophile coupling.

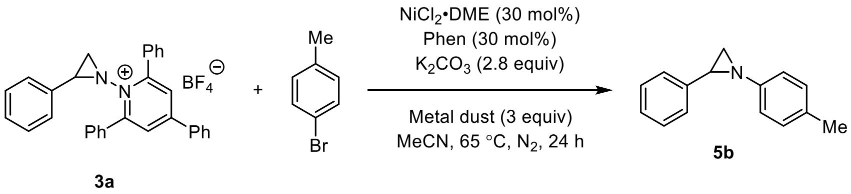

Entry	Metal dust	NMR Yield
1	Mn	50%
2	Zn	11%

The reactions were carried out according to the literature with slight modifications.[23] A 20-mL scintillation vial was charged with $NiCl_2$•DME (5.3 mg, 0.024 mmol, 30 mol%),1,10-phenanthroline (4.3 mg, 0.024 mmol, 30 mol%), potassium carbonate (31 mg, 0.22 mmol, 2.8 equiv), *p*-bromotoluene (16 mg ,0.096 mmol, 1.2 equiv),metal dust (0.24 mmol, 3.0 equiv) pyridinium aziridine **3a** (41 mg, 1 equiv, 0.08 mmol) and a magnetic stir bar. In an N_2 filled dry box, 1.0 mL of acetonitrile was added to the scintillation vial. The reaction vial was heated at 65 °C for 24 h. 1,3,5-Trimethoxybenzene was added to the crude mixture as an internal standard and the yield was determined by crude 1H NMR spectroscopy.

Supplementary Table 8. Variations with Solvent and Bases.

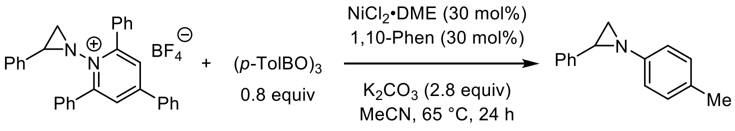

3a **5b**

Entry	Variations	NMR yield
1	1,4-dioxane	Trace
2	THF	0
3	K_3PO_4	57%
4	Cs_2CO_3	0
5	KO^tBu	0
6	KF (2.8 equiv as base)	53%
7	KF (1 equiv as additive)	49%

Supplementary Table 9. Investigation of Precatalyst.

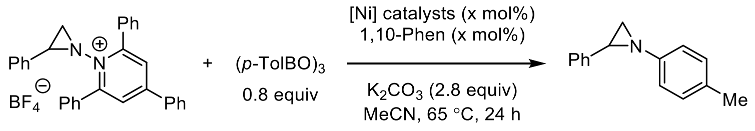

Entry	Catalysts	NMR yield
**1**	**$NiBr_2$·DME (20 mol%)**	**60%**
2	$Ni(OAc)_2$ (30 mol%)	0
3	$Ni(OAc)_2 \cdot 4H_2O$ (30 mol%)	13%
4	$Ni(acac)_2$ (30 mol%)	0
5	$Ni(cod)_2$ (30 mol%)	--
6	$Ni(Cp)_2$ (20 mol%)	27%
7	$NiSO_4 \cdot 6H_2O$ (20 mol%)	0
8	$NiCl_2$ (20 mol%)	0
9	NiF_2 (20 mol%)	0
10	NiI_2 (20 mol%)	17%
**11**	**$Ni(Phen)Br_2$ (20 mol%)[6]**	**60%**
12	$Ni(Phen)_3Br_2$ (20 mol%)[6]	0

[6] 1,10-Phenanthroline was not added.

Supplementary Table 10. *Screen of N-Containing Additives.* Reactions were carried out using 0.08 mmol of pyridinium aziridine with 1 mL of acetonitrile in an N_2 filled dry box. NMR yields were determined by adding 5~10 mg of 1,3,5-trimethoxybenzene as the internal standard to the crude mixture after reaction workup.

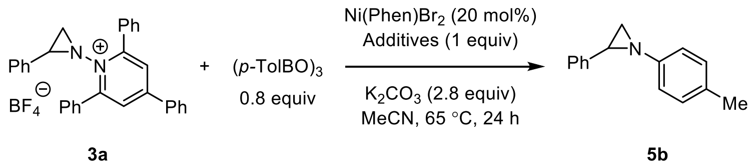

Entry	Additives	NMR yield
1	2,4,6-Triphenylpyridine	70%
**2**	**2,4,6-Collidine**	**79%**
3	DABCO	Trace
4	DBU	40%
5	Et_3N	13%
6	Imidazole	4%
7	2,6-Lutidine	0
8	DIPEA	17%

C.3. Stereochemistry of Aziridination with *trans*- and *cis*-β-Methylstyrene

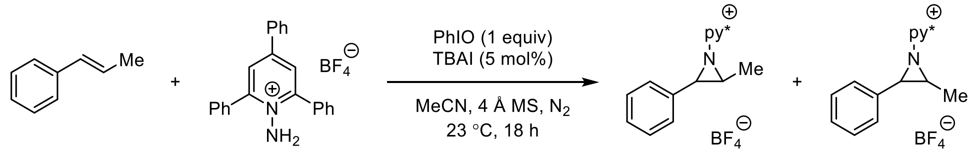

trans-**1u** **2** *trans*-**3u** : *cis*-**3u** = 55:45

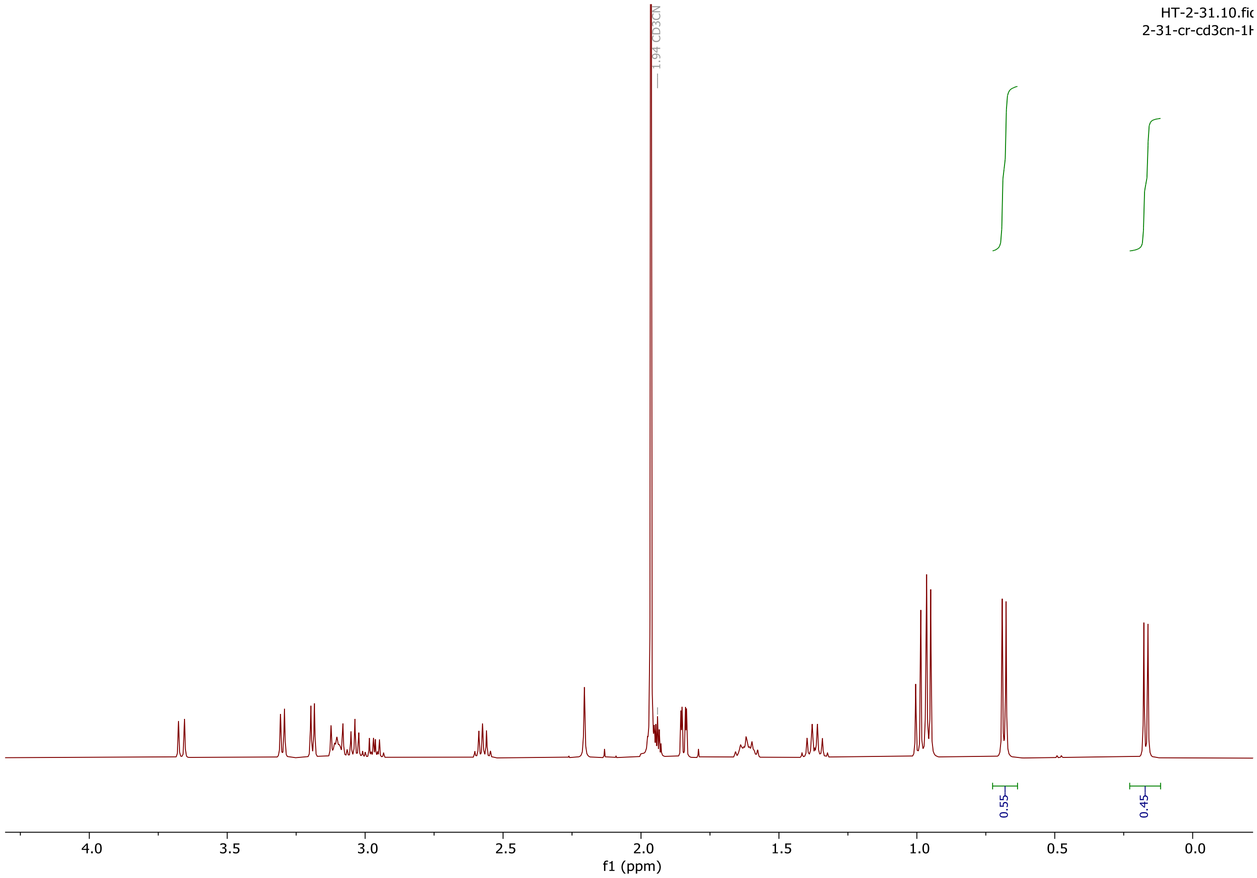

Supplementary Figure 1. ^{1}H NMR spectrum of the crude mixture of the aziridination of *trans*-**1u** in CD_3CN. The reaction was carried out on 0.2 mmol scale (based on *trans*-**1u**) according to the general procedure A indicated in section **B.5**. The doublets at δ 0.68 and δ 0.17 were assigned as the methyl peaks in *trans*-**1u** and *cis*-**1u** respectively, the integration of which indicated the ratio of *trans*-**1u** to *cis*-**1u**. The chemical shifts and the coupling constants used to assign the formed products were similar as their analogs, 2-methyl-3-phenyl-1-tosylaziridine (*cis*- and *trans*) that were reported in literature.[24, 25] The observed diastereoselectivity is not impacted by the exclusion of ambient light.

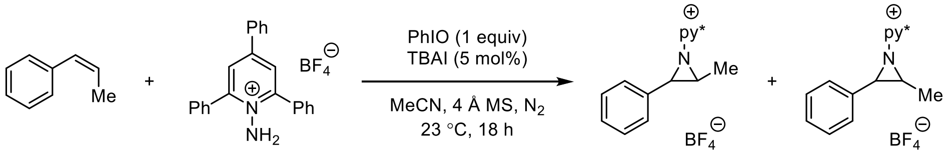

cis-**1u** **2** *trans*-**3u** : *cis*-**3u** = 65:35

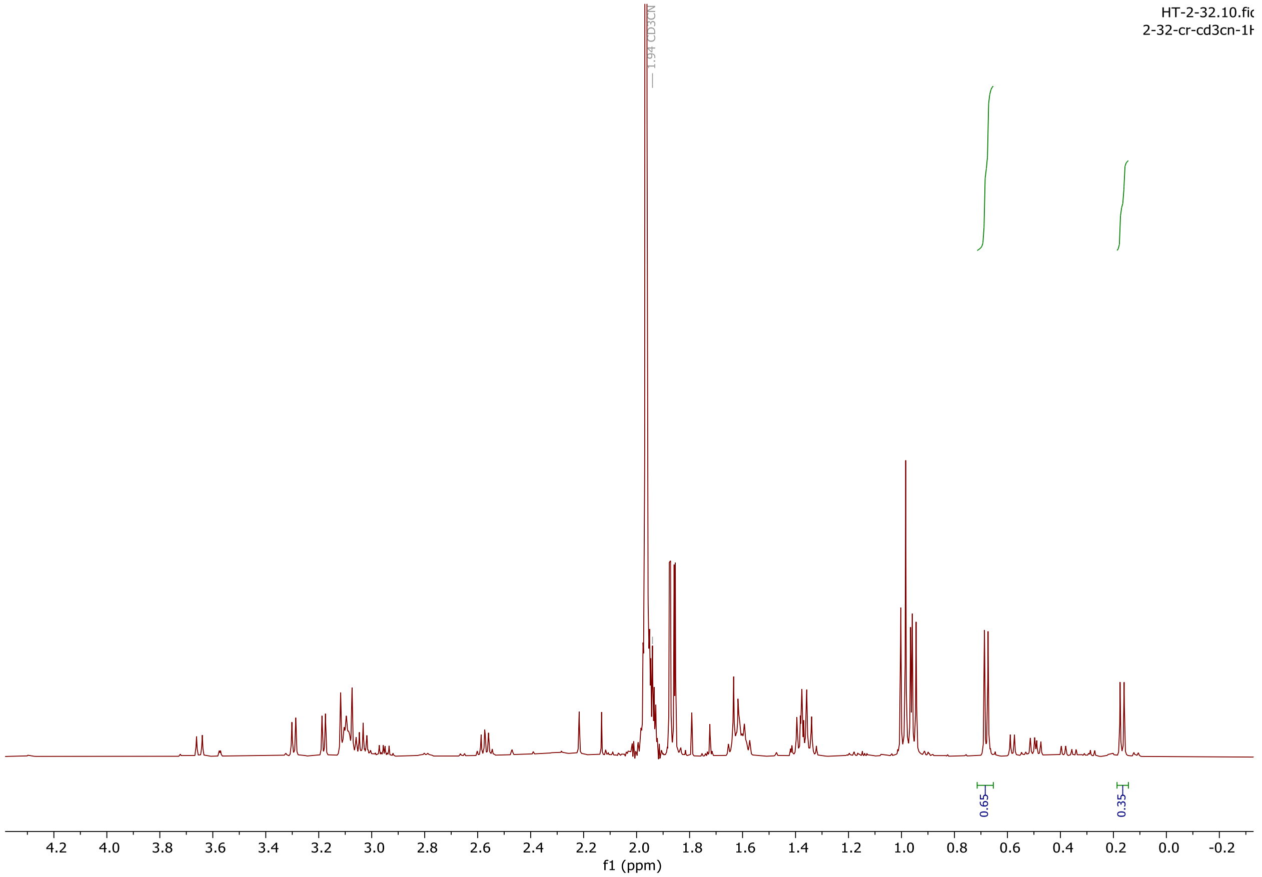

Supplementary Figure 2. ^{1}H NMR spectrum of the crude mixture of the aziridination of *cis*-**1u** in CD_3CN. The reaction was carried out on 0.2 mmol scale (based on *cis*-**1u**) according to the general procedure A indicated in section **B.5**. The doublets at δ 0.68 and δ 0.17 were assigned as the methyl peaks in *trans*-**3u** and *cis*-**3u** respectively, the integration of which indicated the ratio of *trans*-**3u** to *cis*-**3u.** The chemical shifts and the coupling constants used to assign the formed products were similar as their analogs, 2-methyl-3-phenyl-1-tosylaziridine (*cis*- and *trans*) that were reported in literature.[24, 25] The observed diastereoselectivity is not impacted by the exclusion of ambient light.

C.4. Observation of Pyridinium Aziridine Ring-Opening under Cross-Coupling Conditions.

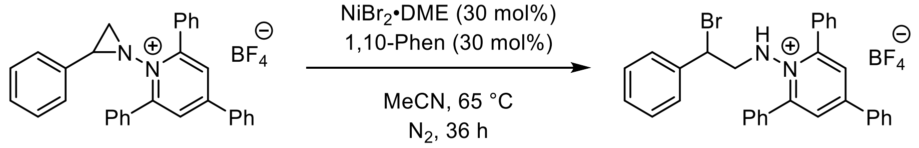

3a **6a**

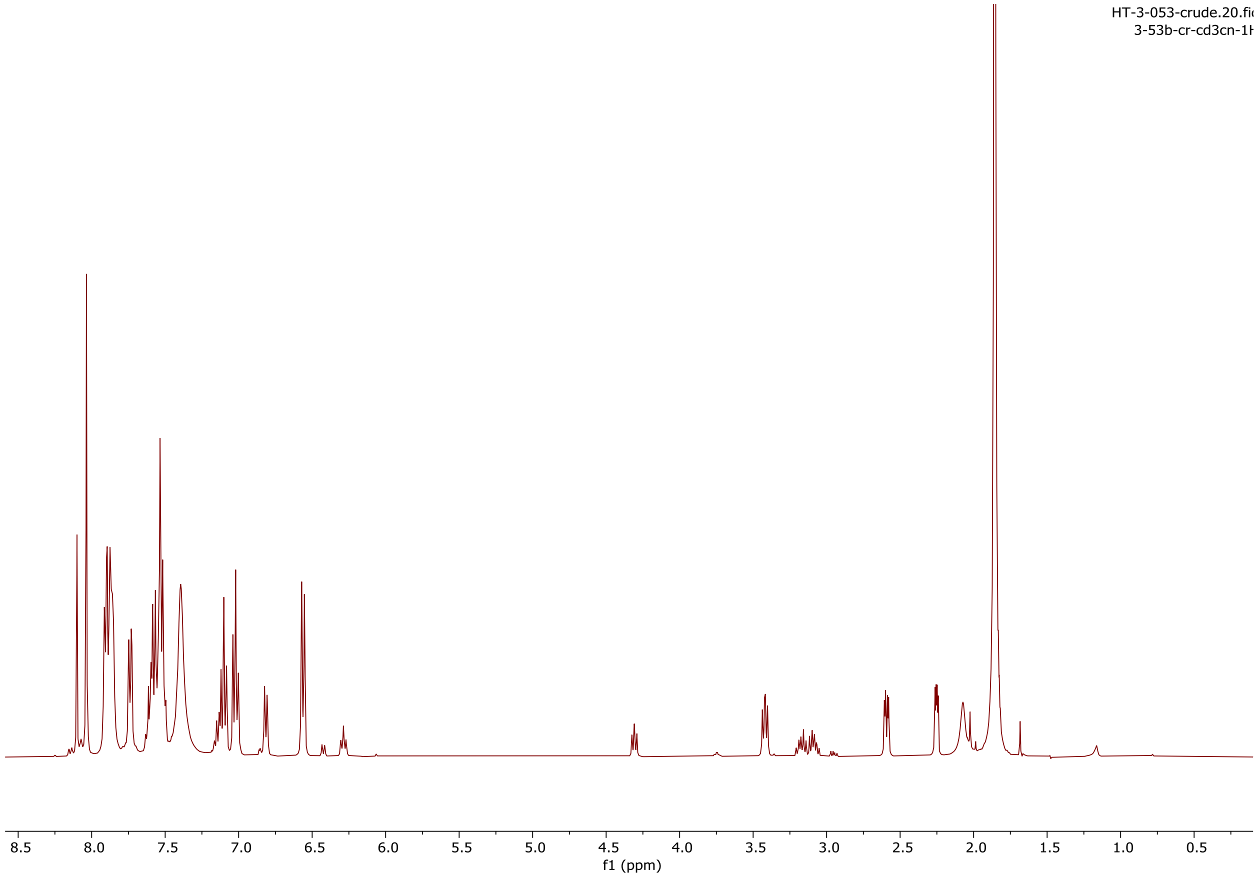

Supplementary Figure 3. ^{1}H NMR spectrum of the crude mixture of **3a**, $NiBr_2$(dme), and 1,10-phenanthroline in CD_3CN. The reaction was carried out on 0.1 mmol scale (based on **3a**). The doublet of doublet peak at δ 3.53 was assigned to unreacted **3a**. Formation of **6a** was indicated by observation of a doublet of doublet peak at δ 4.42.

C.5 Ring-Closing and Cross-Coupling with NiX_2.

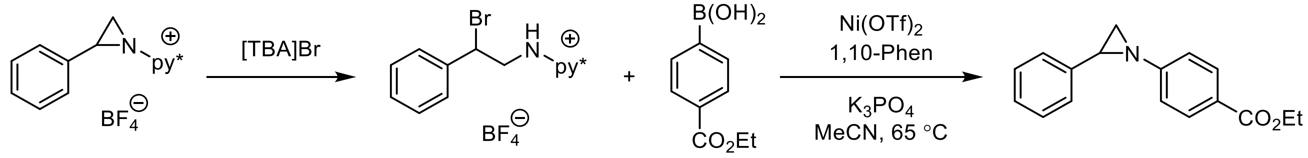

3a **6a** **5j**

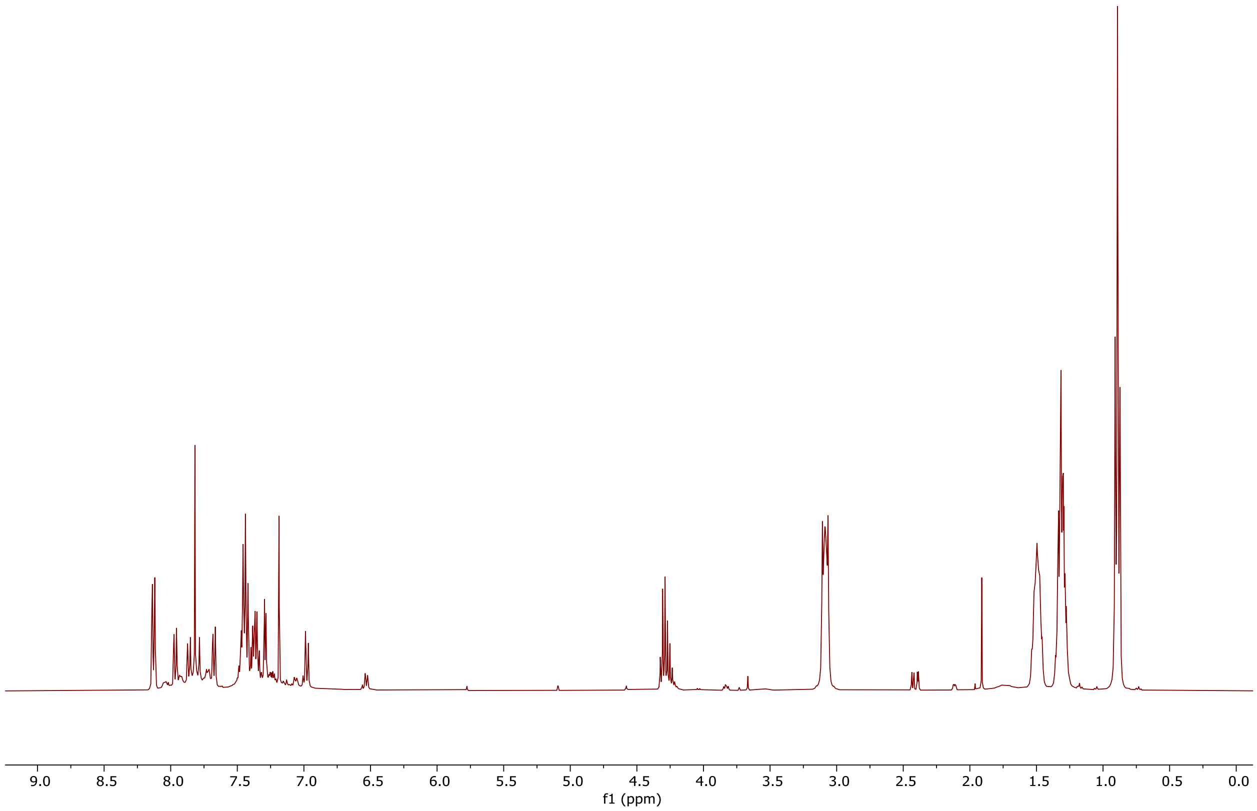

Supplementary Figure 4. ^1H NMR spectrum of the crude mixture following cross-coupling in $CDCl_3$. **6a** was formed by reacting **3a** with [TBA]Br according to the general procedure in section **B.7** on 0.1 mmol scale (based on **6a**). The reaction mixture was concentrated under reduced pressure and then dissolved in dry acetonitrile without further purification. The crude solution was subjected to cross-coupling condition as indicated in section **B.8**. Formation of aziridine product **5j** was indicated by the observation of the doublet of doublet peaks at δ 2.50 and δ 2.46.

C.6 Attempts of Aliphatic Alkene Aziridination using *N*-Aminopyridinium Reagents.

Supplementary Table 11. Failed aziridination of aliphatic alkenes with aminopyridinium **2** under differenct conditions. Reactions were carried out on 0.20 mmol scale (based on **2**) using 1.0 equiv of cyclohexene or allylbenzene under N_2.

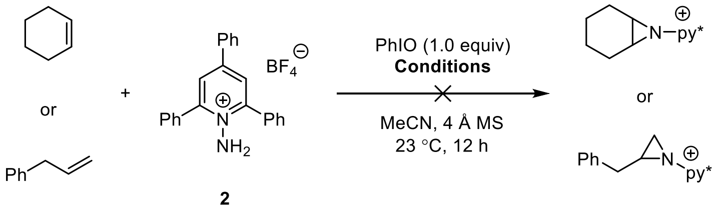

Entry	Conditions
1	CuI (10 mol%), BPhen (10 mol%)
2	CuI (10 mol%)
3	CuI (10 mol%), Phen (10 mol%)
4	MnTPPCl (10 mol%)
5	Rh_2esp_2 (5 mol%)
6	$Rh_2tfacam_4$ (5 mol%)
7	TBAI (5 mol%)
8	No catalyst, under air

Supplementary Table 12. Failed aziridination of alkenes with aminopyridinium salts **2** or **S3** by TBAI catalysis. Reactions were carried out on 0.20 mmol scale (based on **2** or **S3**) using 1.0 equiv of corresponding alkenes under N_2.(Note: for cis-2-hexene, the aziridination occurred with **2** but the aziridine product could not be isolated in analytical purity.)

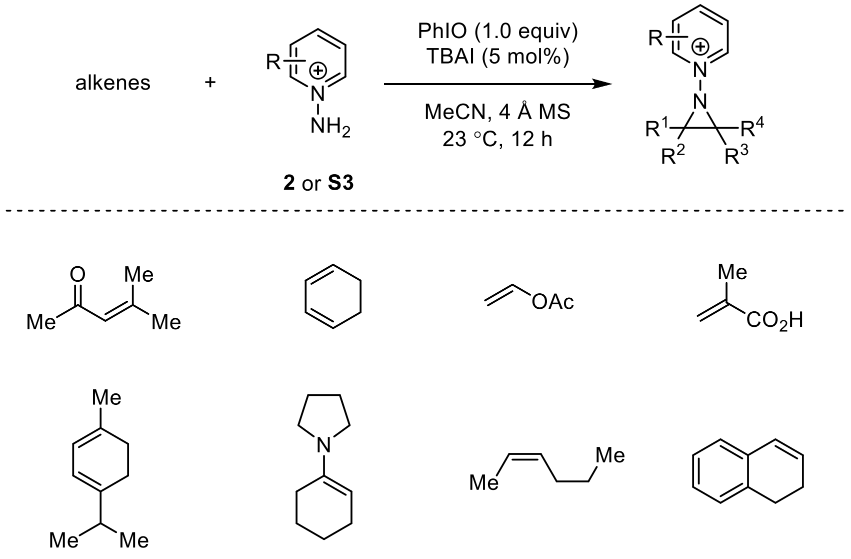

C.7 Cross-Coupling Reactions using 10 mol% of Nickel Catalyst

Supplementary Table 13. NMR yields of selected substrates when 10 mol% of [Ni] and 1 equiv or 10 mol% of additional [TBA]Br were used. Reactions were carried out using 0.08 mmol of pyridinium aziridine with 1 mL of acetonitrile in an N_2 filled drybox. NMR yields were determined by integration of 2,4,6-triphenylpyridine (byproduct) and the product peaks.

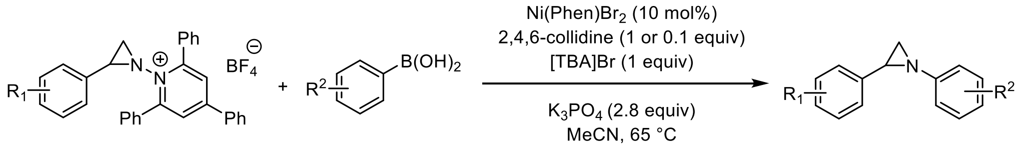

Entry	R_1, R_2	10 mol%	1 equiv	NMR yield (parent condition)
1	R_1 = H, R_2 = H, **5a**	Quant.	35%	70%
2	R_1 = H, R_2 = 4-Ph, **5c**	66%	52%	80%
3	R_1 = H, R_2 = 4-Cl, **5g**	40%	60%	49%
4	R_1 = H, R_2 = 4-CO_2Et, **5j**	63%	61%	72%
5	R_1 = 4-Me, R_2 = 4-Br, **5m**	33%	77%	72%
6	R_1 = 4-Ph, R_2 = 4-Br, **5n**	50%	66%	50%
7	**5w**	--	46%	30%
8	**5x**	--	59%	40%
9	**5y**	--	72%	47%

Supplementary Table 14. NMR yields of selected substrates when 10 mol% of [Ni] was used. Reactions were carried out using 0.08 mmol of **3a** with 1 mL of acetonitrile in an N_2 filled drybox. NMR yields were determined by integration of 2,4,6-triphenylpyridine (byproduct) and the product peaks.

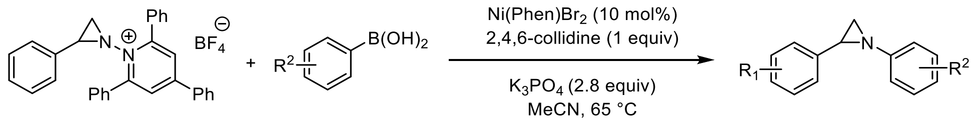

Entry	R2	NMR yield with 10 mol% Ni	NMR yield of the parent condition
1	H, **5a**	87%	70%
2	4-Me, **5b**	66%	79%
3	4-Ph, **5c**	79%	80%
4	4-MeO, **5d**	0	71%
5	4-CHO, **5e**	<50%	99%
6	4-Br, **5f**	<30%	95%
7	4-Cl, **5g**	72%	49%
8	4-F, **5h**	93%	72%
9	4-CF_3, **5i**	<40%	59%
10	4-CO_2Et, **5j**	68%	77%
11	3-MeS, **5k**	58%	61%

C.8 Failed Cross-Coupling of 2-(2-Phenylaziridin-1-yl)isoindoline-1,3-dione and 2-Phenyl-1-tosylaziridine.

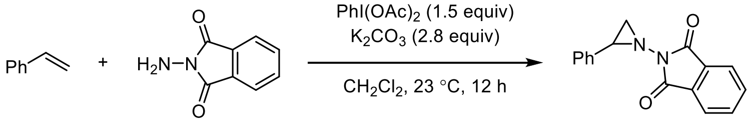

S4

2-(2-Phenylaziridin-1-yl)isoindoline-1,3-dione (**S4**) was prepare according to the literature.[26] A 100-mL flask was charged with styrene (520 mg, 5.00 mmol, 1.00 equiv), *N*-aminophthalimide (1.14 g, 7.00 mmol, 1.40 equiv), potassium carbonate (1.93 g, 14.0 mmol, 2.80 equiv) and 30.0 mL of dichloromethane. To this stirred suspension was added iodobenzene diacetate (2.42 g, 7.50 mmol, 1.50 equiv). The reaction was stirred at 23 °C for 12 h followed by filtration. The filtrate was concentrated under reduced pressure and the crude mixture was purified on silica gel flash chromatography (hexanes:EtOAc = 4:1) to yield a white solid (793 mg, 60% yield). ^{1}H NMR (499 MHz, $CDCl_3$) δ 7.80 (dd, *J* = 5.4, 3.1 Hz, 2H), 7.70 (dd, *J* = 5.5, 3.0 Hz, 2H), 7.49–7.44 (m, 2H), 7.38 (m, 2H), 7.35–7.31 (m, 1H), 3.62 (dd, *J* = 8.0, 5.9 Hz, 1H), 2.90 (dd, *J* = 8.0, 2.5 Hz, 1H), 2.80 (dd, *J* = 5.8, 2.5 Hz, 1H). These spectral data are well-matched to those reported in the literature.

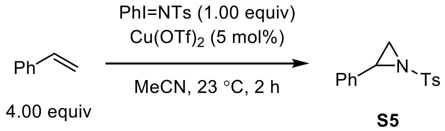

S5

2-Phenyl-1-tosylaziridine (**S5**) was prepare according to the literature.[25] A 50-mL flask was charged with styrene (446 mg, 4.28 mmol, 4.00 equiv), copper(II) triflate (19.3 mg, 0.0535 mmol, 5 mol%). In an N_2 filled glovebox, 10 mL of acetonitrile was added, followed by PhI=Ts (400 mg, 1.07 mmol, 1.00 equiv). The reaction was stirred at 23 °C for 2 h and filtered through a pad of celite. The filtrate was concentrated under reduced pressure and the crude mixture was purified on silica gel flash chromatography (hexanes:EtOAc = 10:1) to yield a pale-yellow solid (187 mg, 64% yield). ^{1}H NMR (499 MHz, $CDCl_3$) δ 7.88 (d, *J* = 8.3 Hz, 2H), 7.36–7.33 (m, 2H), 7.32–7.27 (m, 3H), 7.25–7.21 (m, 2H), 3.78 (dd, *J* = 7.2, 4.5 Hz, 1H), 2.99 (d, *J* = 7.2 Hz, 1H), 2.44 (s, 3H), 2.39 (d, *J* = 4.5 Hz, 1H). These spectral data are well-matched to those reported in the literature.

Supplementary Table 15. Attempts of cross-coupling of **S4** or **S5** using Phth and Ts as the potential leaving group. Reactions were carried out on 0.1 mmol scale (based on **S4** and **S5**) using 2.4 equiv of 4-ethoxycarbonyl phenylboronic acid under conditions indicated below. Crude NMR showed no formation of desired product **5j**.

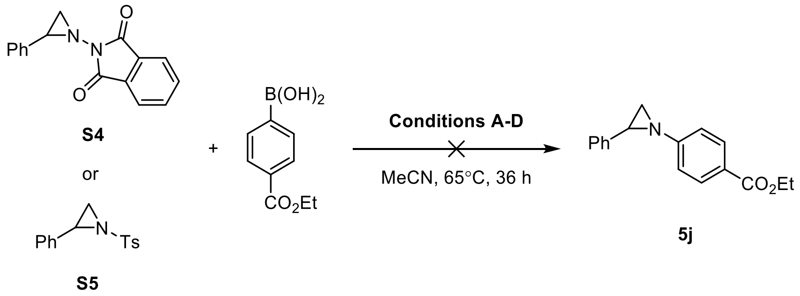

Entry	Conditions
A	Ni(Phen)Br_2 (20 mol%), collidine (1.0 equiv), K_3PO_4 (2.8 equiv)
B	$Pd_2(dba)_3$ (10 mol%), BINAP (20 mol%), K_2CO_3 (2.8 equiv)
C	$Cu(OTf)_2$ (20 mol%), phen (20 mol%), K_2CO_3 (2.8 equiv)
D	$Rh_2(esp)_2$ (10 mol%), K_2CO_3 (2.8 equiv)

C.9 Cross-Coupling of 2-Phenylaziridine (S8).

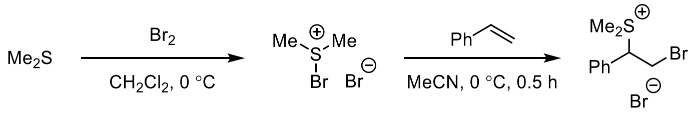

S6 S7

S6 was prepared according to the literature.[27] The solution of bromine (16.0 g, 0.100 mol) in dichloromethane (20.0 ml each) was added drop wised over 30 min into the ice-cooled solution of dimethyl sulfide(6.20 g, 0.100 mol) at 0 °C. The reaction was filtered and washed with diethyl ether (3×30 mL). The obtained orange crystal was dried under vacuum to yield **S6** (18.2 g, 82% yield).
Styrene (8.54 g,82.0 mmol) was added drop wise to the ice-cooled 85.0 mL CH_3CN solution of **S6** (18.2 g, 82.0 mmol) at 0 °C. The solution was stirred for 10 min after the addition of styrene was completed. The reaction was filtered and the obtained white solid was dried under vacuum to give **S7** (8.36 g, 31% yield).

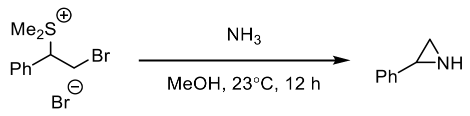

S7 S8

Ammonia in methanol (2.50 mL, 7N, 15.0 mmol, 5.00 equiv) was added drop wise to a stirred solution of **S7** (980 mg, 3.00 mmol, 1.00 equiv) in 7.0 mL of H_2O at room temperature and the resulting mixture was stirred overnight. The mixture was added into 10 mL of saturated brine, extracted with diethyl ether (3×10 mL), dried with anhydrous $NaSO_4$ and the solvent was evaporated under reduced pressure. The crude was purified by silica gel flash chromatography using 100% ethyl acetate as the eluent. 2-Phenylaziridine (**S8)** was isolated as a colorless oil (121 mg, 34% yield). ^{1}H NMR (400 MHz, $CDCl_3$) δ 7.35–7.28 (m, 2H), 7.26–7.20 (m, 3H), 3.02 (dd, J = 6.0, 3.4 Hz, 1H), 2.21 (d, J = 6.0 Hz, 1H), 1.82 (d, J = 3.4 Hz, 1H). These spectral data are well-matched to those reported in the literature.

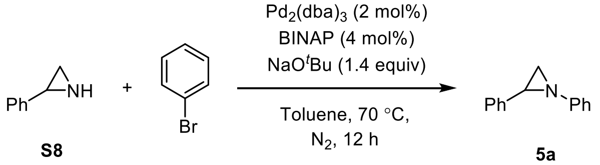

S8
1.2 equiv

5a
<5% nmr

The palladium catalyzed cross-coupling of 2-phenylaziridine (**S8**) was carried out according to the literature, on 0.10 mmol scale (based on bromobenzene).[28] The crude NMR yield was determined by adding 10.8 mg of 1,3,5-trimethoxybenzene as the internal standard and less than 5% of the desired product **5a** was present in the crude mixture.

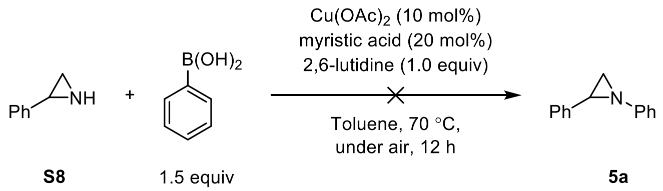

The copper(II)-catalyzed oxidative cross-coupling of 2-phenylaziridine (**S8**) was carried out according to the literature, on 0.10 mmol scale (based on **S8**).[28] The crude NMR was measured and no desired product **5a** was present in the mixture.

C.10 Attempts to Observe Intermediates in Iodide-Catalyzed Aziridination

Supplementary Table 16. *Exploration of potential oxidizing species in styrene aziridination with amino pyridinium **2***. Aminopyridinium (**2**, 0.10 mmol) in MeCN (1.0 mL) was added to oxidants (0.10 mmol) indicated in the table in an N_2 filled dry box, followed by addition of styrene (**1a**, 0.1 mmol). ^{1}H NMR showed unreacted **2** and no formation of **3a** as indicated in the top spectrum (experiment entry 1).

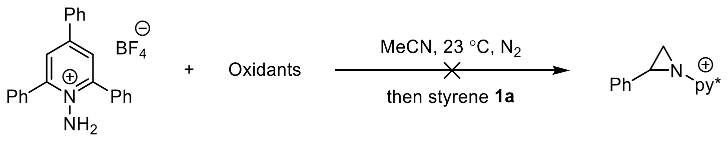

2 **3a**

Entry	Oxidants
1	I_2
2	[TBA]I_3
3	[TBA]IO

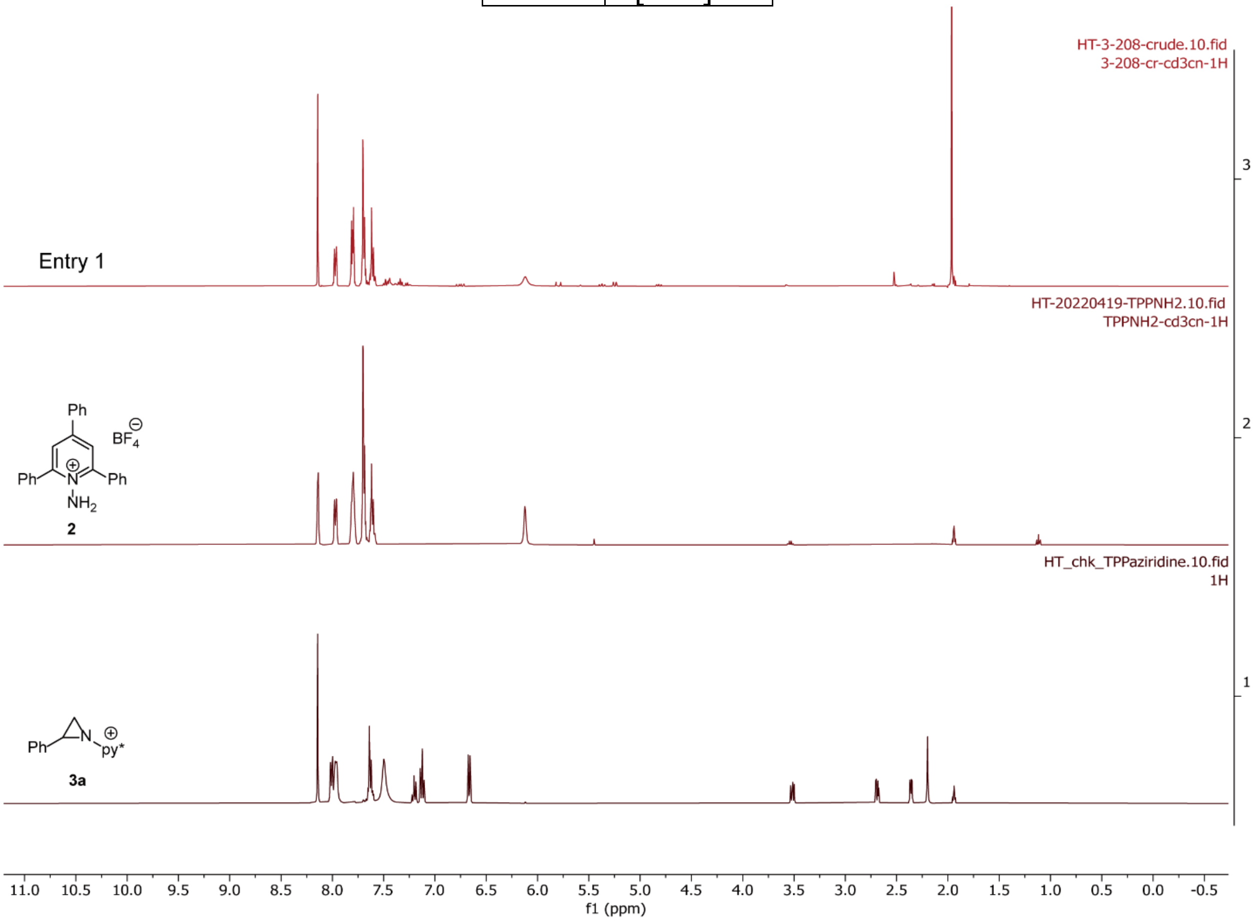

Supplementary Table 17. *Exploration of potential oxidizing species in styrene aziridination with amino pyridinium **2***. Aminopyridinium (**2**, 0.10 mmol) in CD_3CN (1.0 mL) was added to oxidants (0.10 mmol) indicated in the table in an N_2 filled dry box. ^1H NMR showed did not provide evidence for the formation of an observable intermediate. (Note: Entry 2 showed 50% decomposition of **2** into triphenyl pyridine, and no other observable intermediates.)

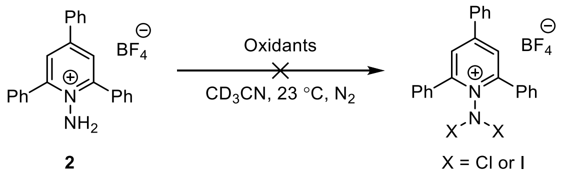

Entry	Oxidants
1	I_2
2	PhIO, TBAI (1.0 equiv)
3	$NaClO•5H_2O$, then I_2

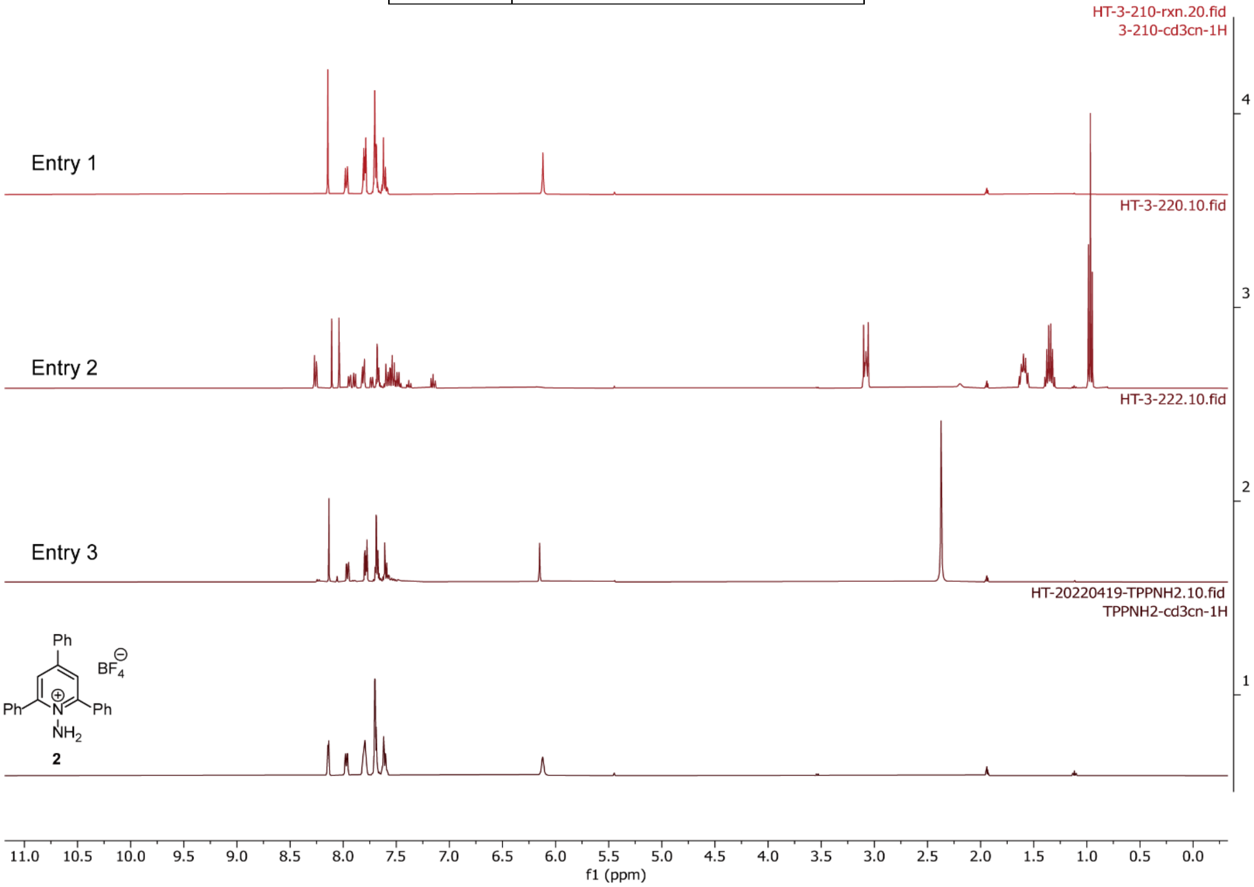

D. X-Ray Diffraction Data

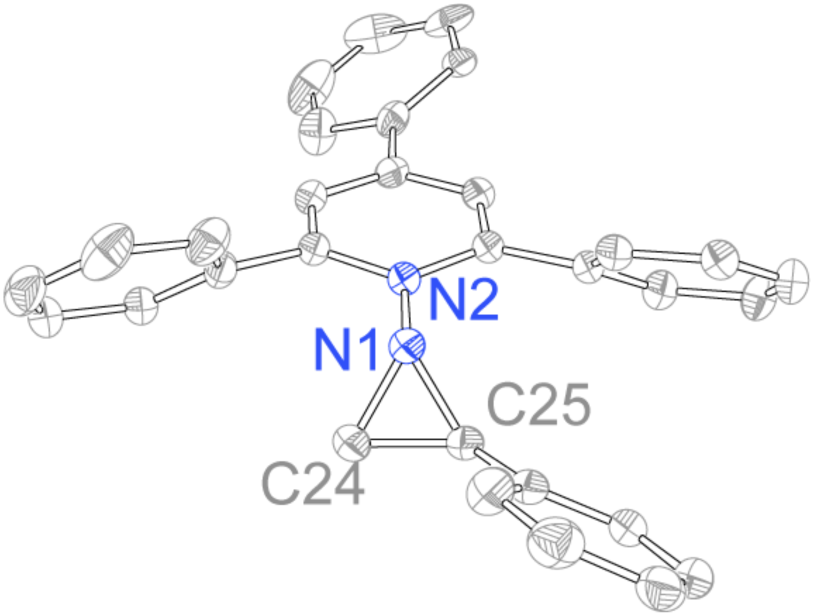

Supplementary Figure 5. Displacement ellipsoid plot of **3a** plotted at 50% probability. H-atoms, disordered phenyl ring and BF_4^- counterion are removed for clarity. The crystalline sample used for the diffraction experiment was obtained via pentane diffusion into an EtOAc/DCM (1:1) solution.

Supplementary Table 18. X-ray experimental details for **3a** (CCDC 2159504)

Crystal data	
Chemical formula	$BF_4{\cdot}C_{31}H_{25}N_2$
M_r	512.34
Crystal system, space group	Monoclinic, $P2_1/n$
Temperature (K)	110
a, b, c (Å)	12.2269(9), 12.9704(8), 16.356(1)
β (°)	104.726(2)
V (Å^3)	2508.7(3)
Z	4
Radiation type	Mo $K\alpha$
μ (mm^{-1})	0.10
Crystal size (mm)	0.52 × 0.31 × 0.13
Data collection	
Diffractometer	Bruker *APEX*-II CCD
Absorption correction	Multi-scan<br>*SADABS2016*/2 (Bruker,2016/2) was used for absorption correction. wR2(int) was 0.1181 before and 0.0657 after correction. The Ratio of minimum to maximum transmission is 0.9177. The λ/2 correction factor is not present.
T_{min}, T_{max}	0.685, 0.746
No. of measured, independent and found, [$I > 2\sigma(I)$] reflections	108461, 7363, 5819
R_{int}	0.062
$(\sin \theta/\lambda)_{max}$ (Å^{-1})	0.706
Refinement	
$R[F^2 > 2\sigma(F^2)]$, $wR(F^2)$, S	0.063, 0.173, 1.10
No. of reflections	7363
No. of parameters	454
H-atom treatment	H atoms treated by a mixture of independent and constrained refinement
$\Delta\rho_{max}$, $\Delta\rho_{min}$ (e Å^{-3})	0.38, −0.40

E. NMR Spectra for New Compounds

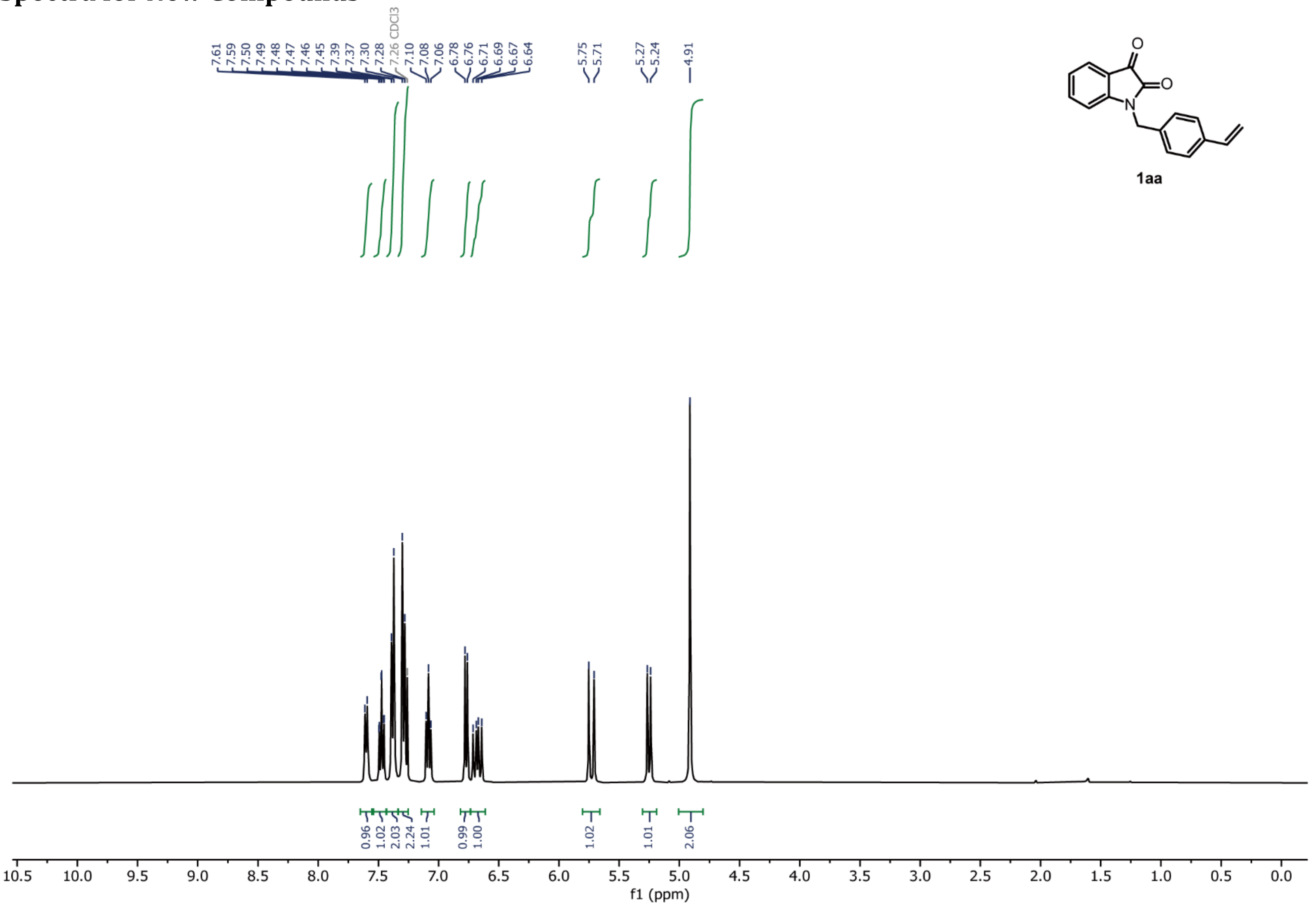

Supplementary Figure 6. ^{1}H NMR spectrum of 1-(4-vinylbenzyl)indoline-2,3-dione (**1aa**) in $CDCl_3$ (400 MHz) at 23 °C.

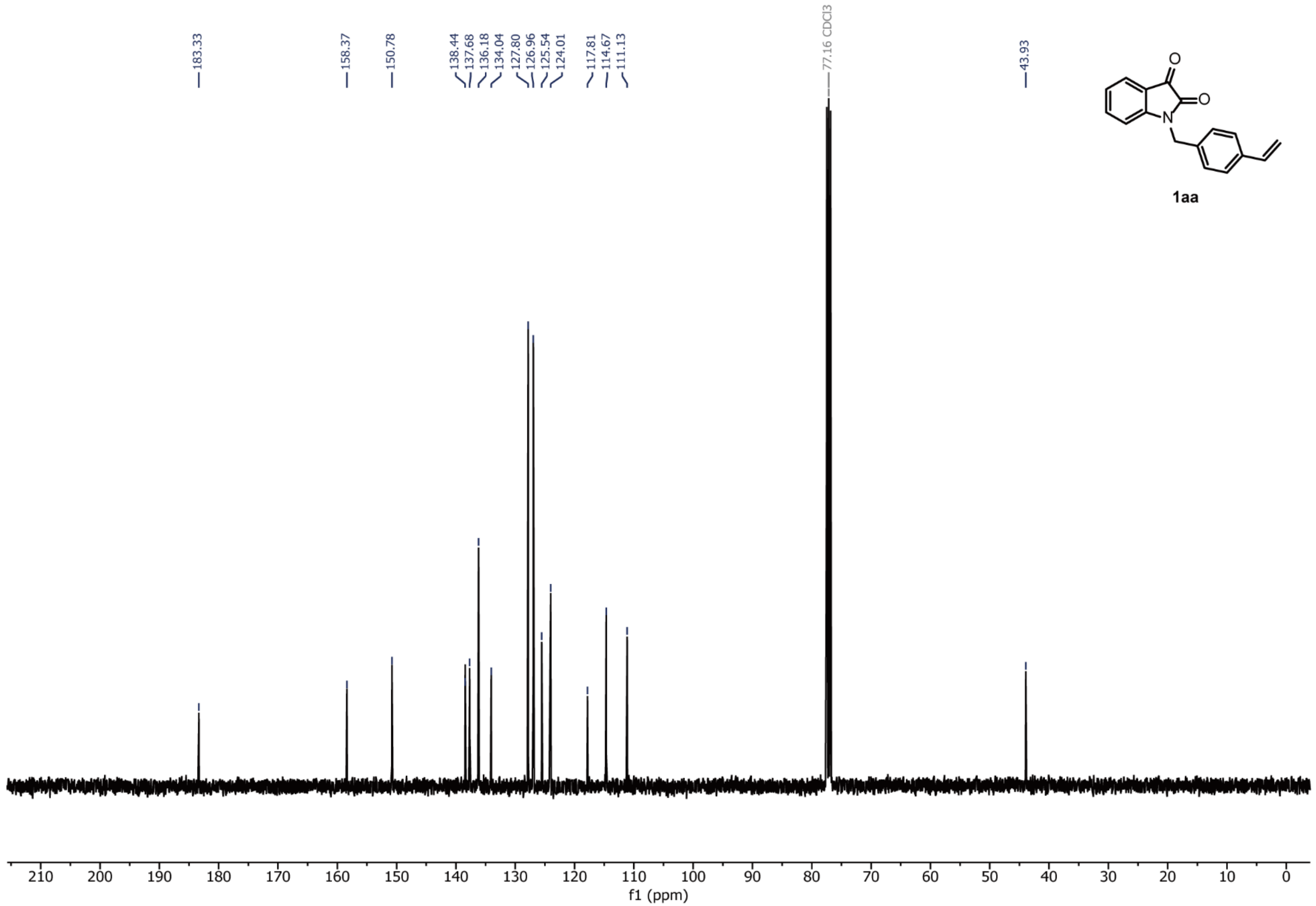

Supplementary Figure 7. ^{13}C NMR spectrum of 1-(4-vinylbenzyl)indoline-2,3-dione (**1aa**) in $CDCl_3$ (101 MHz) at 23 °C.

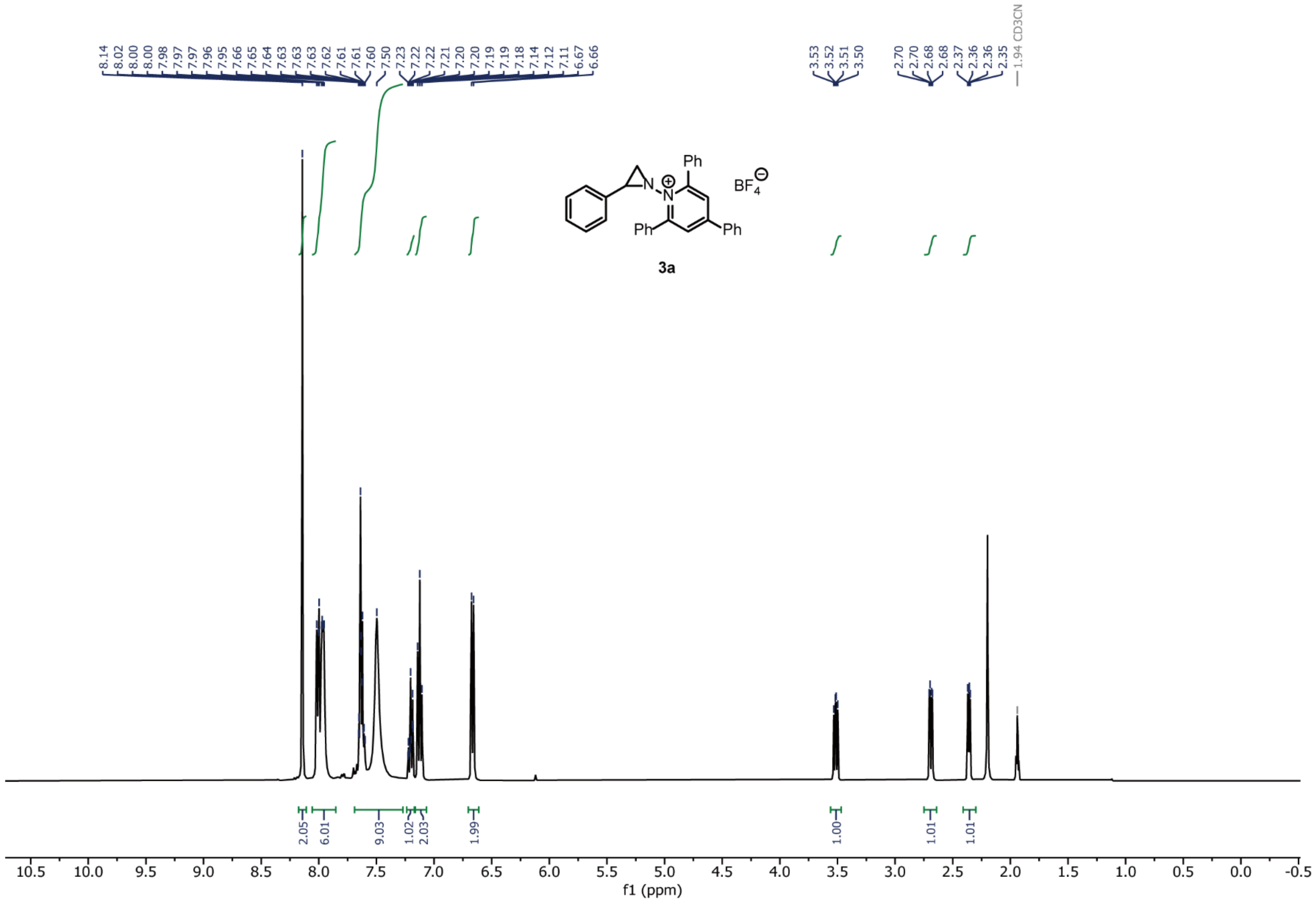

Supplementary Figure 8. ^{1}H NMR spectrum of 2,4,6-triphenyl-1-(2-phenylaziridin-1-yl)pyridin-1-ium tetrafluoroborate (**3a**) in CD_3CN (400 MHz) at 23 °C.

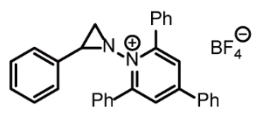

3a

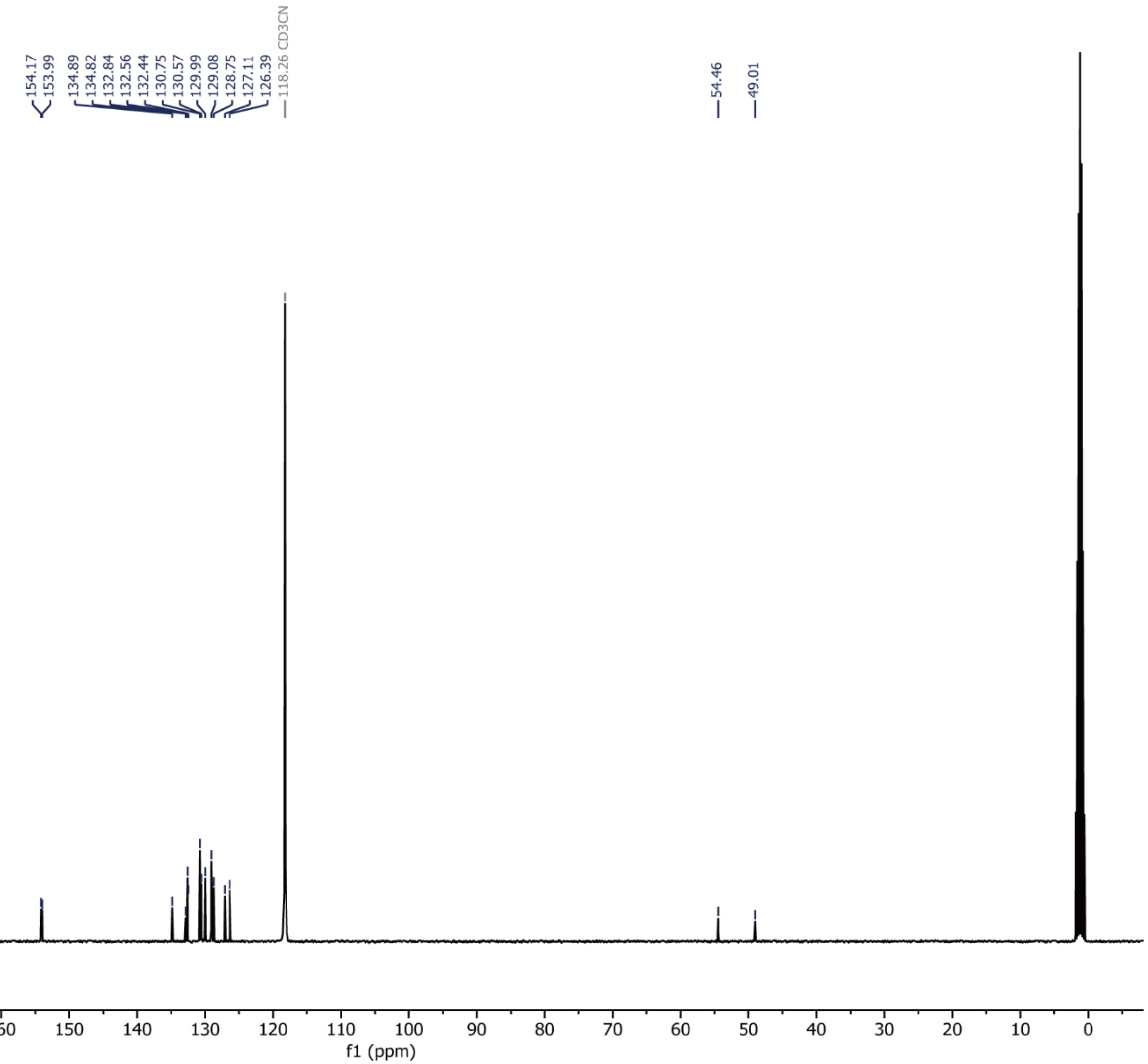

Supplementary Figure 9. ^{13}C NMR spectrum of 2,4,6-triphenyl-1-(2-phenylaziridin-1-yl)pyridin-1-ium tetrafluoroborate (**3a**) in CD_3CN (101 MHz) at 23 °C.

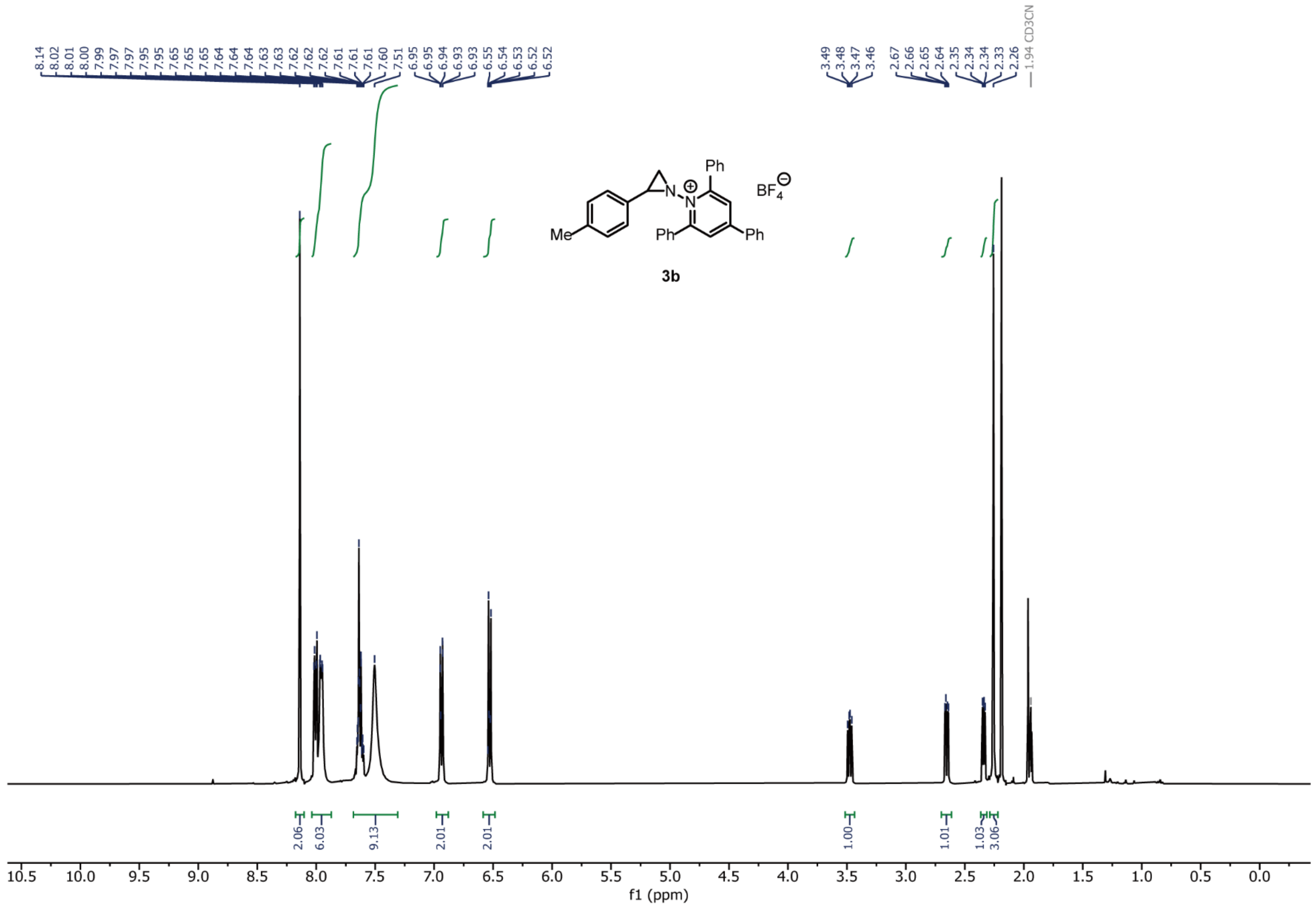

Supplementary Figure 10. ^{1}H NMR spectrum of 2,4,6-triphenyl-1-(2-(*p*-tolyl)aziridin-1-yl)pyridin-1-ium tetrafluoroborate (**3b**) in CD_3CN (400 MHz) at 23 °C.

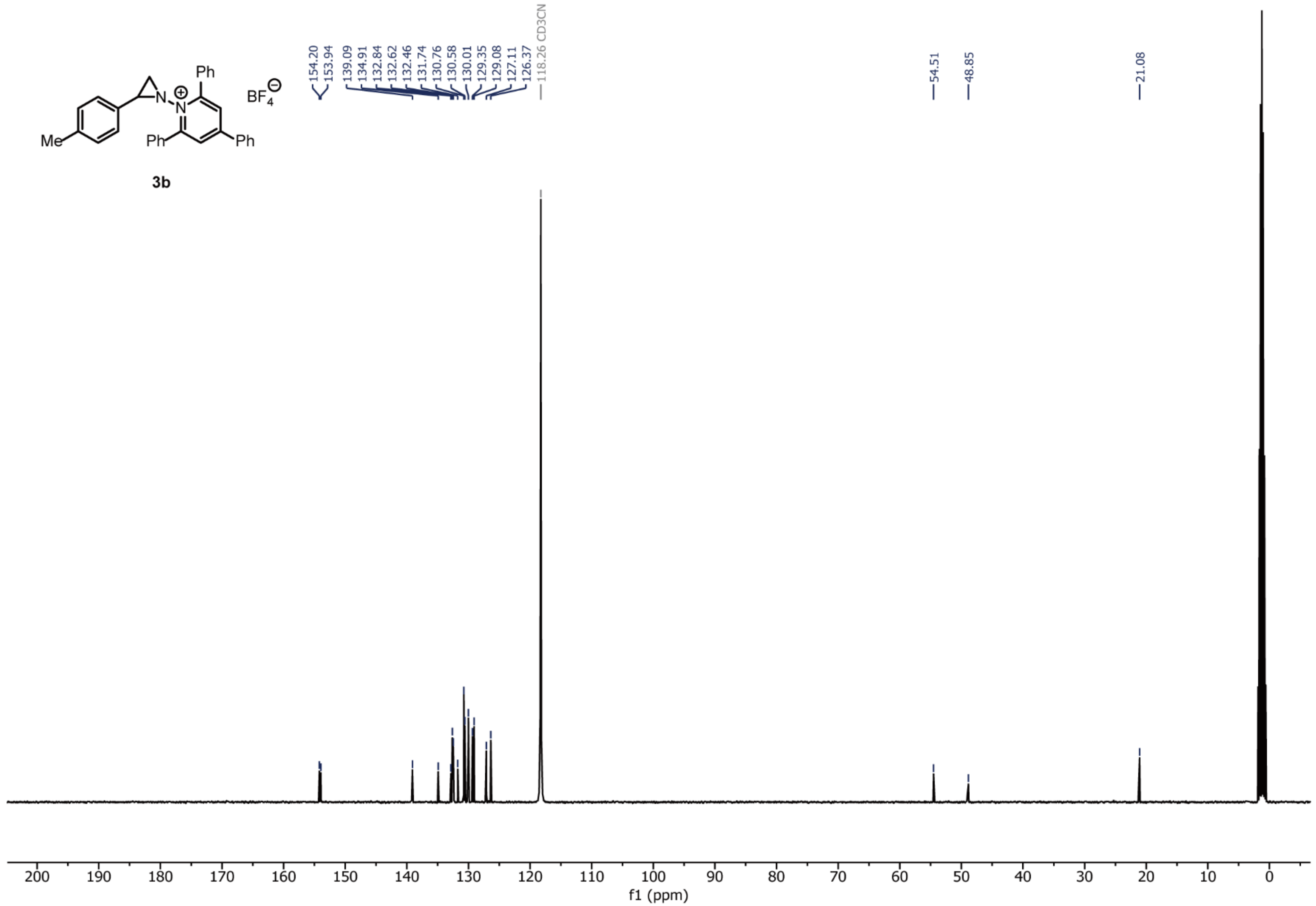

Supplementary Figure 11. ^{13}C NMR spectrum of 2,4,6-triphenyl-1-(2-(*p*-tolyl)aziridin-1-yl)pyridin-1-ium tetrafluoroborate (**3b**) in CD_3CN (101 MHz) at 23 °C.

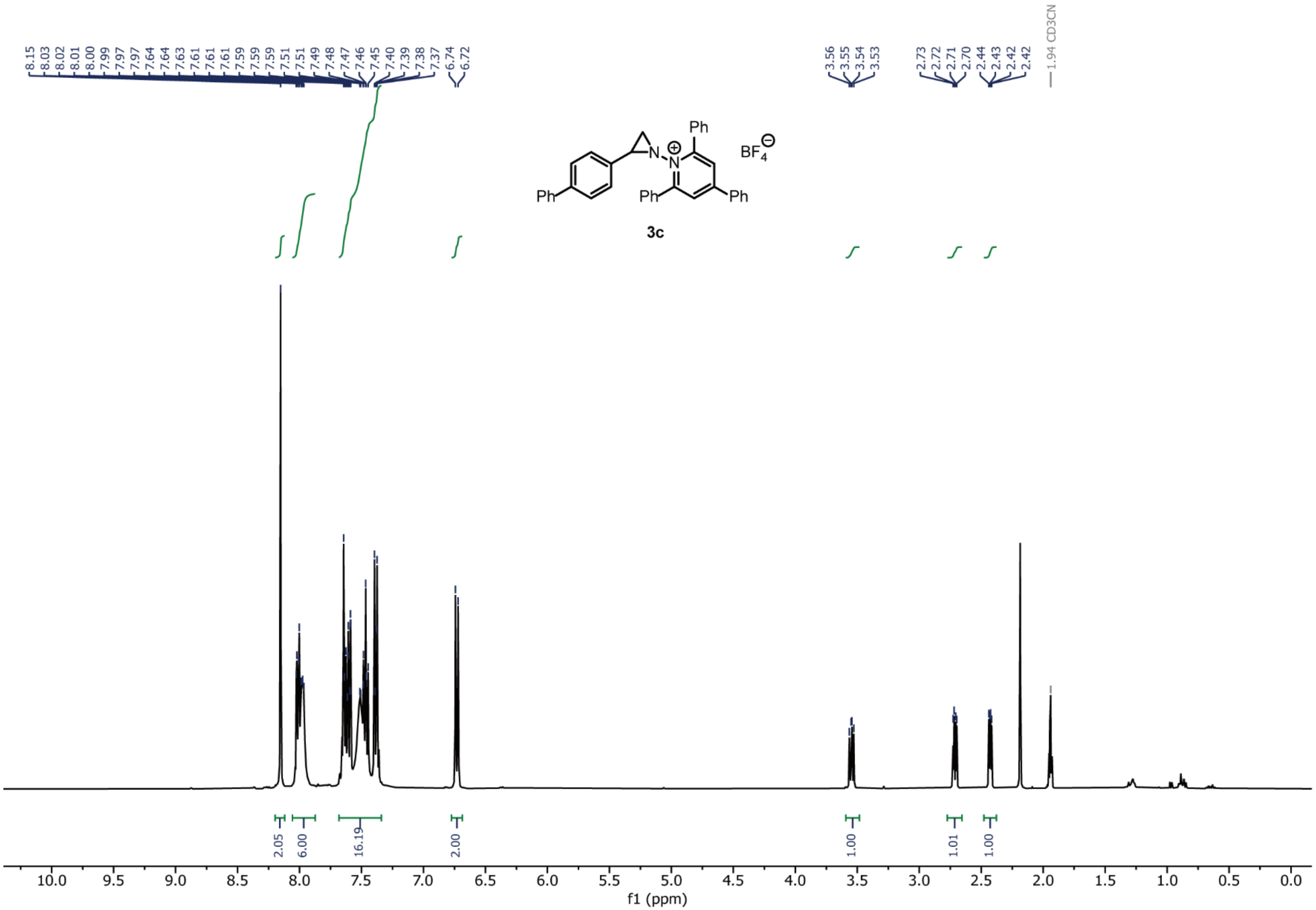

Supplementary Figure 12. ^{1}H NMR spectrum of 1-(2-([1,1'-biphenyl]-4-yl)aziridin-1-yl)-2,4,6-triphenylpyridin-1-ium tetrafluoroborate (**3c**) in CD_3CN (400 MHz) at 23 °C.

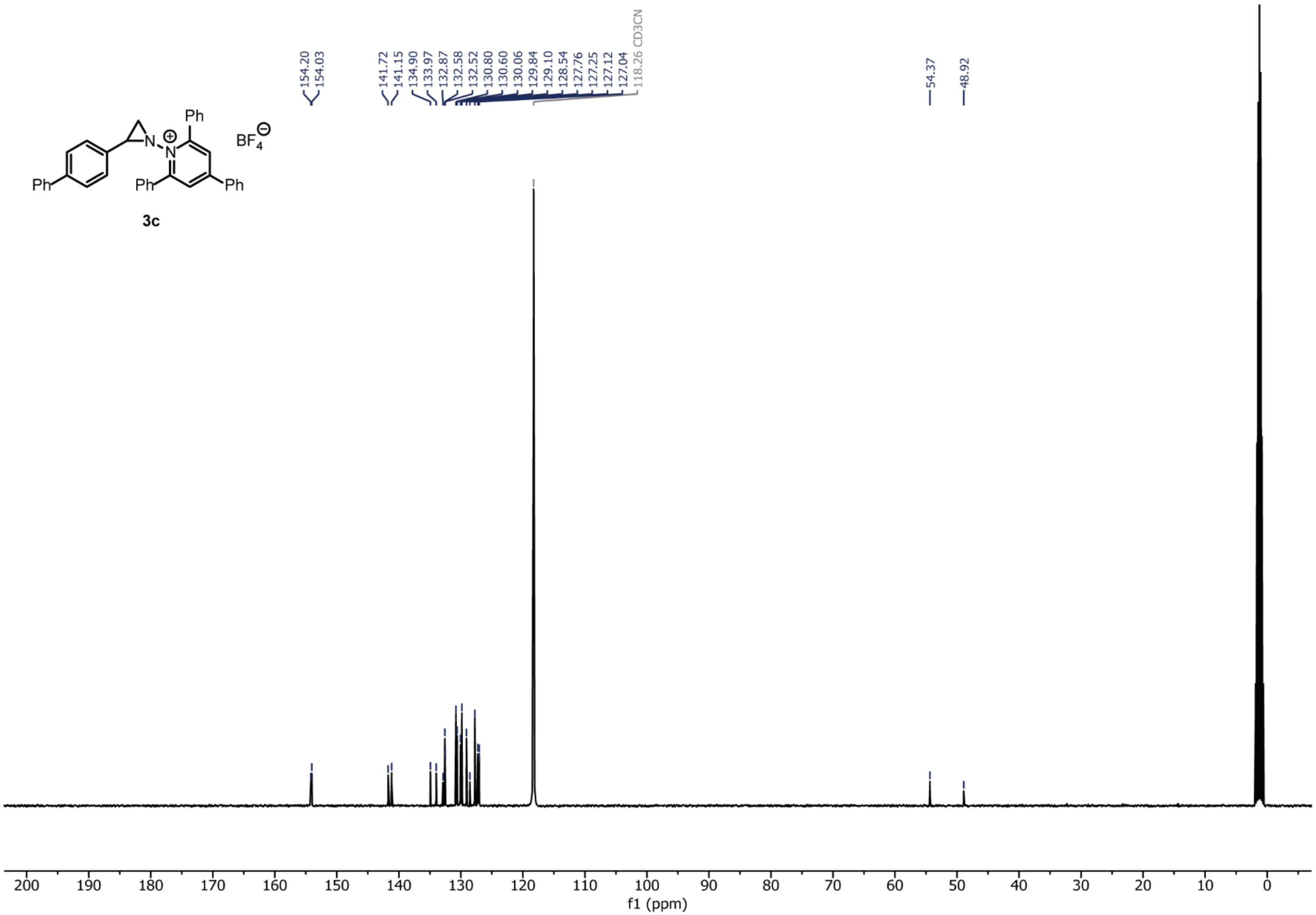

Supplementary Figure 13. ^{13}C NMR spectrum of 1-(2-([1,1'-biphenyl]-4-yl)aziridin-1-yl)-2,4,6-triphenylpyridin-1-ium tetrafluoroborate (**3c**) in CD_3CN (101 MHz) at 23 °C.

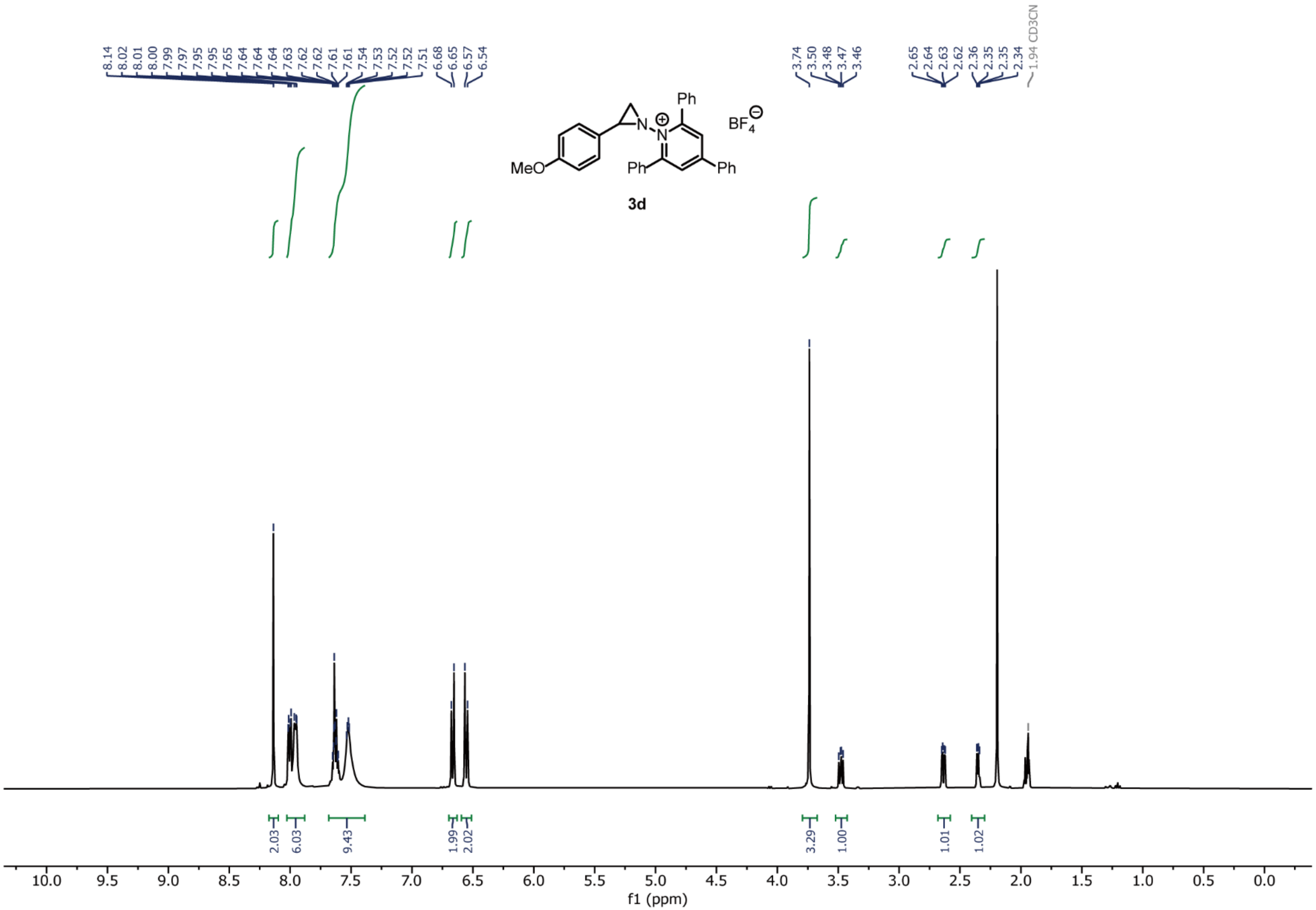

Supplementary Figure 14. ^{1}H NMR spectrum of 1-(2-(4-methoxyphenyl)aziridin-1-yl)-2,4,6-triphenylpyridin-1-ium tetrafluoroborate (**3d**) in CD_3CN (400 MHz) at 23 °C.

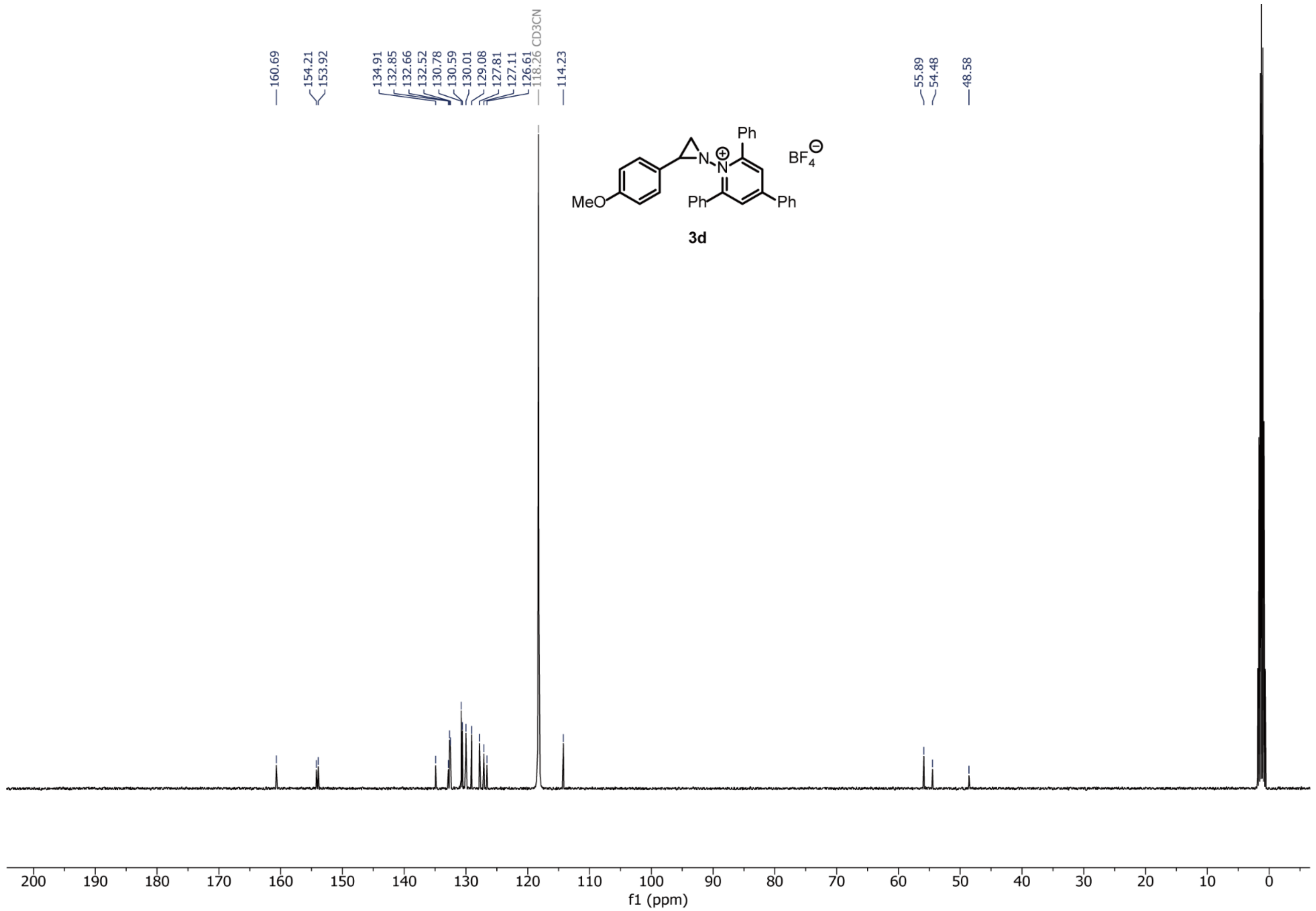

Supplementary Figure 15. ^{13}C NMR spectrum of 1-(2-(4-methoxyphenyl)aziridin-1-yl)-2,4,6-triphenylpyridin-1-ium tetrafluoroborate (**3d**) in CD_3CN (101 MHz) at 23 °C.

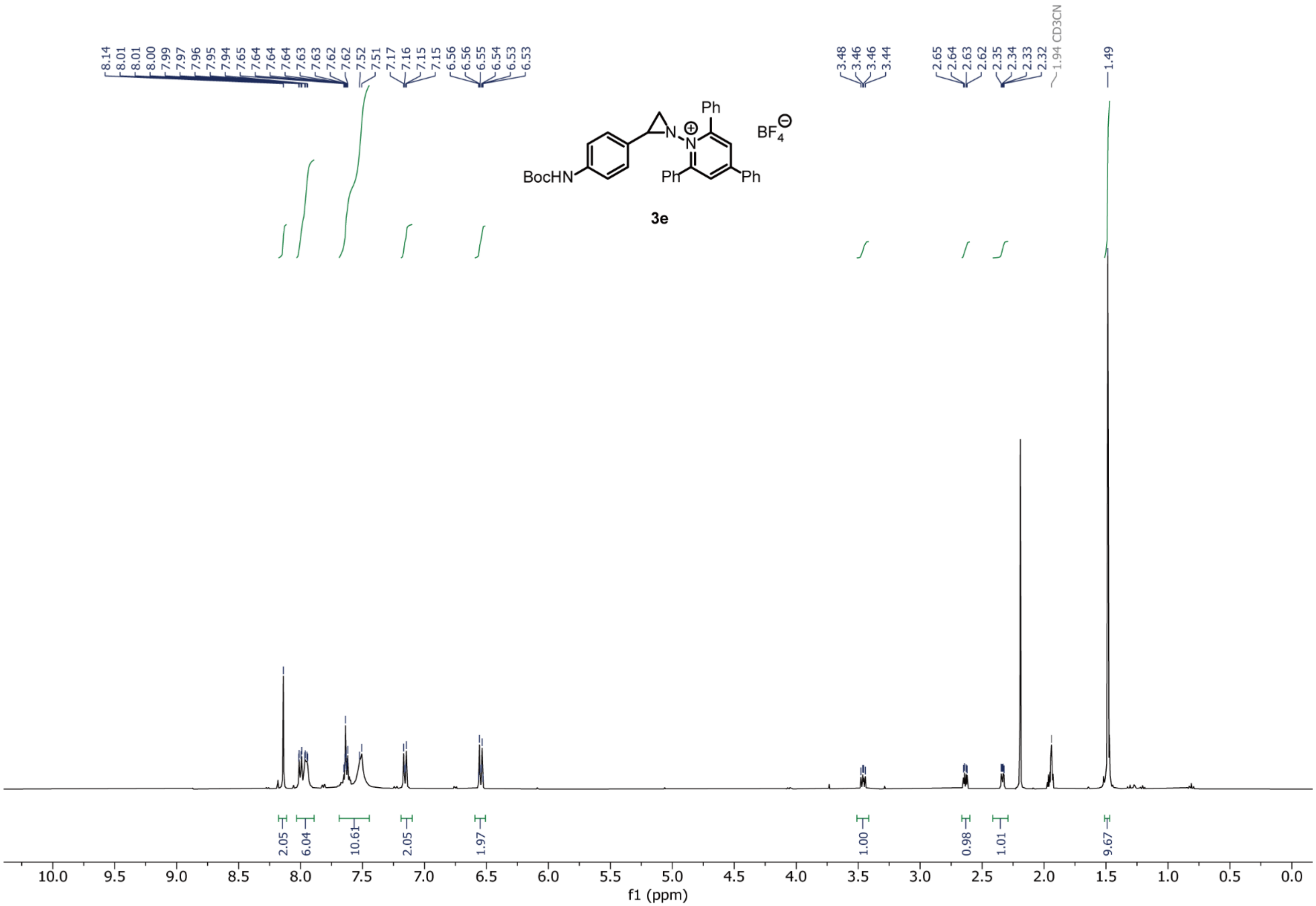

Supplementary Figure 16. ^{1}H NMR spectrum of 1-(2-(4-((tert-butoxycarbonyl)amino)phenyl)aziridin-1-yl)-2,4,6-triphenylpyridin-1-ium tetrafluoroborate (**3e**) in CD_3CN (400 MHz) at 23 °C.

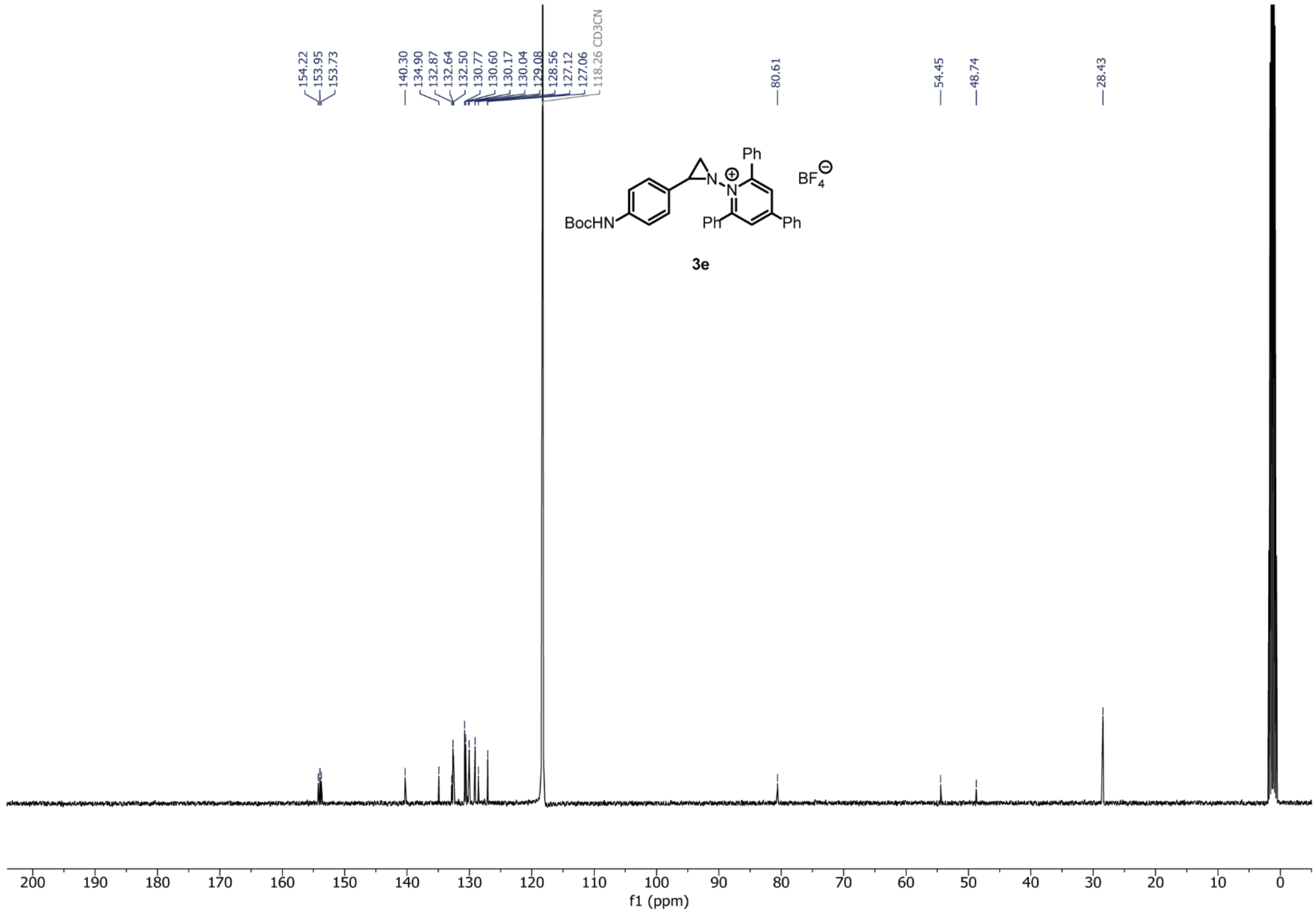

Supplementary Figure 17. ^{13}C NMR spectrum of 1-(2-(4-((tert-butoxycarbonyl)amino)phenyl)aziridin-1-yl)-2,4,6-triphenylpyridin-1-ium tetrafluoroborate (**3e**) in CD_3CN (101 MHz) at 23 °C.

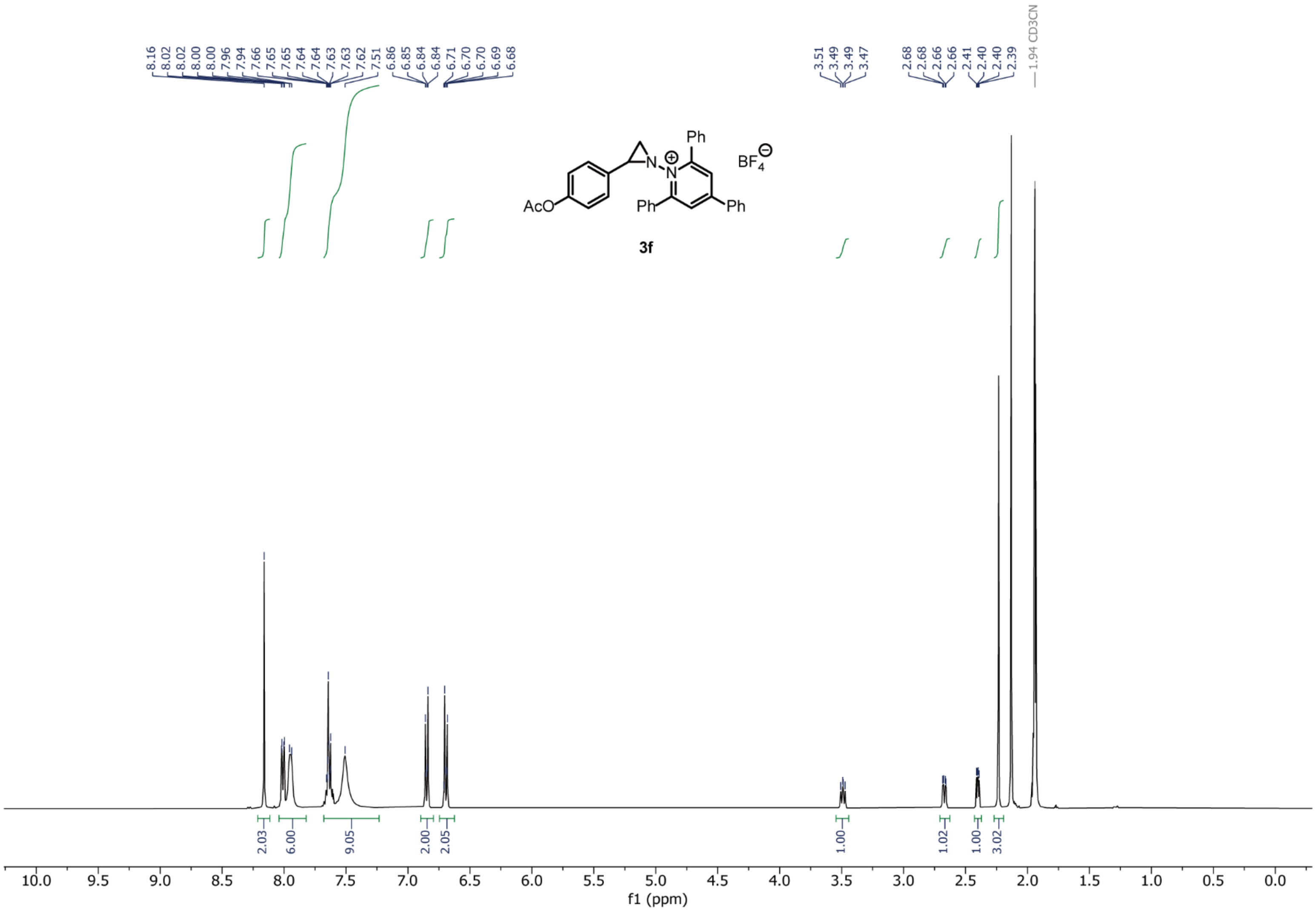

Supplementary Figure 18. ^{1}H NMR spectrum of 1-(2-(4-acetoxyphenyl)aziridin-1-yl)-2,4,6-triphenylpyridin-1-ium tetrafluoroborate (**3f**) in CD_3CN (400 MHz) at 23 °C.

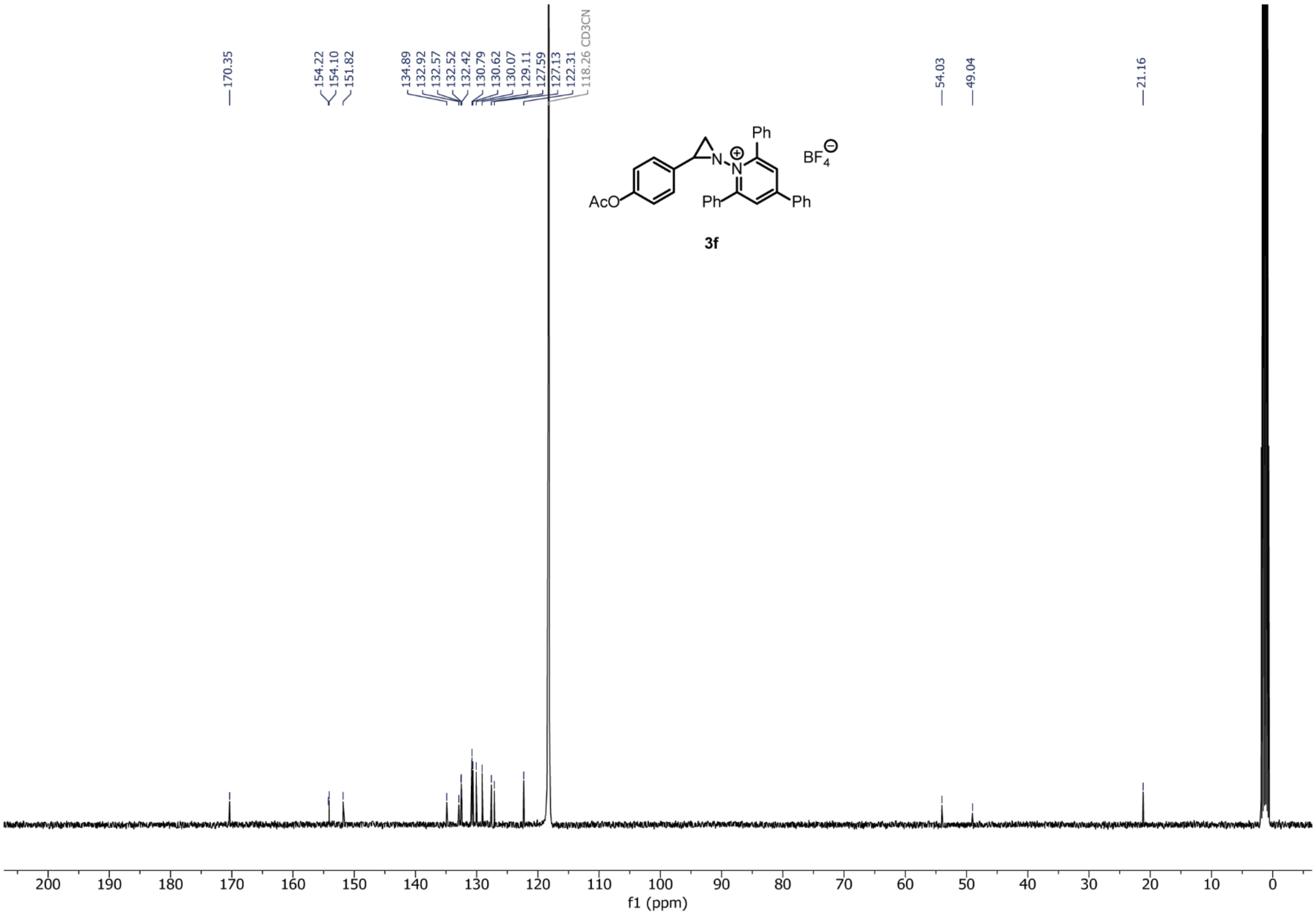

Supplementary Figure 19. ^{13}C NMR spectrum of 1-(2-(4-acetoxyphenyl)aziridin-1-yl)-2,4,6-triphenylpyridin-1-ium tetrafluoroborate (**3f**) in CD_3CN (101 MHz) at 23 °C.

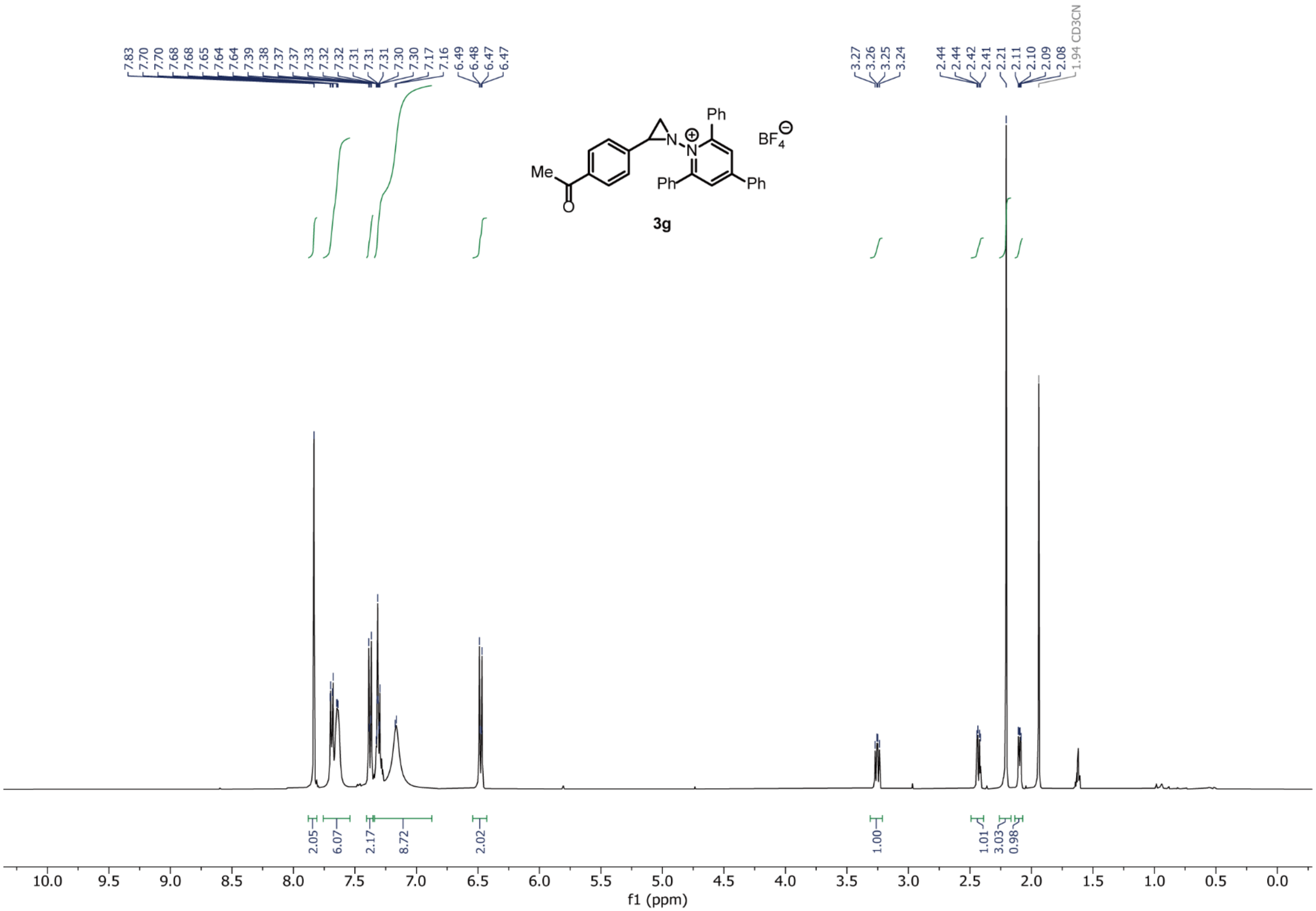

Supplementary Figure 20. ^{1}H NMR spectrum of 1-(2-(4-acetylphenyl)aziridin-1-yl)-2,4,6-triphenylpyridin-1-ium tetrafluoroborate (**3g**) in CD_3CN (400 MHz) at 23 °C.

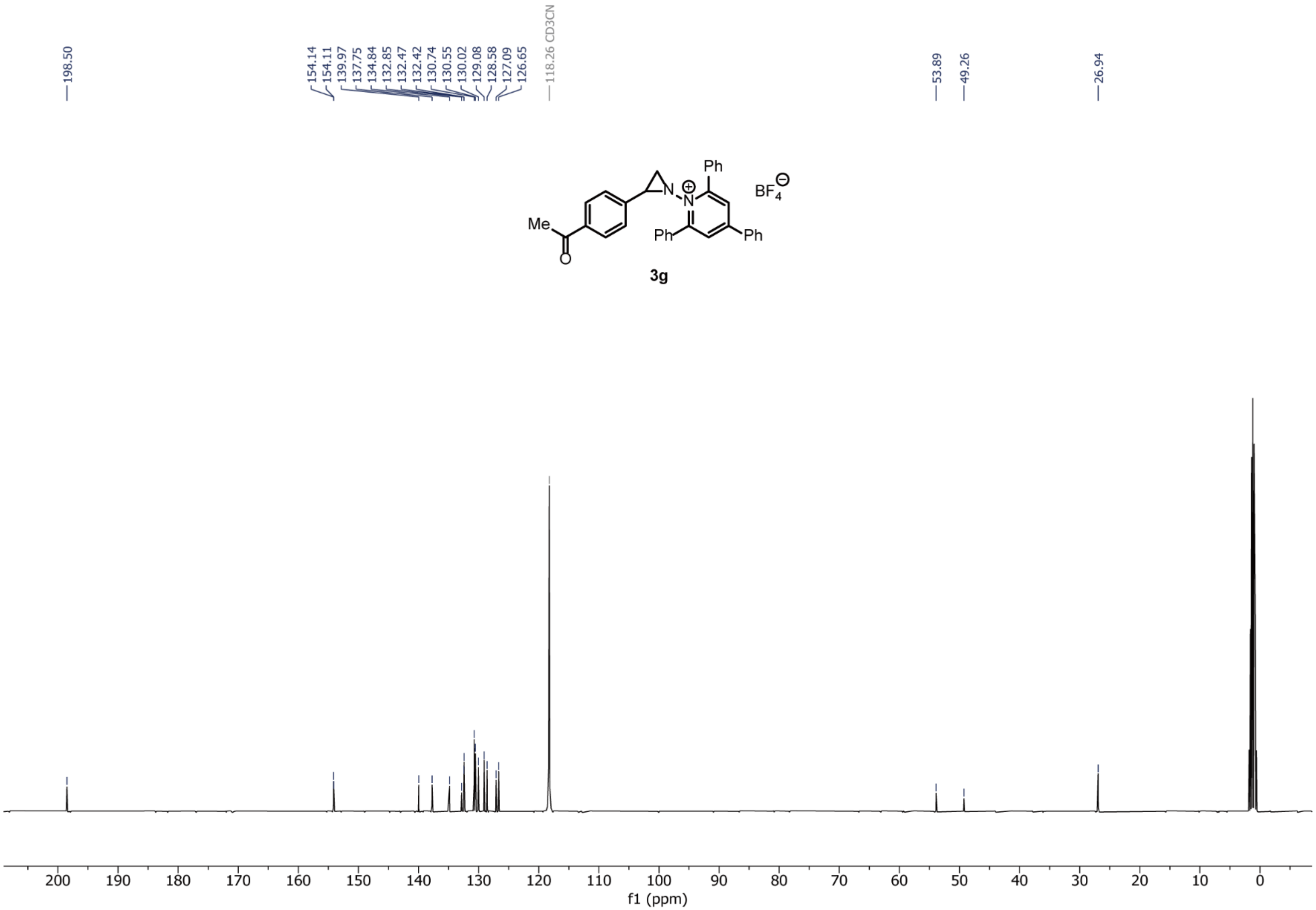

Supplementary Figure 21. ^{13}C NMR spectrum of 1-(2-(4-acetylphenyl)aziridin-1-yl)-2,4,6-triphenylpyridin-1-ium tetrafluoroborate (**3g**) in CD_3CN (101 MHz) at 23 °C.

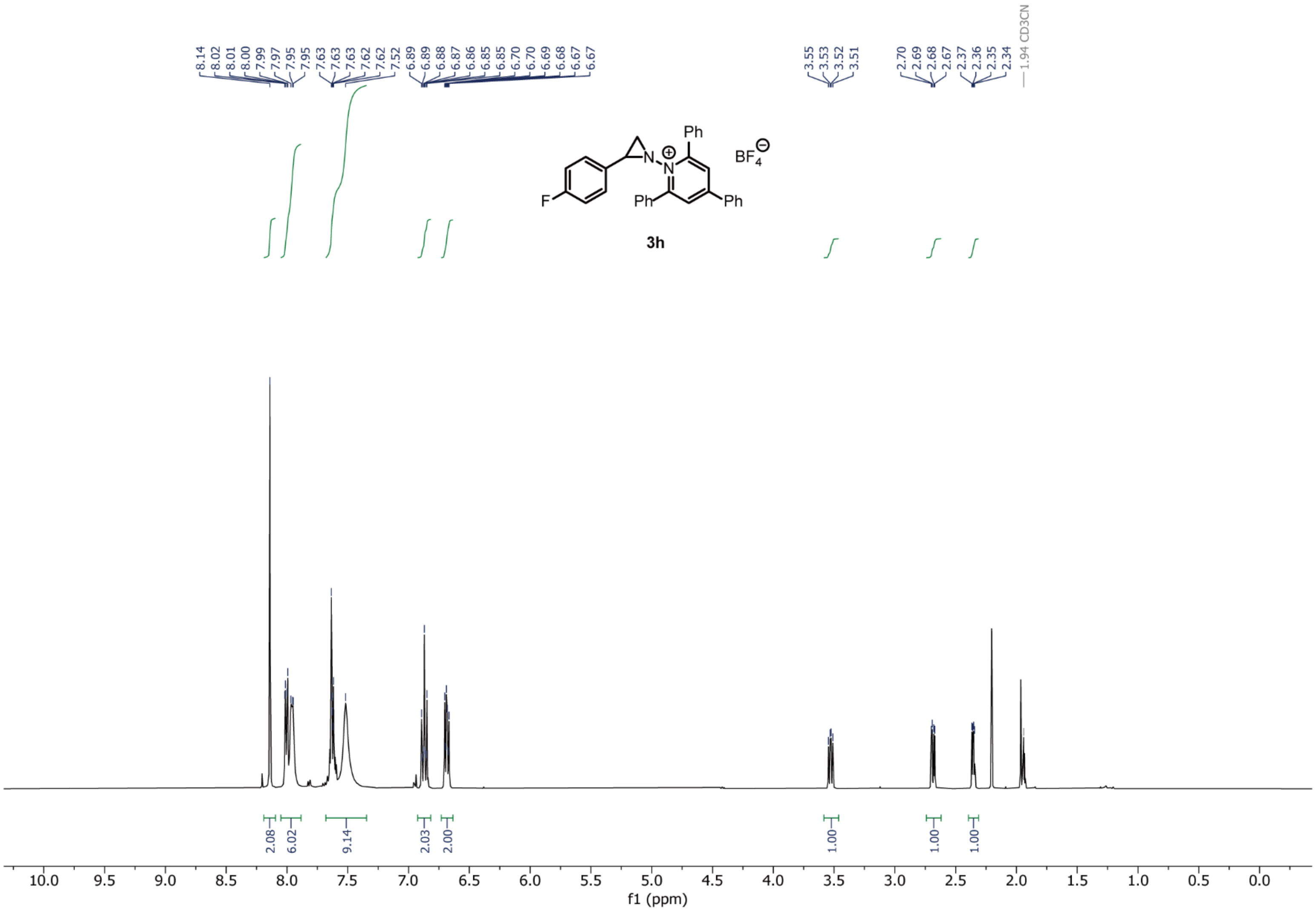

Supplementary Figure 22. ^{1}H NMR spectrum of 1-(2-(4-fluorophenyl)aziridin-1-yl)-2,4,6-triphenylpyridin-1-ium tetrafluoroborate (**3h**) in CD_3CN (400 MHz) at 23 °C.

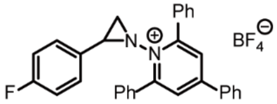

3h

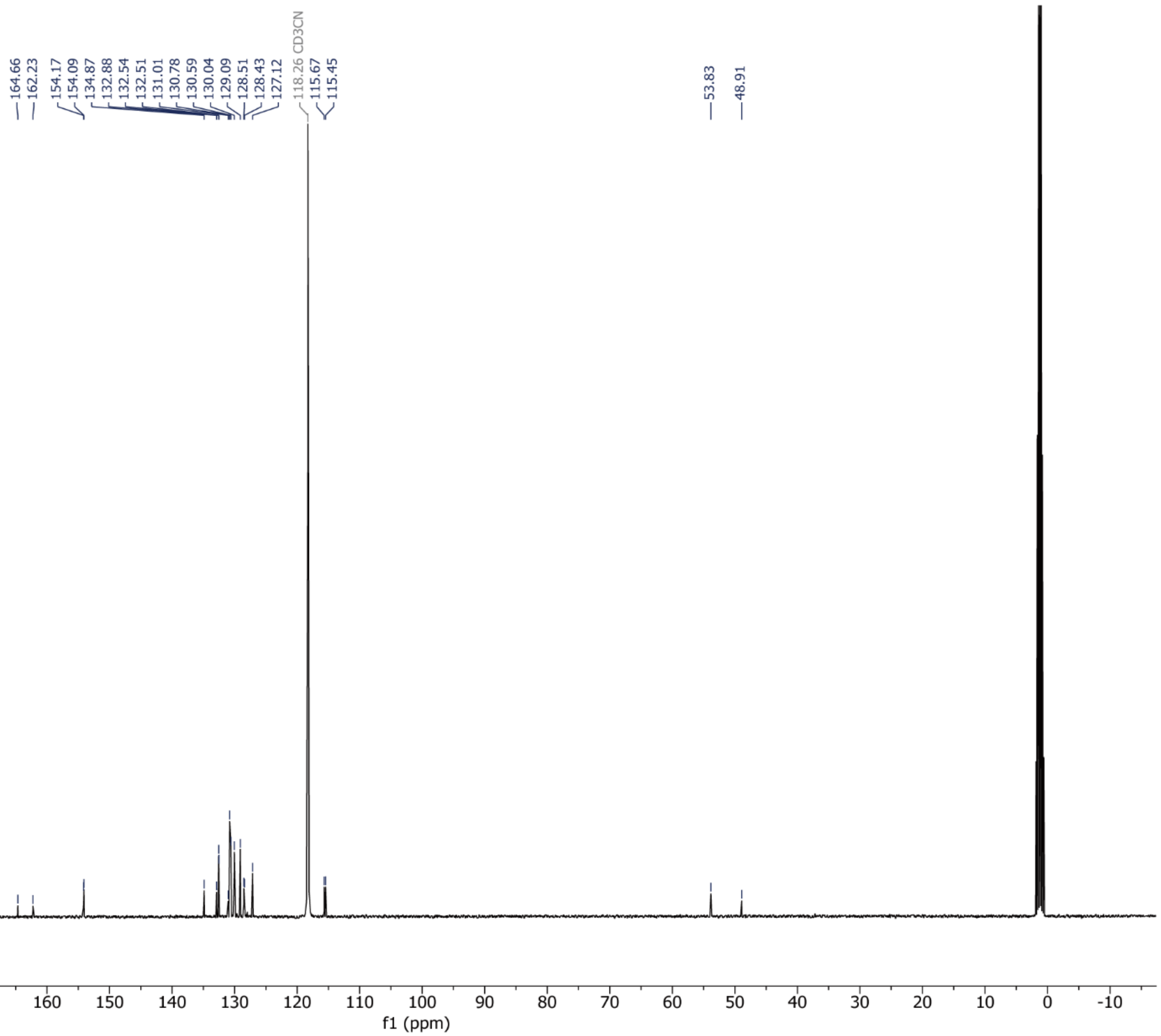

Supplementary Figure 23. ^{13}C NMR spectrum of 1-(2-(4-fluorophenyl)aziridin-1-yl)-2,4,6-triphenylpyridin-1-ium tetrafluoroborate (**3h**) in CD_3CN (101 MHz) at 23 °C.

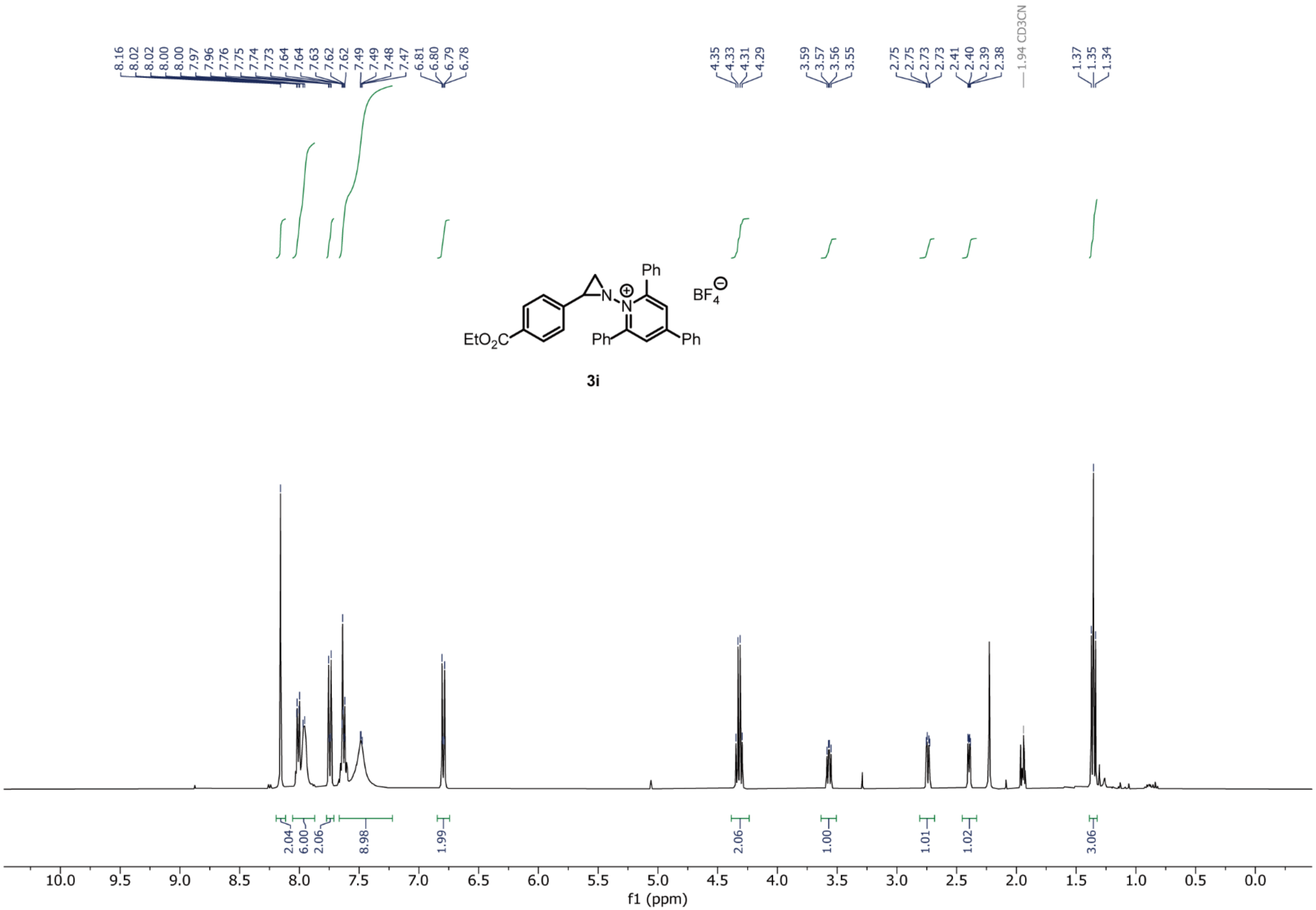

Supplementary Figure 24. ^{1}H NMR spectrum of 1-(2-(4-(ethoxycarbonyl)phenyl)aziridin-1-yl)-2,4,6-triphenylpyridin-1-ium tetrafluoroborate (**3i**) in CD_3CN (400 MHz) at 23 °C.

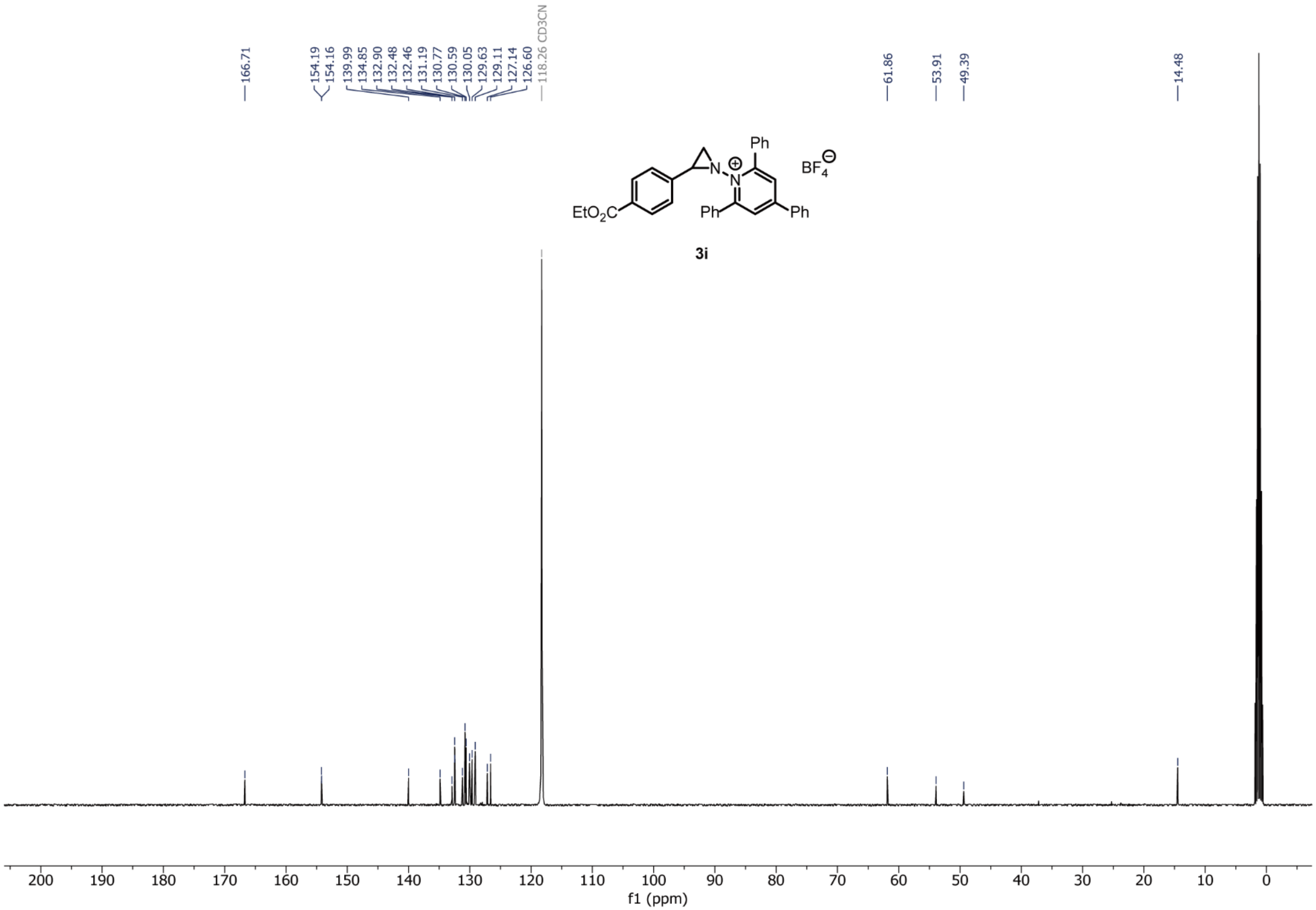

Supplementary Figure 25. ^{13}C NMR spectrum of 1-(2-(4-(ethoxycarbonyl)phenyl)aziridin-1-yl)-2,4,6-triphenylpyridin-1-ium tetrafluoroborate (**3i**) in CD_3CN (101 MHz) at 23 °C.

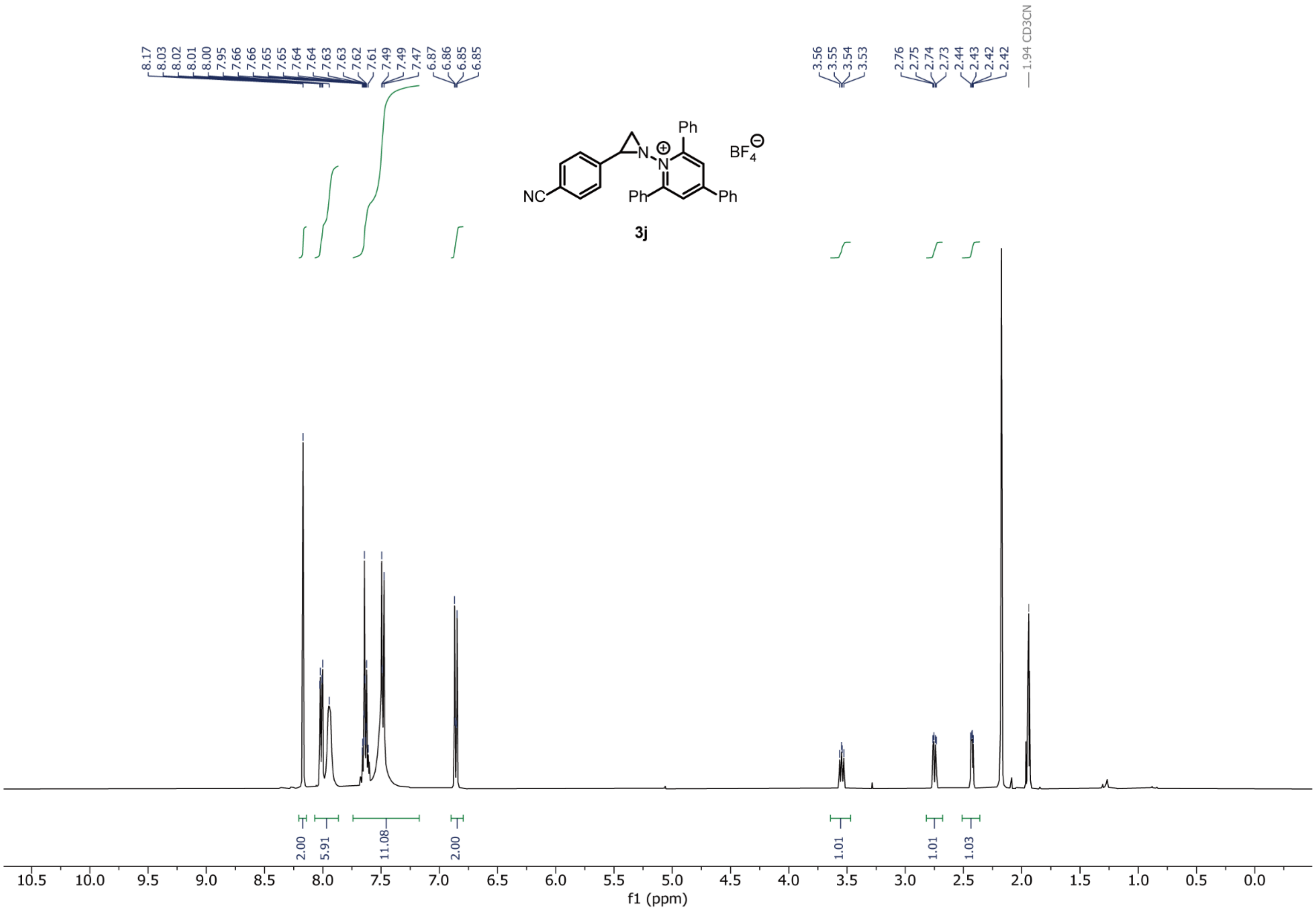

Supplementary Figure 26. ^{1}H NMR spectrum of 1-(2-(4-cyanophenyl)aziridin-1-yl)-2,4,6-triphenylpyridin-1-ium tetrafluoroborate (**3j**) in CD_3CN (400 MHz) at 23 °C.

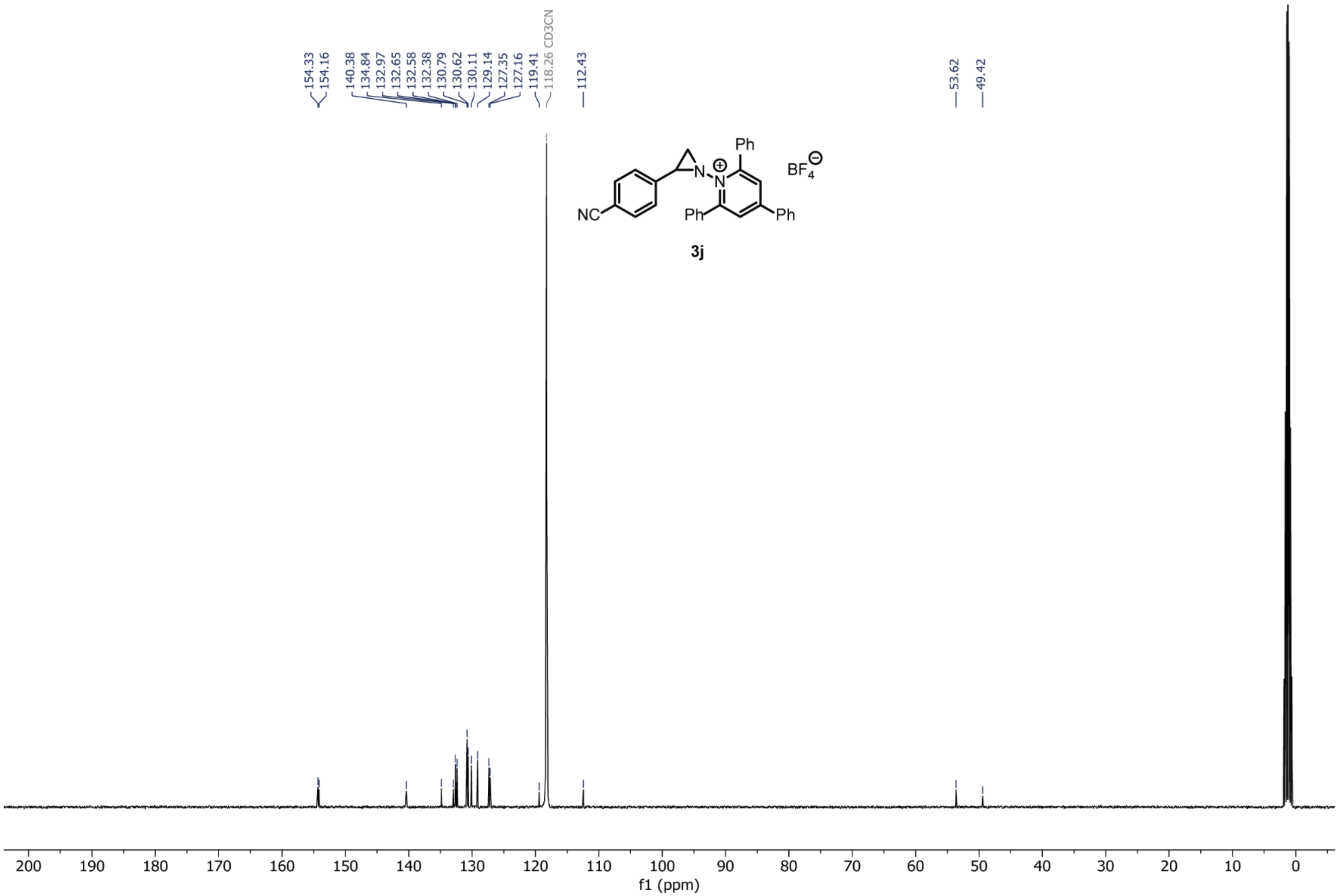

Supplementary Figure 27. ^{13}C NMR spectrum of 1-(2-(4-cyanophenyl)aziridin-1-yl)-2,4,6-triphenylpyridin-1-ium tetrafluoroborate (**3j**) in CD_3CN (101 MHz) at 23 °C.

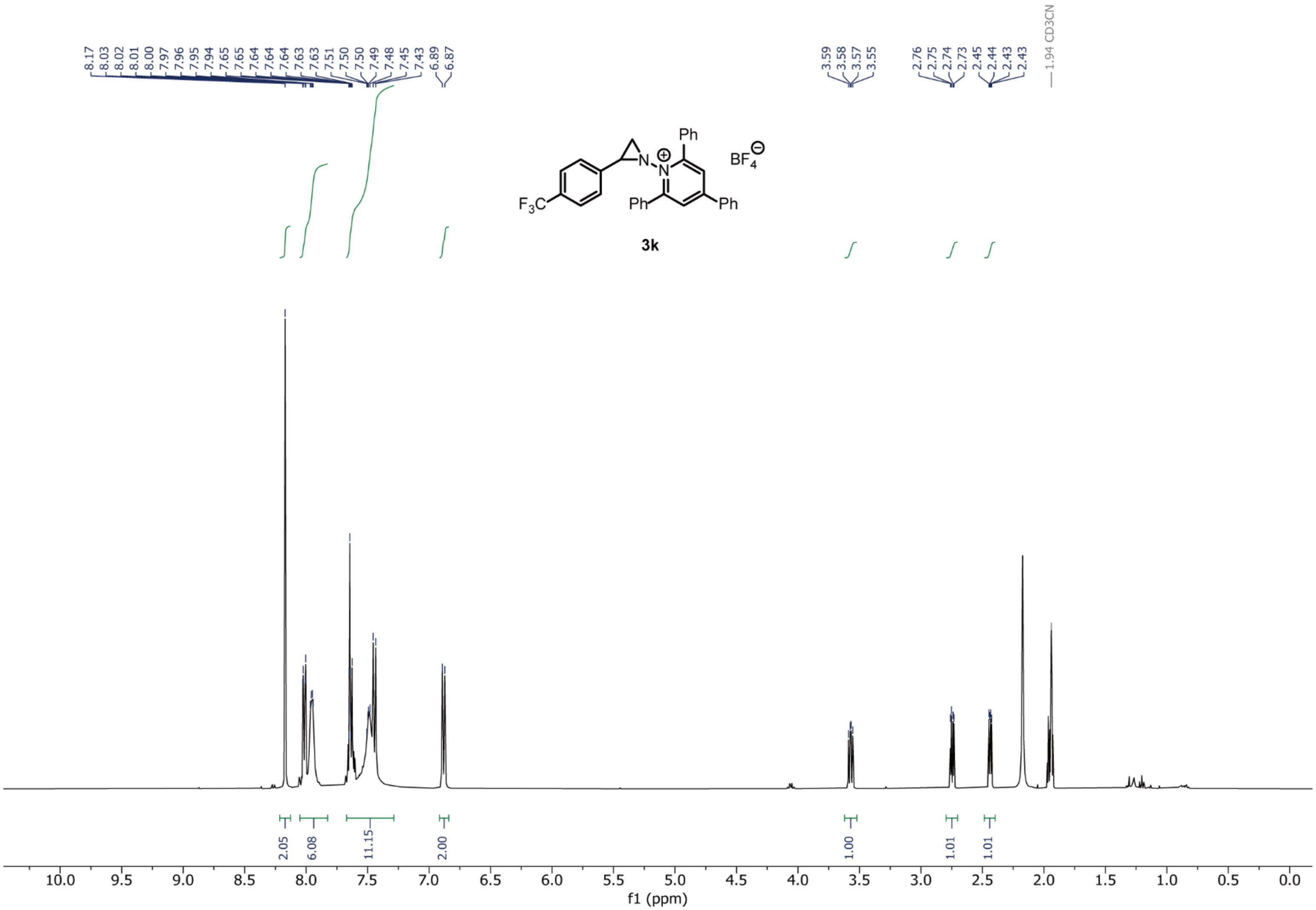

Supplementary Figure 28. ^{1}H NMR spectrum of 2,4,6-triphenyl-1-(2-(4-(trifluoromethyl)phenyl)aziridin-1-yl)pyridin-1-ium tetrafluoroborate (**3k**) in CD_3CN (400 MHz) at 23 °C.

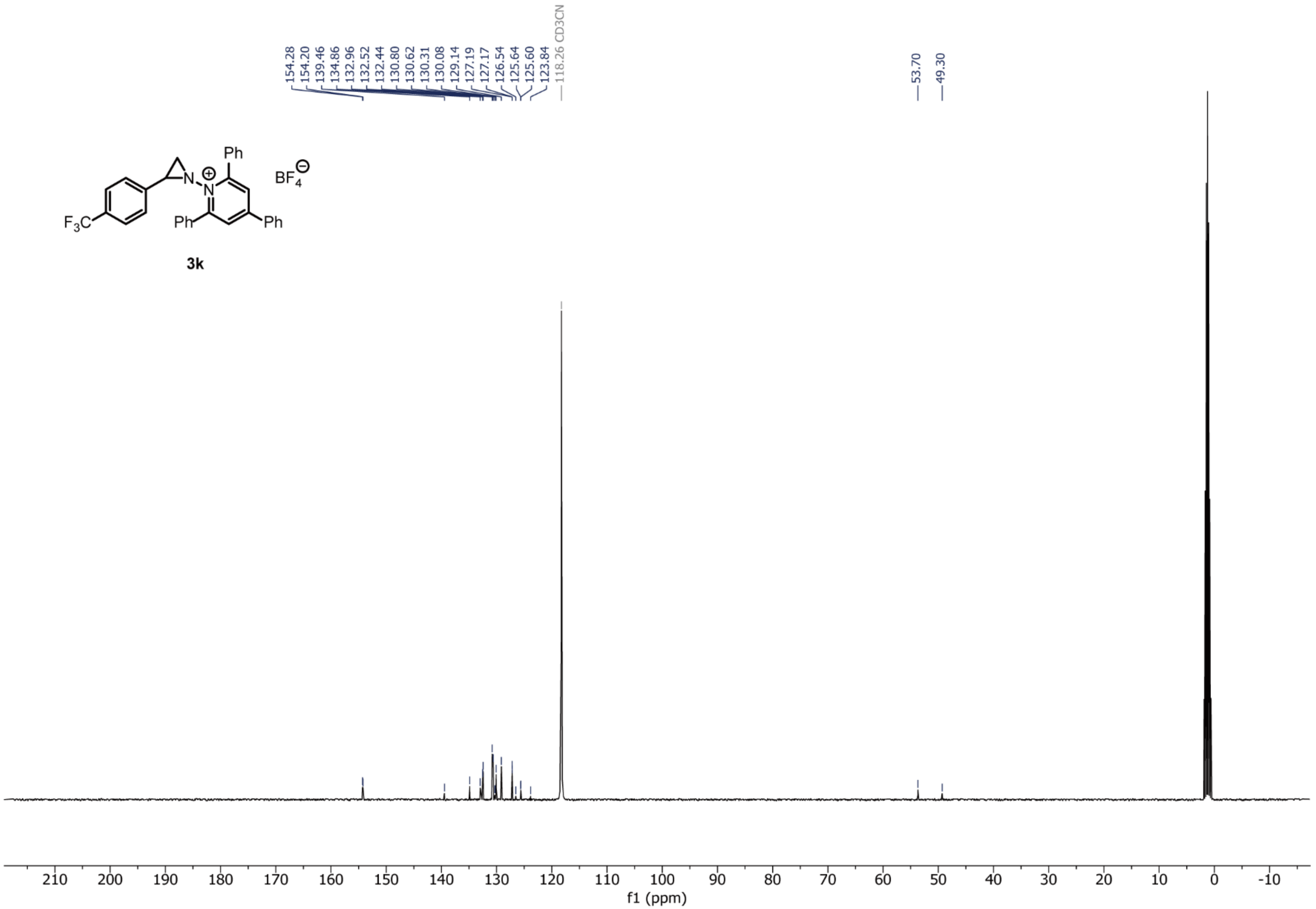

Supplementary Figure 29. ^{13}C NMR spectrum of 2,4,6-triphenyl-1-(2-(4-(trifluoromethyl)phenyl)aziridin-1-yl)pyridin-1-ium tetrafluoroborate (**3k**) in CD_3CN (101 MHz) at 23 °C.

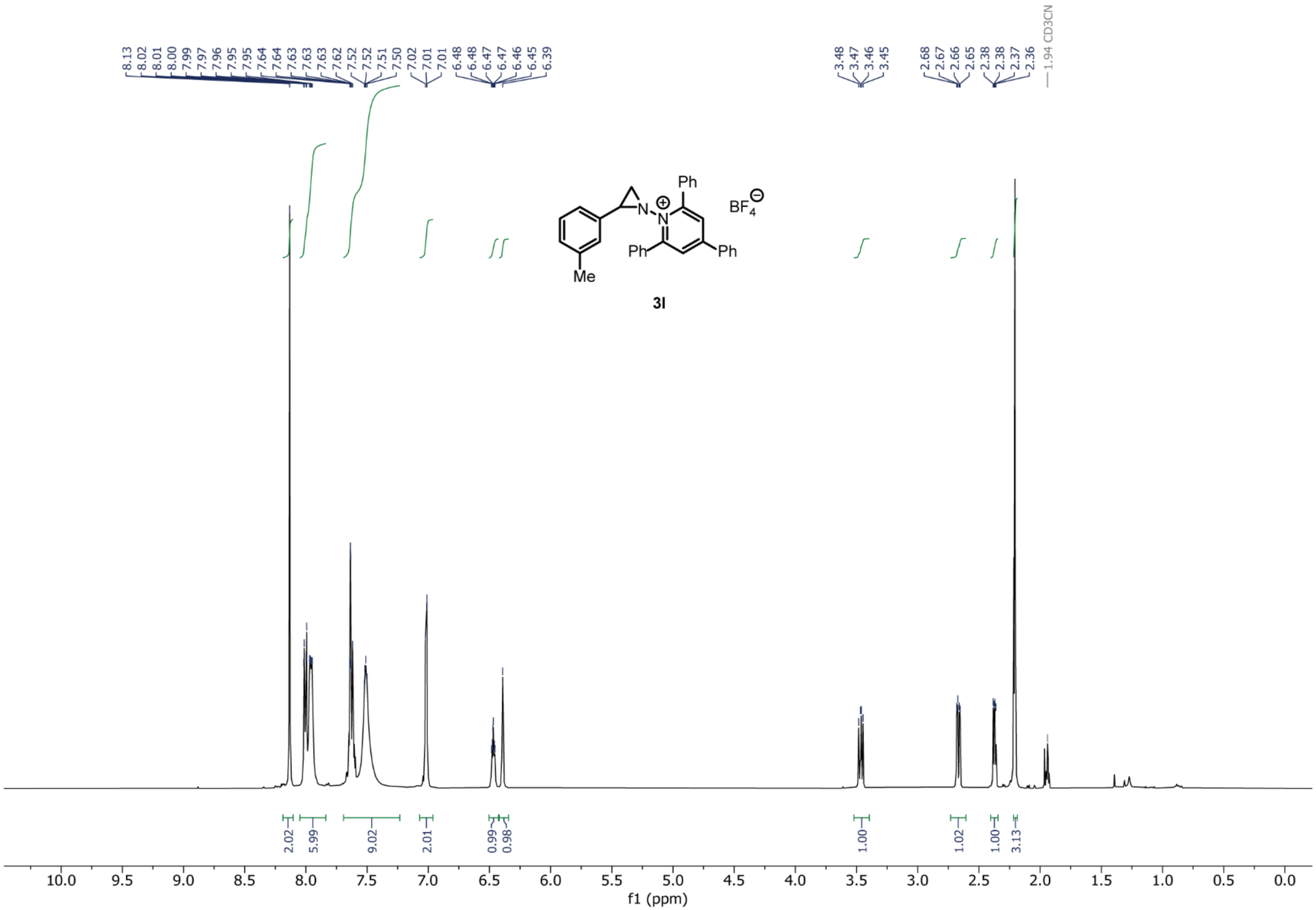

Supplementary Figure 30. ^{1}H NMR spectrum of 2,4,6-triphenyl-1-(2-(*m*-tolyl)aziridin-1-yl)pyridin-1-ium tetrafluoroborate (**3l**) in CD_3CN (400 MHz) at 23 °C.

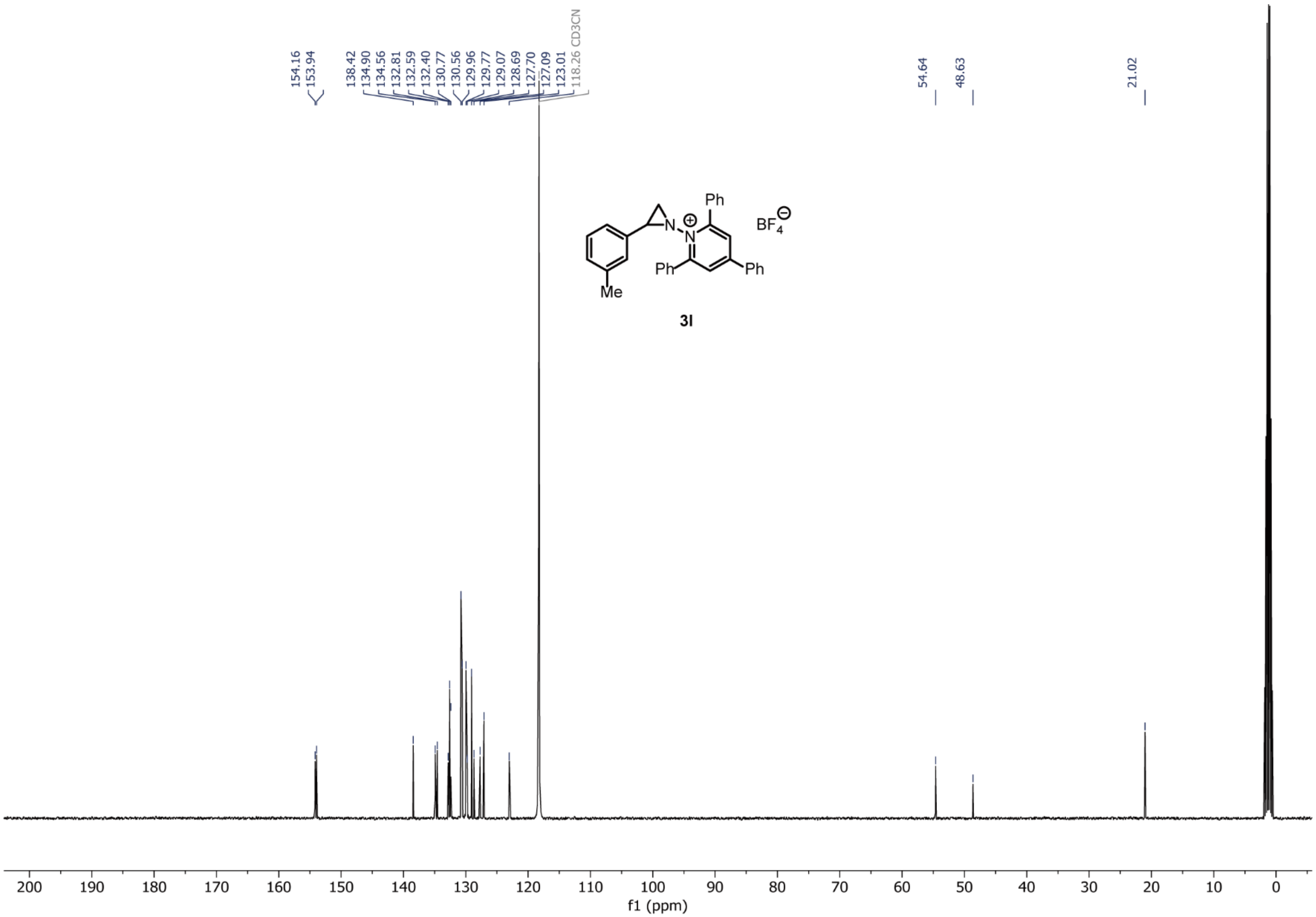

Supplementary Figure 31. ^{13}C NMR spectrum of 2,4,6-triphenyl-1-(2-(*m*-tolyl)aziridin-1-yl)pyridin-1-ium tetrafluoroborate (**3l**) in CD_3CN (101 MHz) at 23 °C.

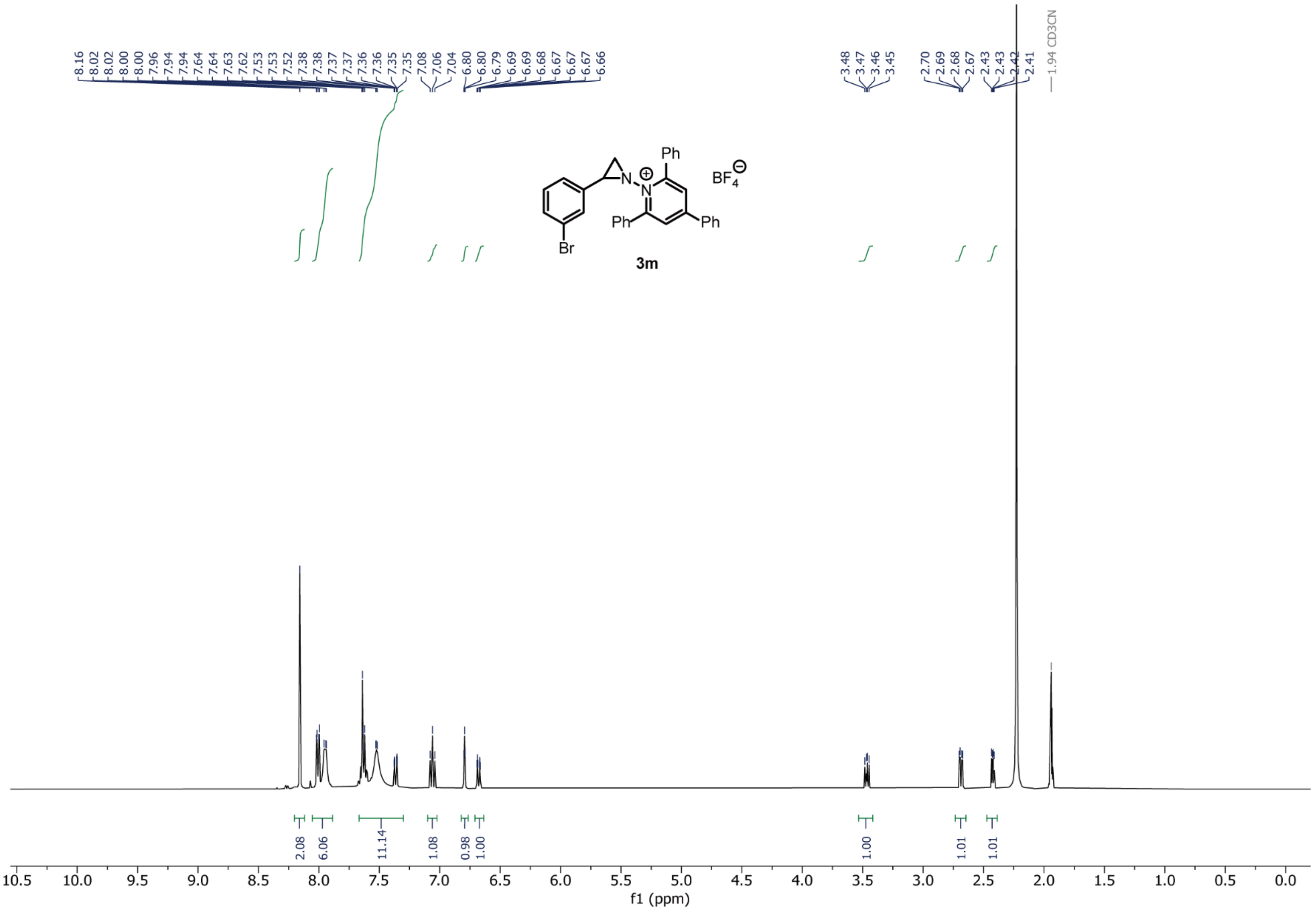

Supplementary Figure 32. ^{1}H NMR spectrum of 1-(2-(3-bromophenyl)aziridin-1-yl)-2,4,6-triphenylpyridin-1-ium tetrafluoroborate (**3m**) in CD_3CN (400 MHz) at 23 °C.

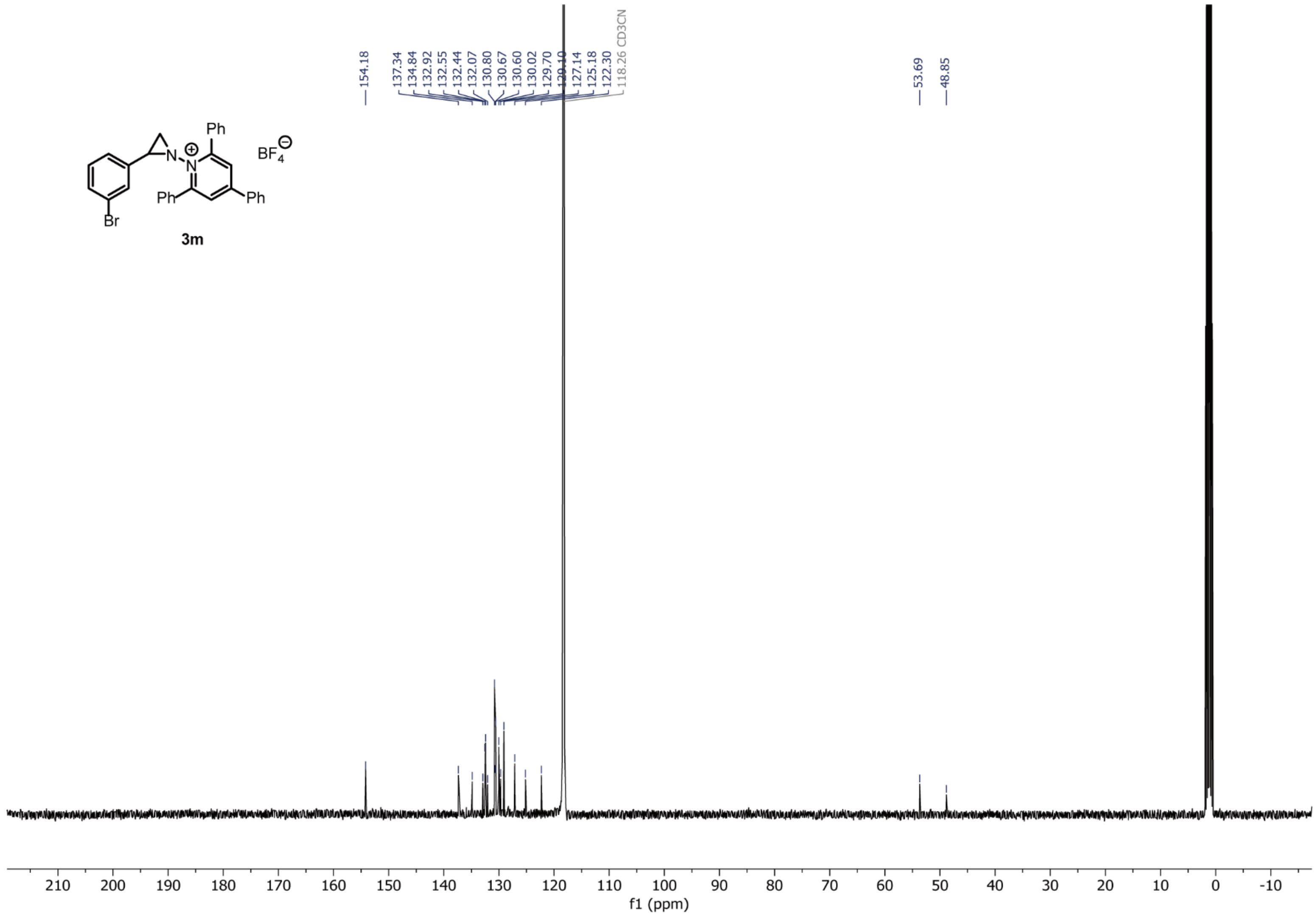

Supplementary Figure 33. ^{13}C NMR spectrum of 1-(2-(3-bromophenyl)aziridin-1-yl)-2,4,6-triphenylpyridin-1-ium tetrafluoroborate (**3m**) in CD_3CN (101 MHz) at 23 °C.

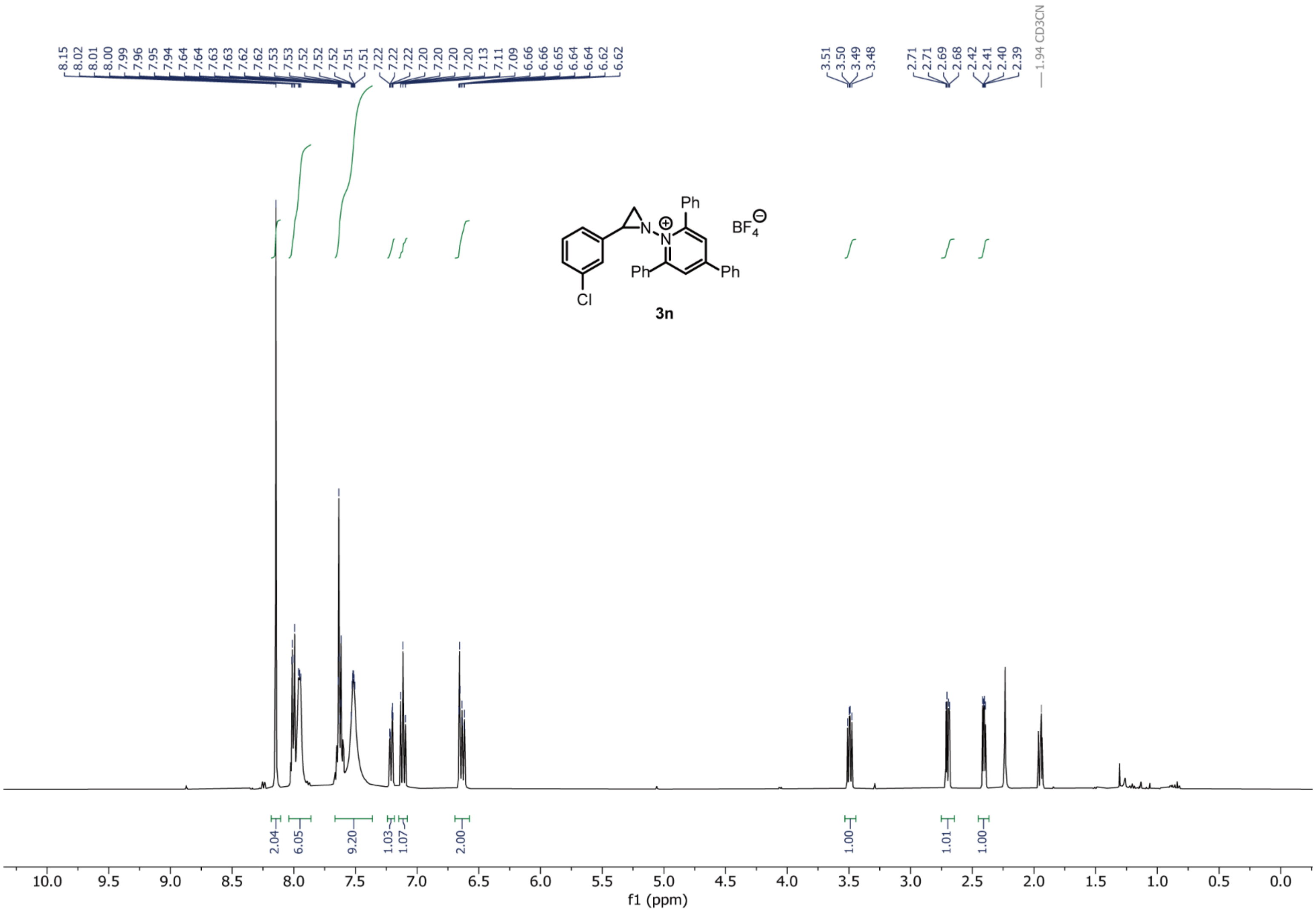

Supplementary Figure 34. ^{1}H NMR spectrum of 1-(2-(3-chlorophenyl)aziridin-1-yl)-2,4,6-triphenylpyridin-1-ium tetrafluoroborate (**3n**) in CD_3CN (400 MHz) at 23 °C.

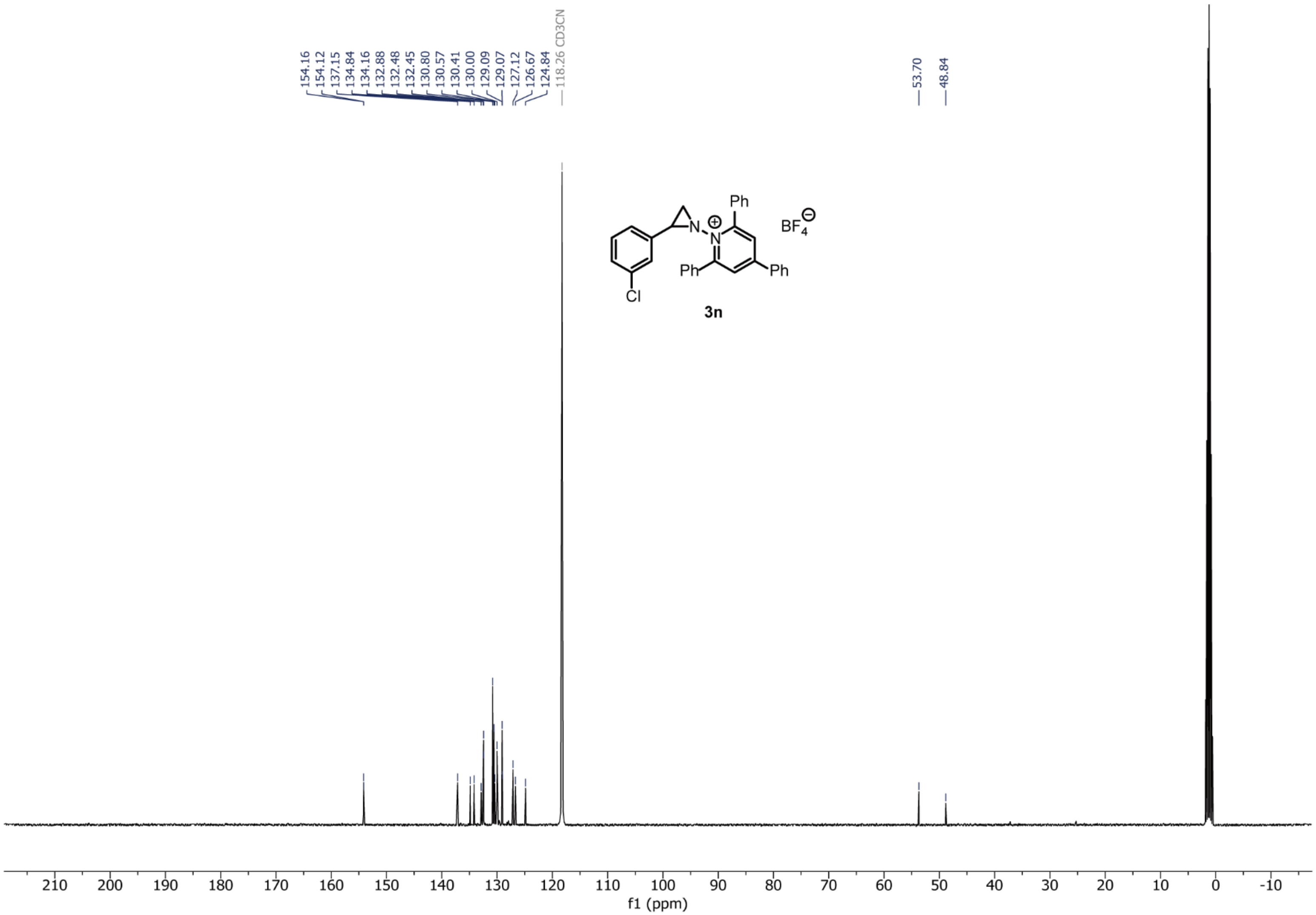

Supplementary Figure 35. ^{13}C NMR spectrum of 1-(2-(3-chlorophenyl)aziridin-1-yl)-2,4,6-triphenylpyridin-1-ium tetrafluoroborate (**3n**) in CD_3CN (101 MHz) at 23 °C.

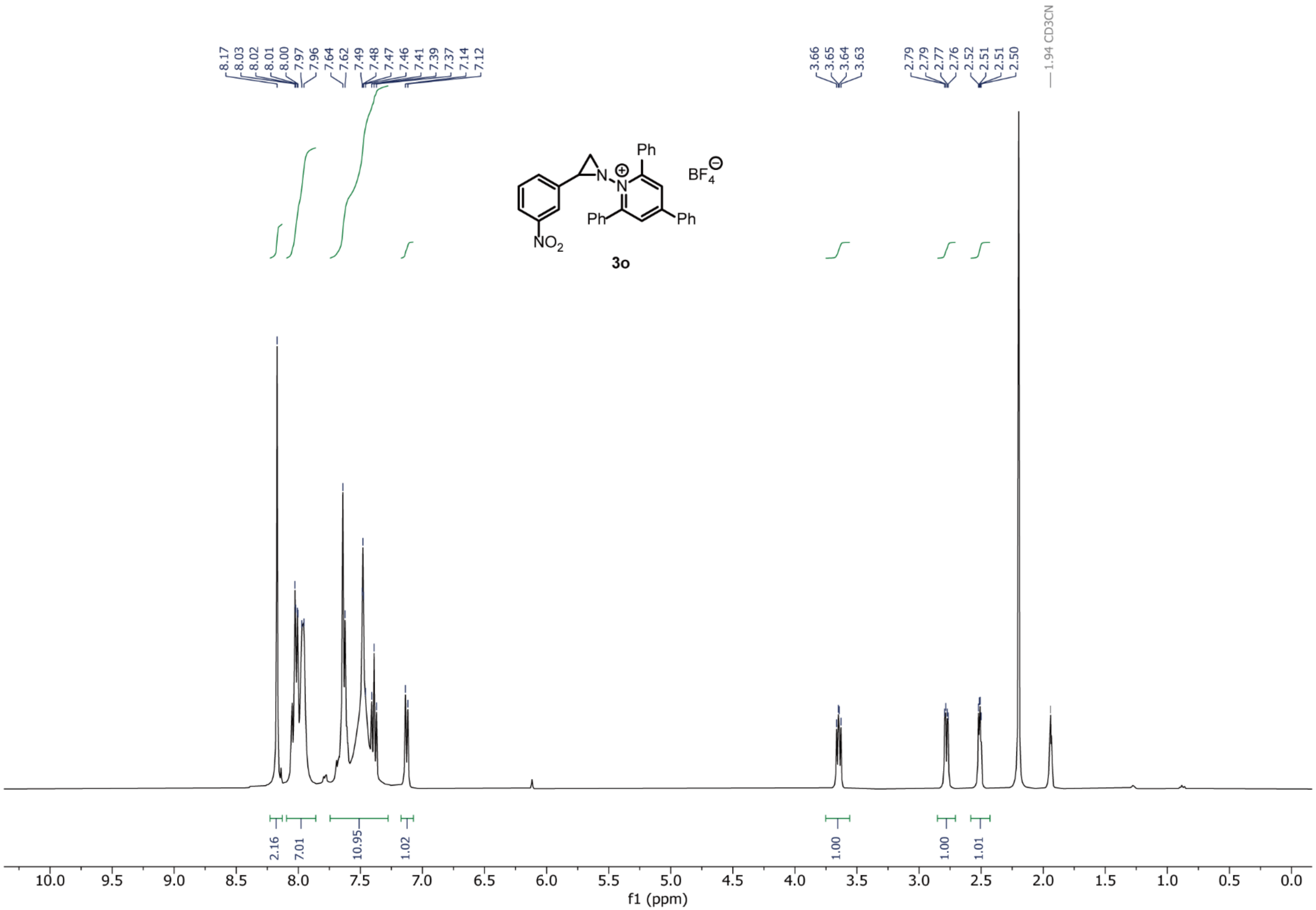

Supplementary Figure 36. ^{1}H NMR spectrum of 1-(2-(3-nitrophenyl)aziridin-1-yl)-2,4,6-triphenylpyridin-1-ium tetrafluoroborate (**3o**) in CD_3CN (400 MHz) at 23 °C.

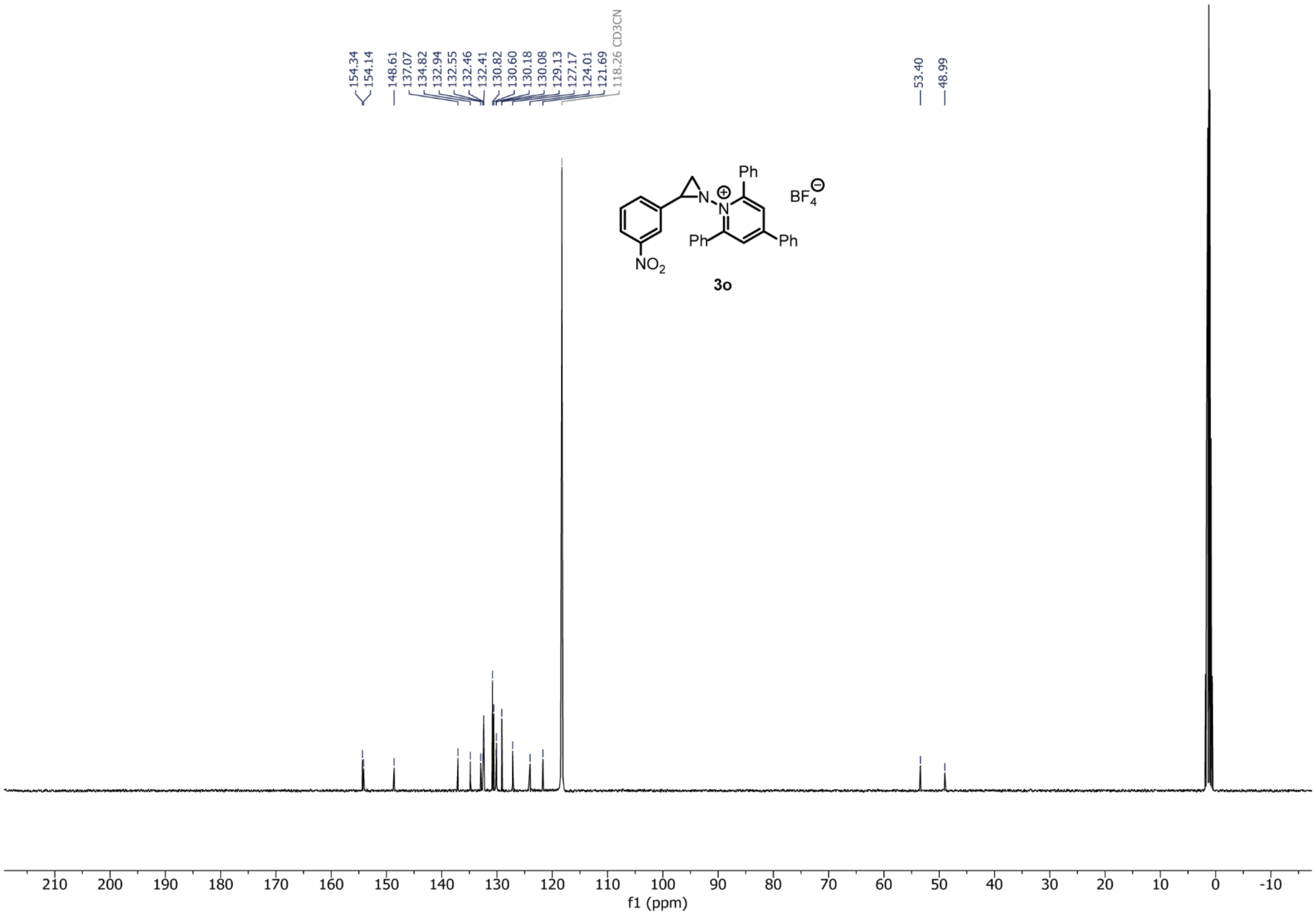

Supplementary Figure 37. ^{13}C NMR spectrum of 1-(2-(3-nitrophenyl)aziridin-1-yl)-2,4,6-triphenylpyridin-1-ium tetrafluoroborate (**3o**) in CD_3CN (101 MHz) at 23 °C.

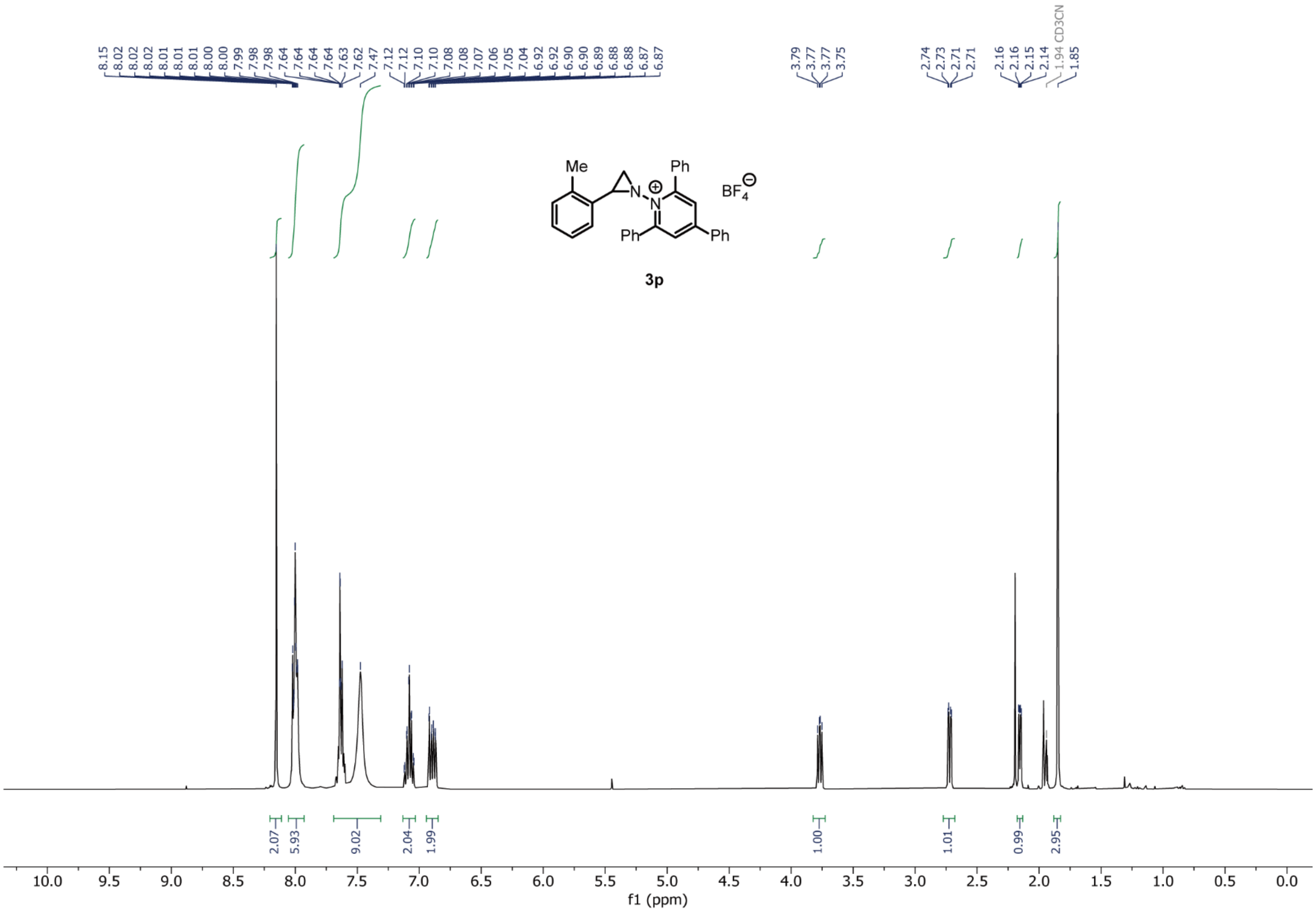

Supplementary Figure 38. ^{1}H NMR spectrum of 2,4,6-triphenyl-1-(2-(*o*-tolyl)aziridin-1-yl)pyridin-1-ium tetrafluoroborate (**3p**) in CD_3CN (400 MHz) at 23 °C.

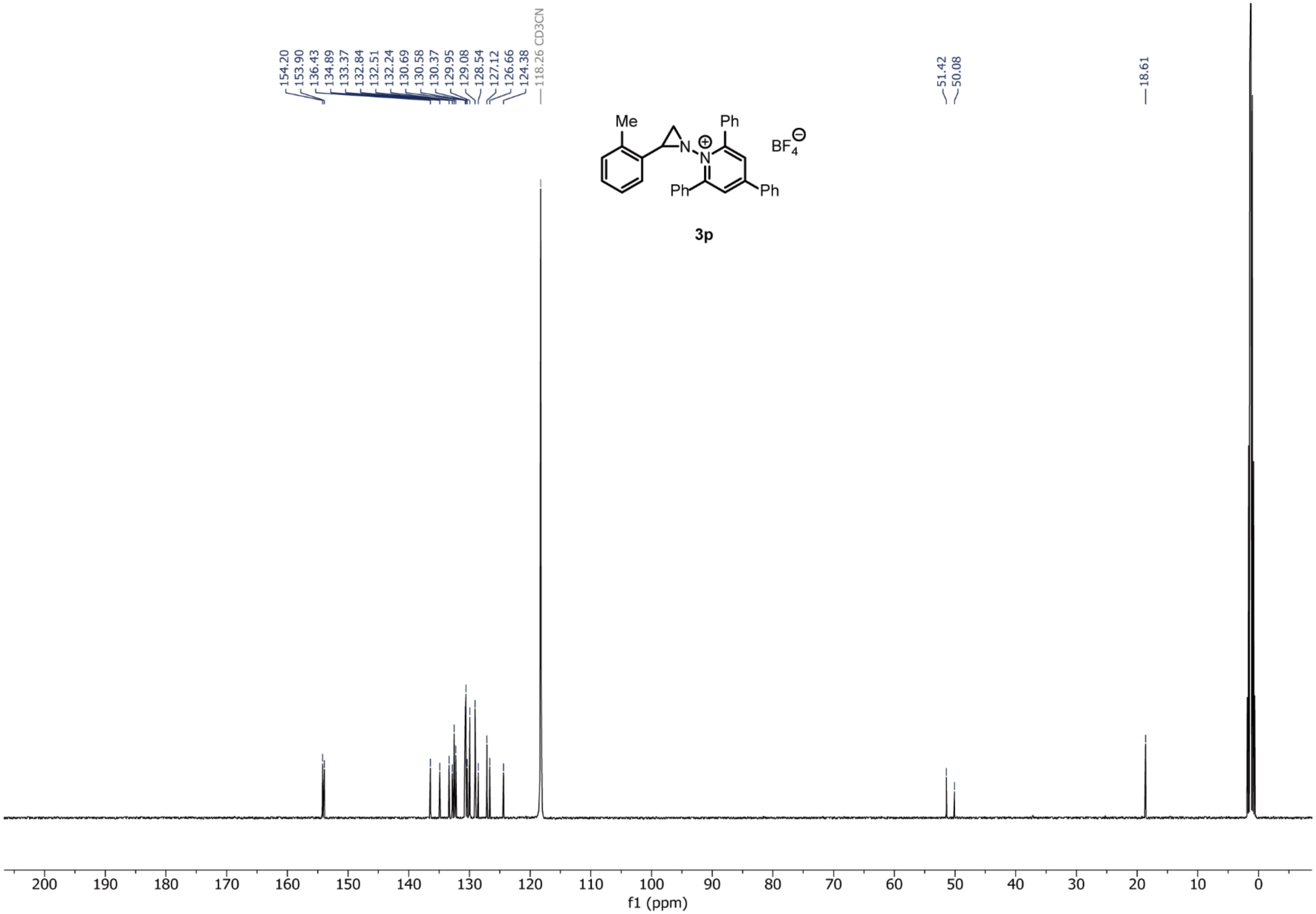

Supplementary Figure 39. ^{13}C NMR spectrum of 2,4,6-triphenyl-1-(2-(*o*-tolyl)aziridin-1-yl)pyridin-1-ium tetrafluoroborate (**3p**) in CD_3CN (101 MHz) at 23 °C.

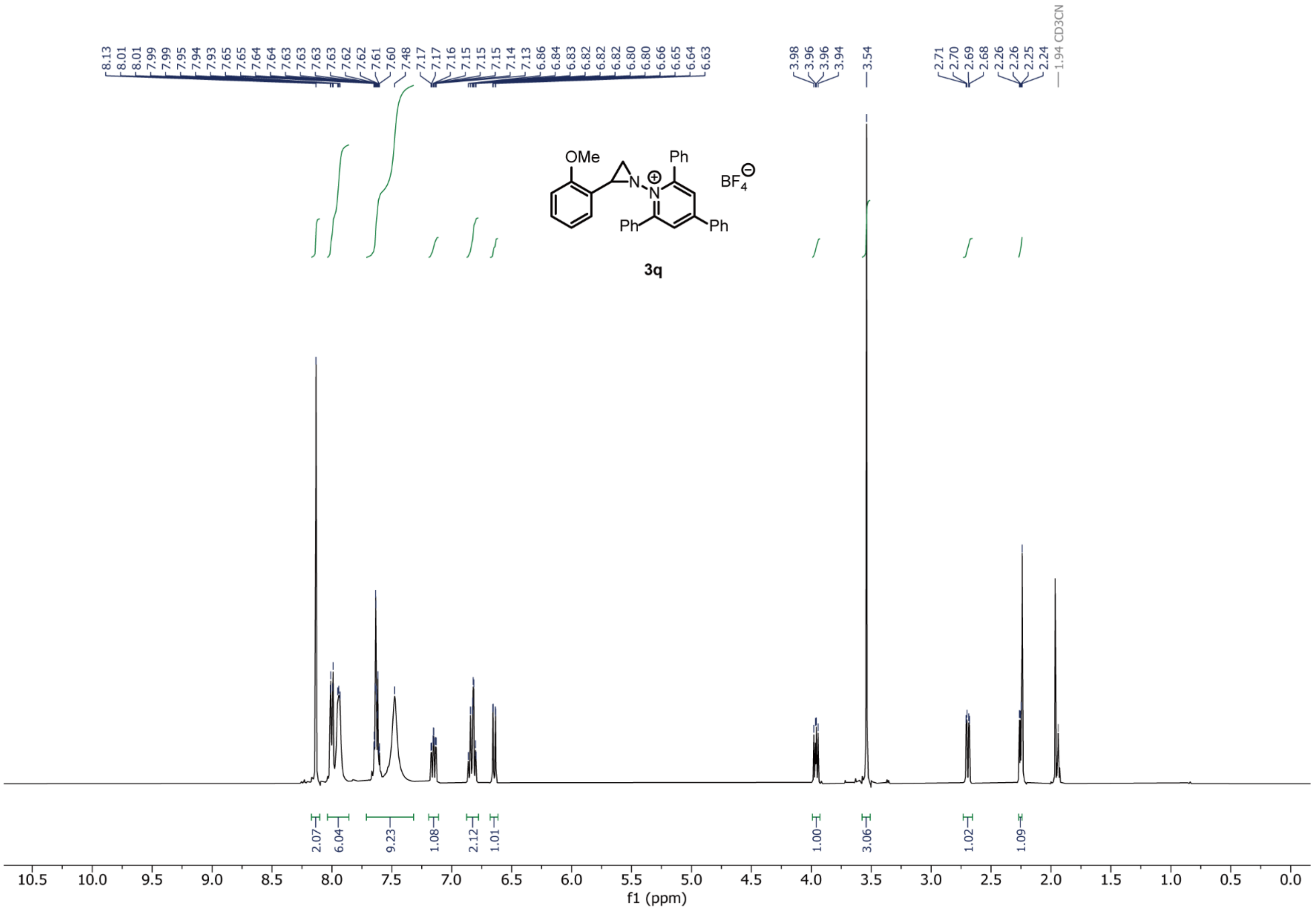

Supplementary Figure 40. ^{1}H NMR spectrum of 1-(2-(2-methoxyphenyl)aziridin-1-yl)-2,4,6-triphenylpyridin-1-ium tetrafluoroborate (**3q**) in CD_3CN (400 MHz) at 23 °C.

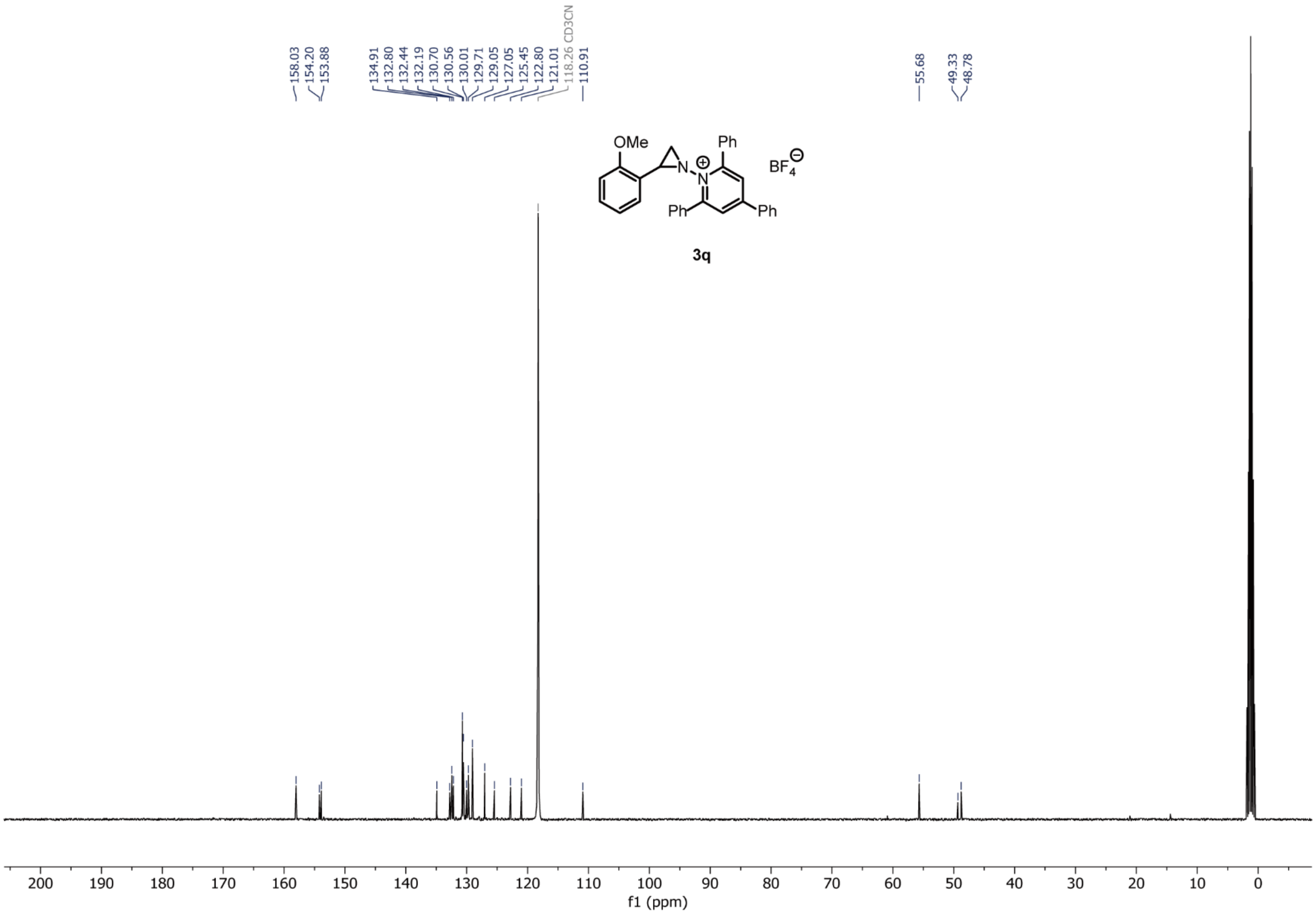

Supplementary Figure 41. ^{13}C NMR spectrum of 1-(2-(2-methoxyphenyl)aziridin-1-yl)-2,4,6-triphenylpyridin-1-ium tetrafluoroborate (**3q**) in CD_3CN (101 MHz) at 23 °C.

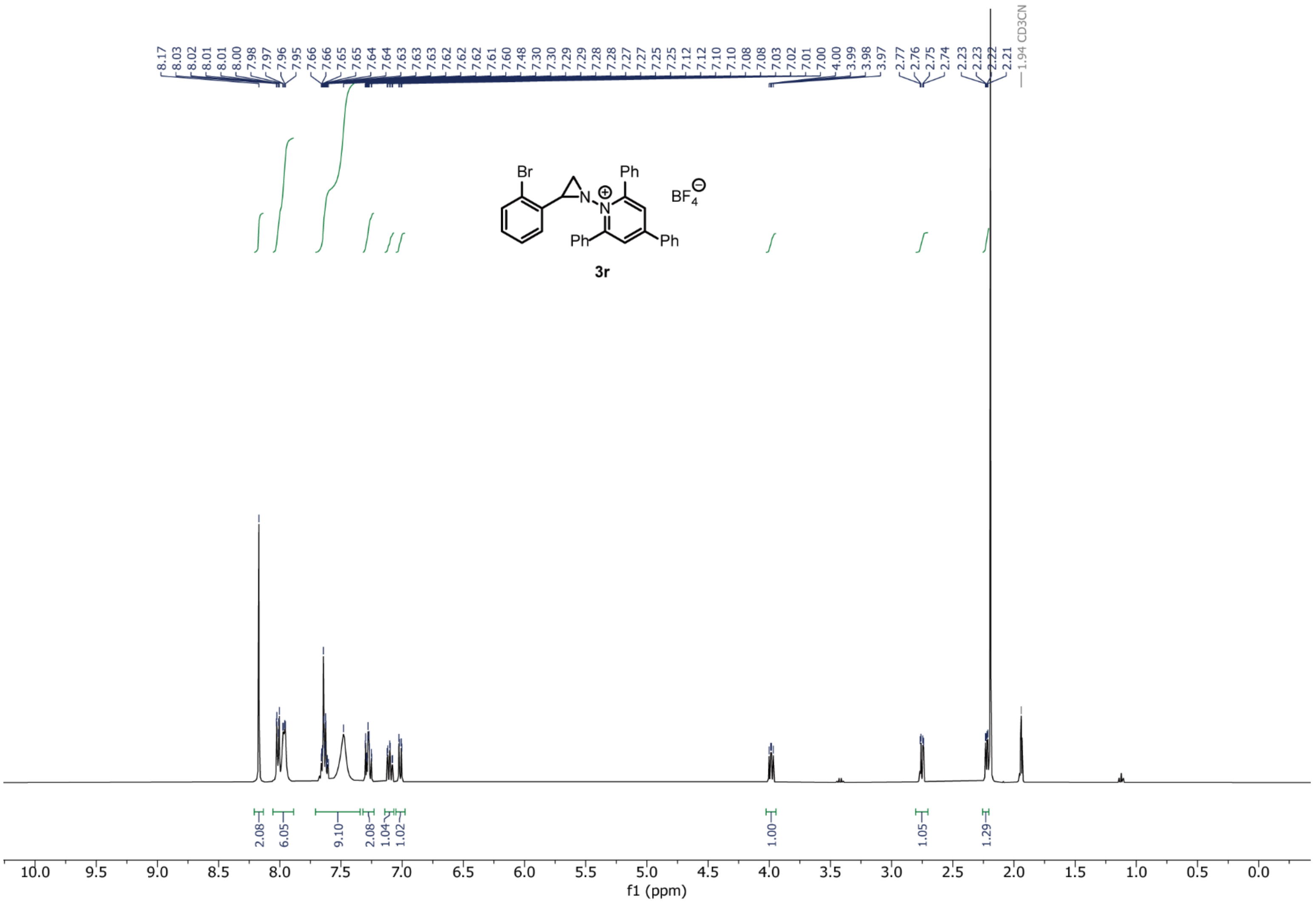

Supplementary Figure 42. ^{1}H NMR spectrum of 1-(2-(2-bromophenyl)aziridin-1-yl)-2,4,6-triphenylpyridin-1-ium tetrafluoroborate (**3r**) in CD_3CN (400 MHz) at 23 °C.

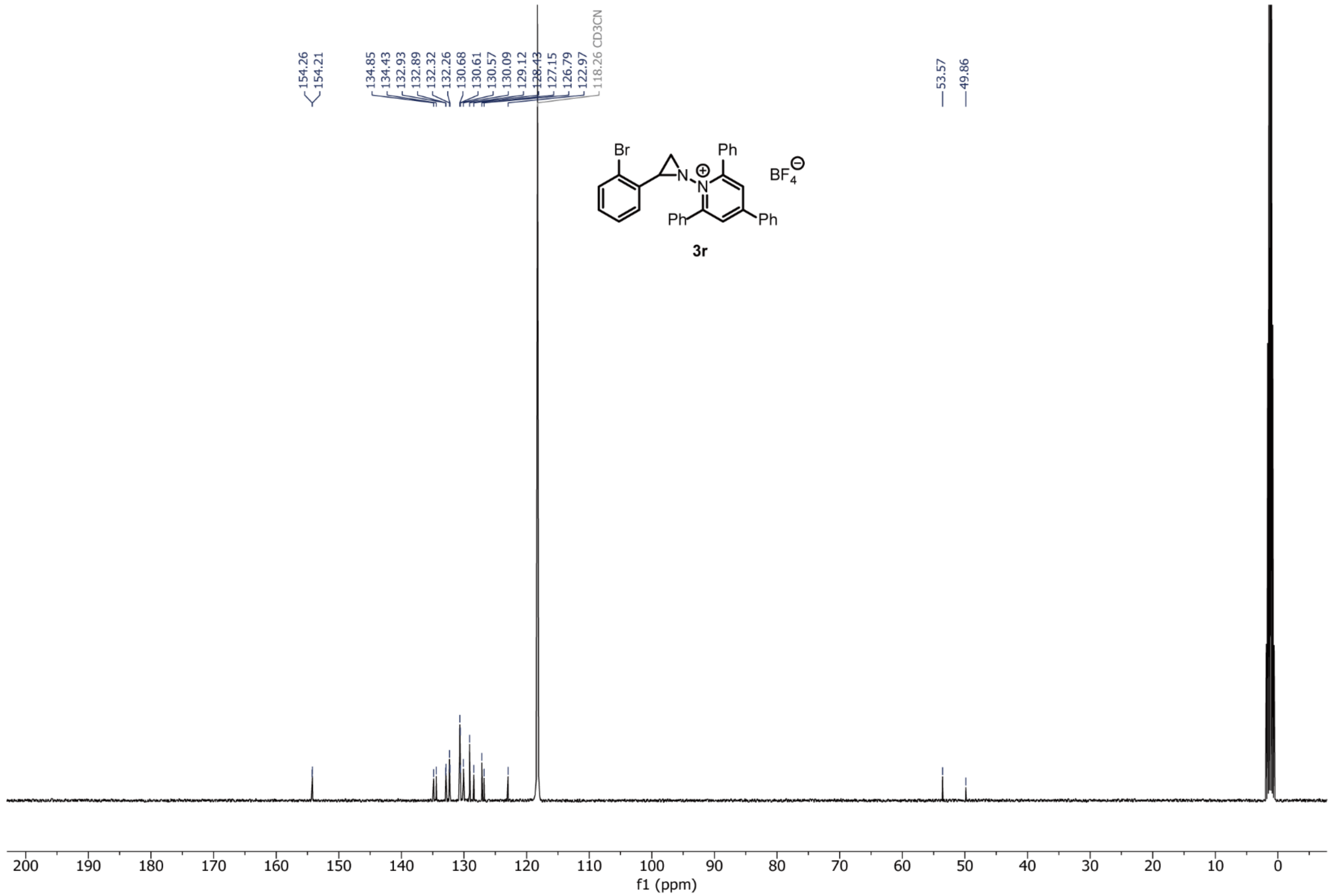

Supplementary Figure 43. ^{13}C NMR spectrum of 1-(2-(2-methoxyphenyl)aziridin-1-yl)-2,4,6-triphenylpyridin-1-ium tetrafluoroborate (**3r**) in CD_3CN (101 MHz) at 23 °C.

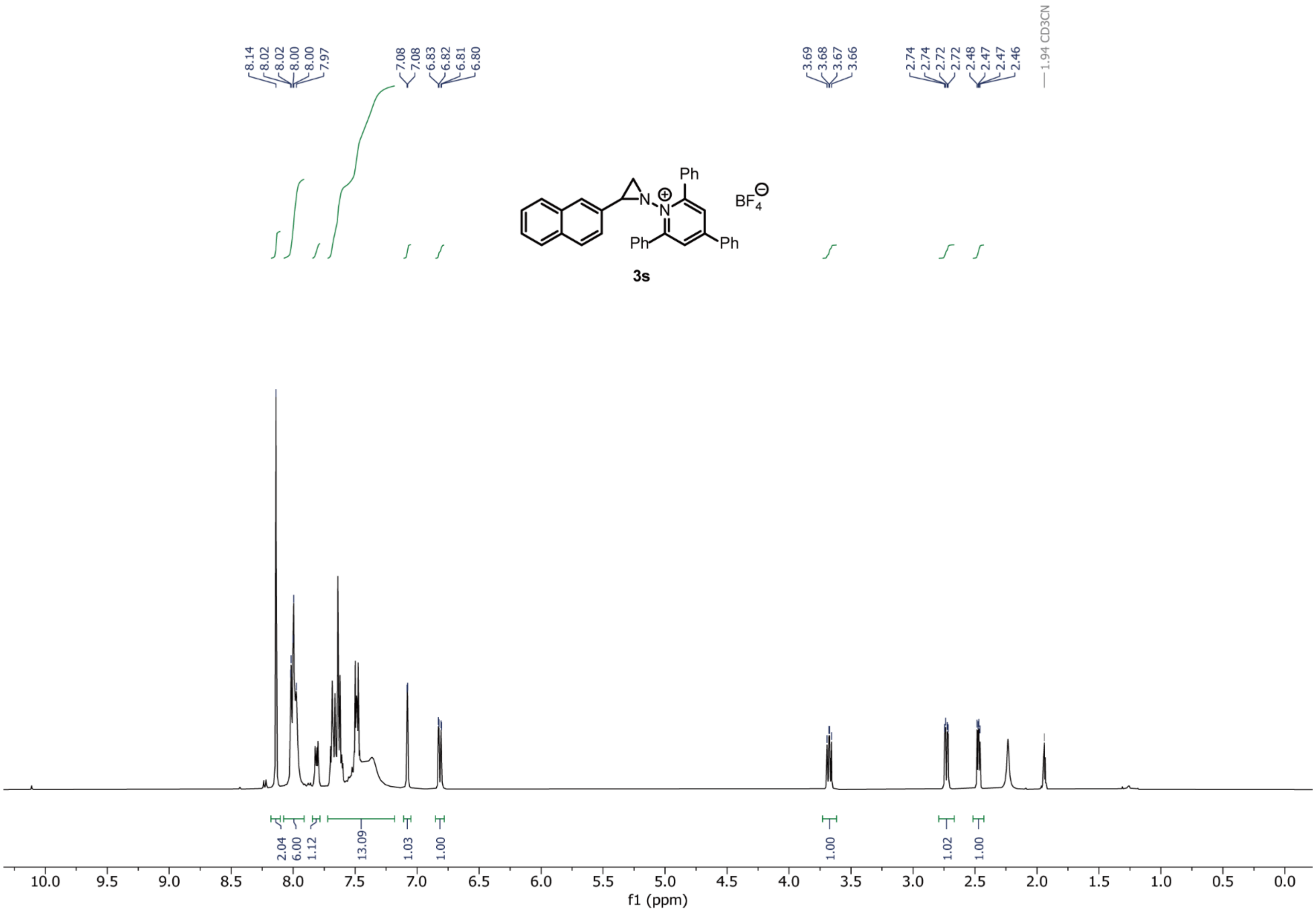

Supplementary Figure 44. ^{1}H NMR spectrum of 1-(2-(naphthalen-2-yl)aziridin-1-yl)-2,4,6-triphenylpyridin-1-ium tetrafluoroborate (**3s**) in CD_3CN (400 MHz) at 23 °C.

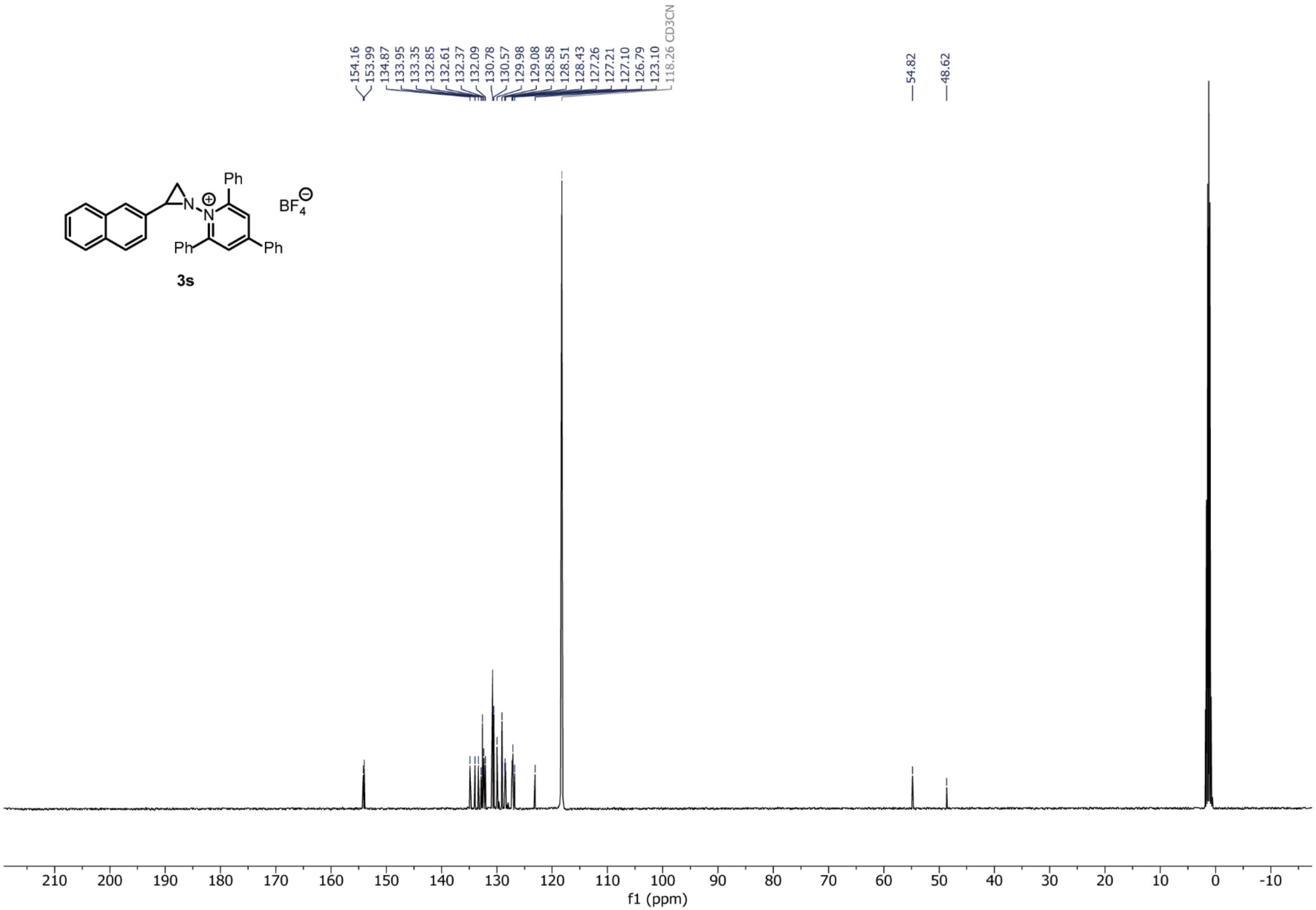

Supplementary Figure 45. ^{13}C NMR spectrum of 1-(2-(naphthalen-2-yl)aziridin-1-yl)-2,4,6-triphenylpyridin-1-ium tetrafluoroborate (**3s**) in CD_3CN (101 MHz) at 23 °C.

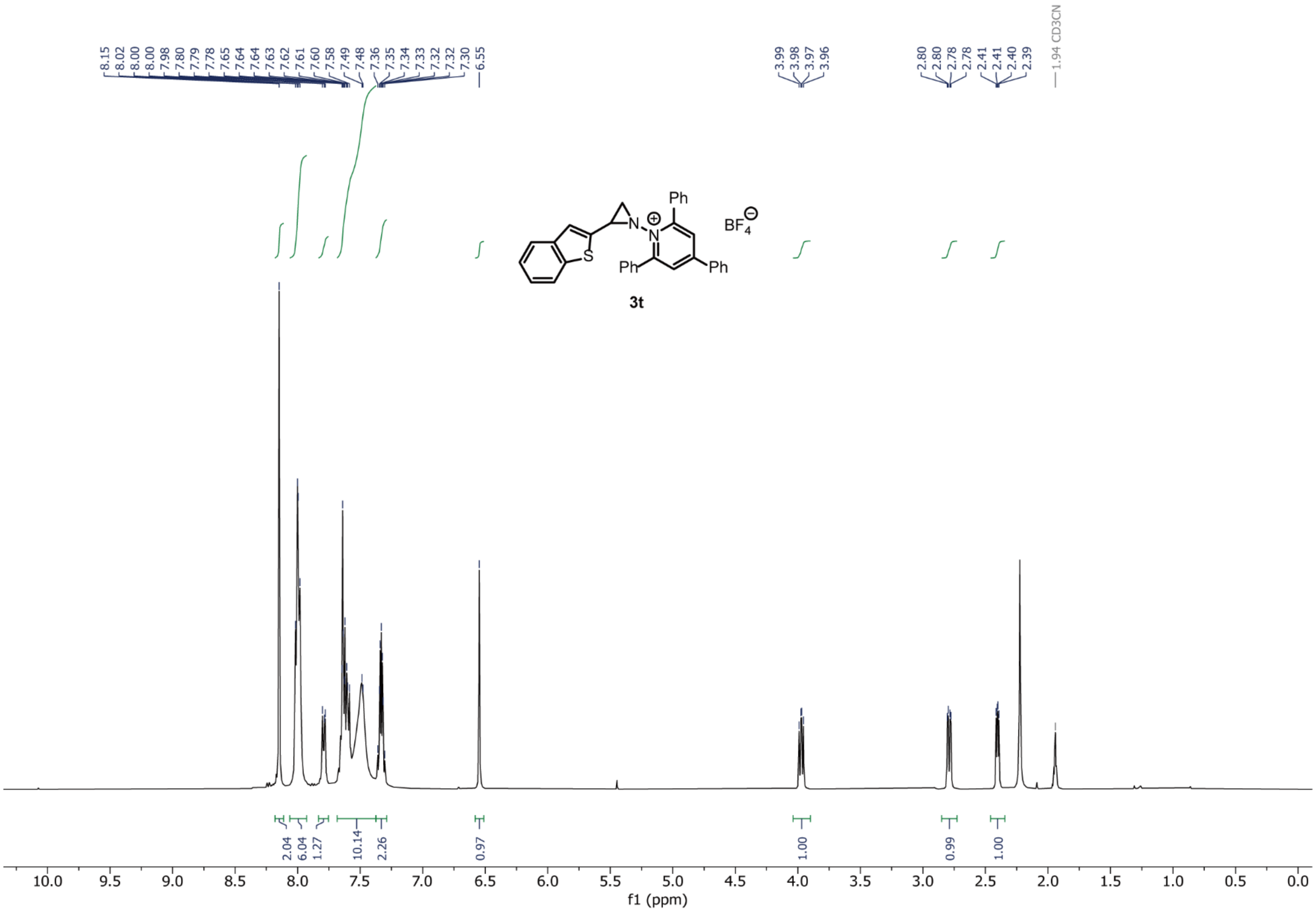

Supplementary Figure 46. ^{1}H NMR spectrum of 1-(2-(benzo[*b*]thiophen-2-yl)aziridin-1-yl)-2,4,6-triphenylpyridin-1-ium tetrafluoroborate (**3t**) in CD_3CN (400 MHz) at 23 °C.

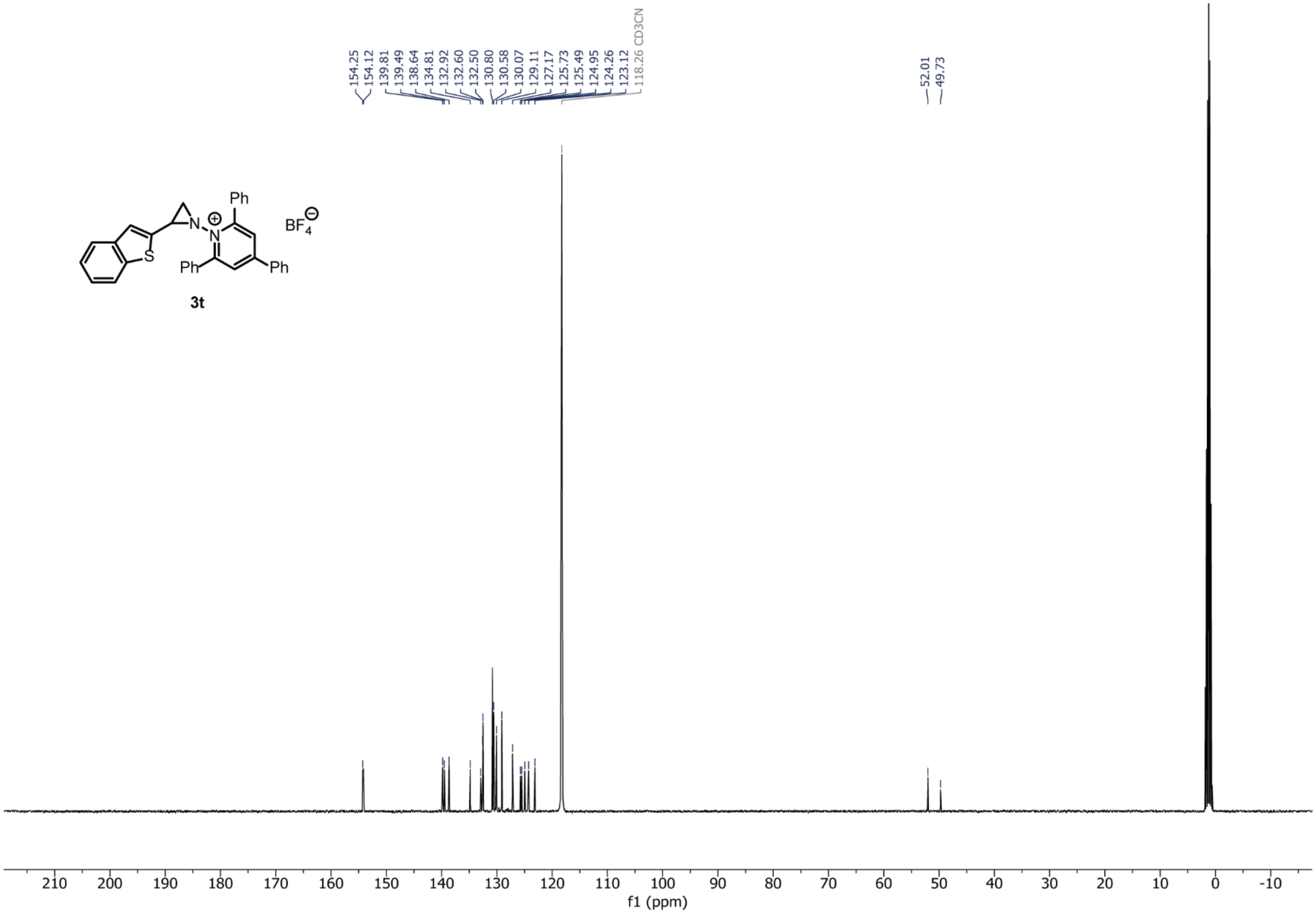

3t

Supplementary Figure 47. ^{13}C NMR spectrum of 1-(2-(benzo[*b*]thiophen-2-yl)aziridin-1-yl)-2,4,6-triphenylpyridin-1-ium tetrafluoroborate (**3t**) in CD_3CN (101 MHz) at 23 °C.

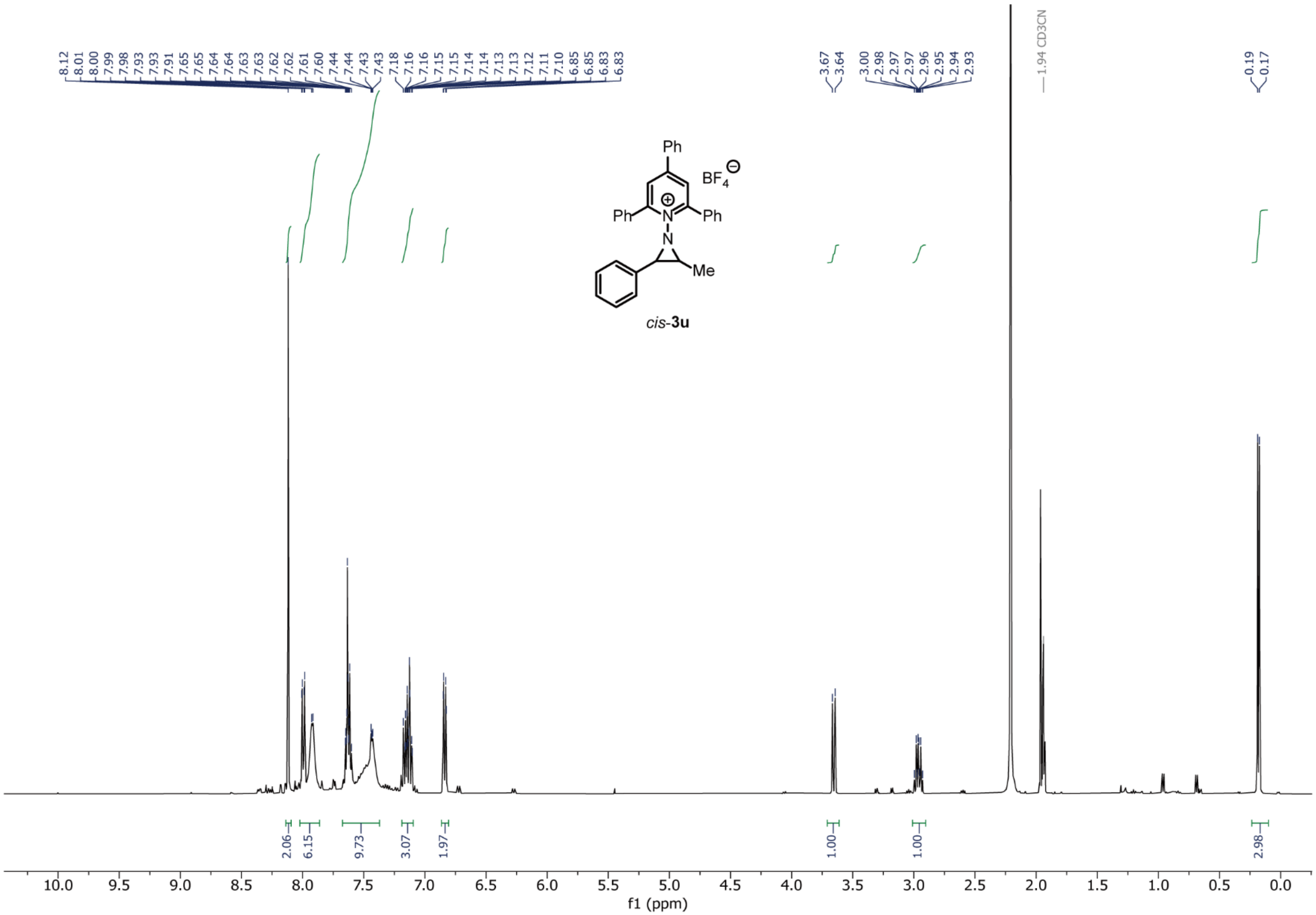

Supplementary Figure 48. ^{1}H NMR spectrum of *cis*-1-(2-methyl-3-phenylaziridin-1-yl)-2,4,6-triphenylpyridin-1-ium tetrafluoroborate (*cis*-**3u**) in CD_3CN (400 MHz) at 23 °C.

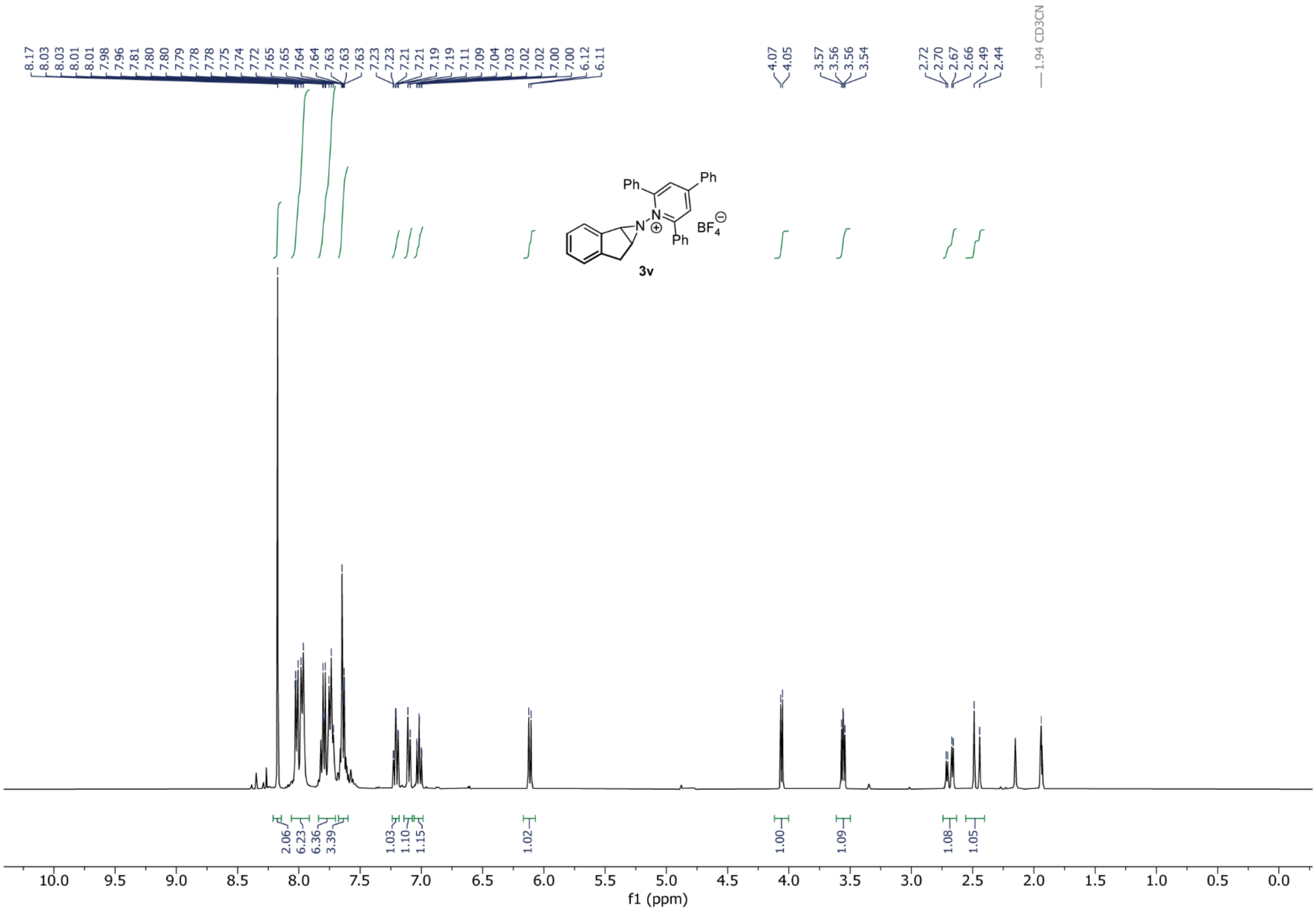

Supplementary Figure 49. ^{1}H NMR spectrum of 1-(6,6a-dihydroindeno[1,2-*b*]azirin-1(1a*H*)-yl)-2,4,6-triphenylpyridin-1-ium tetrafluoroborate (**3v**) in CD_3CN (400 MHz) at 23 °C.

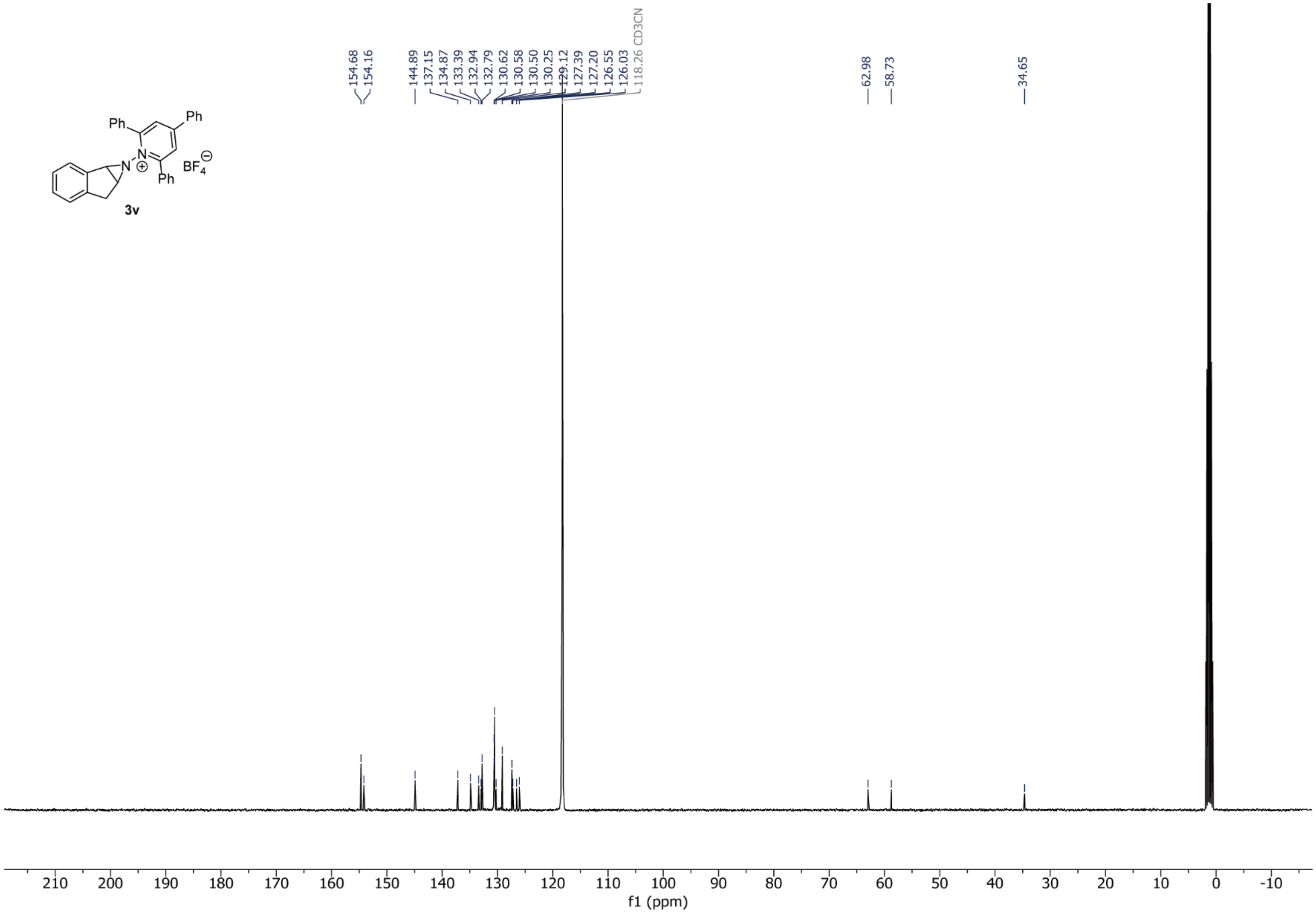

Supplementary Figure 50. ^{13}C NMR spectrum of 1-(6,6a-dihydroindeno[1,2-*b*]azirin-1(1a*H*)-yl)-2,4,6-triphenylpyridin-1-ium tetrafluoroborate (**3v**) in CD_3CN (101 MHz) at 23 °C.

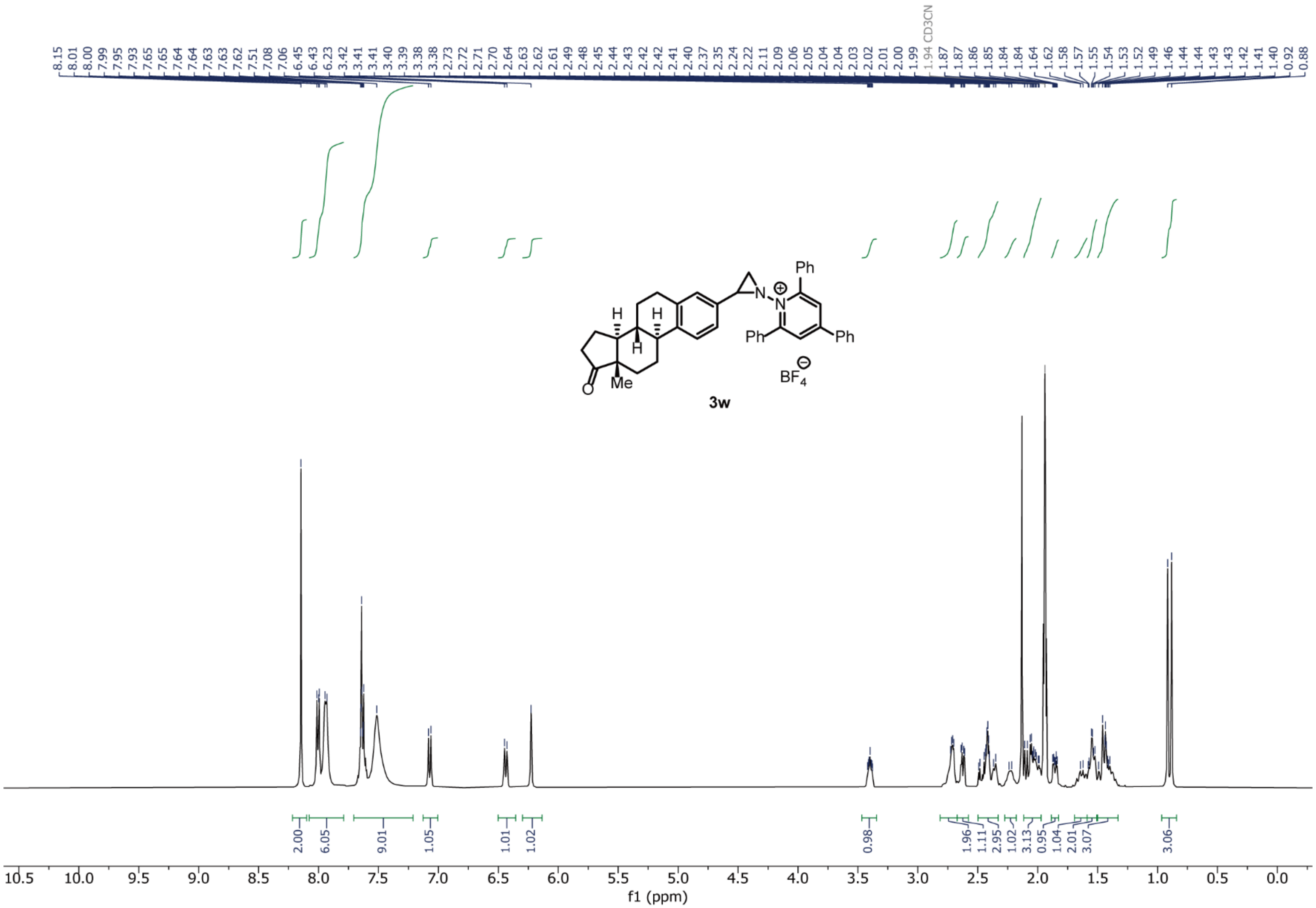

Supplementary Figure 51. ^{1}H NMR spectrum of 1-(2-((8*R*,9*S*,13*S*,14*S*)-13-methyl-17-oxo-7,8,9,11,12,13,14,15,16,17-decahydro-6*H*-cyclopenta[*a*]phenanthren-3-yl)aziridin-1-yl)-2,4,6-triphenylpyridin-1-ium tetrafluoroborate (**3w**) in CD_3CN (400 MHz) at 23 °C.

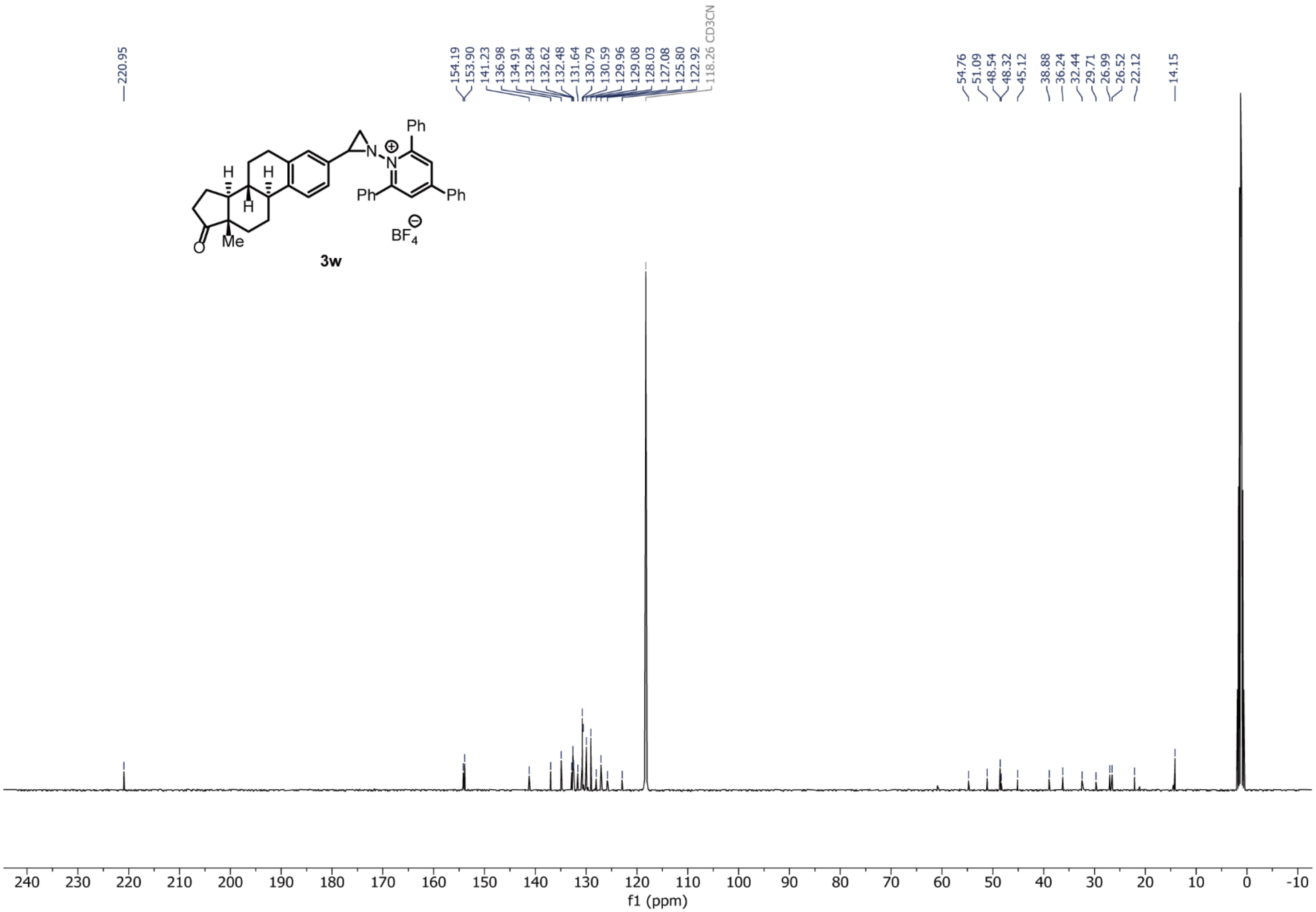

Supplementary Figure 52. ^{13}C NMR spectrum of 1-(2-((8*R*,9*S*,13*S*,14*S*)-13-methyl-17-oxo-7,8,9,11,12,13,14,15,16,17-decahydro-6*H*-cyclopenta[*a*]phenanthren-3-yl)aziridin-1-yl)-2,4,6-triphenylpyridin-1-ium tetrafluoroborate (**3w**) in CD_3CN (101 MHz) at 23 °C.

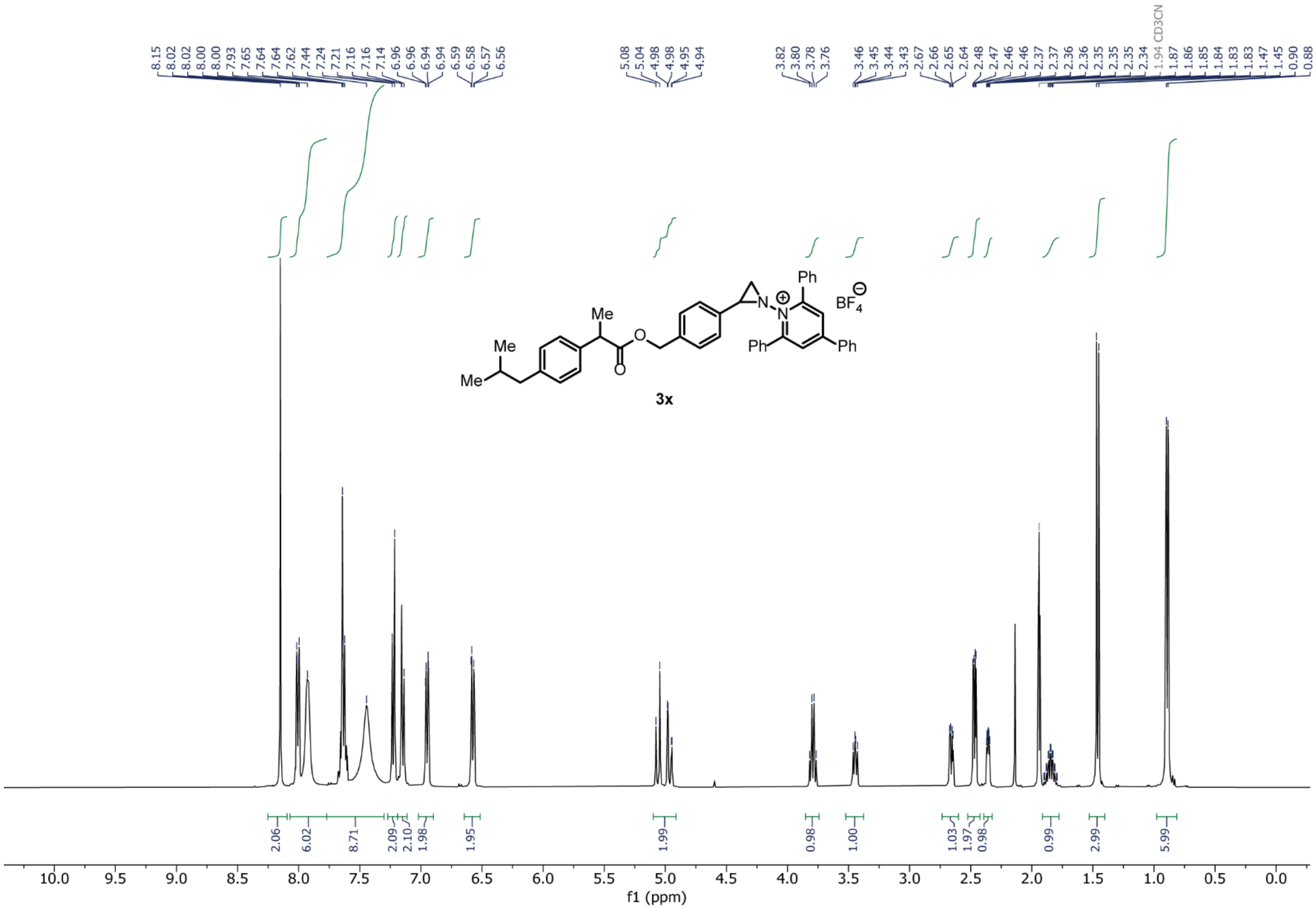

Supplementary Figure 53. ^{1}H NMR spectrum of 1-(2-(4-(((2-(4-isobutylphenyl)propanoyl)oxy)methyl)phenyl)aziridin-1-yl)-2,4,6-triphenylpyridin-1-ium tetrafluoroborate (**3x**) in CD_3CN (400 MHz) at 23 °C.

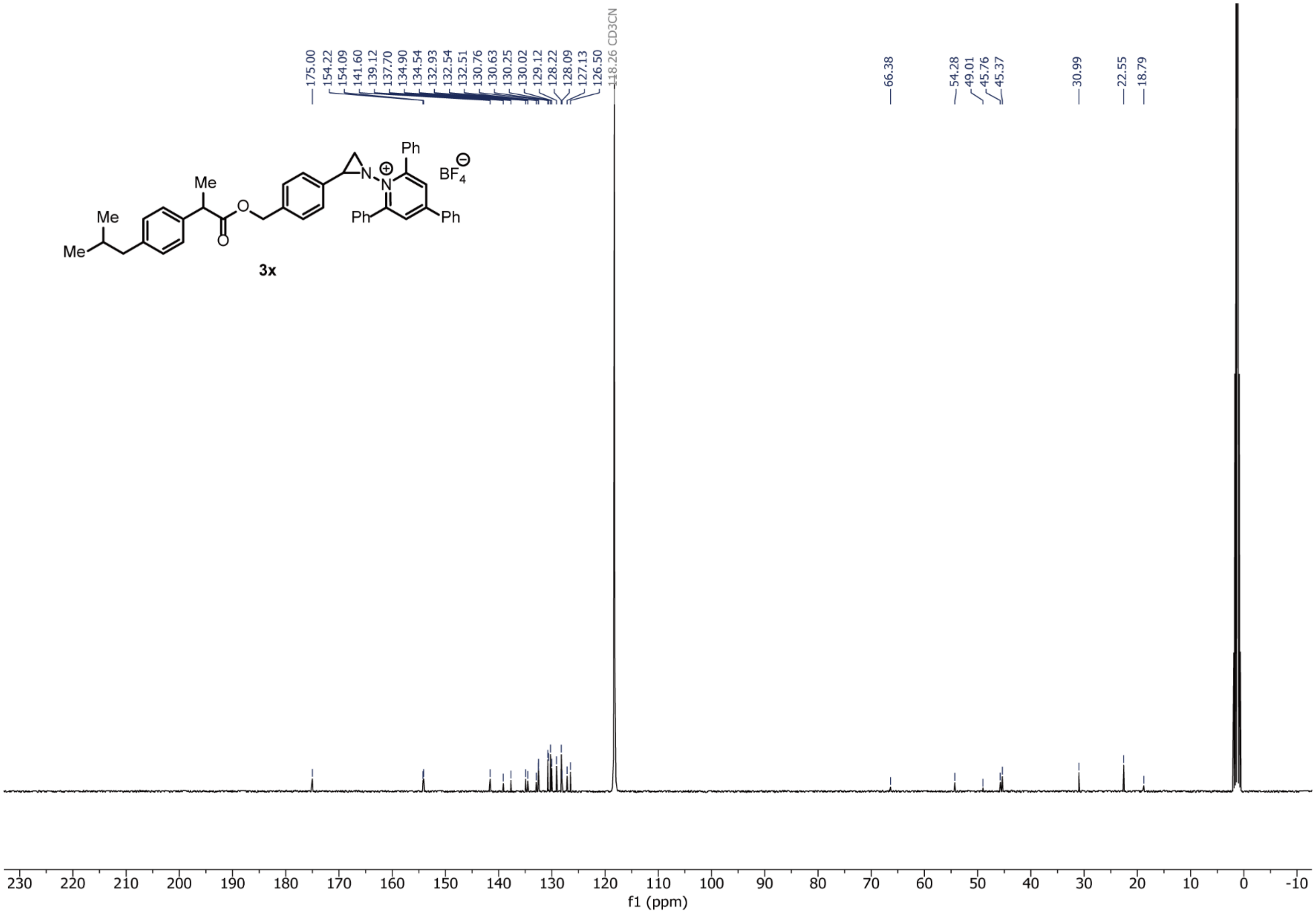

Supplementary Figure 54. ^{13}C NMR spectrum of 1-(2-(4-(((2-(4-isobutylphenyl)propanoyl)oxy)methyl)phenyl)aziridin-1-yl)-2,4,6-triphenylpyridin-1-ium tetrafluoroborate (**3x**) in CD_3CN (101 MHz) at 23 °C.

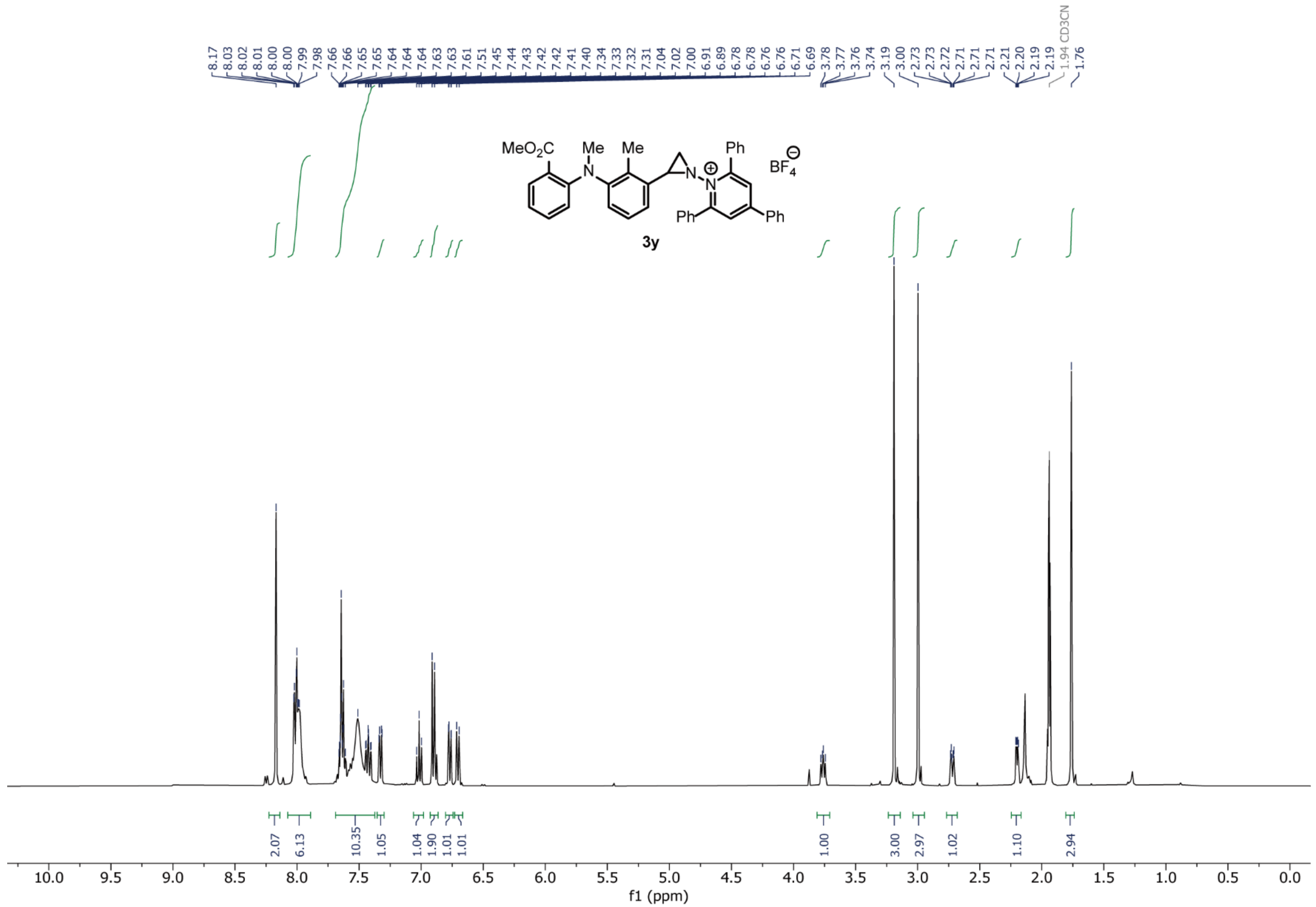

Supplementary Figure 55. ^{1}H NMR spectrum of 1-(2-(3-((2-(methoxycarbonyl)phenyl)(methyl)amino)-2-methylphenyl)aziridin-1-yl)-2,4,6-triphenylpyridin-1-ium tetrafluoroborate (**3y**) in CD_3CN (400 MHz) at 23 °C.

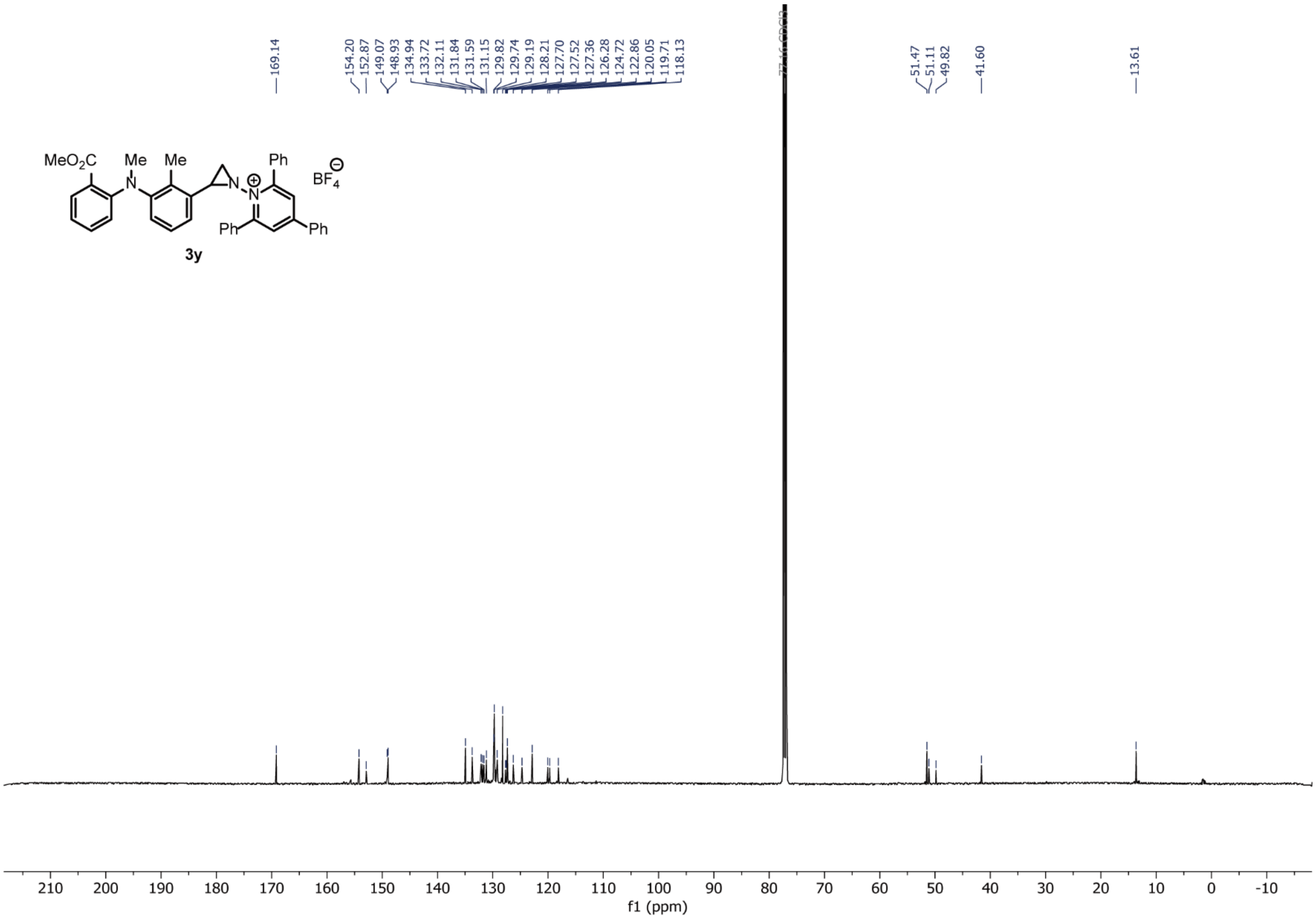

Supplementary Figure 56. ^{13}C NMR spectrum of 1-(2-(3-((2-(methoxycarbonyl)phenyl)(methyl)amino)-2-methylphenyl)aziridin-1-yl)-2,4,6-triphenylpyridin-1-ium tetrafluoroborate (**3y**) in $CDCl_3$ (101 MHz) at 23 °C.

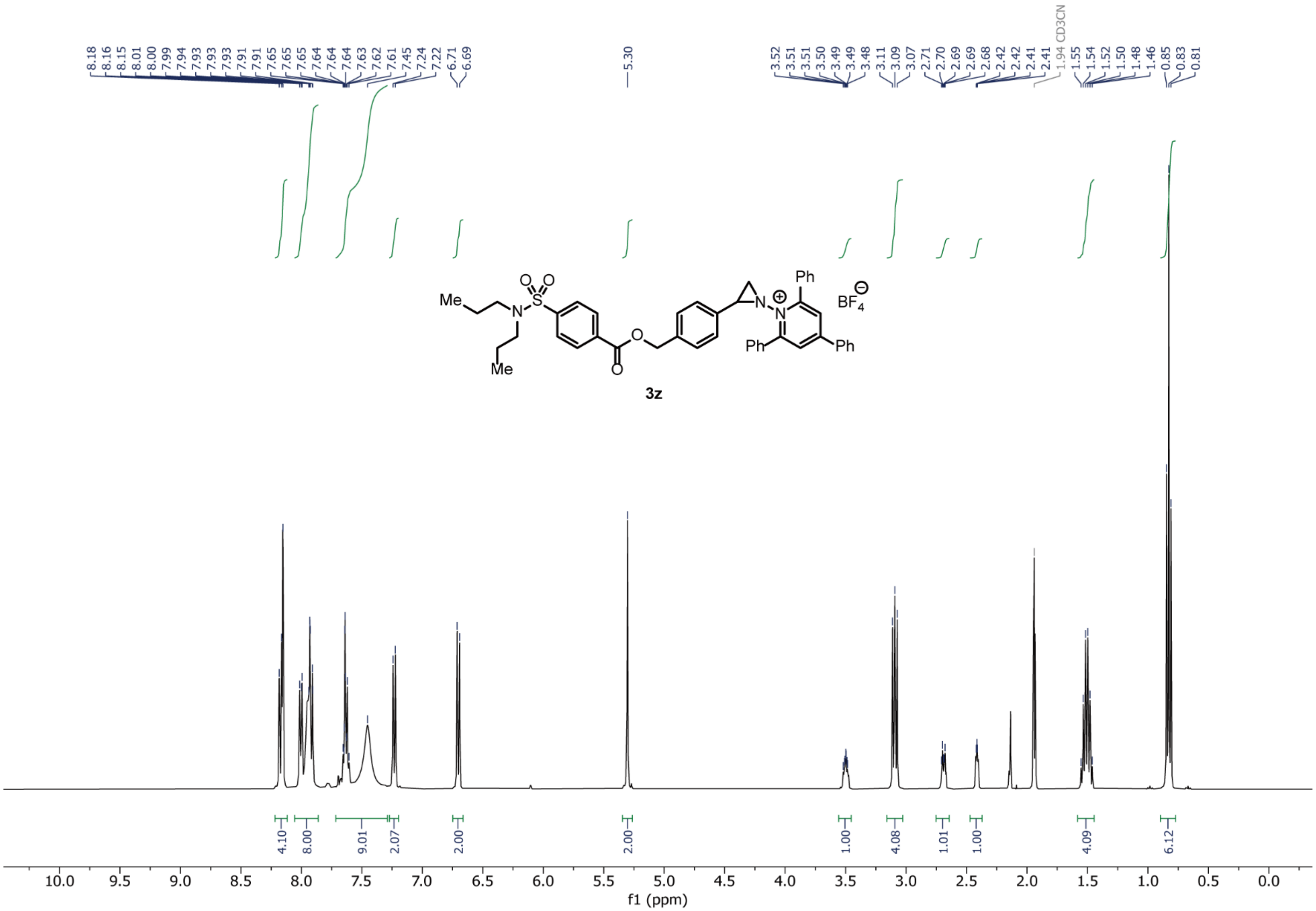

Supplementary Figure 57. ^{1}H NMR spectrum of 1-(2-(4-(((4-(*N*,*N*-dipropylsulfamoyl)benzoyl)oxy)methyl)phenyl)aziridin-1-yl)-2,4,6-triphenylpyridin-1-ium tetrafluoroborate (**3z**) in CD_3CN (400 MHz) at 23 °C.

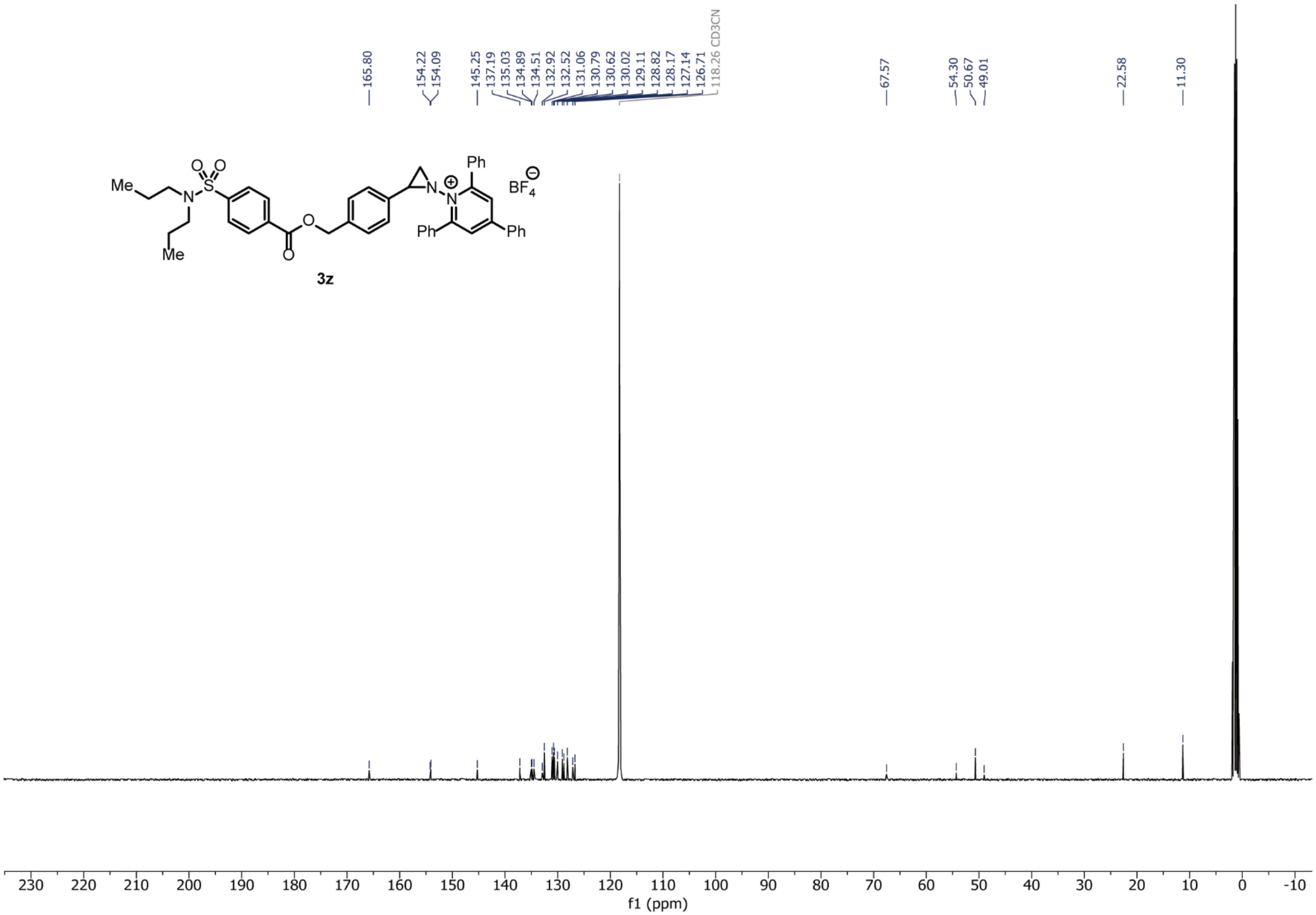

Supplementary Figure 58. ^{13}C NMR spectrum of 1-(2-(4-(((4-(*N*,*N*-dipropylsulfamoyl)benzoyl)oxy)methyl)phenyl)aziridin-1-yl)-2,4,6-triphenylpyridin-1-ium tetrafluoroborate (**3z**) in CD_3CN (101 MHz) at 23 °C.

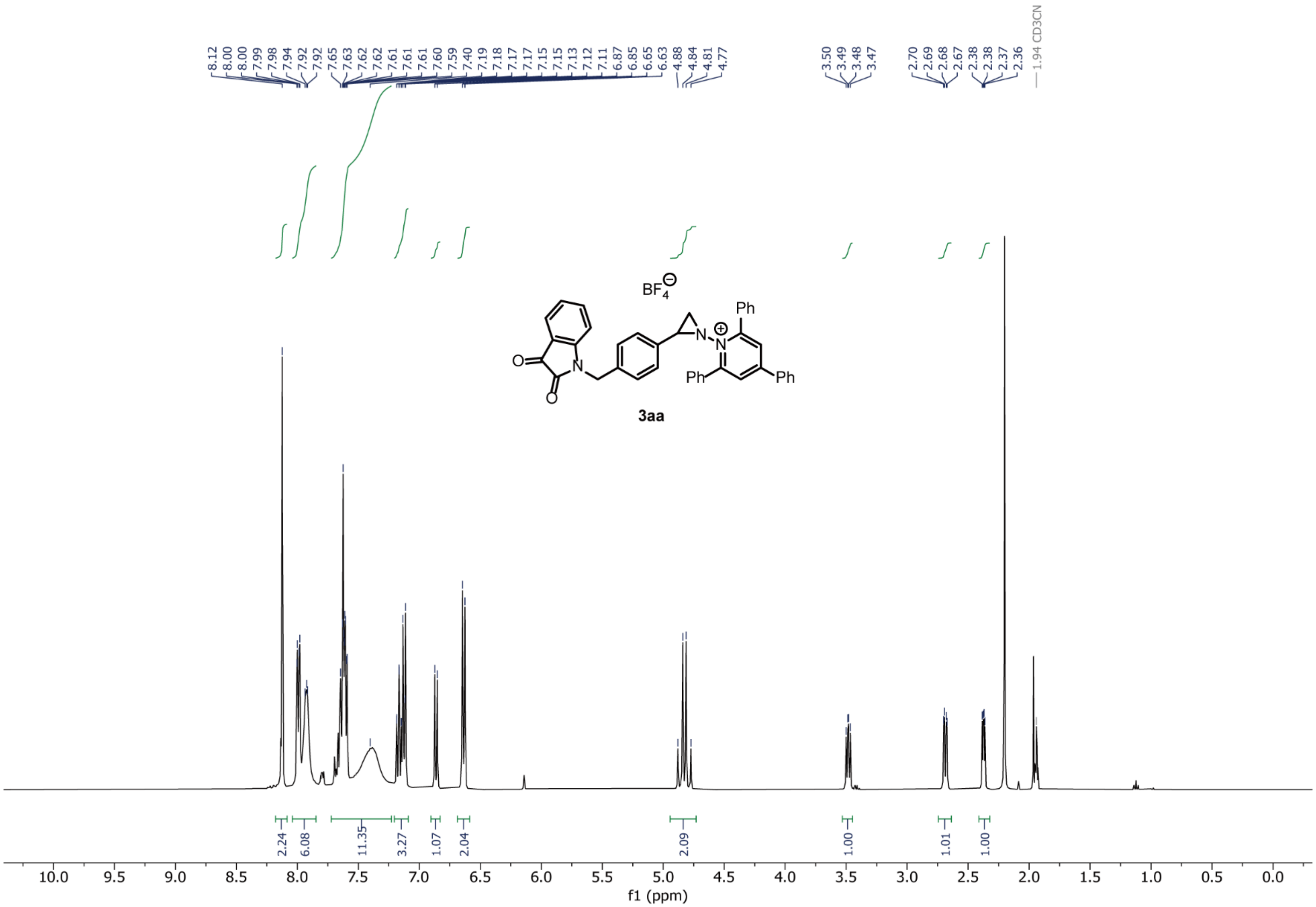

Supplementary Figure 59. ^{1}H NMR spectrum of 1-(2-(4-((2,3-dioxoindolin-1-yl)methyl)phenyl)aziridin-1-yl)-2,4,6-triphenylpyridin-1-ium tetrafluoroborate (**3aa**) in CD_3CN (400 MHz) at 23 °C.

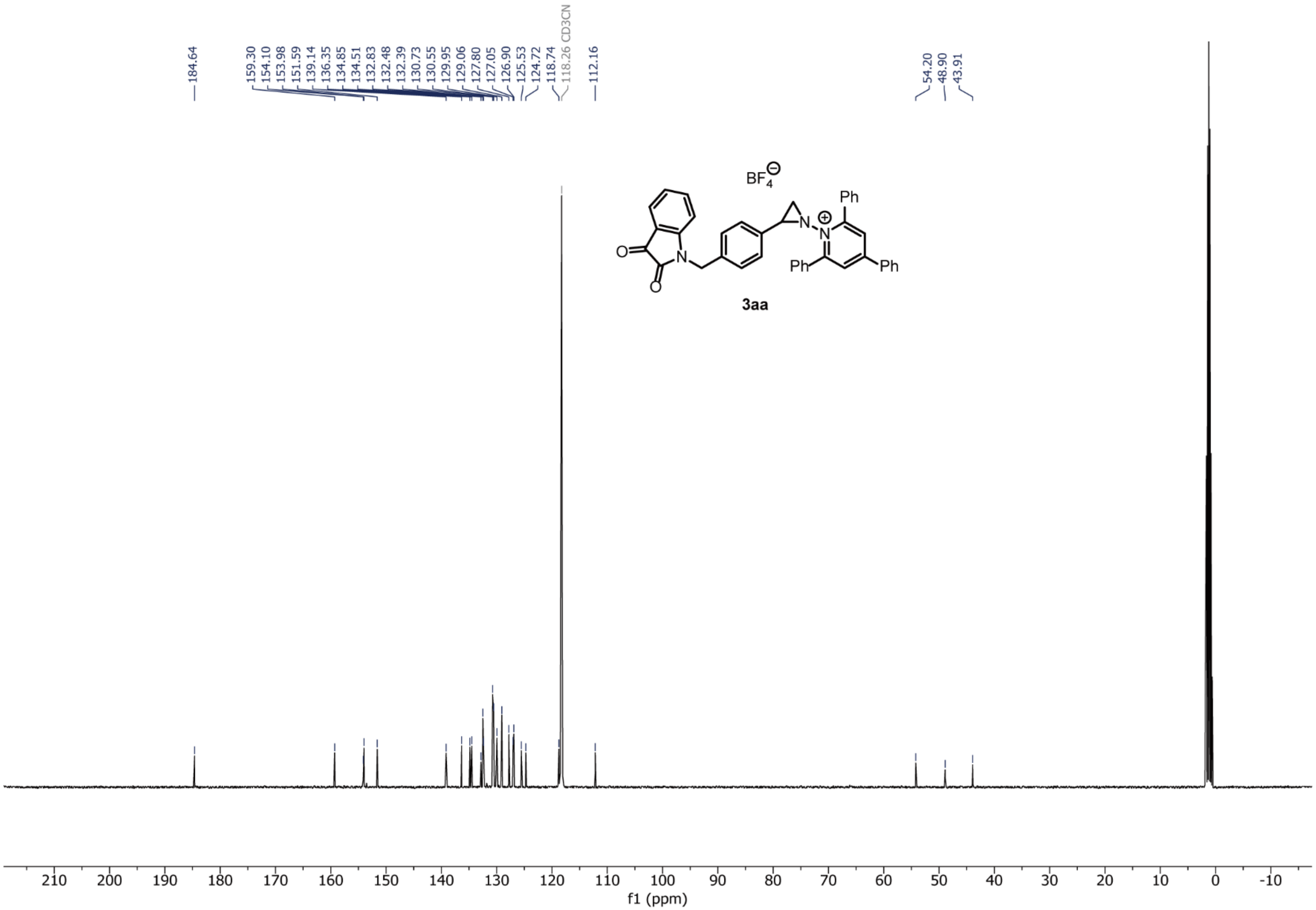

Supplementary Figure 60. ^{13}C NMR spectrum of 1-(2-(4-((2,3-dioxoindolin-1-yl)methyl)phenyl)aziridin-1-yl)-2,4,6-triphenylpyridin-1-ium tetrafluoroborate (**3aa**) in CD_3CN (101 MHz) at 23 °C.

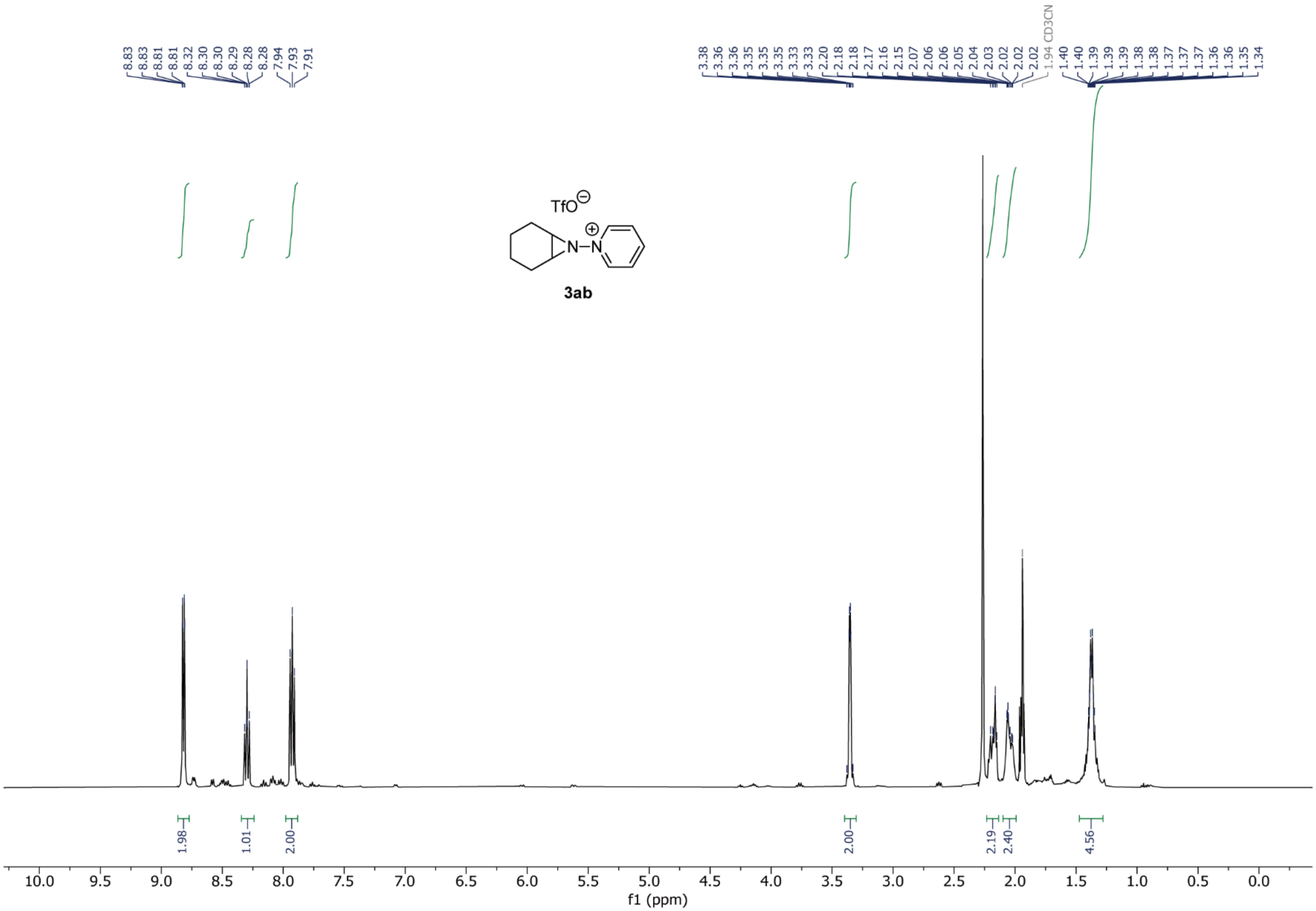

Supplementary Figure 61. ^{1}H NMR spectrum of 1-(7-azabicyclo[4.1.0]heptan-7-yl)pyridin-1-ium trifluoromethanesulfonate (**3ab**) in CD_3CN (400 MHz) at 23 °C.

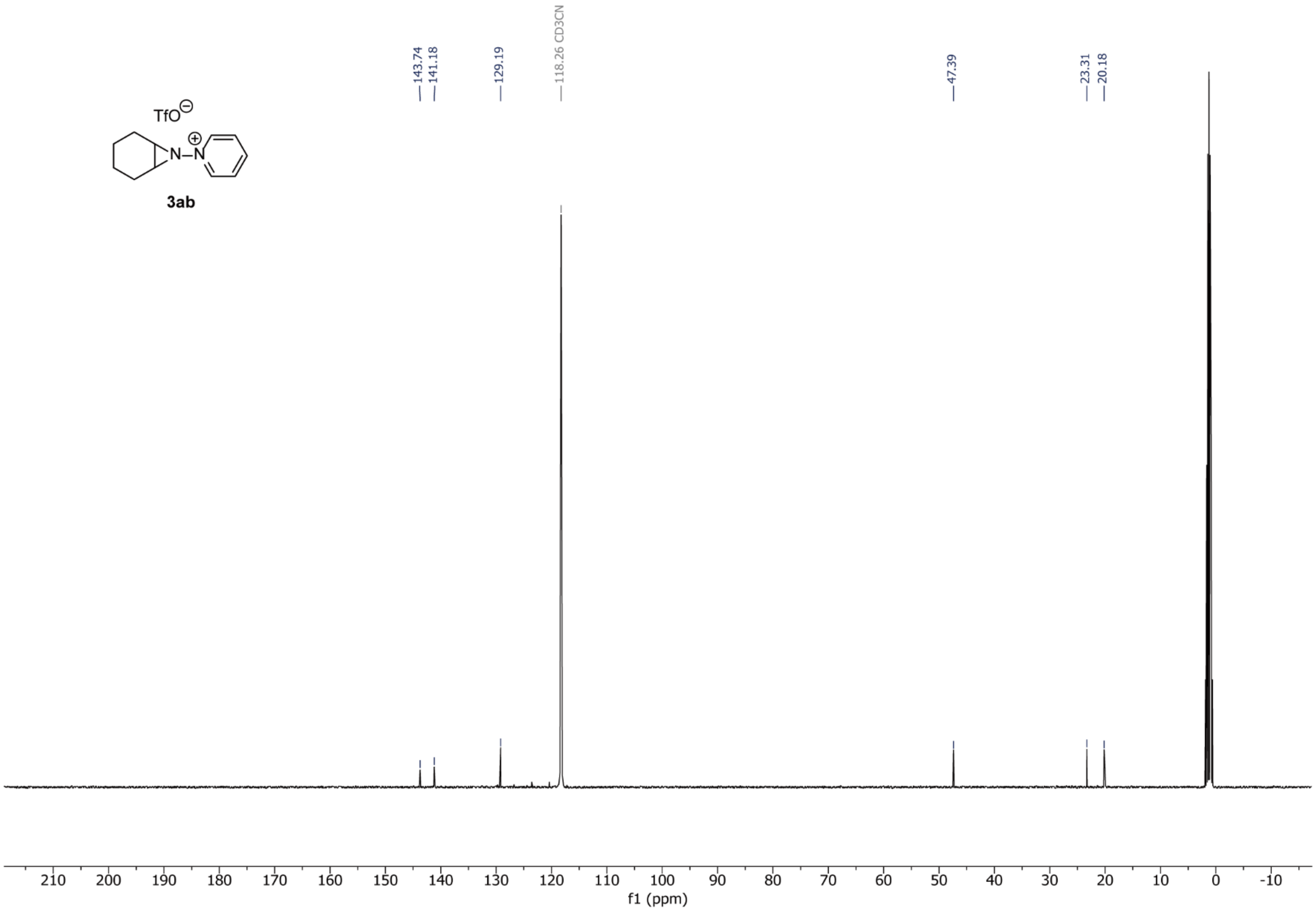

Supplementary Figure 62. ^{13}C NMR spectrum of 1-(7-azabicyclo[4.1.0]heptan-7-yl)pyridin-1-ium trifluoromethanesulfonate (**3ab**) in CD_3CN (101 MHz) at 23 °C.

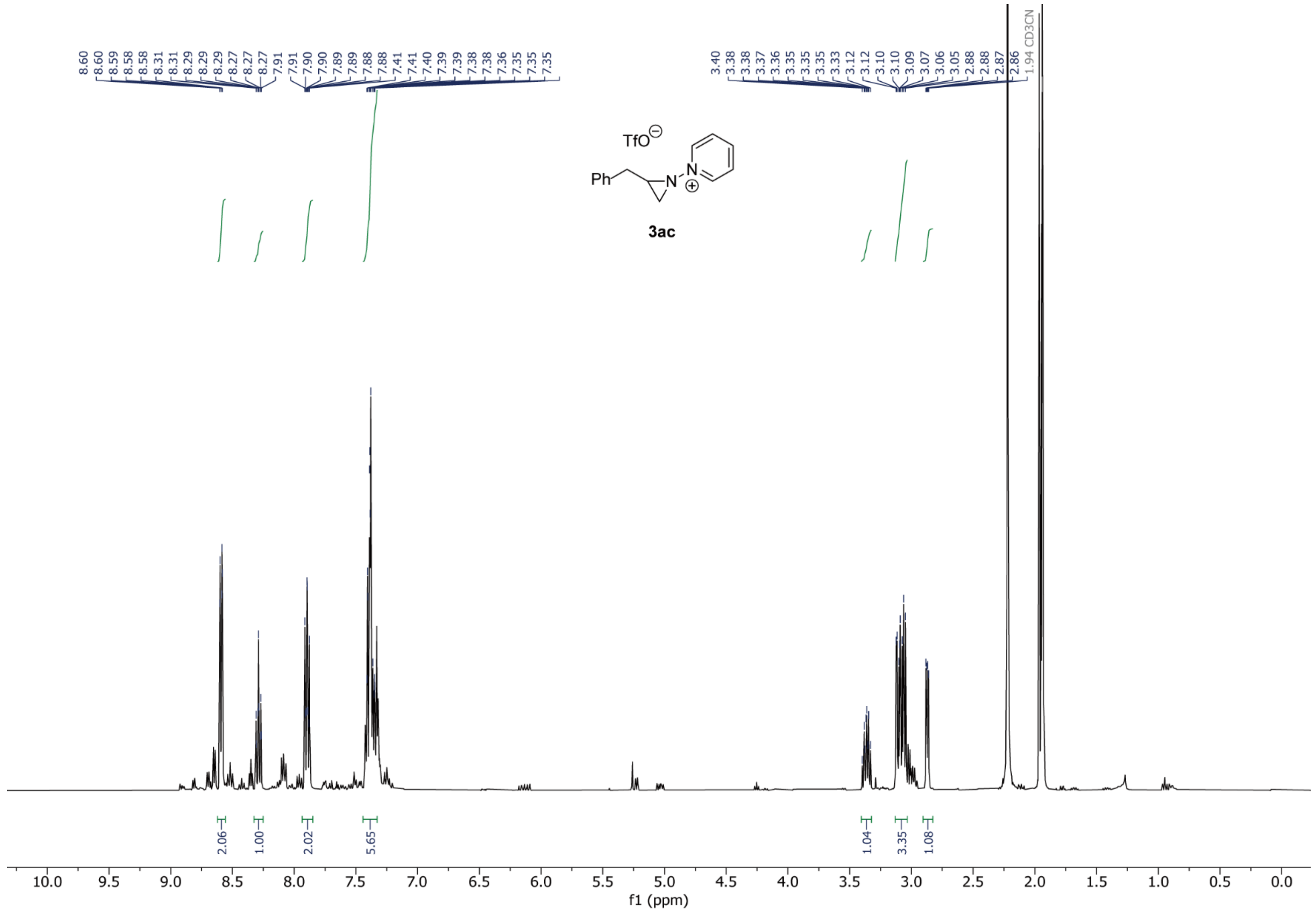

Supplementary Figure 63. ^{1}H NMR spectrum of 1-(2-benzylaziridin-1-yl)pyridin-1-ium trifluoromethanesulfonate (**3ac**) in CD_3CN (400 MHz) at 23 °C.

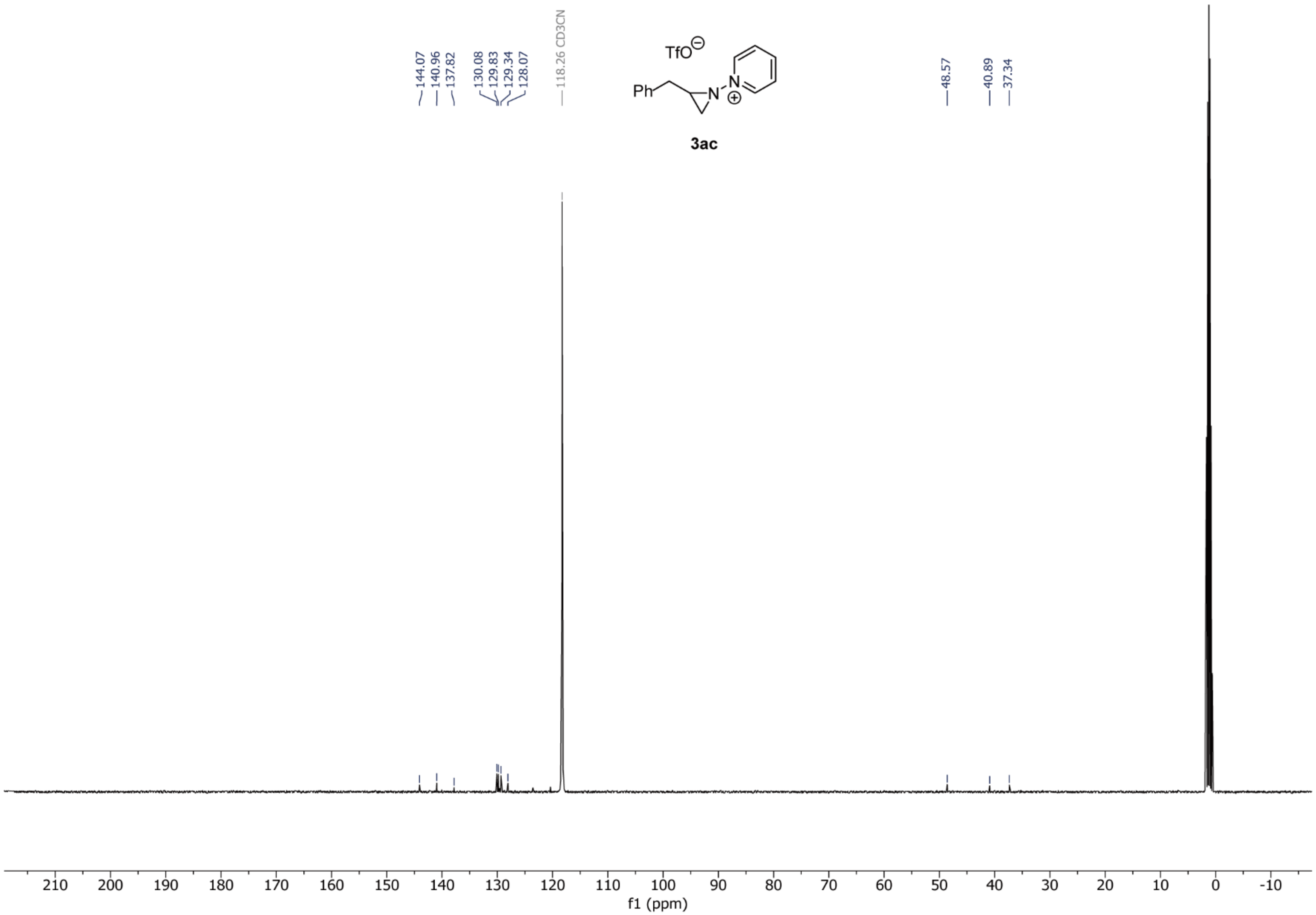

Supplementary Figure 64. ^{13}C NMR spectrum of 1-(2-benzylaziridin-1-yl)pyridin-1-ium trifluoromethanesulfonate (**3ac**) in CD_3CN (101 MHz) at 23 °C.

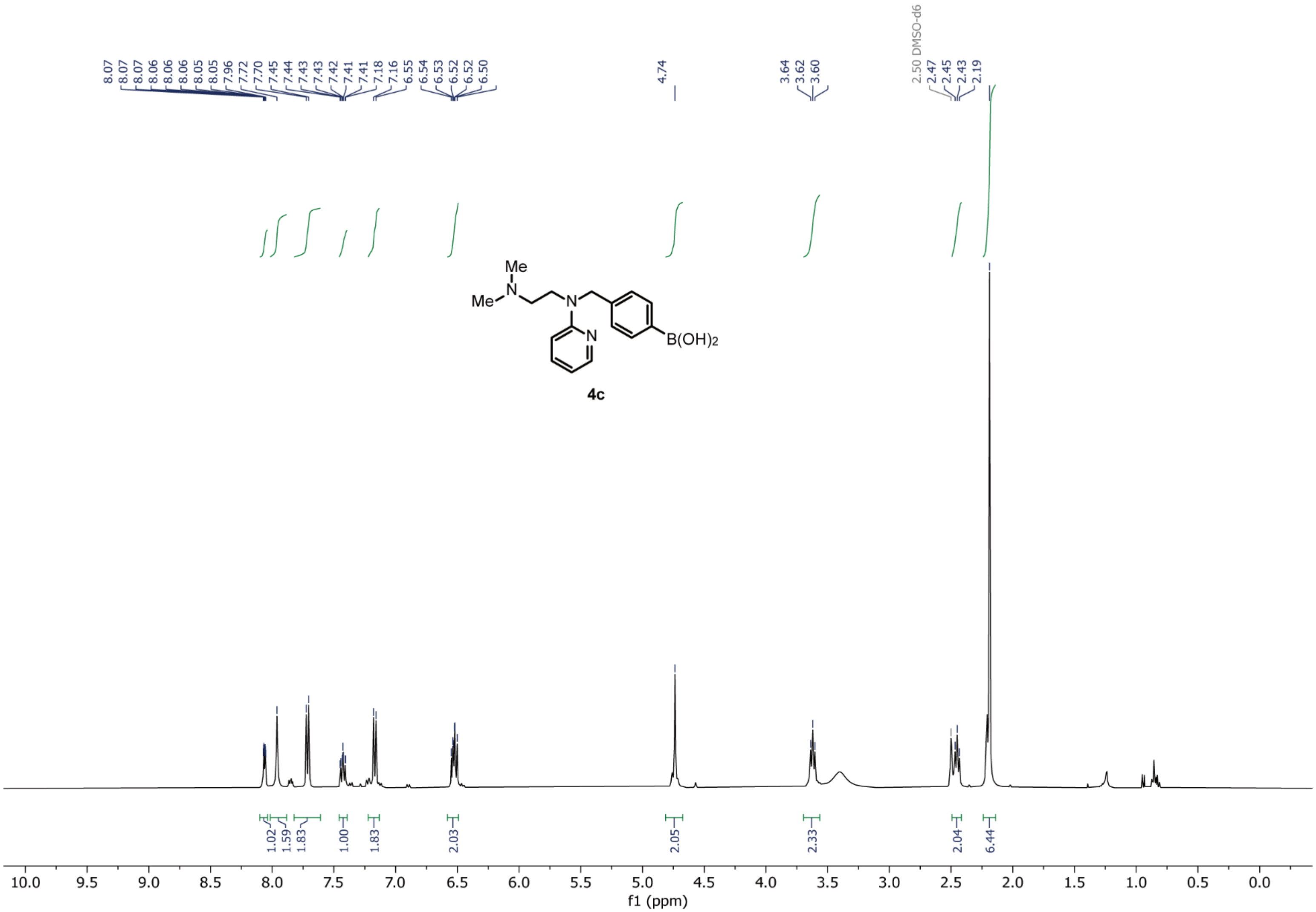

Supplementary Figure 65. ^{1}H NMR spectrum of (4-(((2-(dimethylamino)ethyl)(pyridin-2-yl)amino)methyl)phenyl)boronic acid (**4c**) in DMSO-d_6 (400 MHz) at 23 °C.

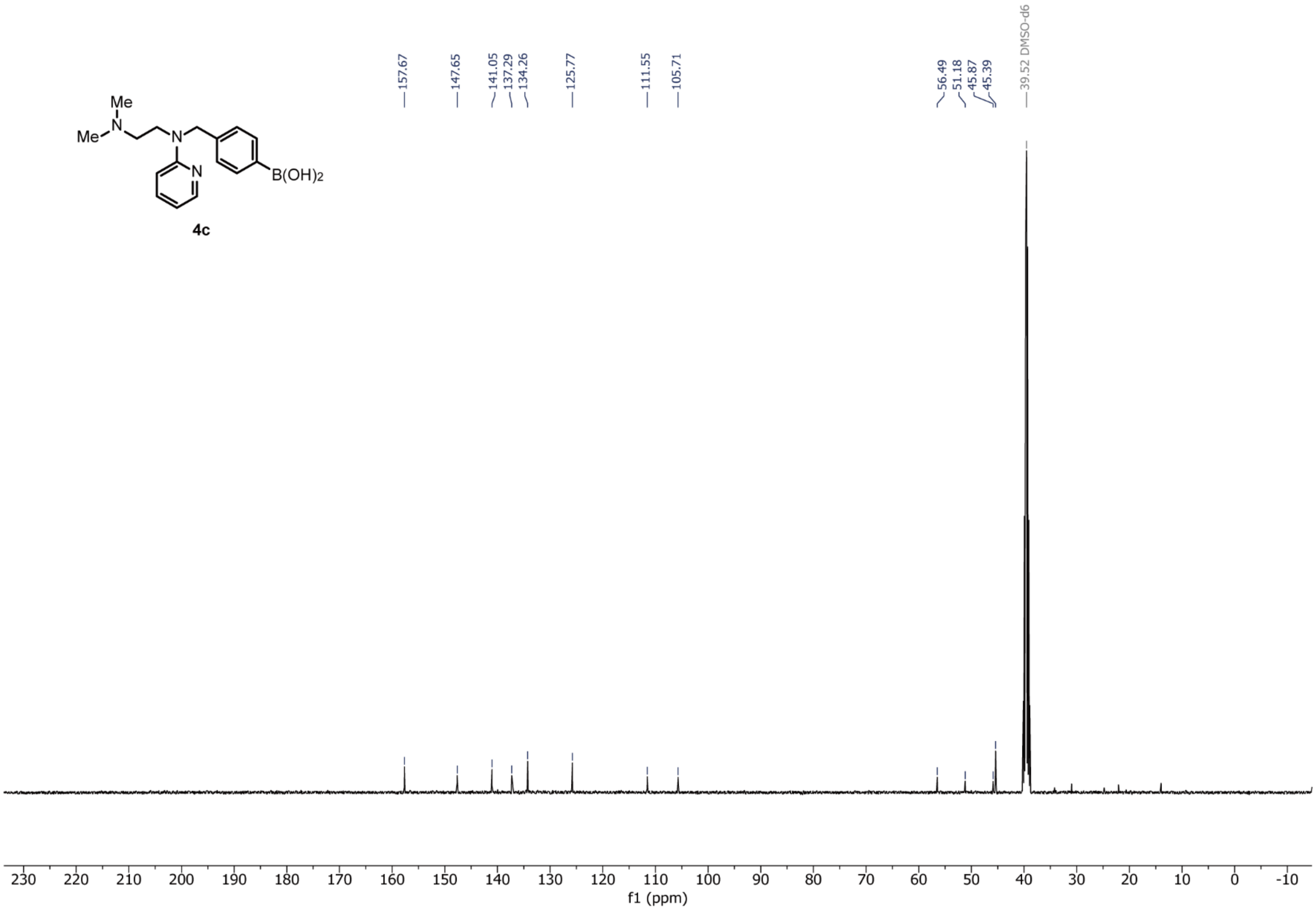

Supplementary Figure 66. ^{13}C NMR spectrum of (4-(((2-(dimethylamino)ethyl)(pyridin-2-yl)amino)methyl)phenyl)boronic acid (**4c**) in DMSO-d_6 (101 MHz) at 23 °C.

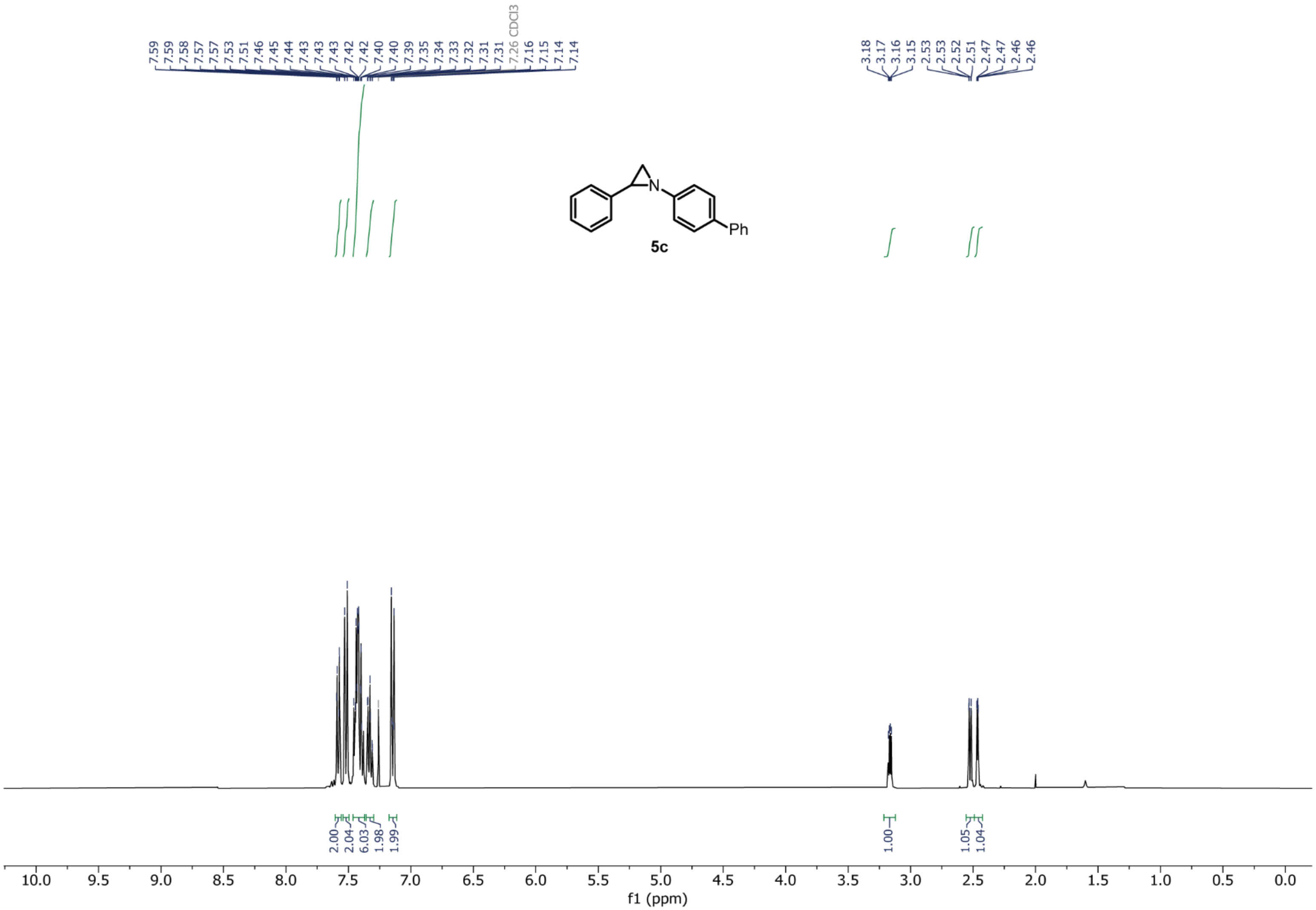

Supplementary Figure 67. ^{1}H NMR spectrum of 1-([1,1'-biphenyl]-4-yl)-2-phenylaziridine (**5c**) in $CDCl_3$ (400 MHz) at 23 °C.

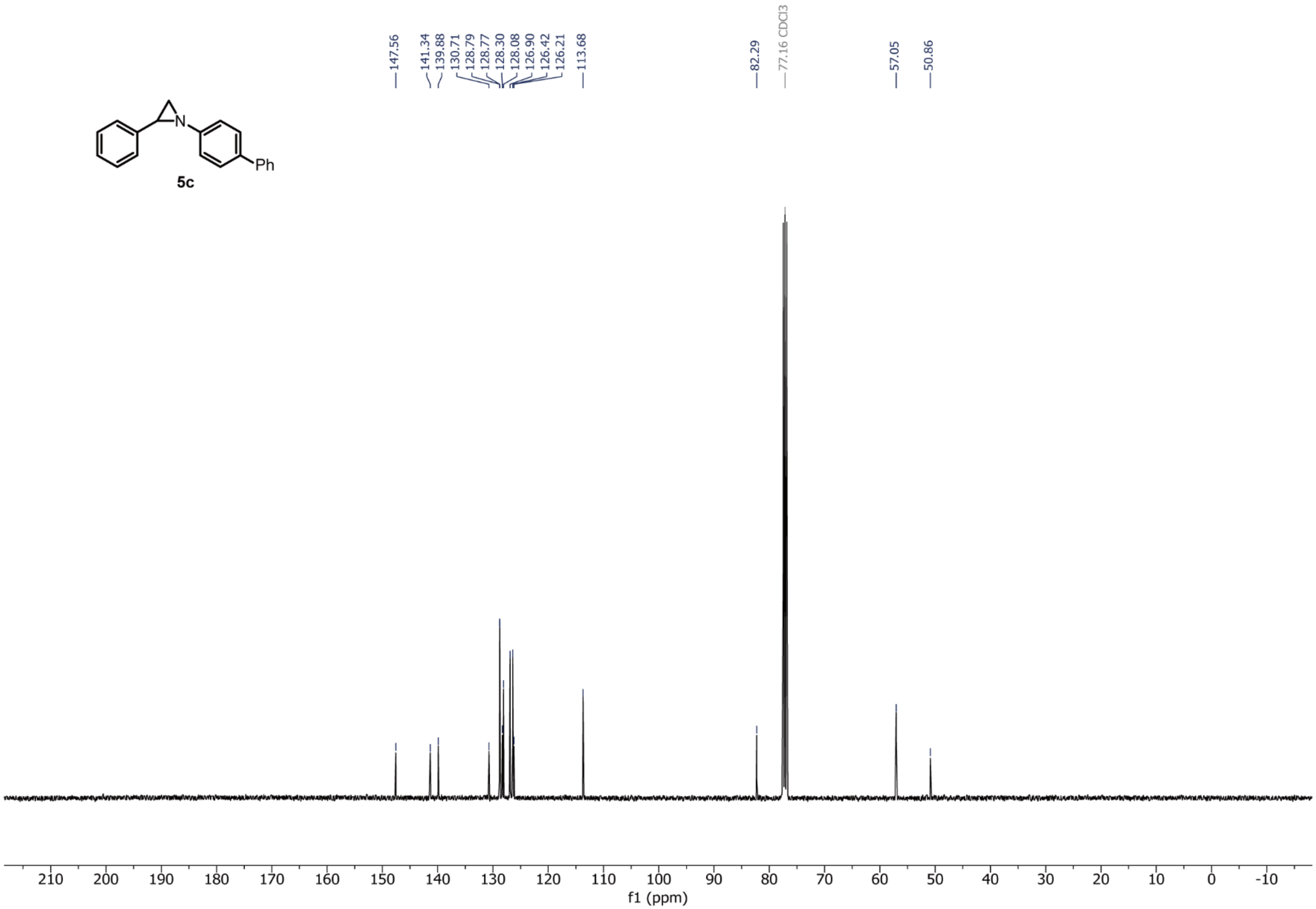

Supplementary Figure 68. ^{13}C NMR spectrum of 1-([1,1'-biphenyl]-4-yl)-2-phenylaziridine (**5c**) in $CDCl_3$ (101 MHz) at 23 °C.

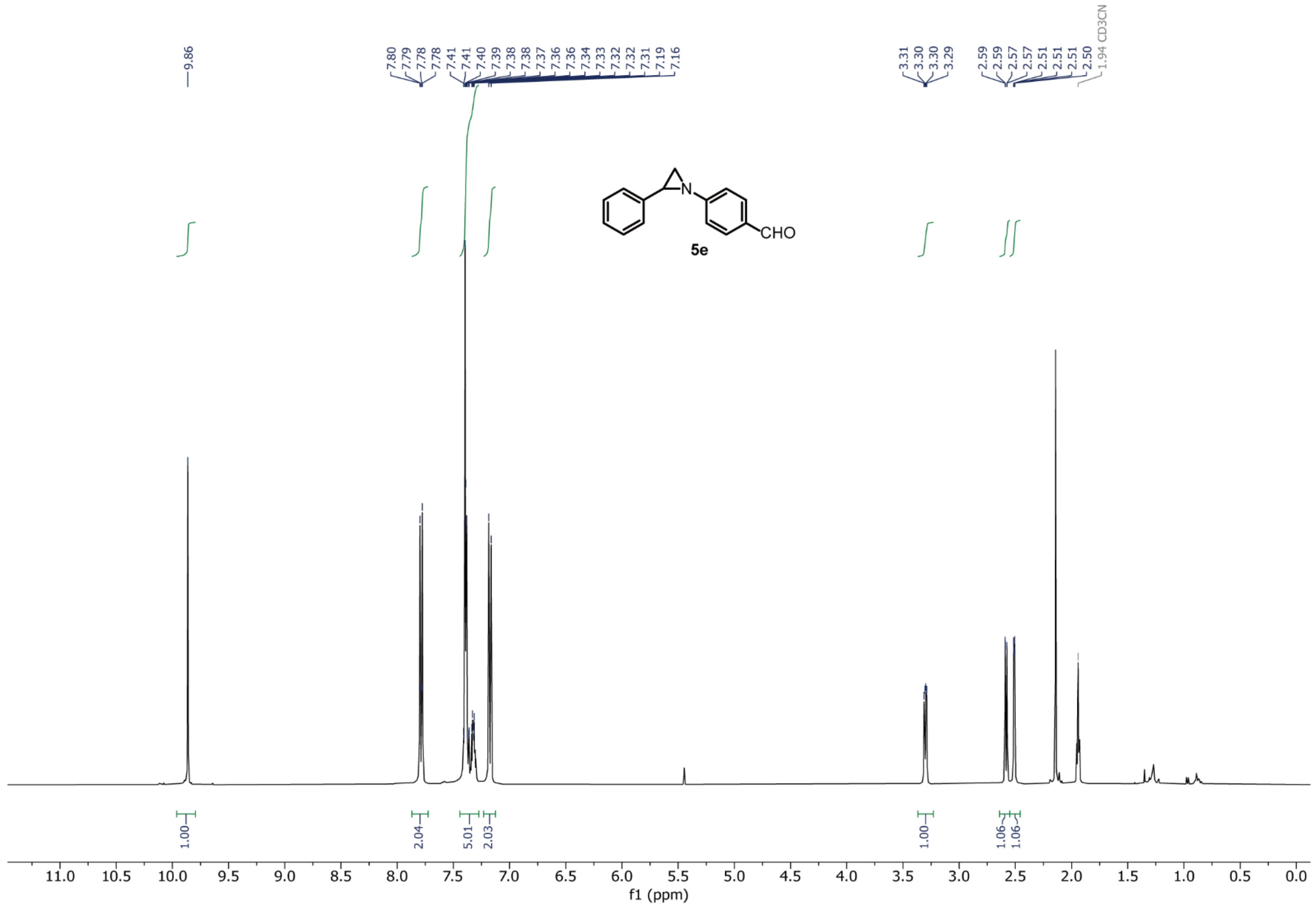

Supplementary Figure 69. ^{1}H NMR spectrum of 4-(2-phenylaziridin-1-yl)benzaldehyde (**5e**) in CD_3CN (400 MHz) at 23 °C.

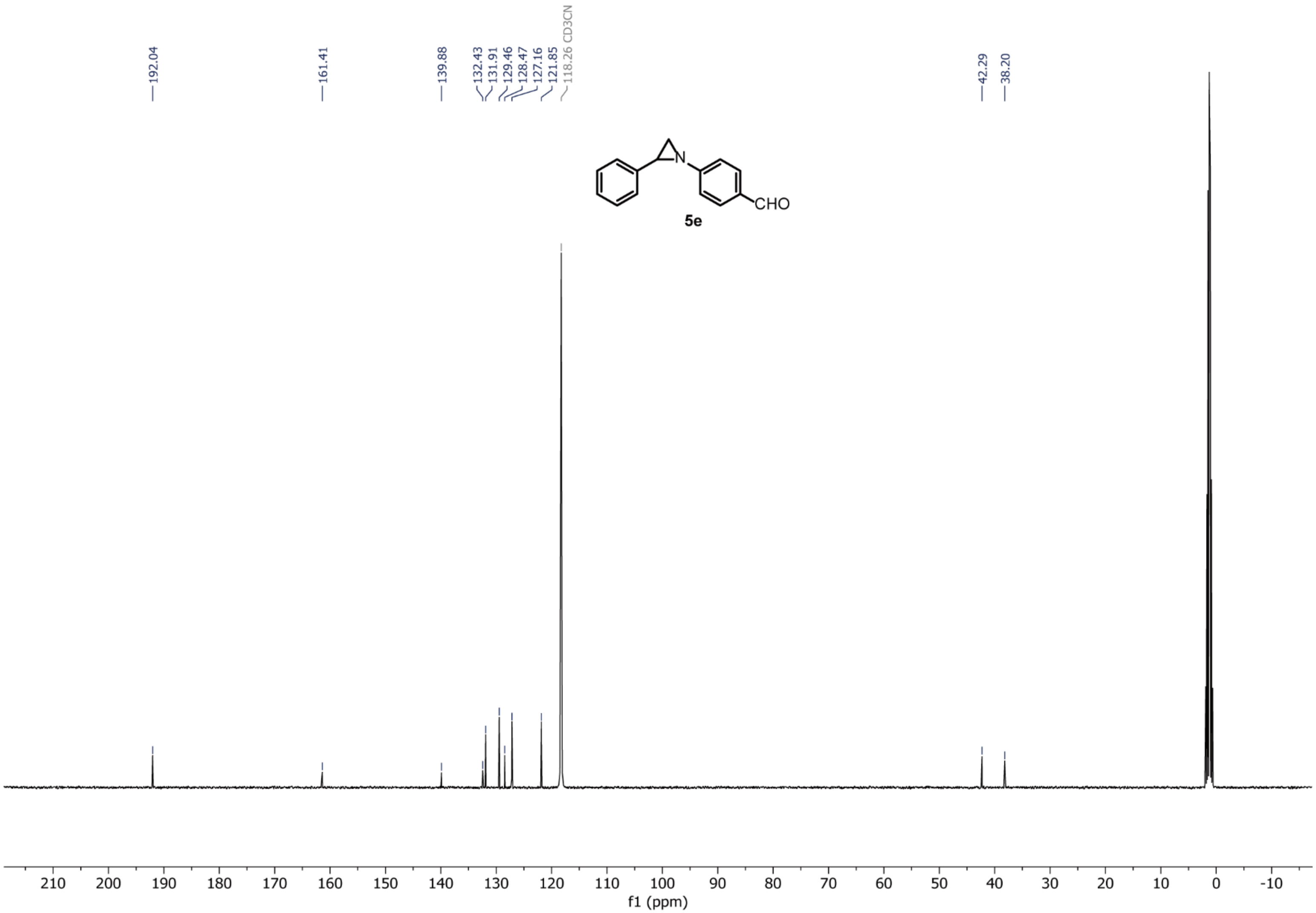

Supplementary Figure 70. ^{13}C NMR spectrum of 4-(2-phenylaziridin-1-yl)benzaldehyde (**5e**) in CD_3CN (101 MHz) at 23 °C.

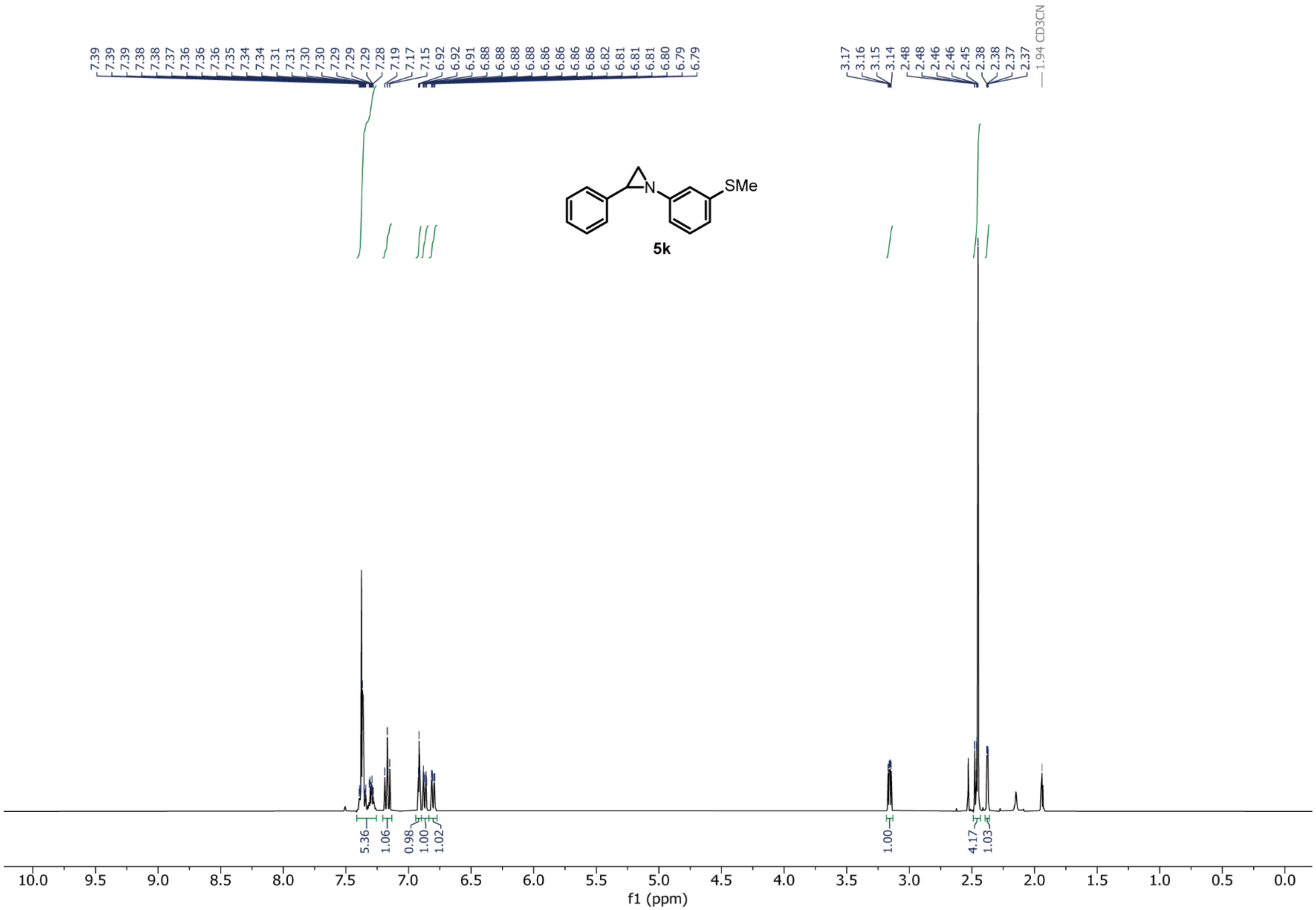

Supplementary Figure 71. ^{1}H NMR spectrum of 1-(3-(methylthio)phenyl)-2-phenylaziridine (**5k**) in CD_3CN (400 MHz) at 23 °C.

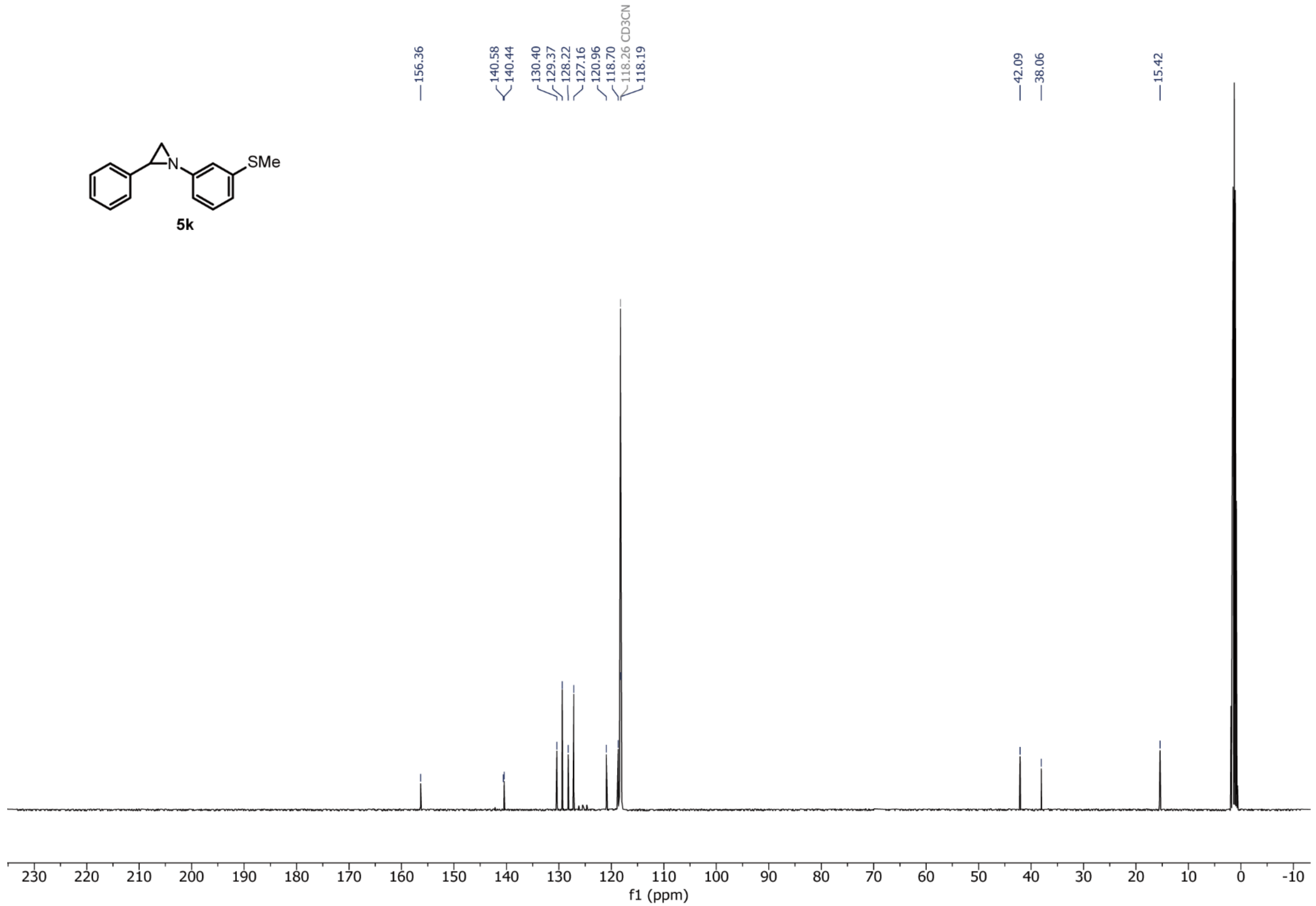

Supplementary Figure 72. ^{13}C NMR spectrum of 1-(3-(methylthio)phenyl)-2-phenylaziridine (**5k**) in CD_3CN (101 MHz) at 23 °C.

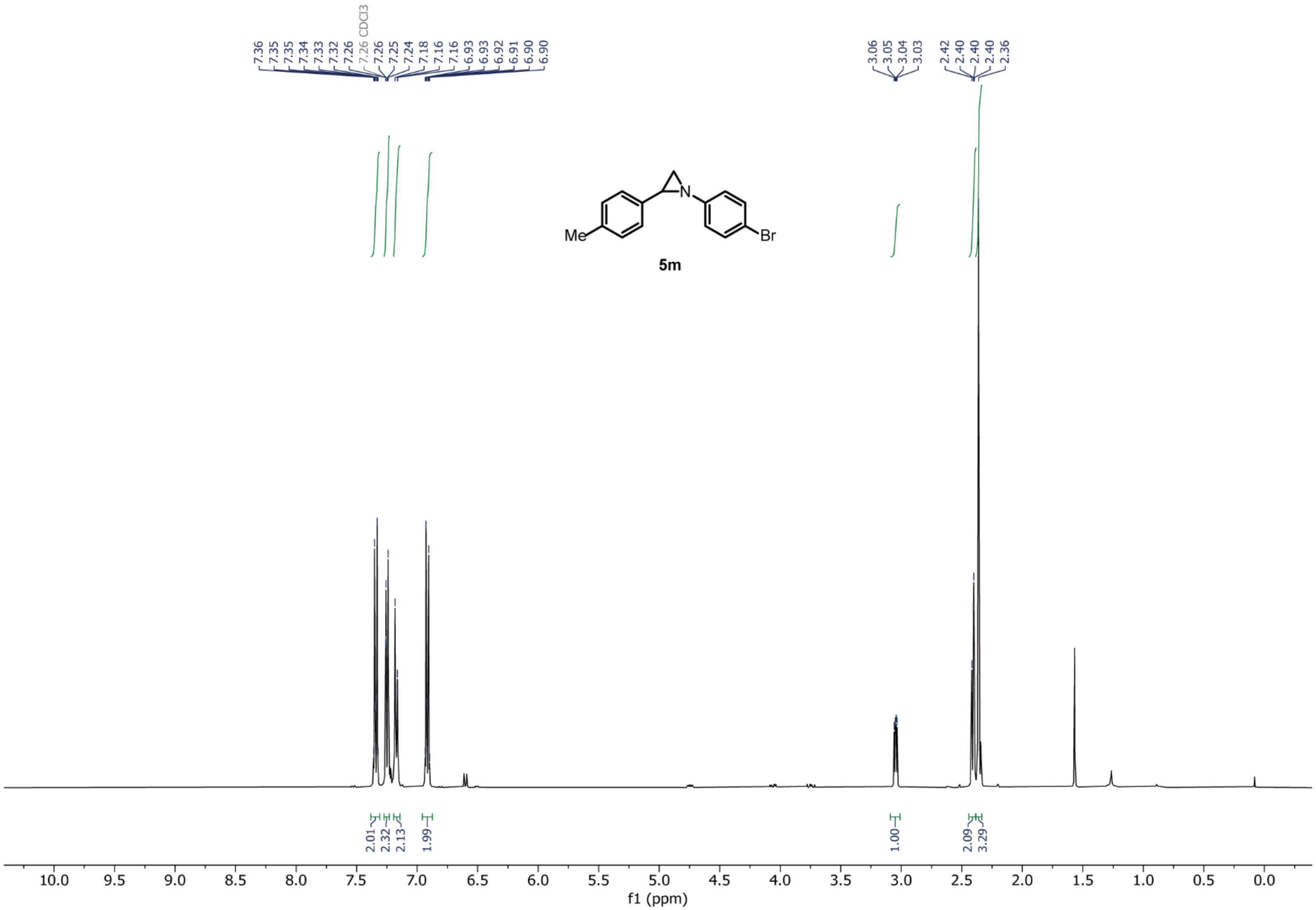

Supplementary Figure 73. ^{1}H NMR spectrum of 1-(4-bromophenyl)-2-(*p*-tolyl)aziridine (**5m**) in $CDCl_3$ (400 MHz) at 23 °C.

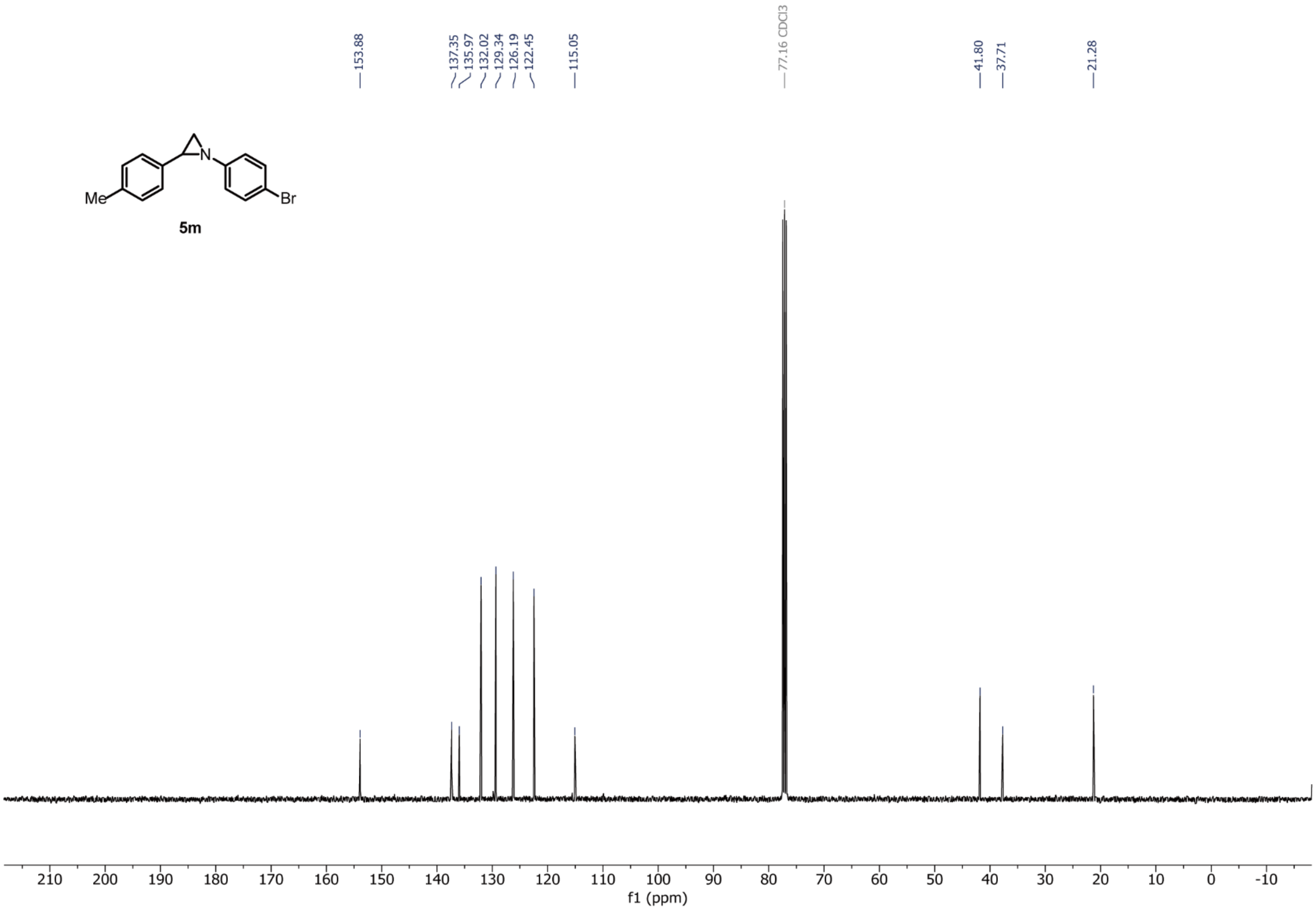

Supplementary Figure 74. ^{13}C NMR spectrum of 1-(4-bromophenyl)-2-(*p*-tolyl)aziridine (**5m**) in $CDCl_3$ (101 MHz) at 23 °C.

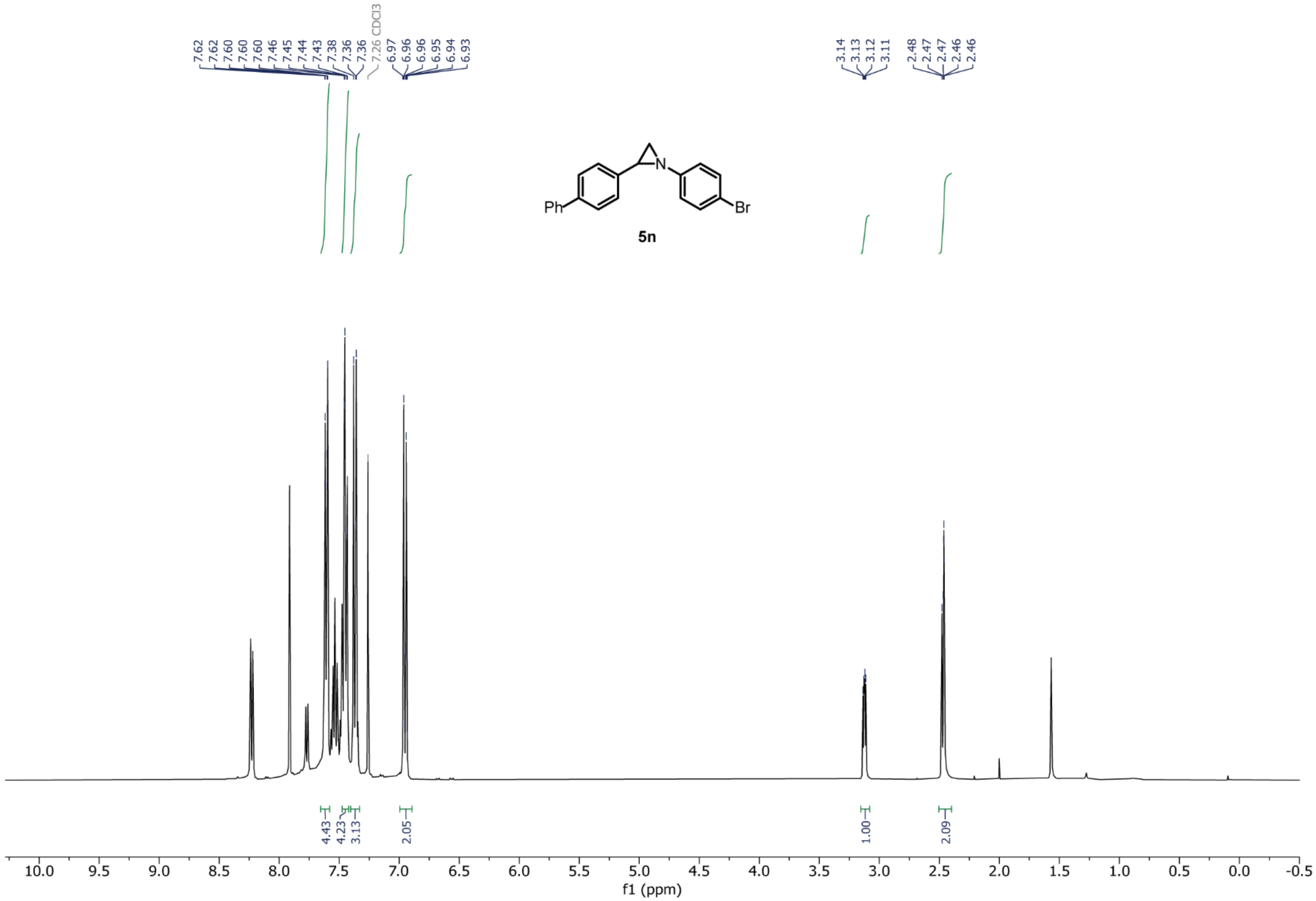

Supplementary Figure 75. ^{1}H NMR spectrum of 2-([1,1'-biphenyl]-4-yl)-1-(4-bromophenyl)aziridine (**5n**) containing 23% 2,4,6-triphenylpyridine in $CDCl_3$ (400 MHz) at 23 °C.

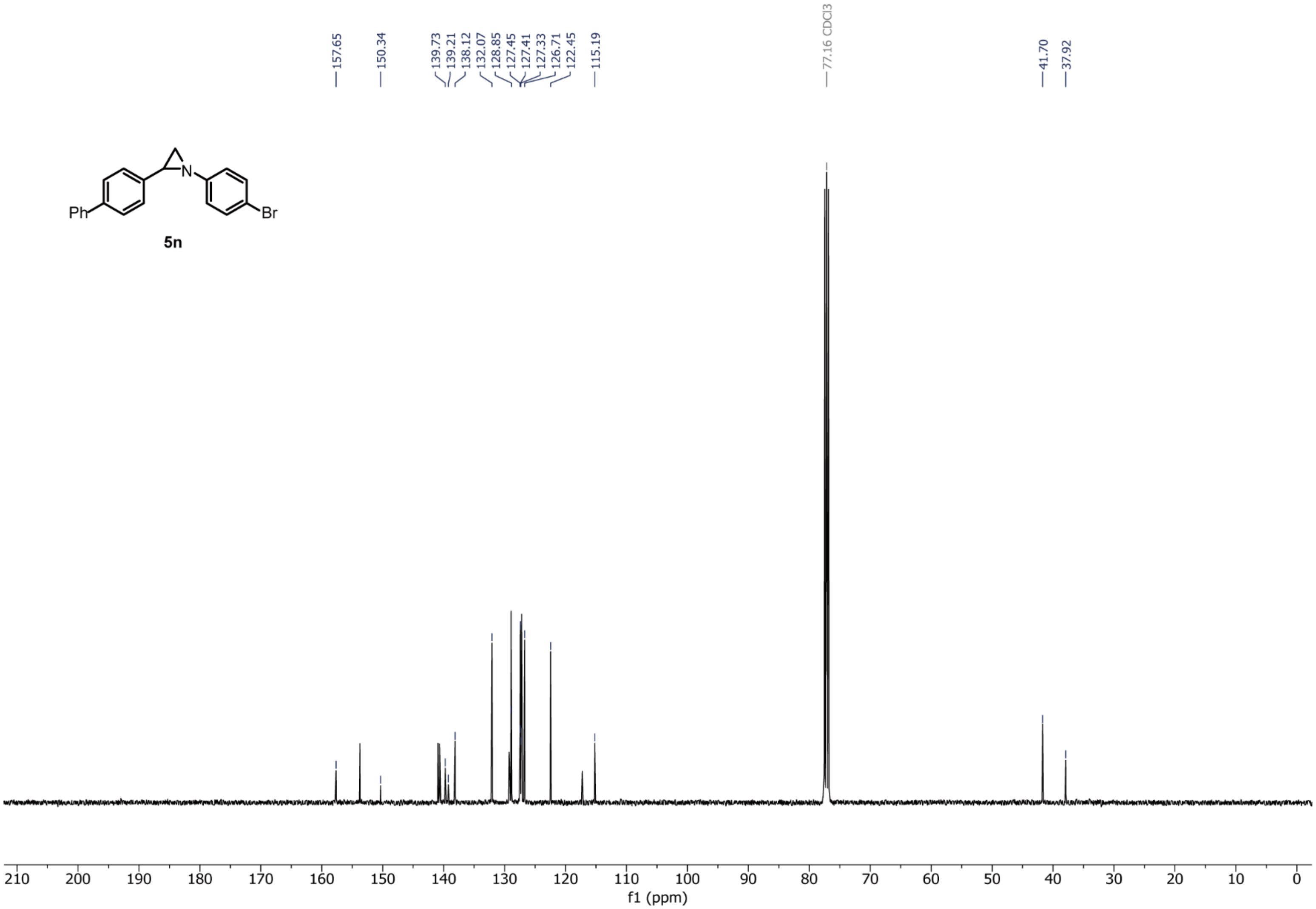

Supplementary Figure 76. ^{13}C NMR spectrum of 2-([1,1'-biphenyl]-4-yl)-1-(4-bromophenyl)aziridine (**5n**) containing 23% 2,4,6-triphenylpyridine in $CDCl_3$ (101 MHz) at 23 °C.

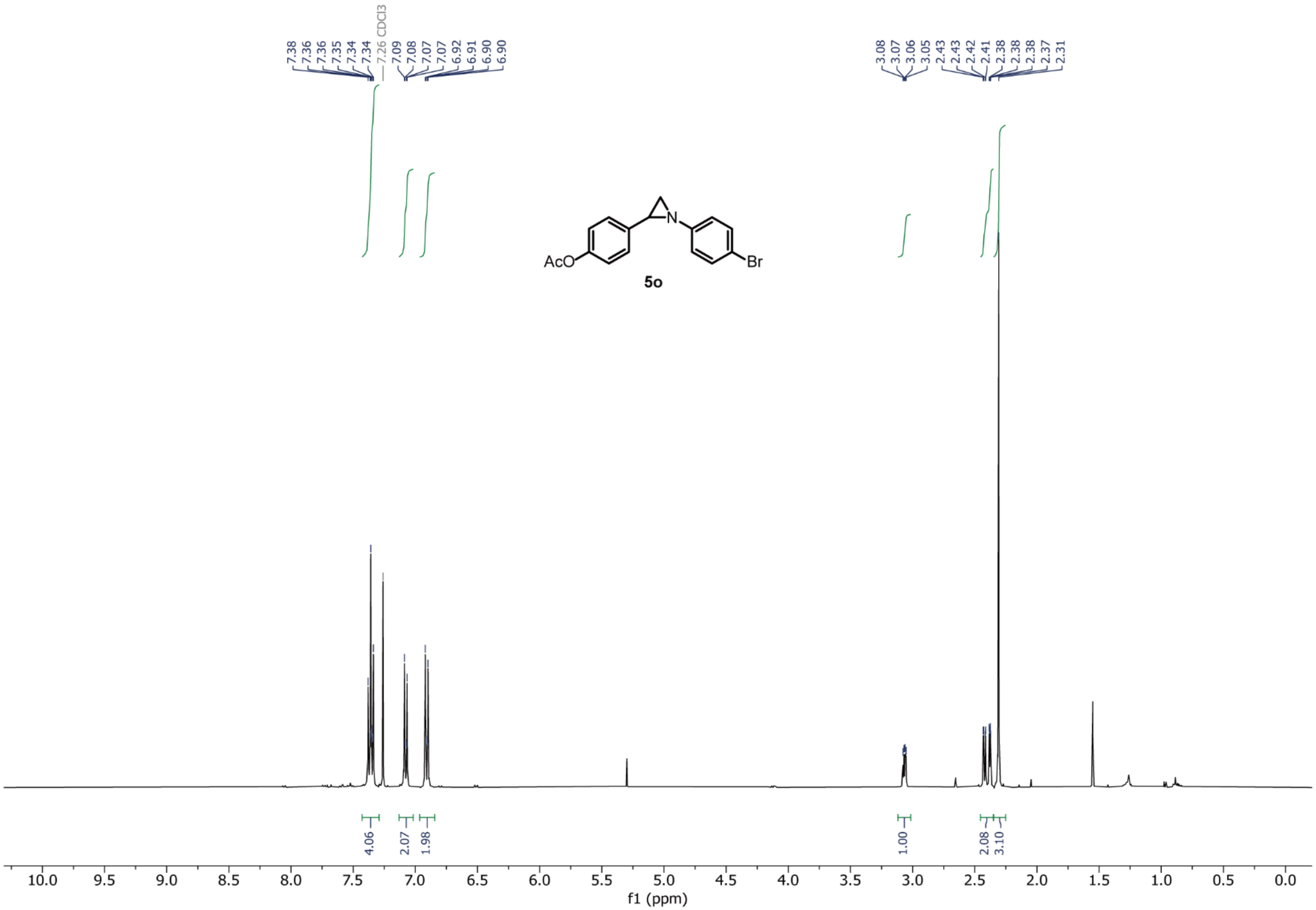

Supplementary Figure 77. ^{1}H NMR spectrum of 4-(1-(4-bromophenyl)aziridin-2-yl)phenyl acetate (**5o**) in $CDCl_3$ (400 MHz) at 23 °C.

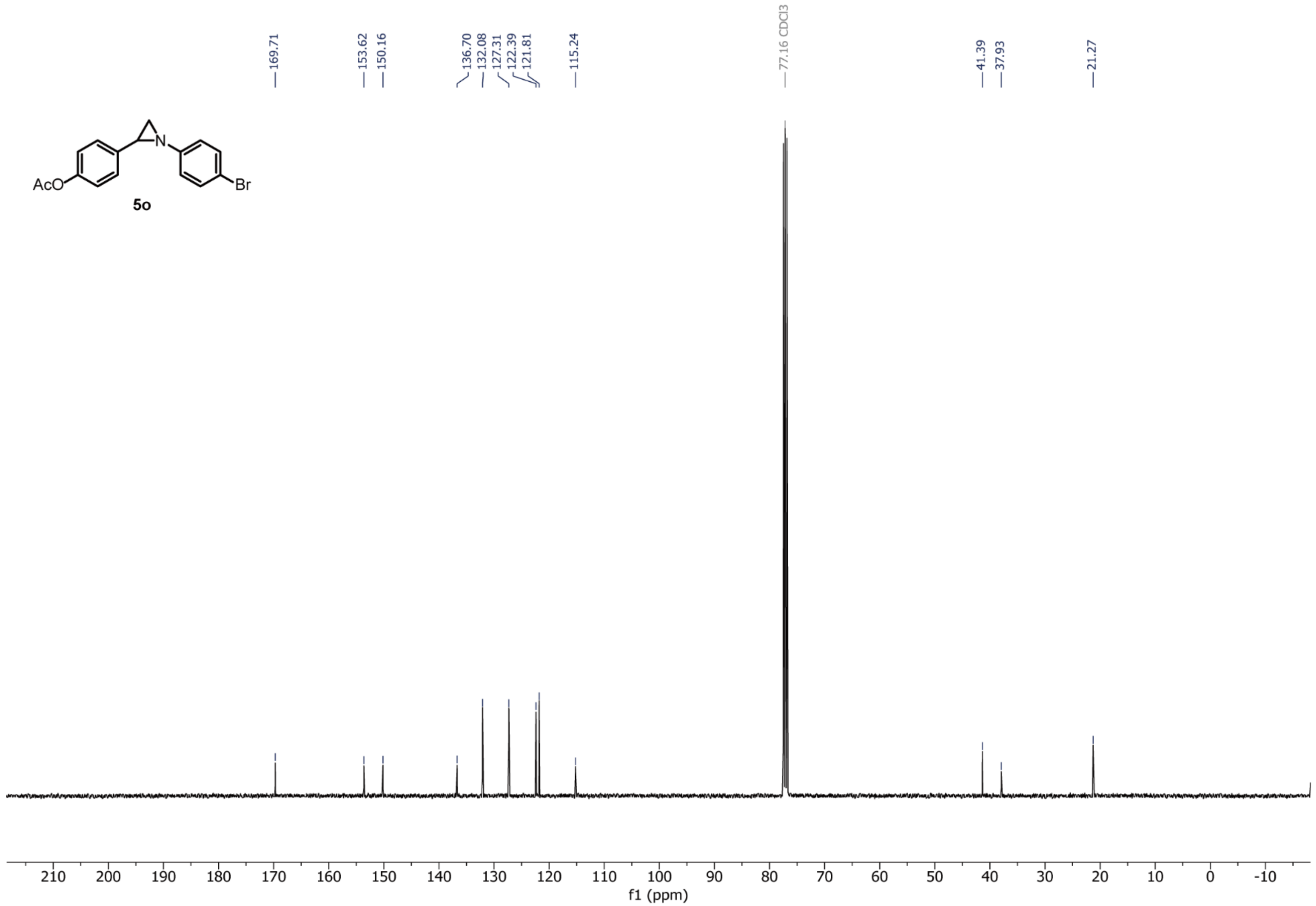

Supplementary Figure 78. ^{13}C NMR spectrum of 4-(1-(4-bromophenyl)aziridin-2-yl)phenyl acetate (**5o**) in $CDCl_3$ (101 MHz) at 23 °C.

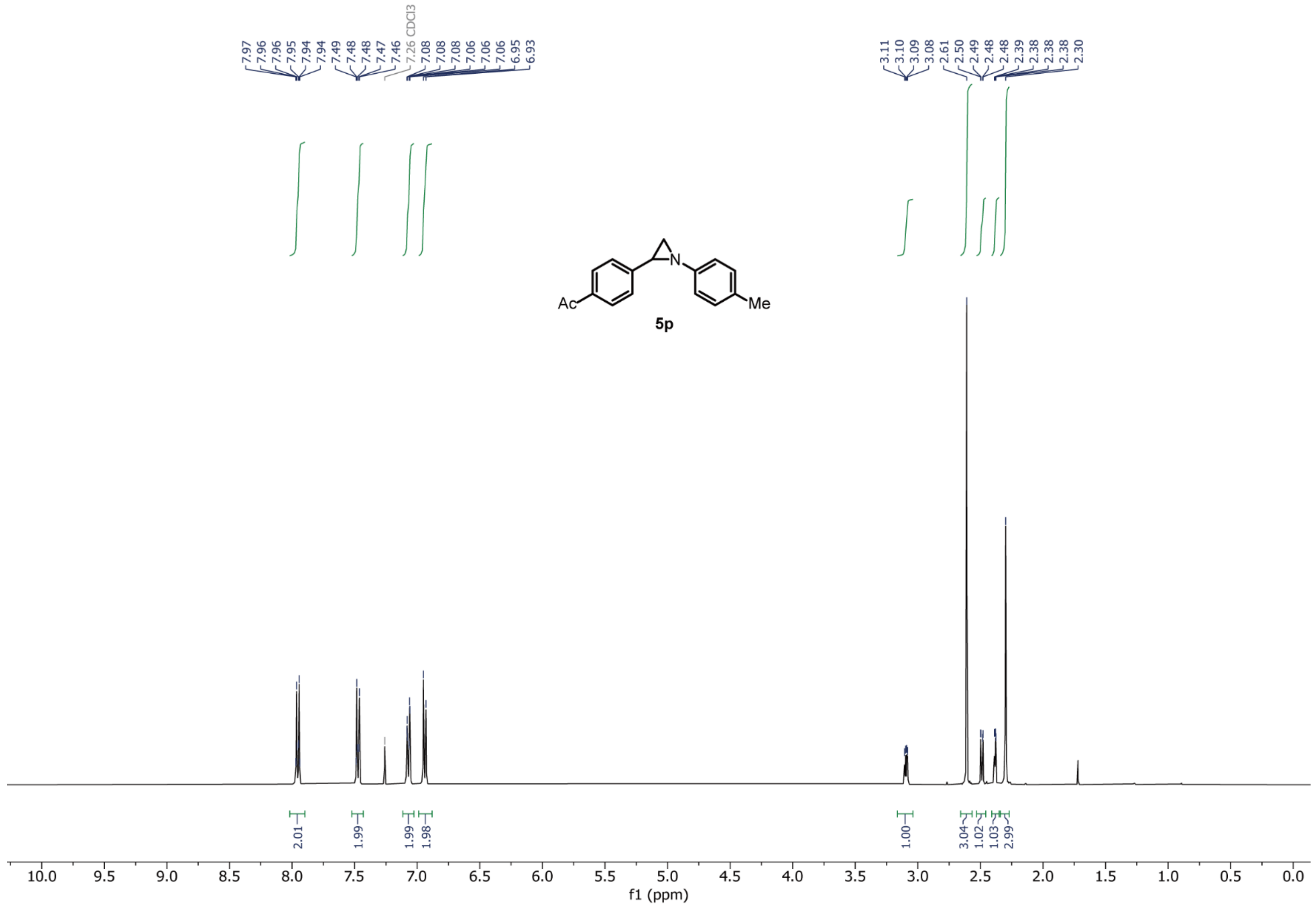

Supplementary Figure 79. ^{1}H NMR spectrum of 1-(4-(1-(*p*-tolyl)aziridin-2-yl)phenyl)ethan-1-one (**5p**) in $CDCl_3$ (400 MHz) at 23 °C.

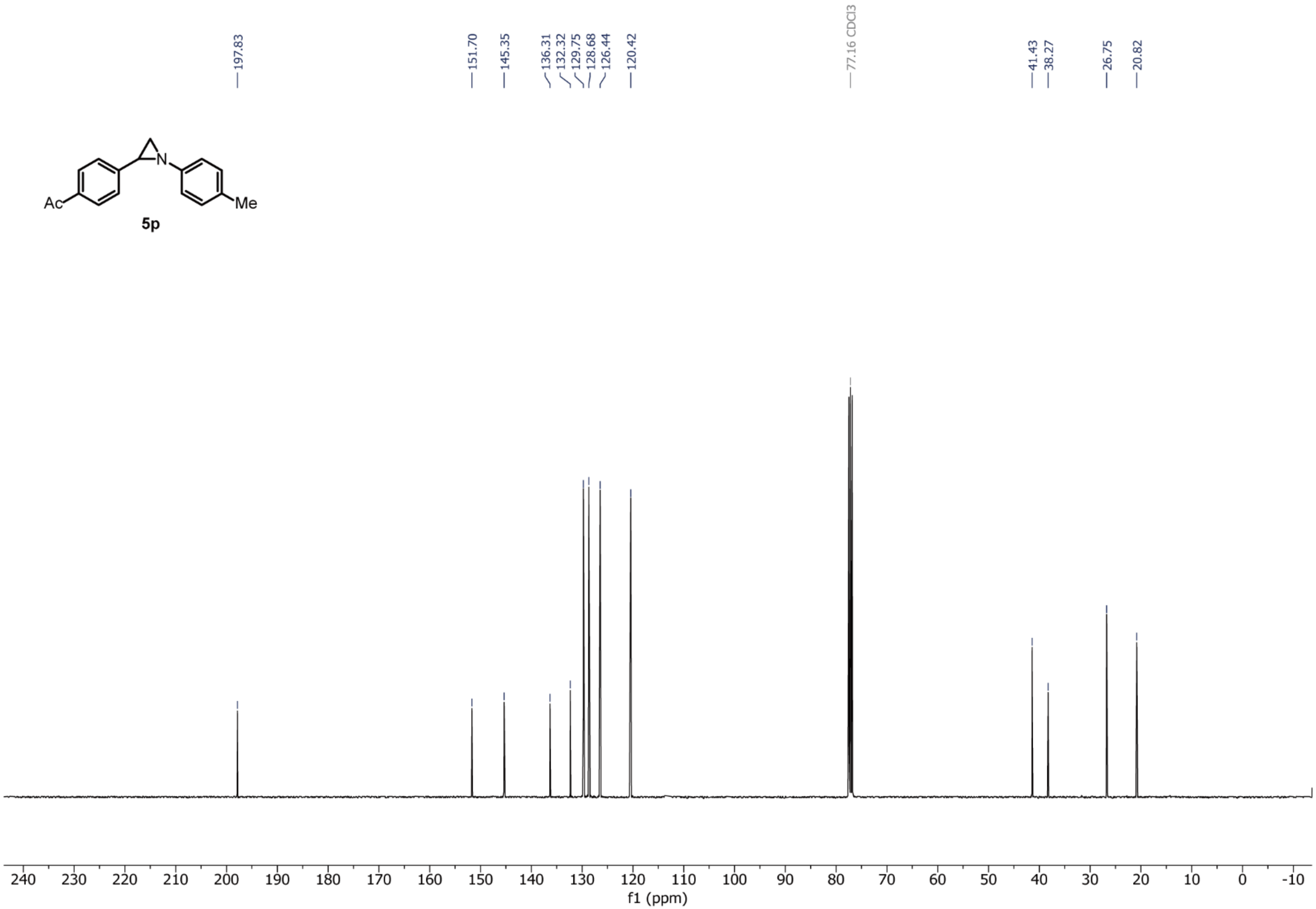

Supplementary Figure 80. ^{13}C NMR spectrum of 1-(4-(1-(*p*-tolyl)aziridin-2-yl)phenyl)ethan-1-one (**5p**) in $CDCl_3$ (101 MHz) at 23 °C.

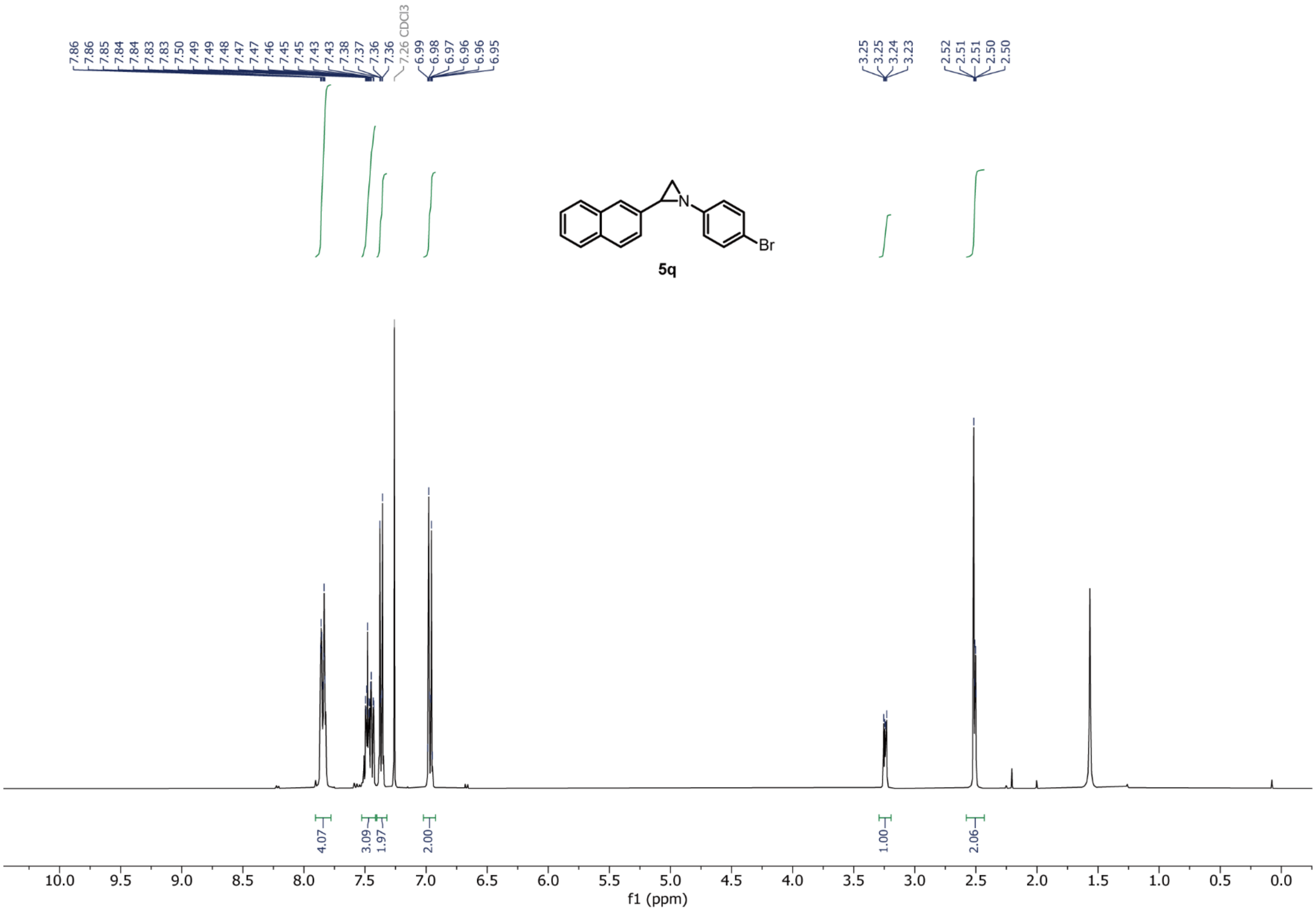

Supplementary Figure 81. ^{1}H NMR spectrum of 1-(4-bromophenyl)-2-(naphthalen-2-yl)aziridine (**5q**) in $CDCl_3$ (400 MHz) at 23 °C.

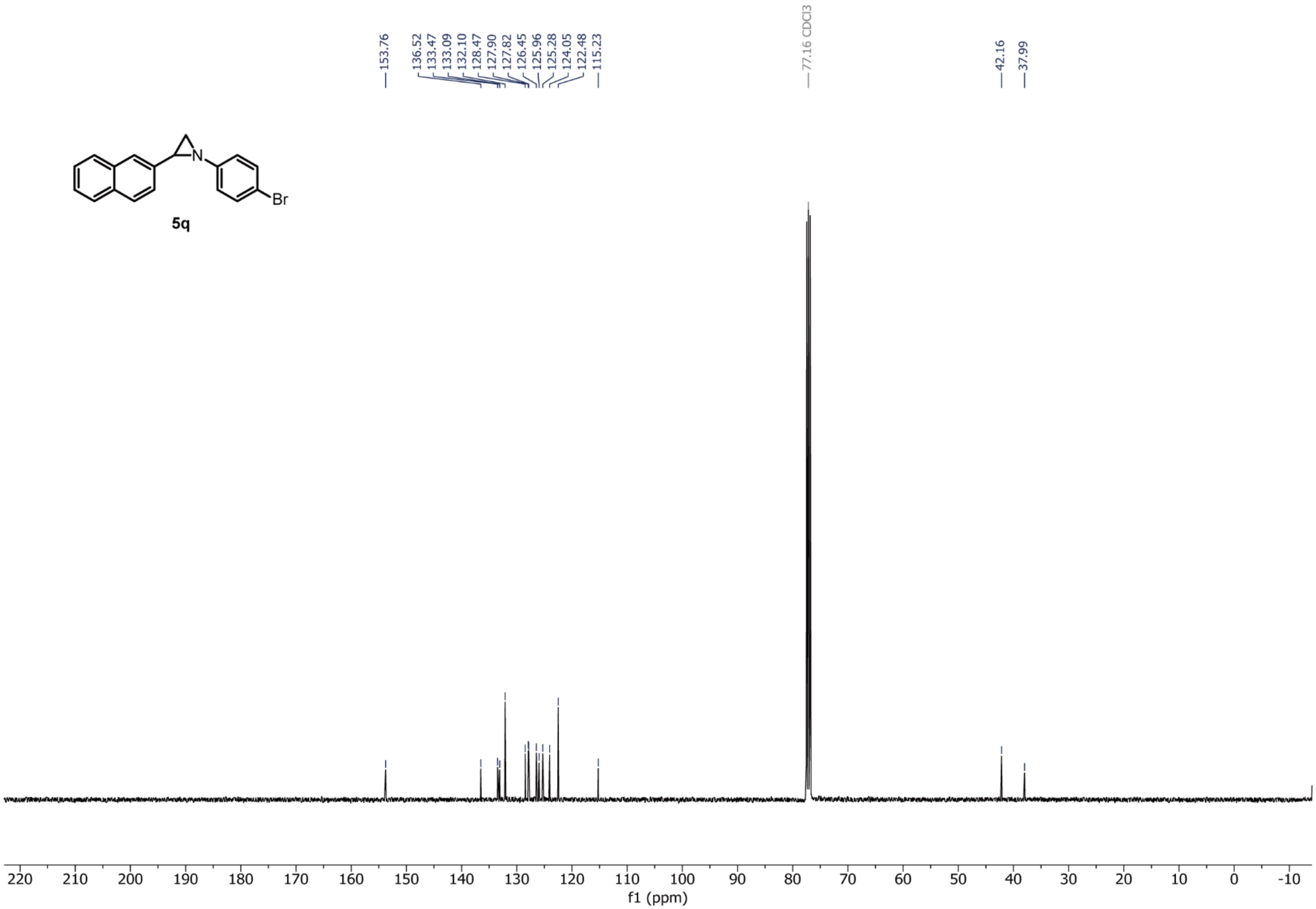

Supplementary Figure 82. ^{13}C NMR spectrum of 1-(4-bromophenyl)-2-(naphthalen-2-yl)aziridine (**5q**) in $CDCl_3$ (101 MHz) at 23 °C.

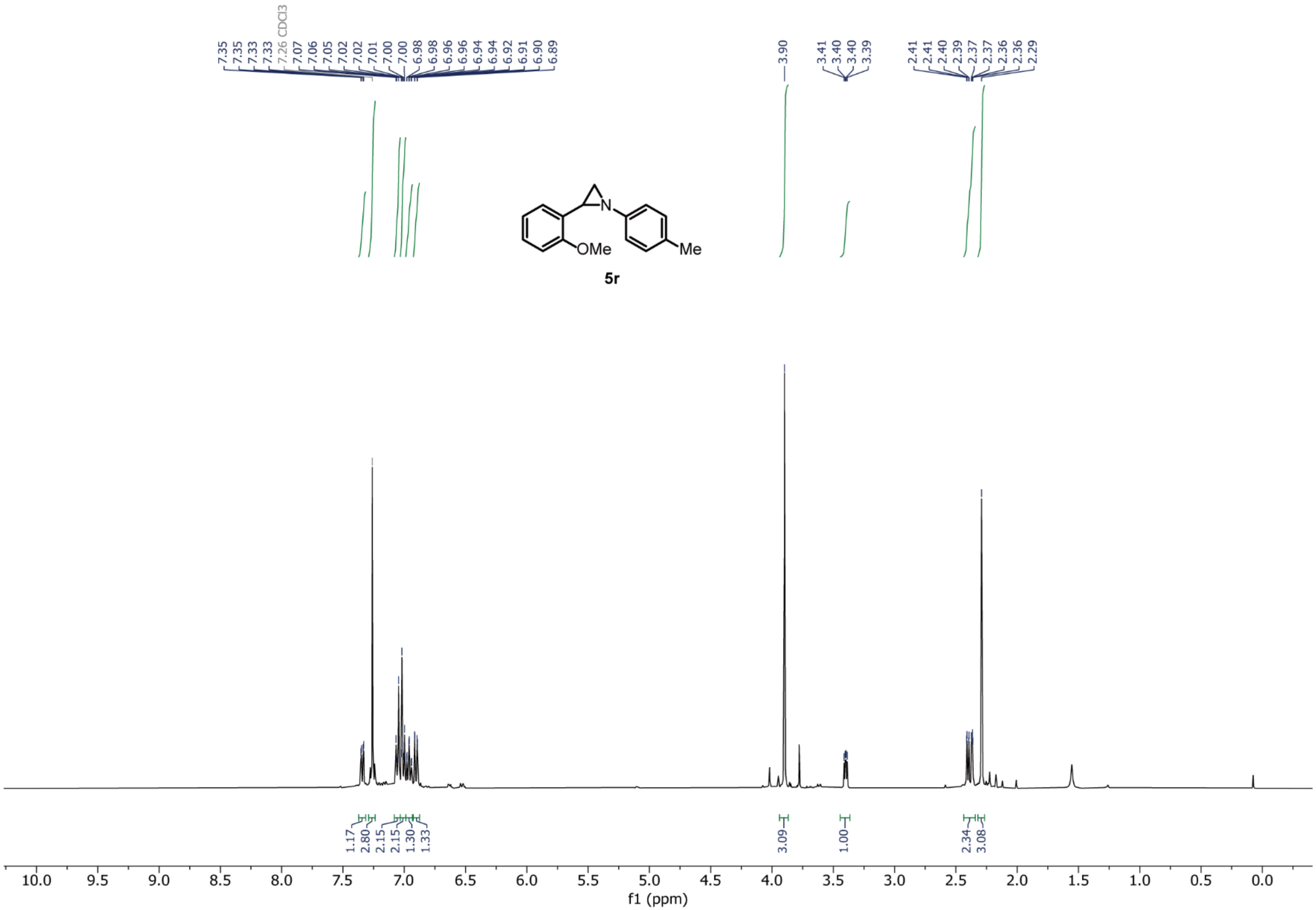

Supplementary Figure 83. ^1H NMR spectrum of 2-(2-methoxyphenyl)-1-(*p*-tolyl)aziridine (**5r**) in $CDCl_3$ (400 MHz) at 23 °C.

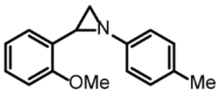

5r

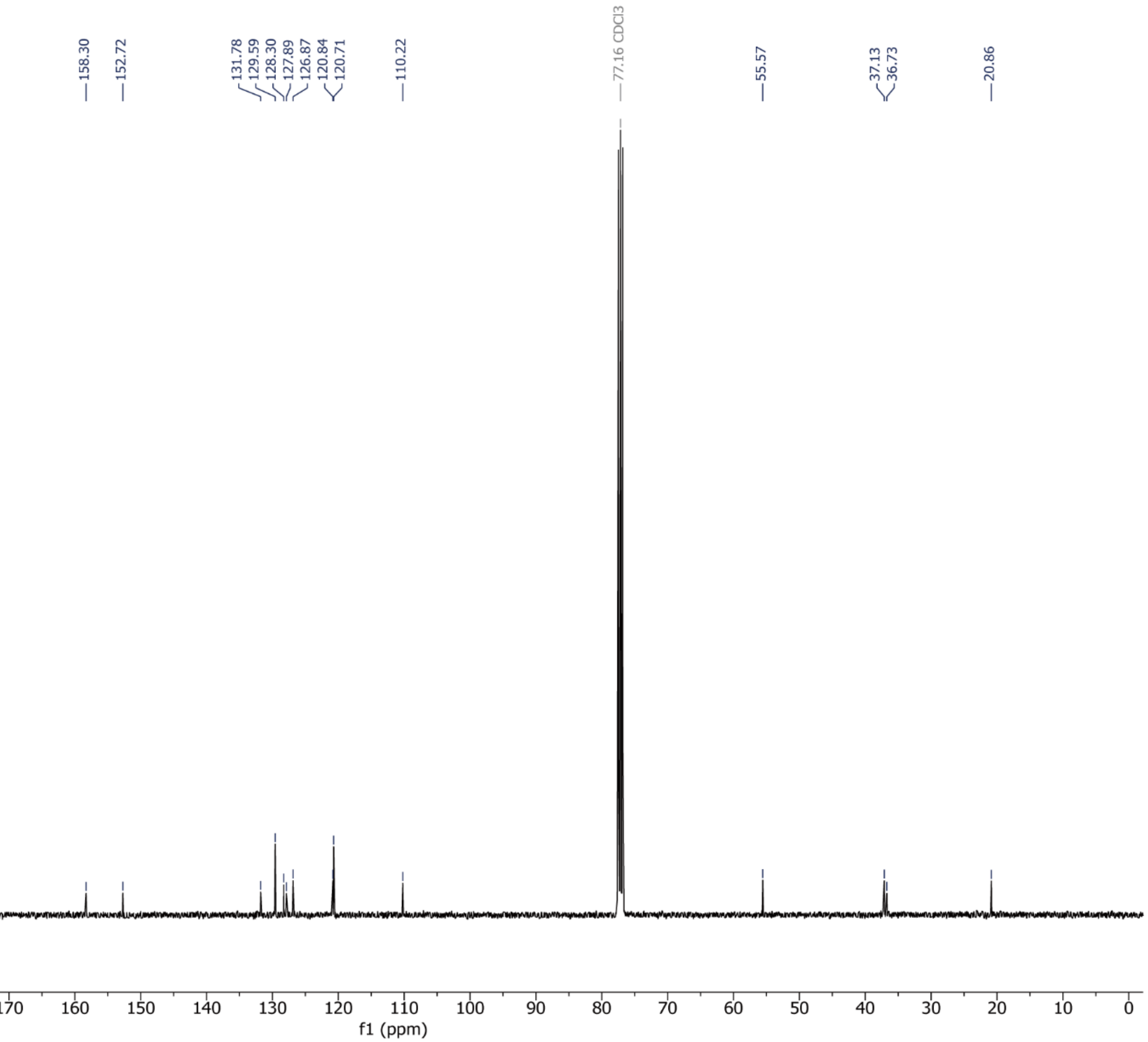

Supplementary Figure 84. ^{13}C NMR spectrum of 2-(2-methoxyphenyl)-1-(*p*-tolyl)aziridine (**5r**) in $CDCl_3$ (101 MHz) at 23 °C.

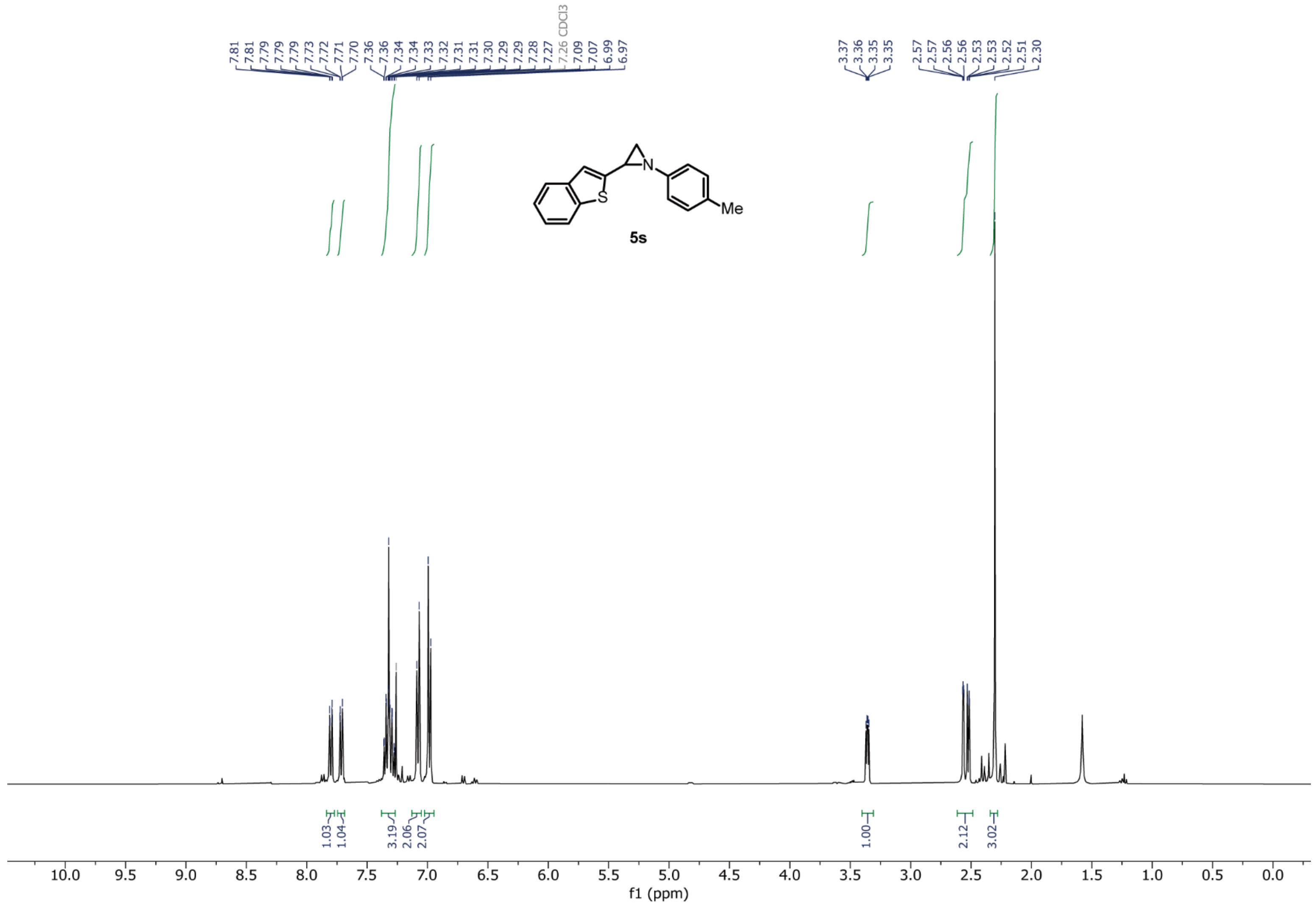

Supplementary Figure 85. ^{1}H NMR spectrum of 2-(benzo[*b*]thiophen-2-yl)-1-(*p*-tolyl)aziridine (**5s**) in $CDCl_3$ (400 MHz) at 23 °C.

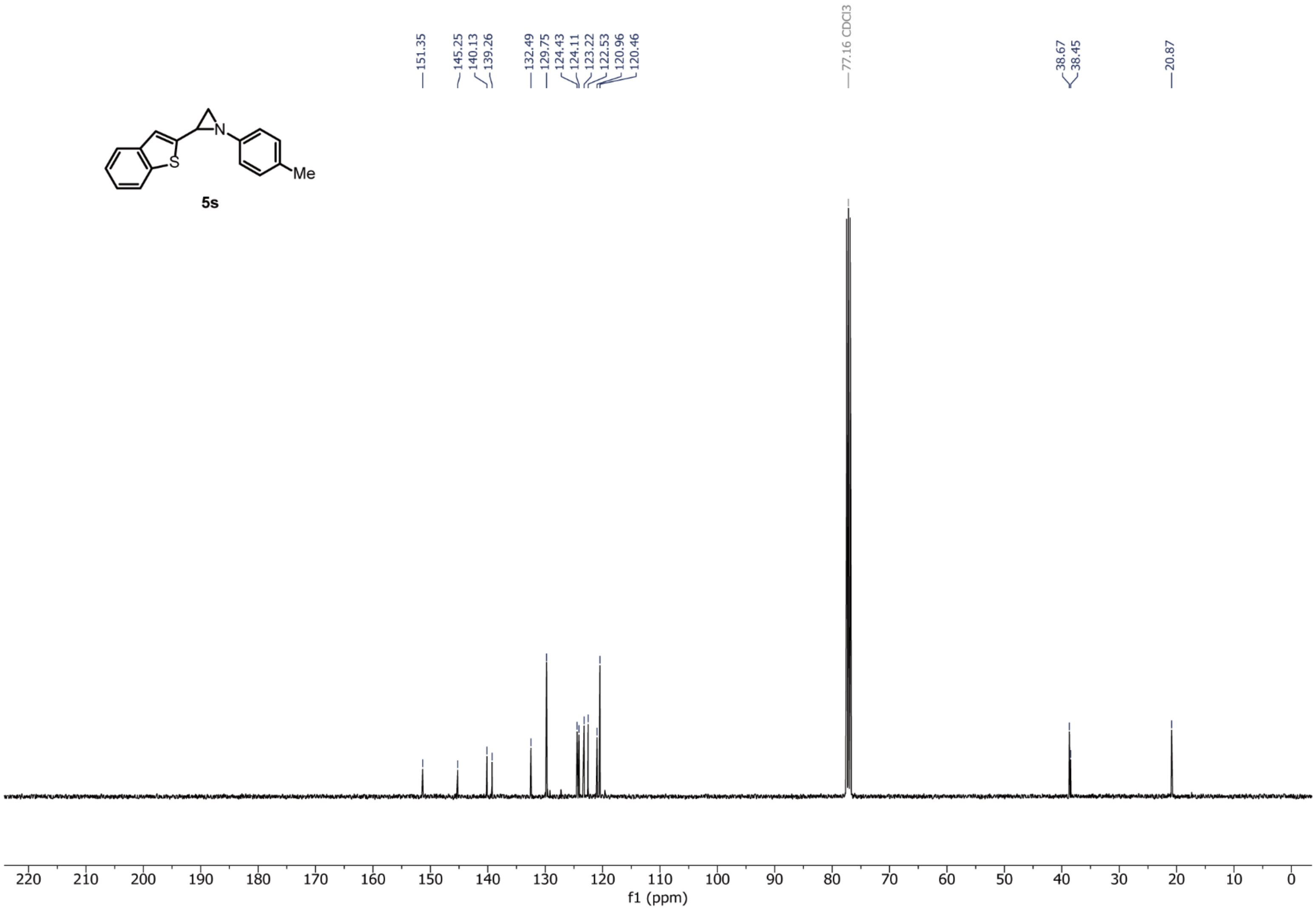

Supplementary Figure 86. ^{13}C NMR spectrum of 2-(benzo[*b*]thiophen-2-yl)-1-(*p*-tolyl)aziridine (**5s**) in $CDCl_3$ (101 MHz) at 23 °C.

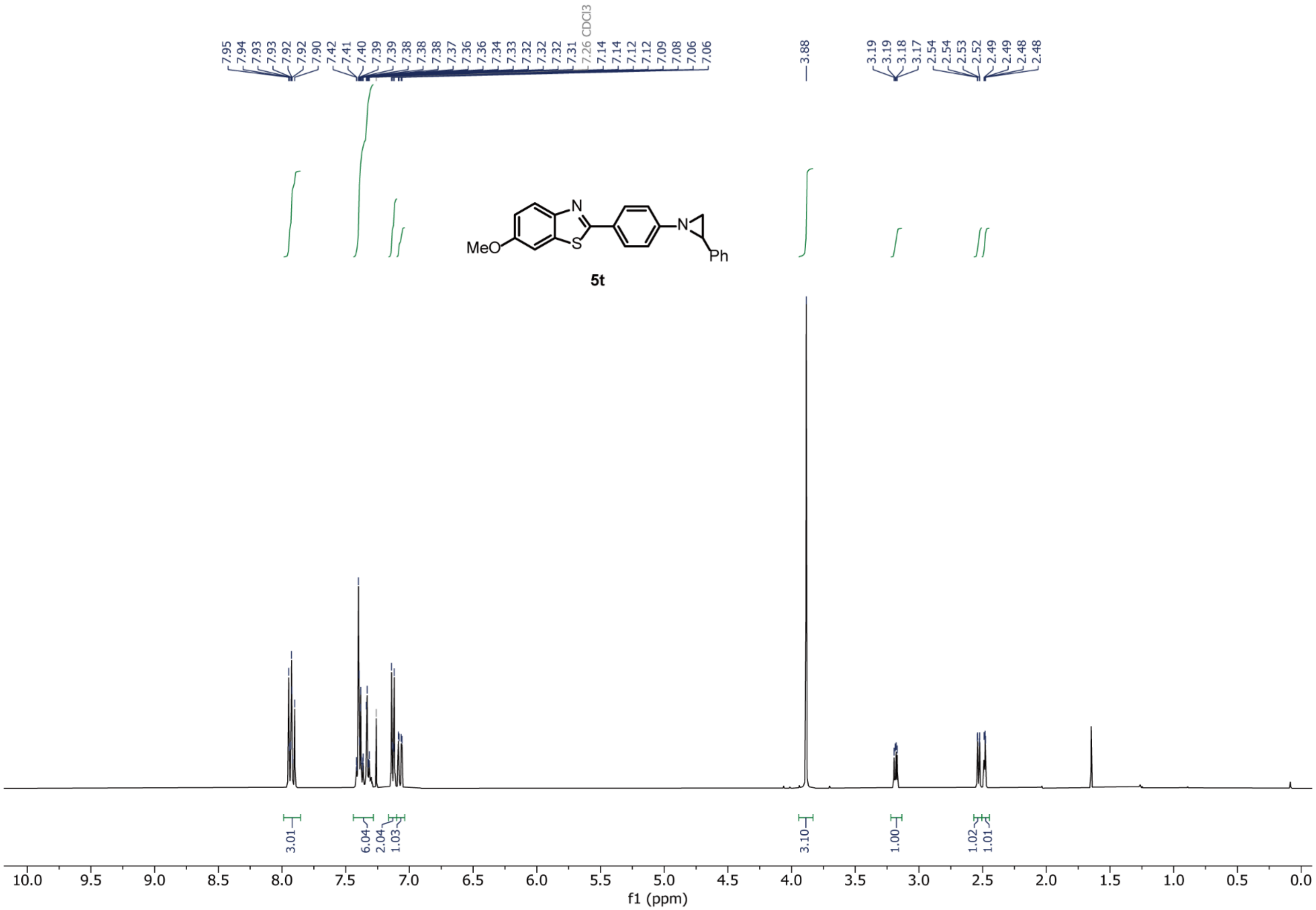

Supplementary Figure 87. ^{1}H NMR spectrum of 6-methoxy-2-(4-(2-phenylaziridin-1-yl)phenyl)benzo[*d*]thiazole (**5t**) in $CDCl_3$ (400 MHz) at 23 °C.

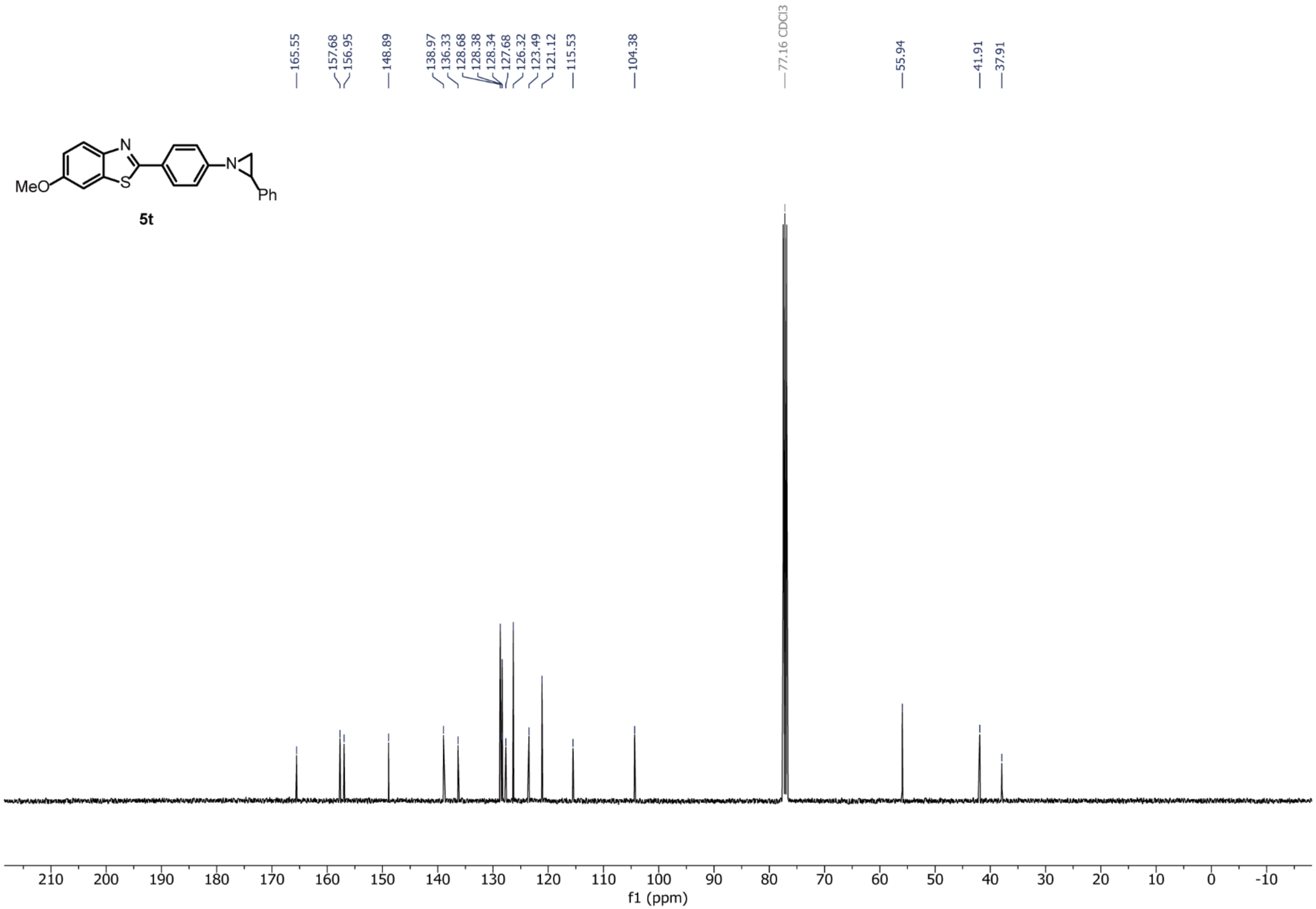

Supplementary Figure 88. ^{13}C NMR spectrum of 6-methoxy-2-(4-(2-phenylaziridin-1-yl)phenyl)benzo[*d*]thiazole (**5t**) in $CDCl_3$ (101 MHz) at 23 °C.

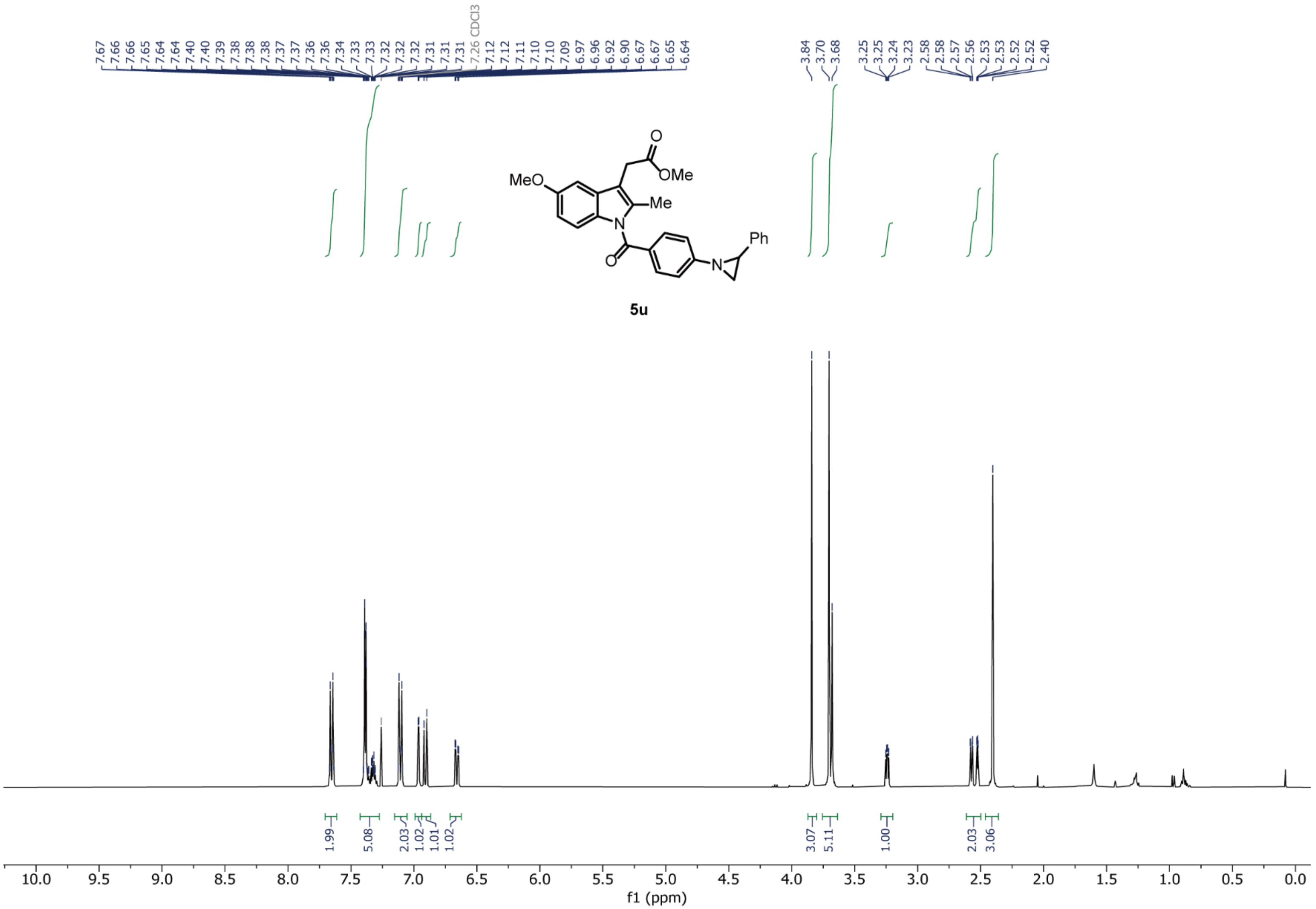

Supplementary Figure 89. ^{1}H NMR spectrum of methyl 2-(5-methoxy-2-methyl-1-(4-(2-phenylaziridin-1-yl)benzoyl)-1*H*-indol-3-yl)acetate (**5u**) in $CDCl_3$ (400 MHz) at 23 °C.

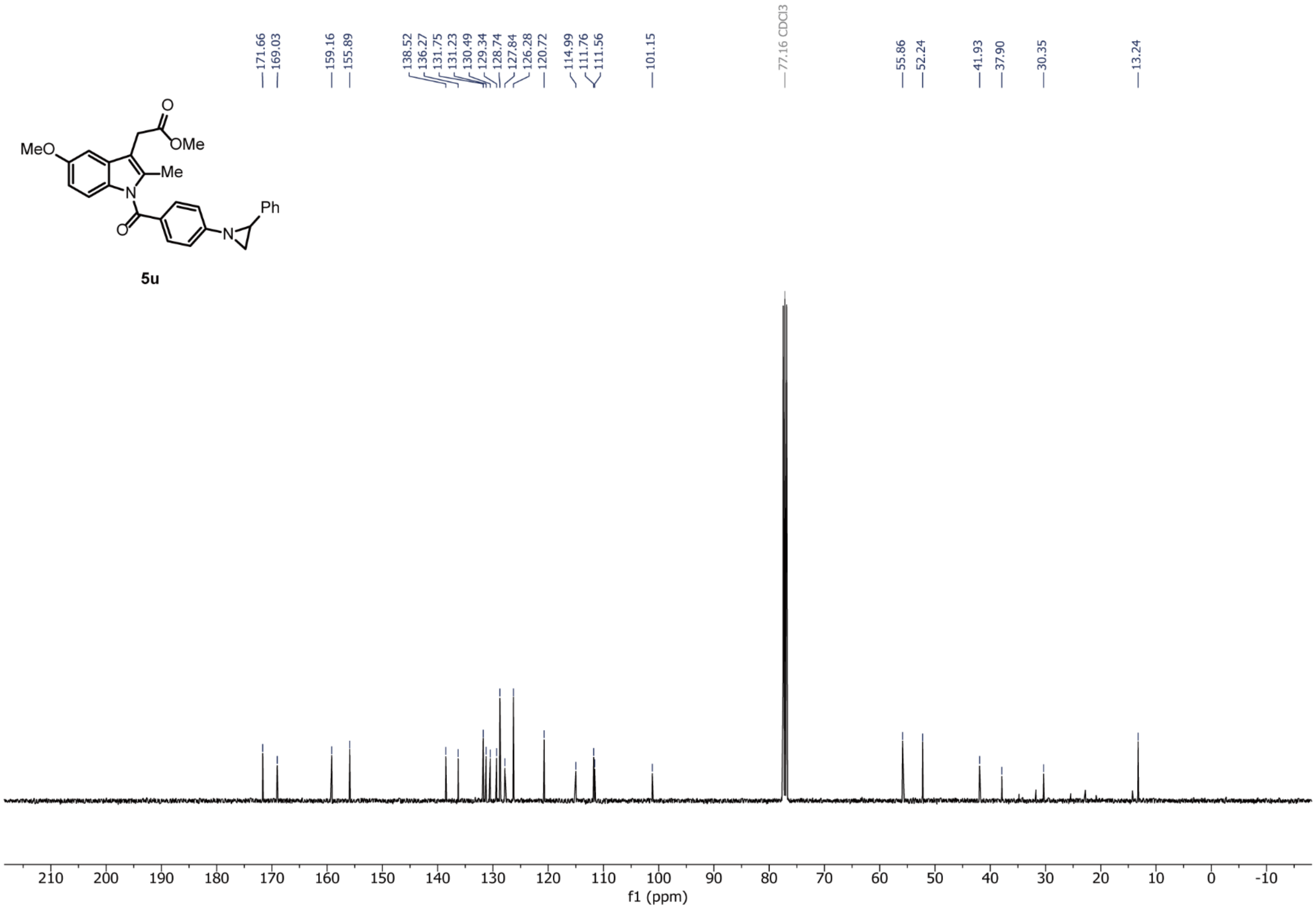

5u

Supplementary Figure 90. ^{13}C NMR spectrum of methyl 2-(5-methoxy-2-methyl-1-(4-(2-phenylaziridin-1-yl)benzoyl)-1*H*-indol-3-yl)acetate (**5u**) in $CDCl_3$ (101 MHz) at 23 °C.

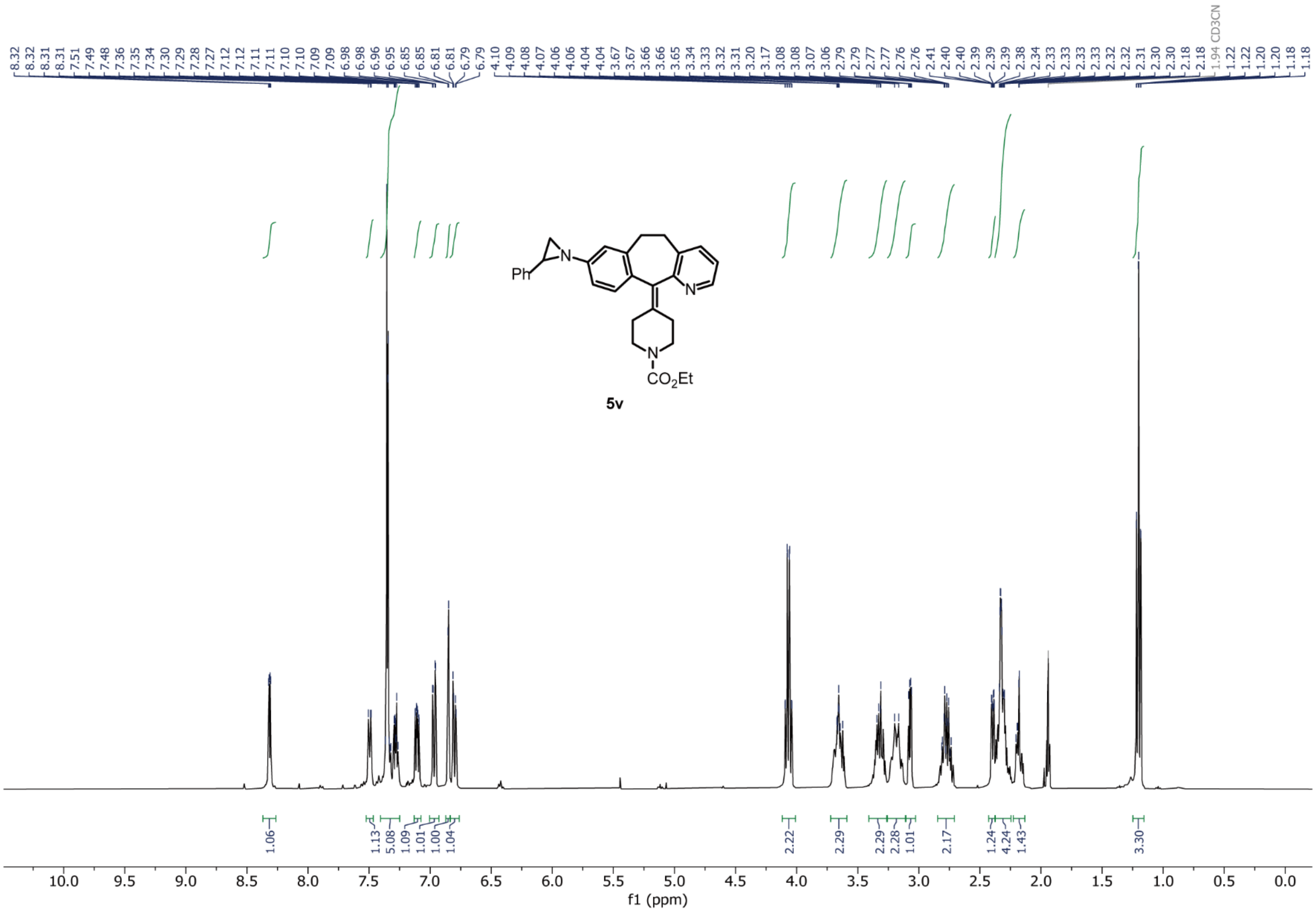

Supplementary Figure 91. ^1H NMR spectrum of ethyl 4-(8-(2-phenylaziridin-1-yl)-5,6-dihydro-11*H*-benzo[5,6]cyclohepta[1,2-*b*]pyridin-11-ylidene)piperidine-1-carboxylate (**5v**) in $CDCl_3$ (400 MHz) at 23 °C.

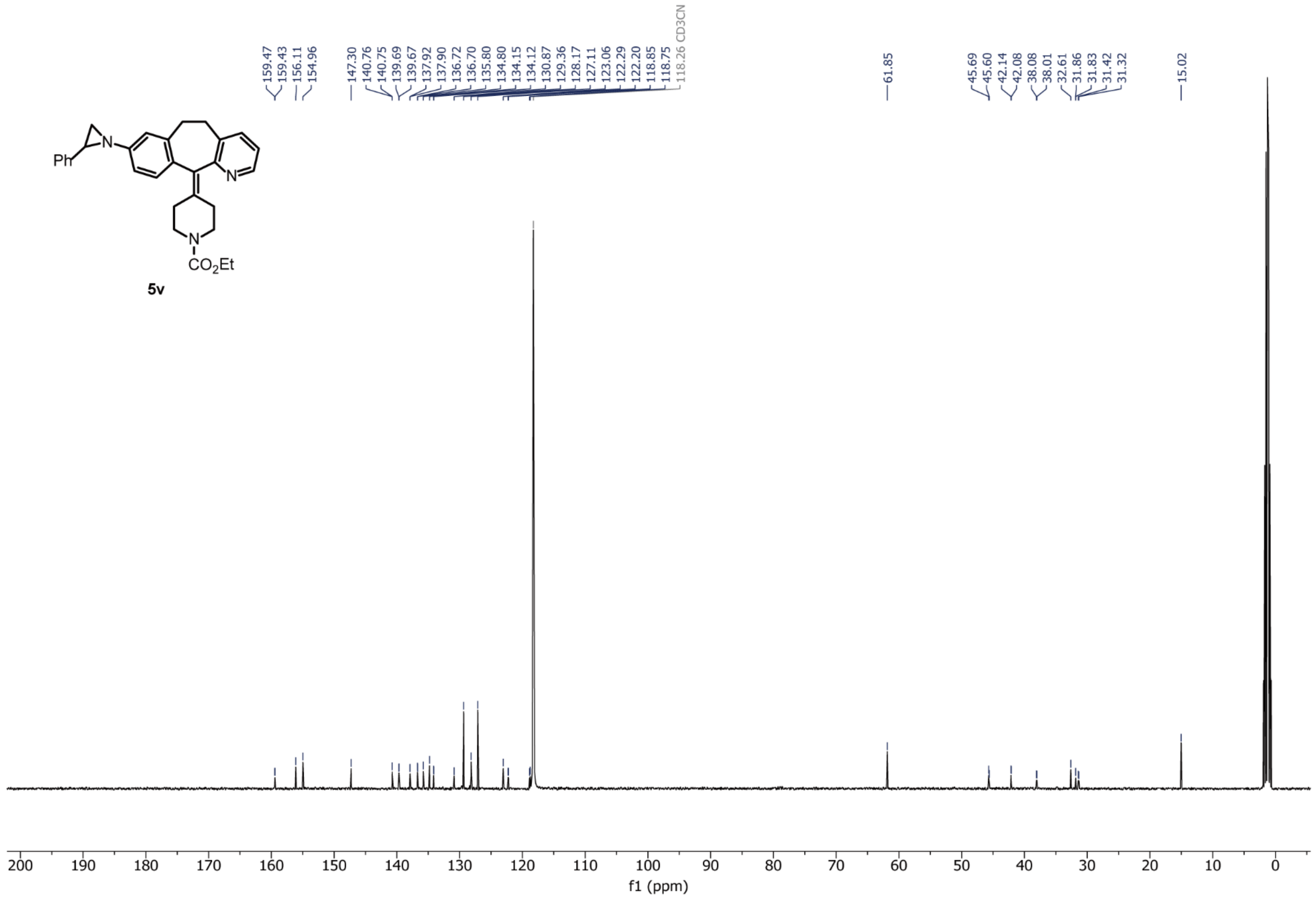

Supplementary Figure 92. ^{13}C NMR spectrum of ethyl 4-(8-(2-phenylaziridin-1-yl)-5,6-dihydro-11*H*-benzo[5,6]cyclohepta[1,2-*b*]pyridin-11-ylidene)piperidine-1-carboxylate (**5v**) in $CDCl_3$ (101 MHz) at 23 °C.

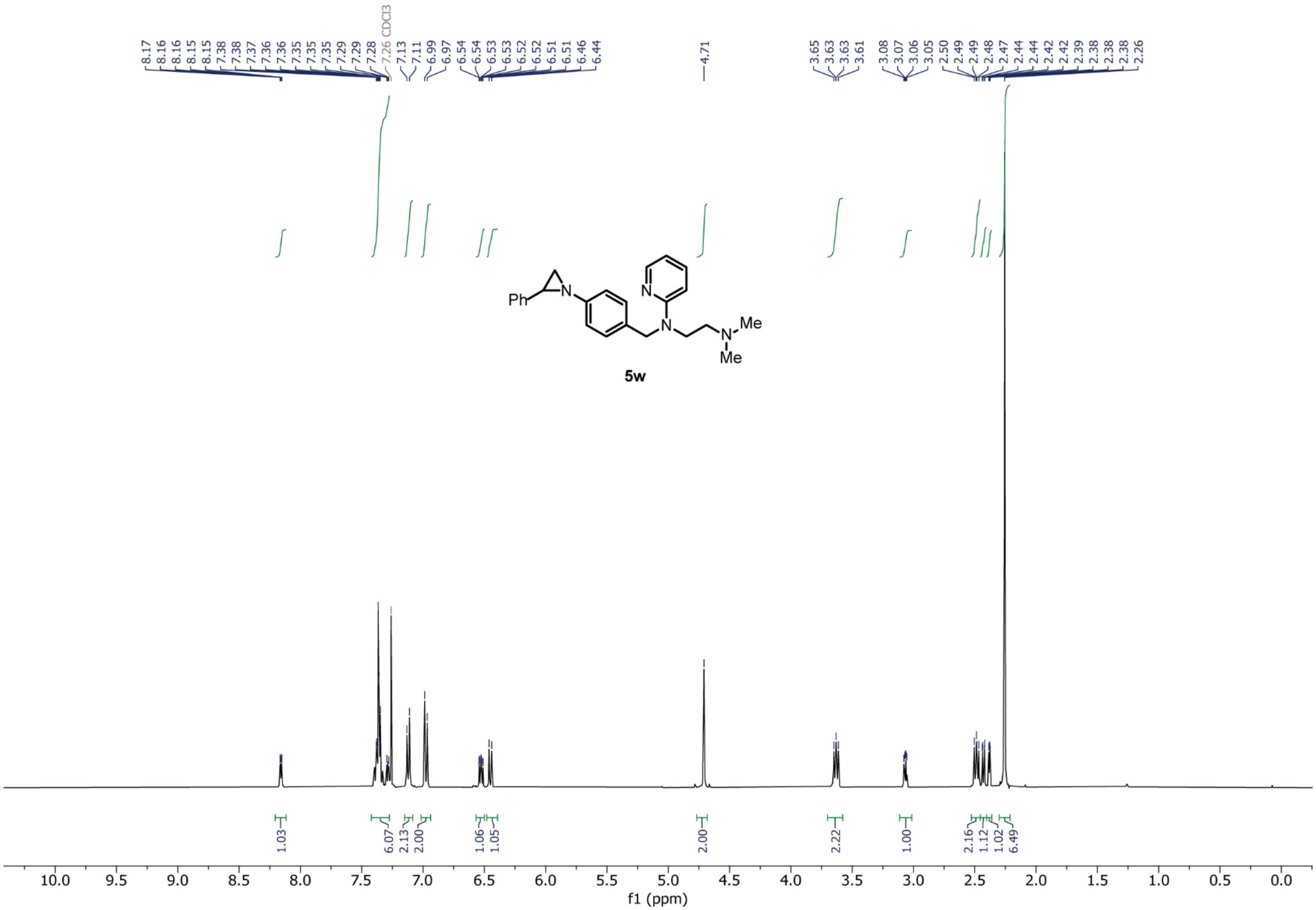

Supplementary Figure 93. ^{1}H NMR spectrum of *N*[1],*N*[1]-dimethyl-*N*[2]-(4-(2-phenylaziridin-1-yl)benzyl)-*N*[2]-(pyridin-2-yl)ethane-1,2-diamine (**5w**) in $CDCl_3$ (400 MHz) at 23 °C.

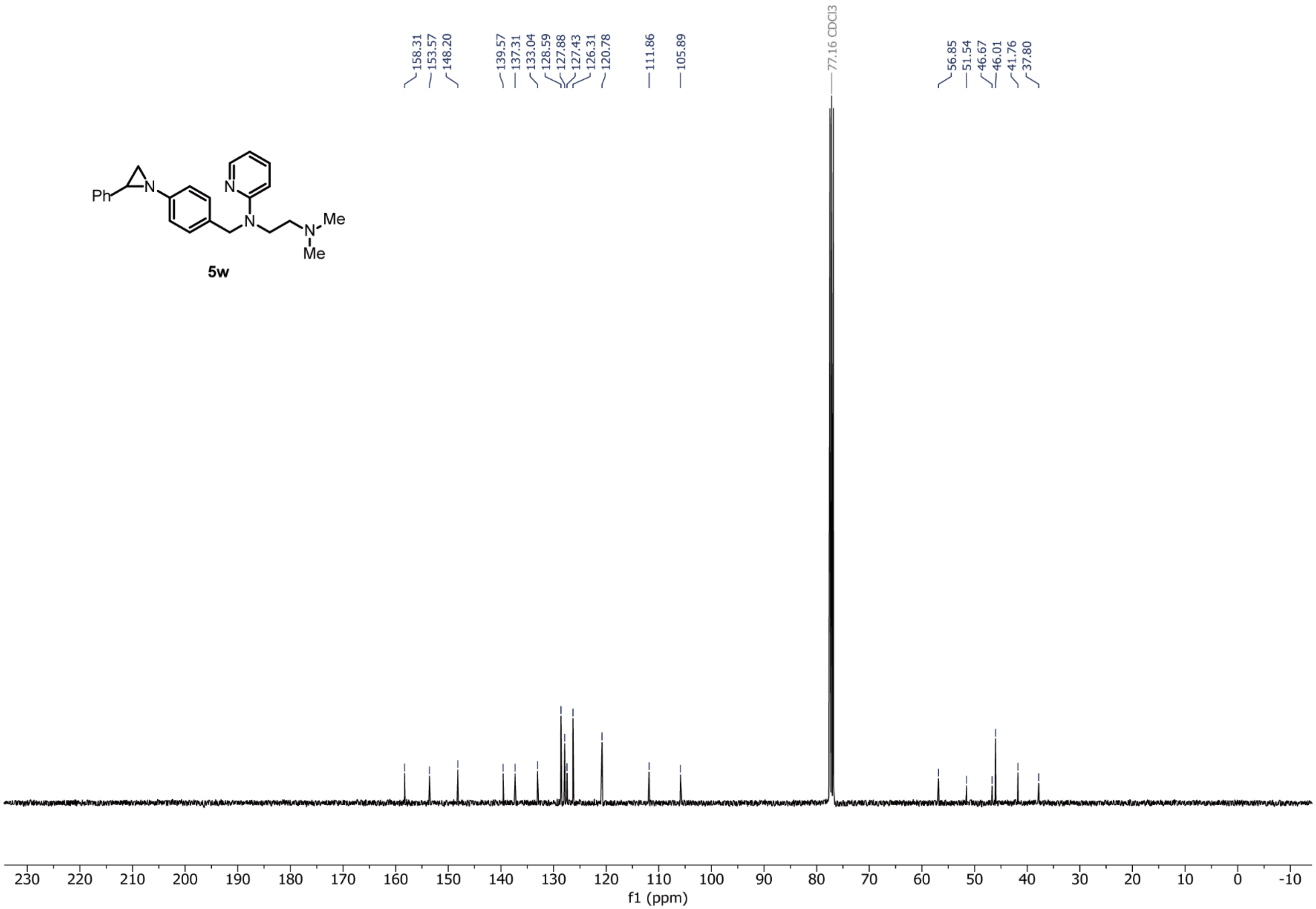

Supplementary Figure 94. ^{13}C NMR spectrum of N^1,N^1-dimethyl-N^2-(4-(2-phenylaziridin-1-yl)benzyl)-N^2-(pyridin-2-yl)ethane-1,2-diamine (**5w**) in $CDCl_3$ (101 MHz) at 23 °C.

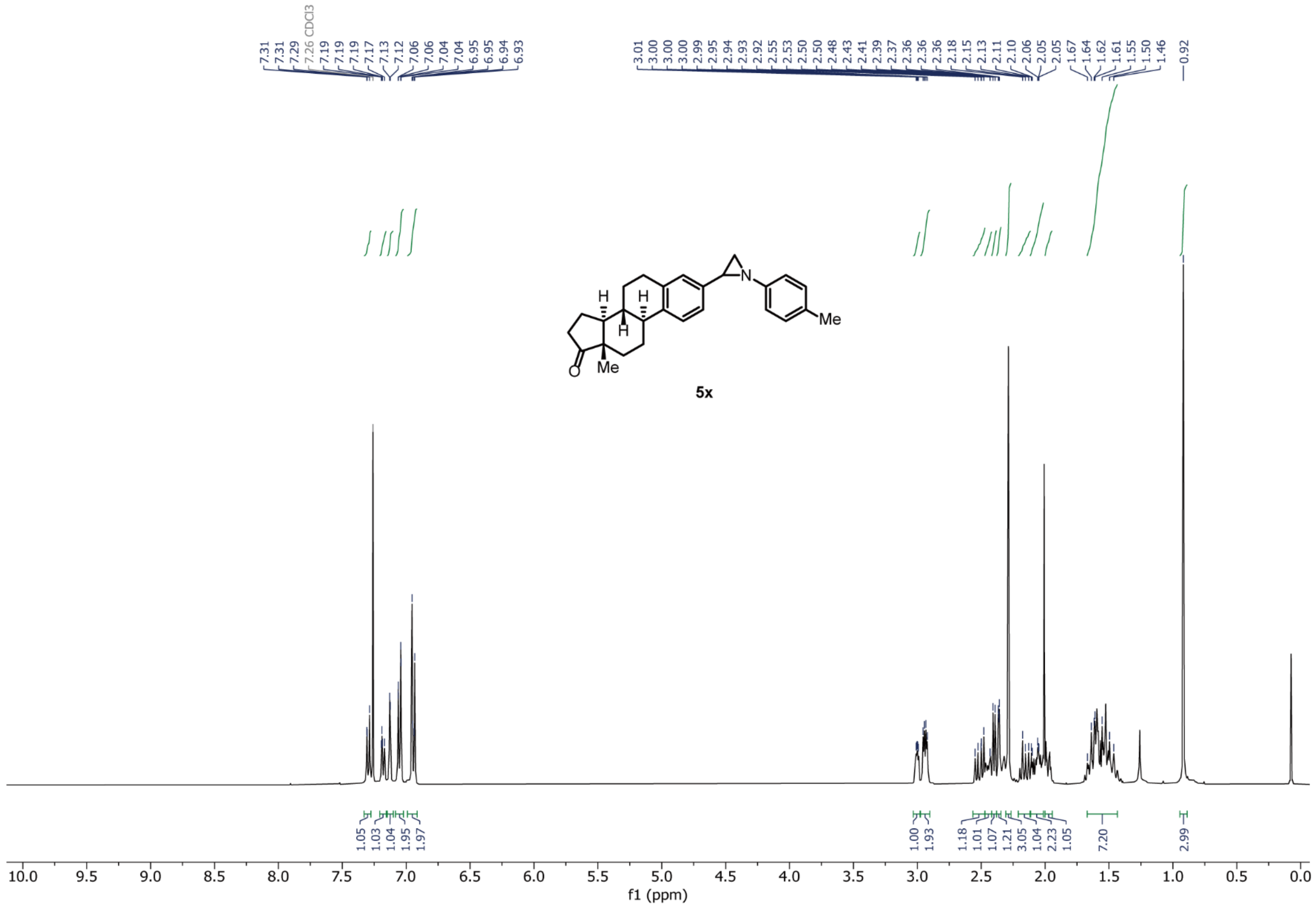

Supplementary Figure 95. ^{1}H NMR spectrum of (8*R*,9*S*,13*S*,14*S*)-13-methyl-3-(1-(*p*-tolyl)aziridin-2-yl)-6,7,8,9,11,12,13,14,15,16-decahydro-17*H*-cyclopenta[*a*]phenanthren-17-one (**5x**) in $CDCl_3$ (400 MHz) at 23 °C.

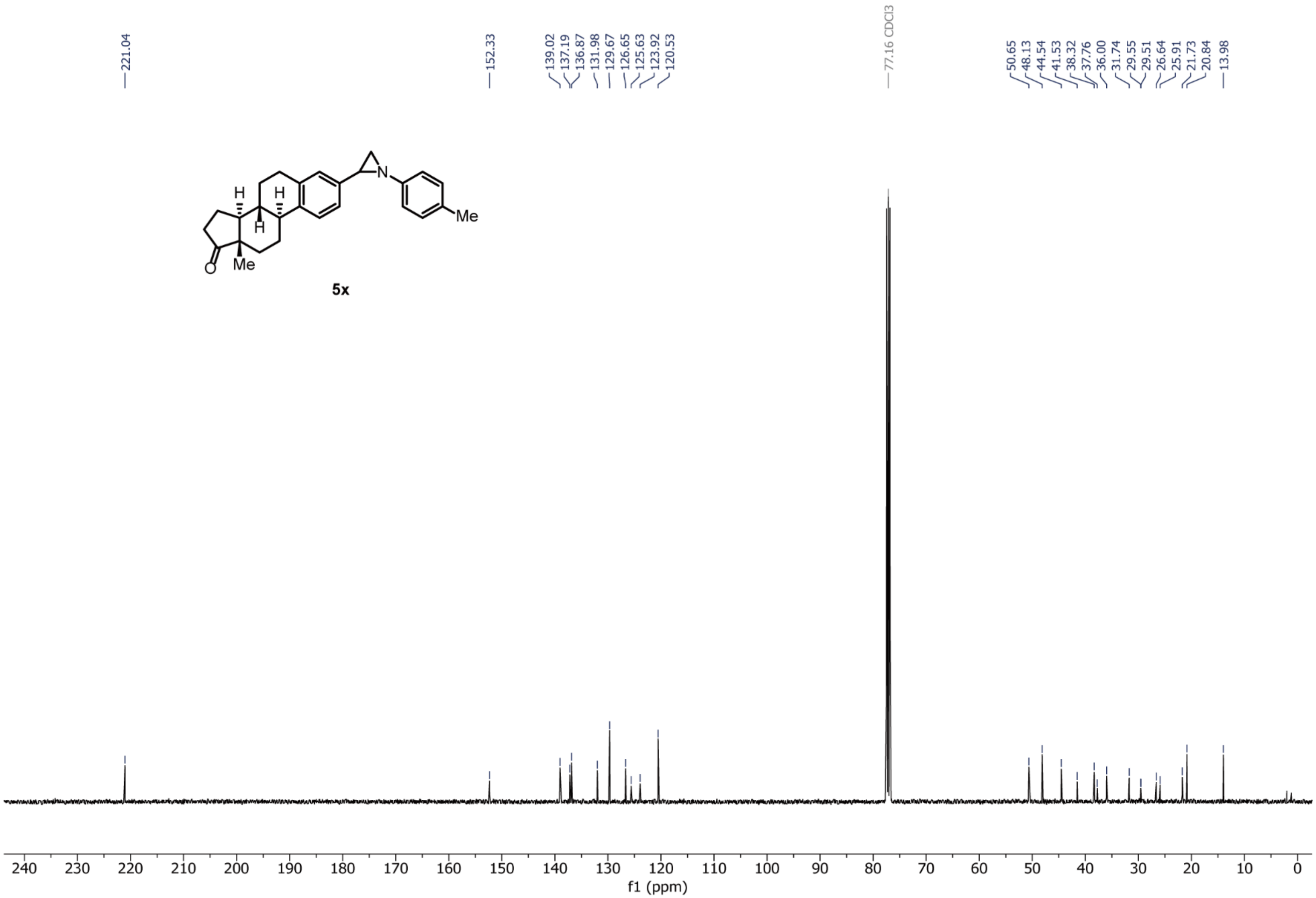

Supplementary Figure 96. ^{13}C NMR spectrum of (8*R*,9*S*,13*S*,14*S*)-13-methyl-3-(1-(*p*-tolyl)aziridin-2-yl)-6,7,8,9,11,12,13,14,15,16-decahydro-17*H*-cyclopenta[*a*]phenanthren-17-one (**5x**) in $CDCl_3$ (101 MHz) at 23 °C.

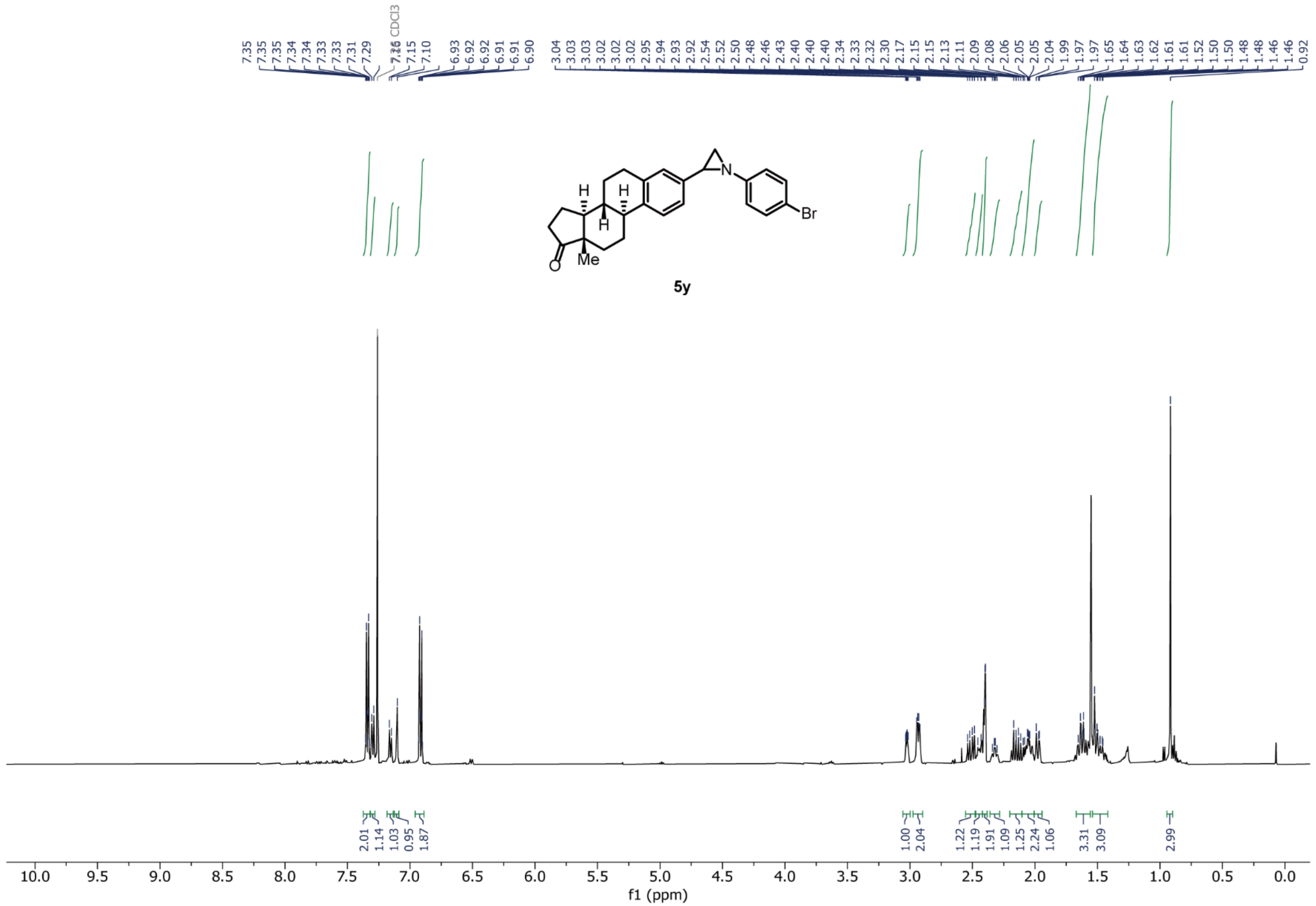

Supplementary Figure 97. ^{1}H NMR spectrum of (8*R*,9*S*,13*S*,14*S*)-3-(1-(4-bromophenyl)aziridin-2-yl)-13-methyl-6,7,8,9,11,12,13,14,15,16-decahydro-17*H*-cyclopenta[*a*]phenanthren-17-one (**5y**) in $CDCl_3$ (400 MHz) at 23 °C.

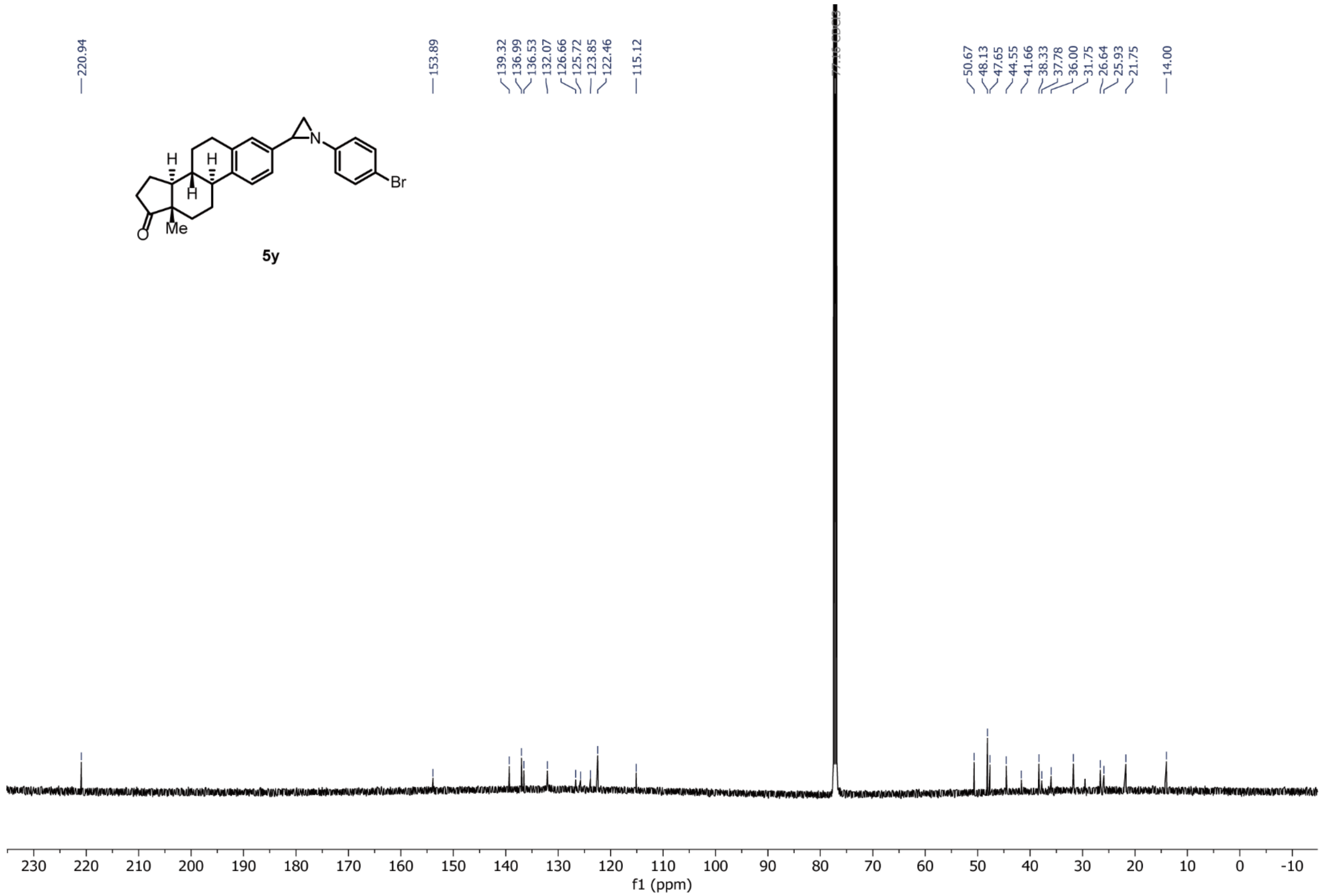

Supplementary Figure 98. ^{13}C NMR spectrum of (8*R*,9*S*,13*S*,14*S*)-3-(1-(4-bromophenyl)aziridin-2-yl)-13-methyl-6,7,8,9,11,12,13,14,15,16-decahydro-17*H*-cyclopenta[*a*]phenanthren-17-one (**5y**) in $CDCl_3$ (101 MHz) at 23 °C.

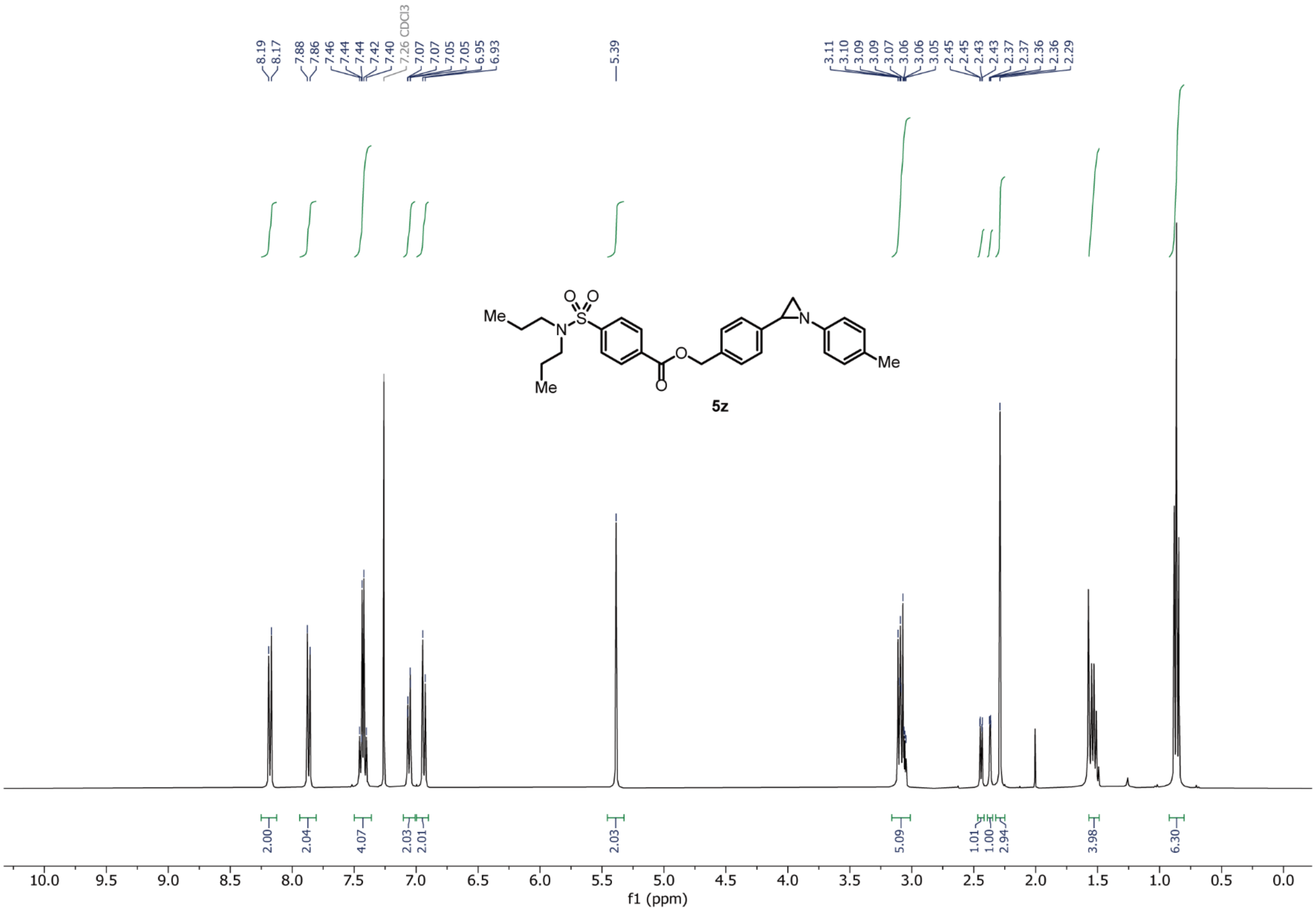

Figure 99. ^{1}H NMR spectrum of 4-(1-(*p*-tolyl)aziridin-2-yl)benzyl 4-(*N*,*N*-dipropylsulfamoyl)benzoate (**5z**) in $CDCl_3$ (400 MHz) at 23 °C.

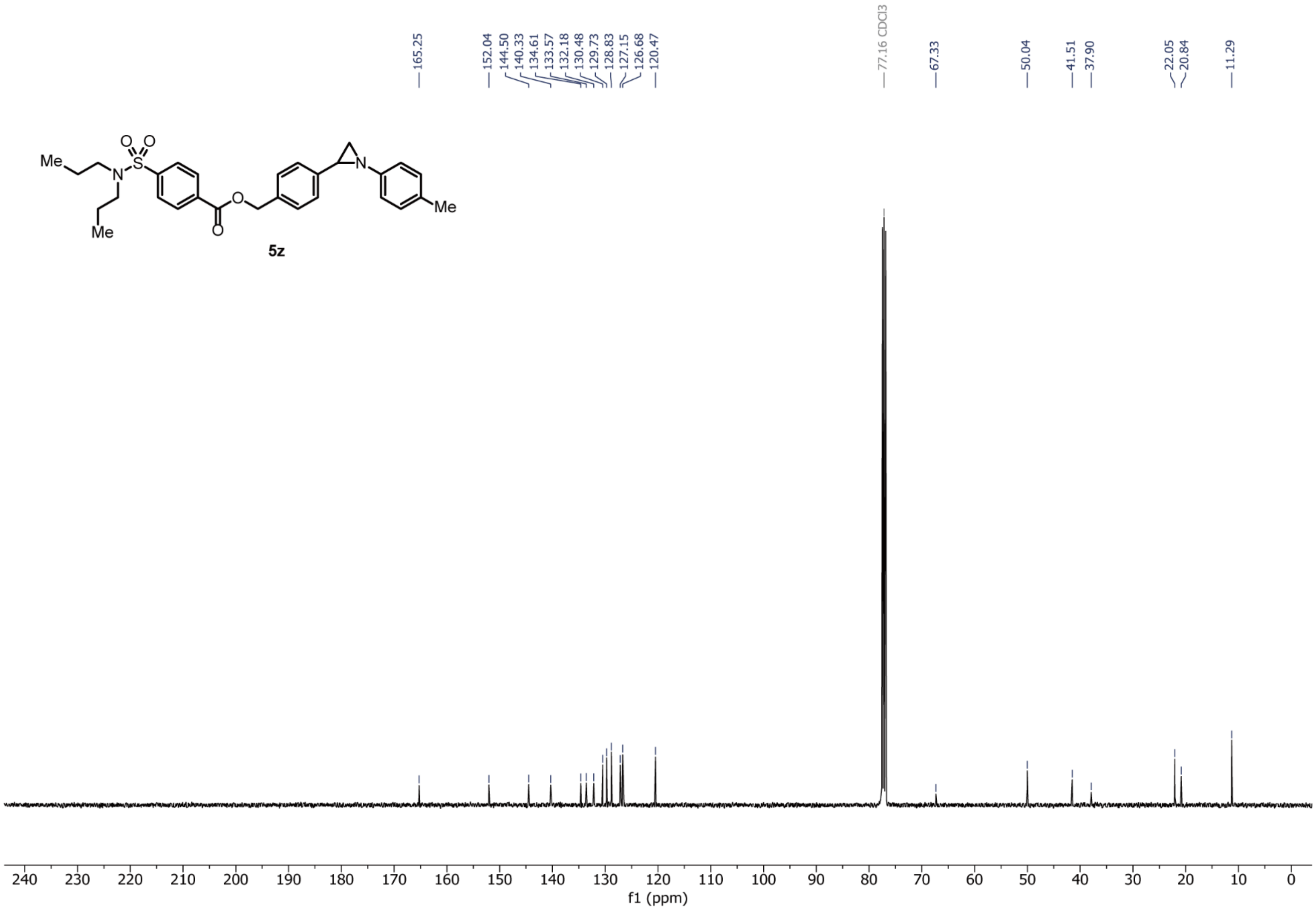

Supplementary Figure 100. ^{13}C NMR spectrum of 4-(1-(*p*-tolyl)aziridin-2-yl)benzyl 4-(*N*,*N*-dipropylsulfamoyl)benzoate (**5z**) in $CDCl_3$ (101 MHz) at 23 °C.

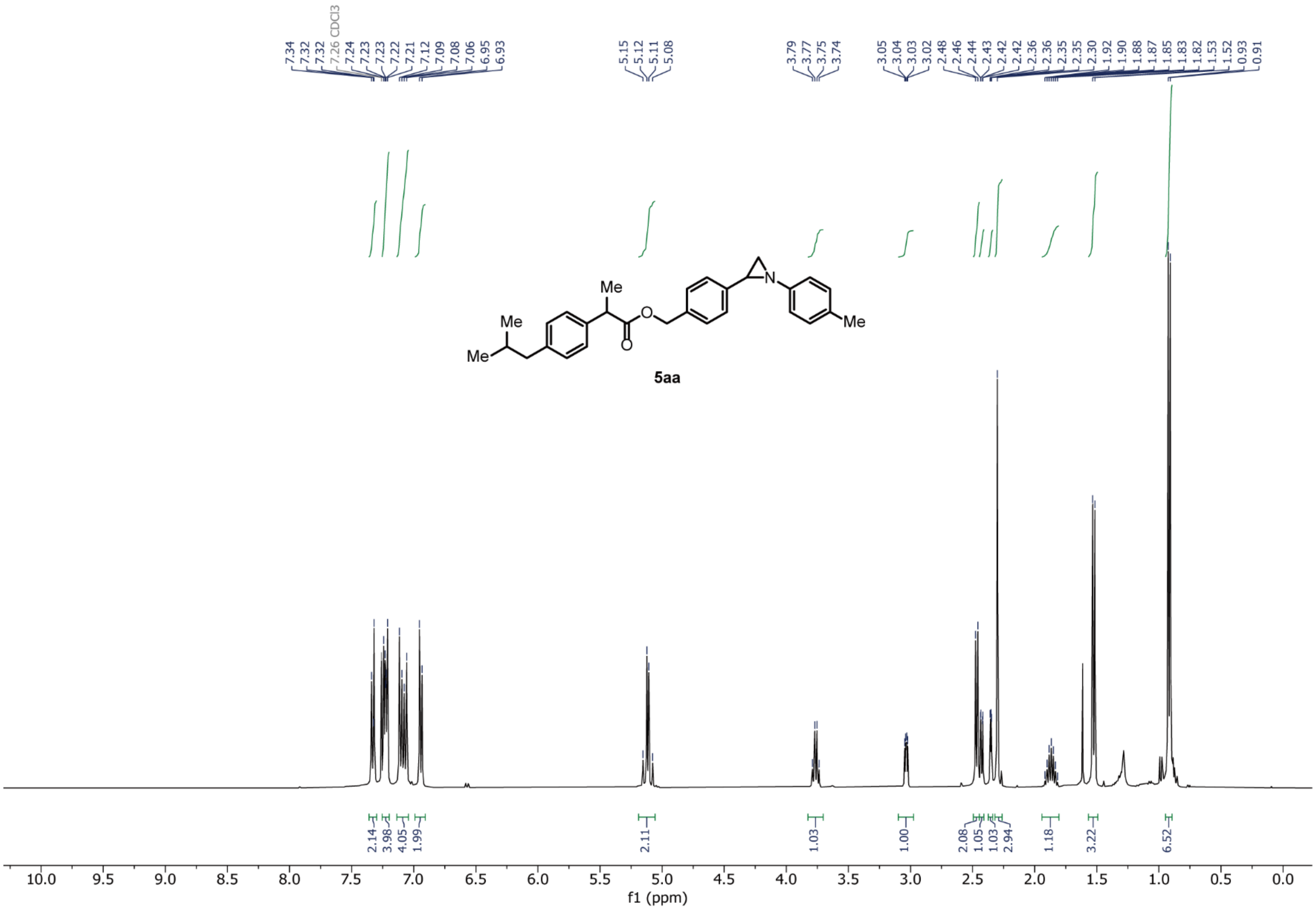

Supplementary Figure 101. ^{1}H NMR spectrum of 4-(1-(*p*-tolyl)aziridin-2-yl)benzyl 2-(4-isobutylphenyl)propanoate (**5aa**) in $CDCl_3$ (400 MHz) at 23 °C.

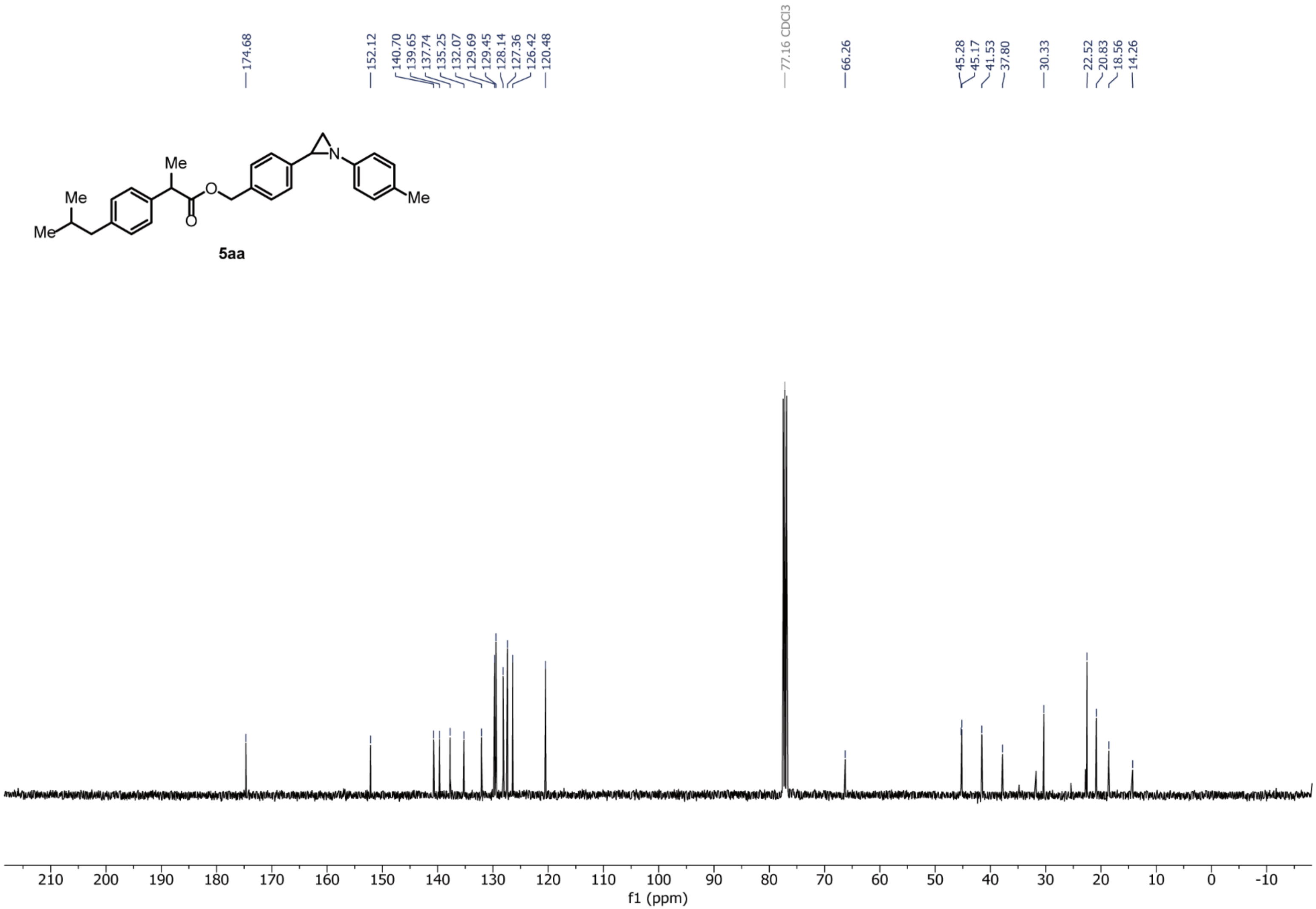

Supplementary Figure 102. ^{13}C NMR spectrum of 4-(1-(*p*-tolyl)aziridin-2-yl)benzyl 2-(4-isobutylphenyl)propanoate (**5aa**) in $CDCl_3$ (101 MHz) at 23 °C.

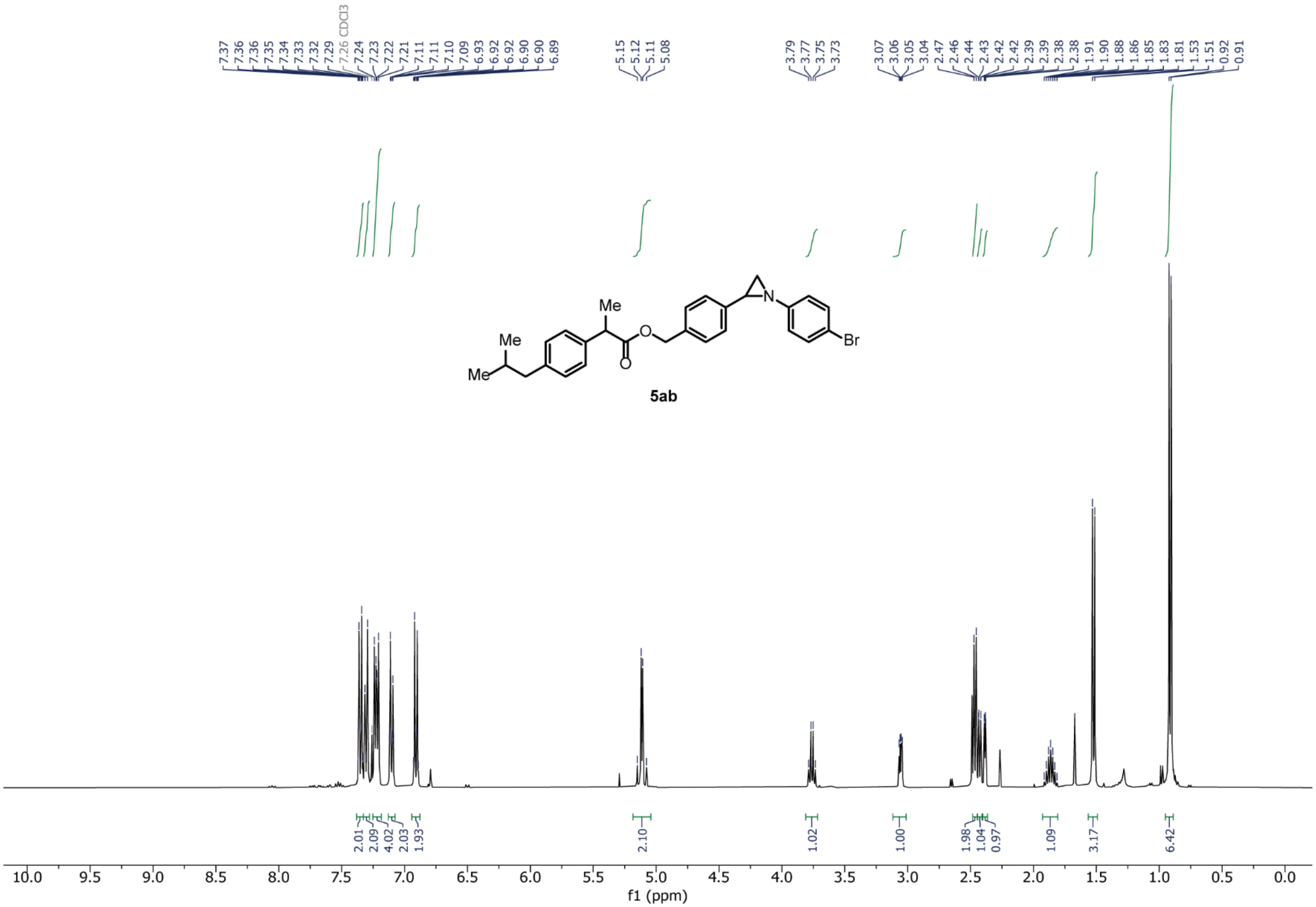

Supplementary Figure 103. ^{1}H NMR spectrum of 4-(1-(4-bromophenyl)aziridin-2-yl)benzyl 2-(4-isobutylphenyl)propanoate (**5ab**) in $CDCl_3$ (400 MHz) at 23 °C.

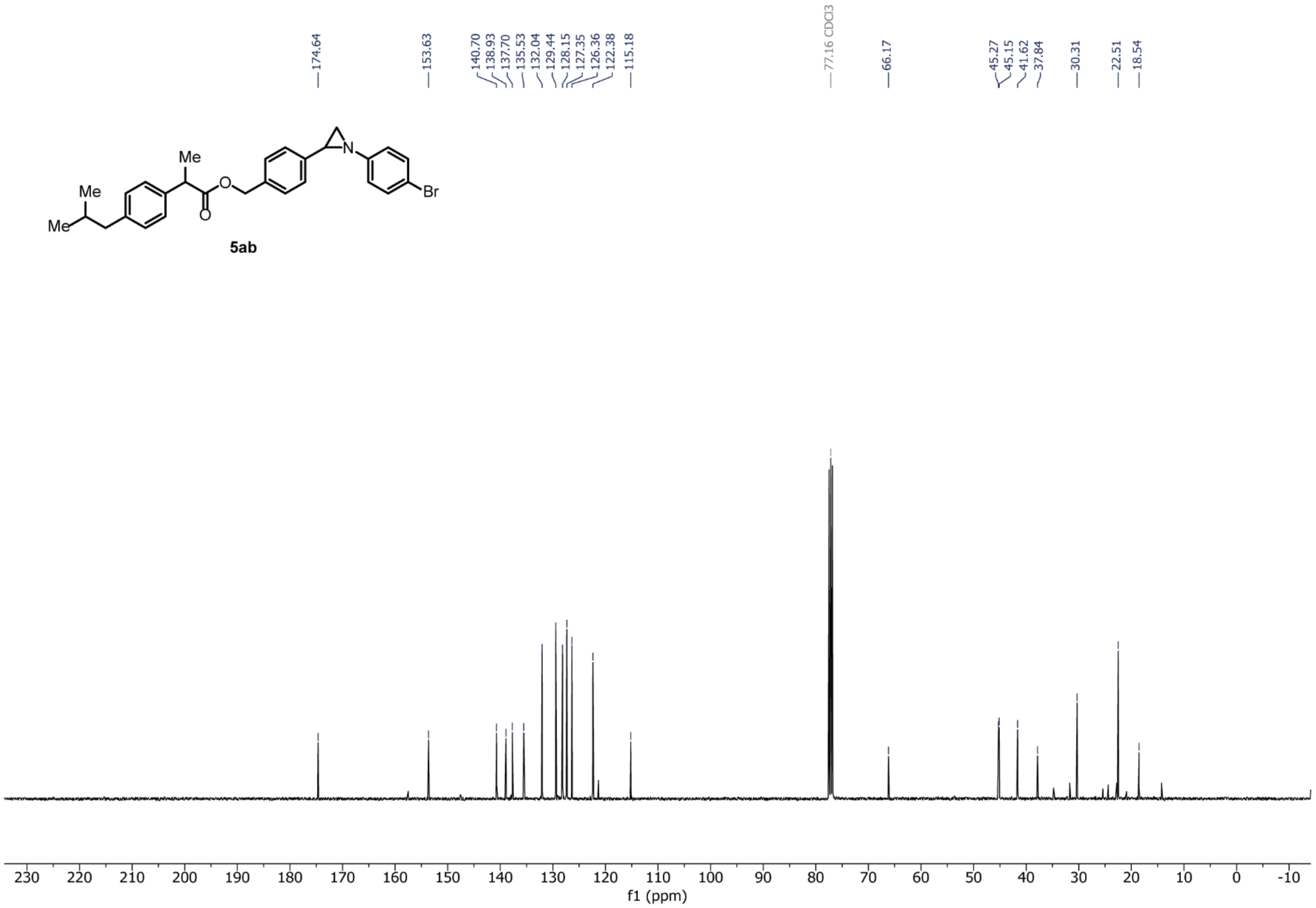

Supplementary Figure 104. ^{13}C NMR spectrum of 4-(1-(4-bromophenyl)aziridin-2-yl)benzyl 2-(4-isobutylphenyl)propanoate (**5ab**) in $CDCl_3$ (101 MHz) at 23 °C.

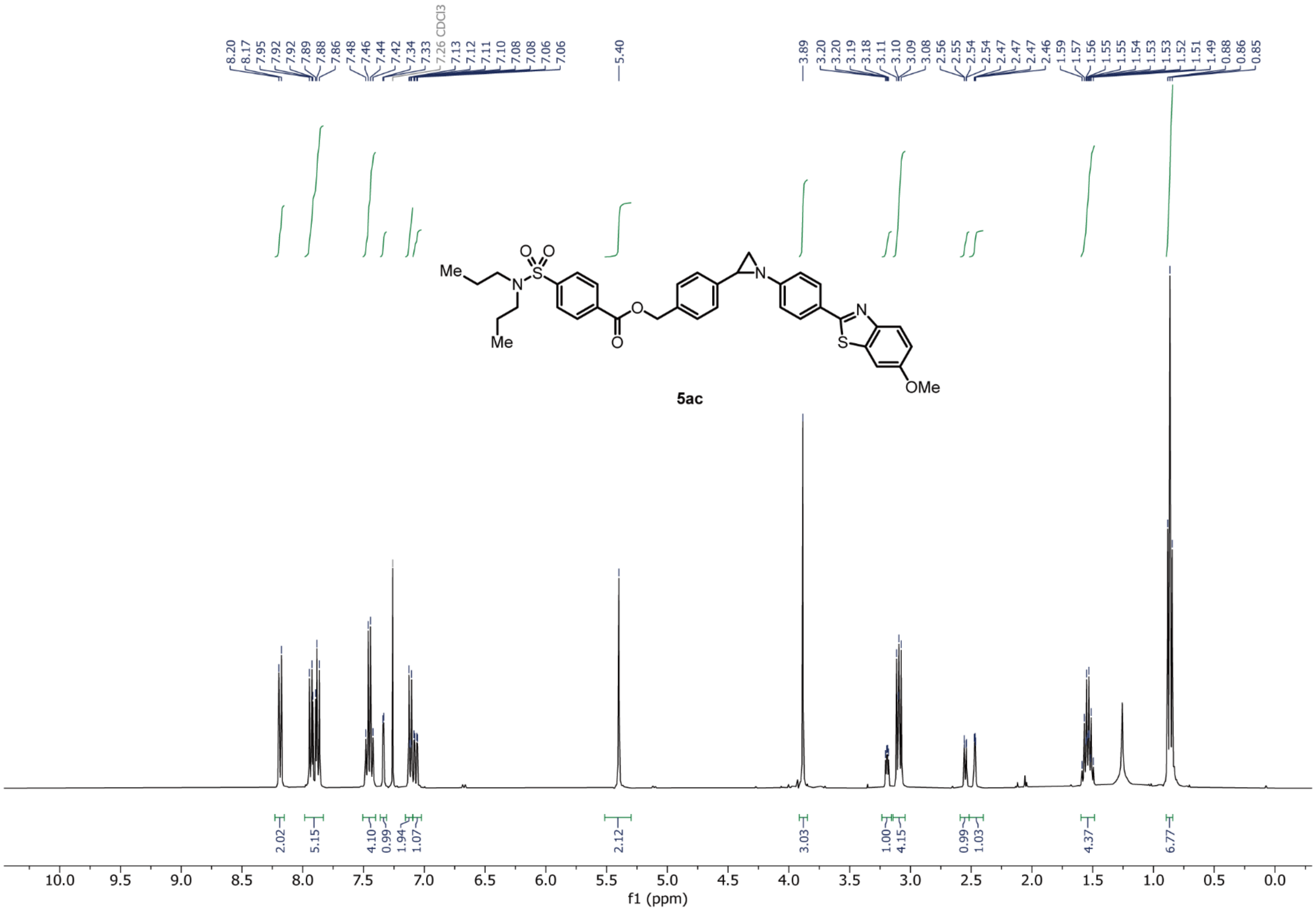

Supplementary Figure 105. ^{1}H NMR spectrum of 4-(1-(4-(6-methoxybenzo[*d*]thiazol-2-yl)phenyl)aziridin-2-yl)benzyl 4-(*N*,*N*-dipropylsulfamoyl)benzoate (**5ac**) in $CDCl_3$ (400 MHz) at 23 °C.

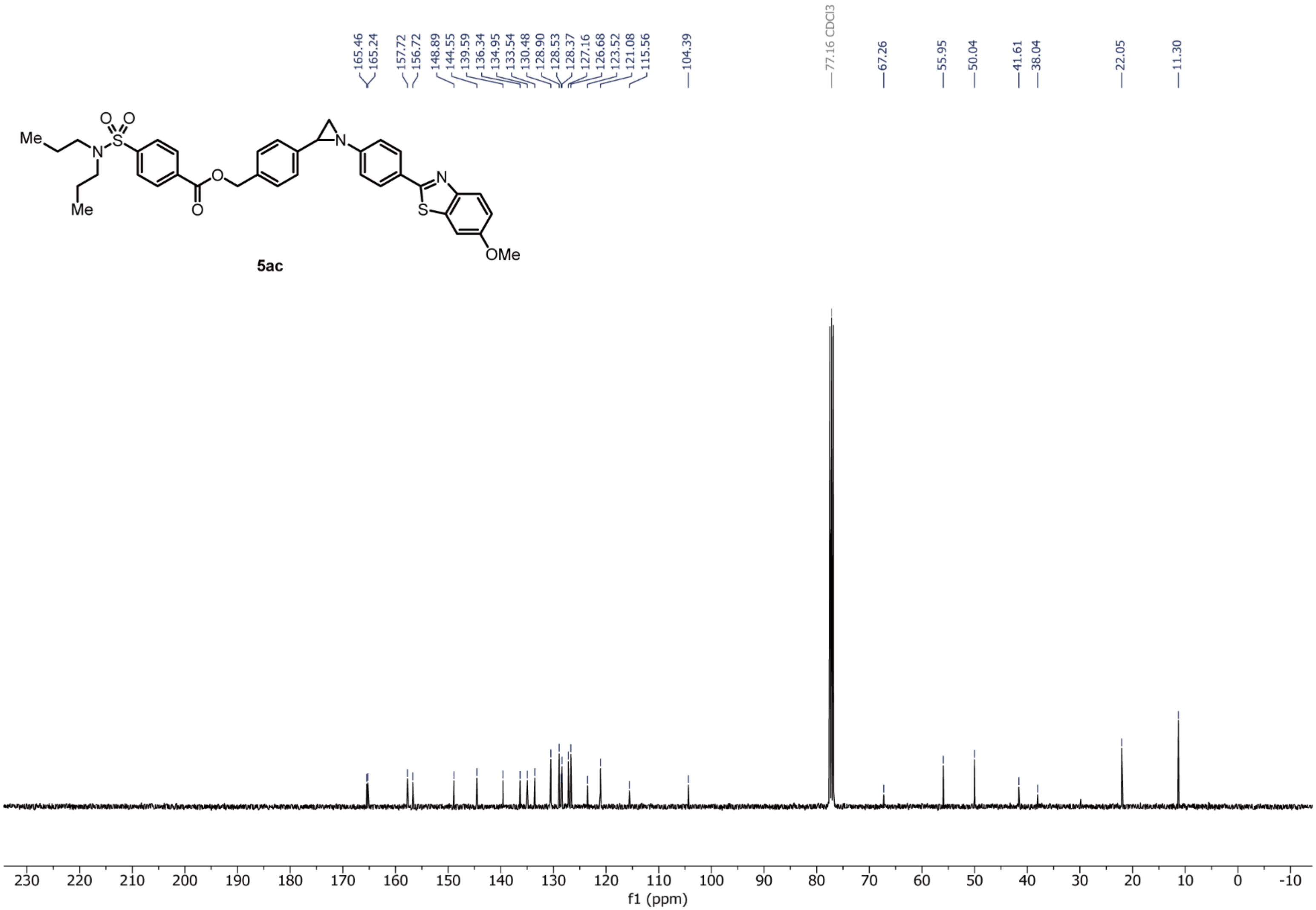

Supplementary Figure 106. ^{13}C NMR spectrum of 4-(1-(4-(6-methoxybenzo[*d*]thiazol-2-yl)phenyl)aziridin-2-yl)benzyl 4-(*N*,*N*-dipropylsulfamoyl)benzoate (**5ac**) in $CDCl_3$ (101 MHz) at 23 °C.

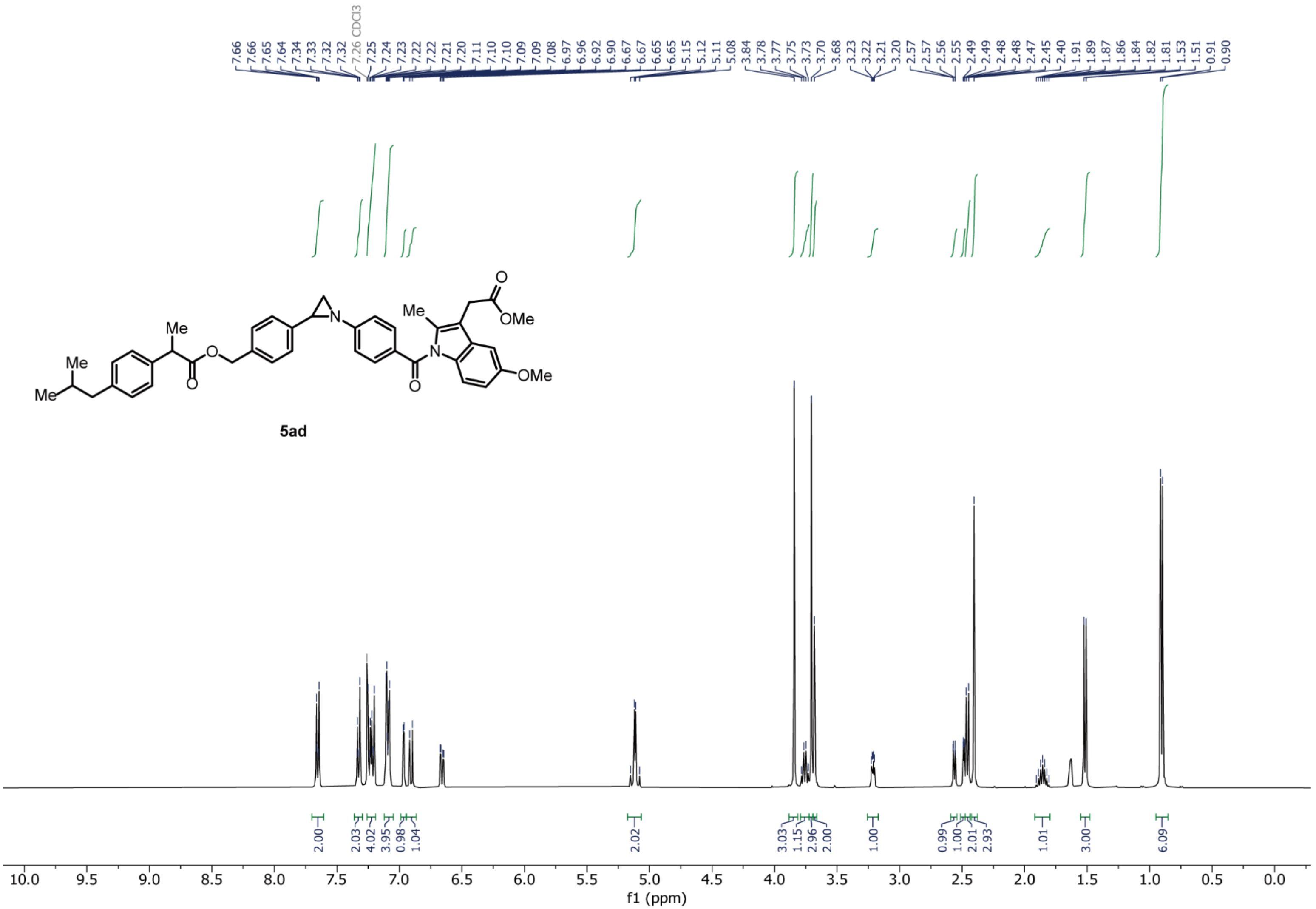

Supplementary Figure 107. ^{1}H NMR spectrum of 4-(1-(4-(5-methoxy-3-(2-methoxy-2-oxoethyl)-2-methyl-1*H*-indole-1-carbonyl)phenyl)aziridin-2-yl)benzyl 2-(4-isobutylphenyl)propanoate (**5ad**) in $CDCl_3$ (400 MHz) at 23 °C.

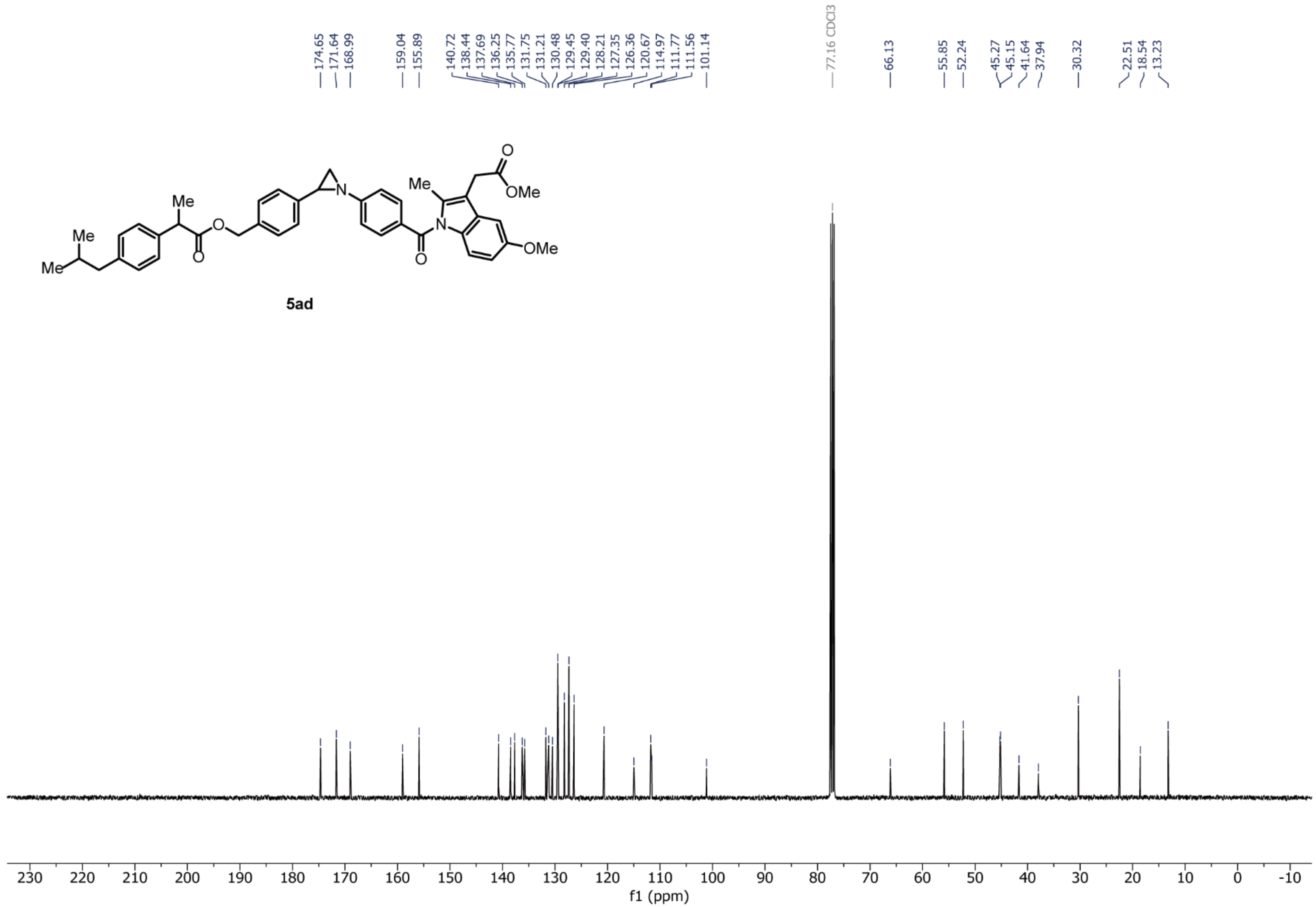

Supplementary Figure 108. ^{13}C NMR spectrum of 4-(1-(4-(5-methoxy-3-(2-methoxy-2-oxoethyl)-2-methyl-1*H*-indole-1-carbonyl)phenyl)aziridin-2-yl)benzyl 2-(4-isobutylphenyl)propanoate (**5ad**) in $CDCl_3$ (101 MHz) at 23 °C.

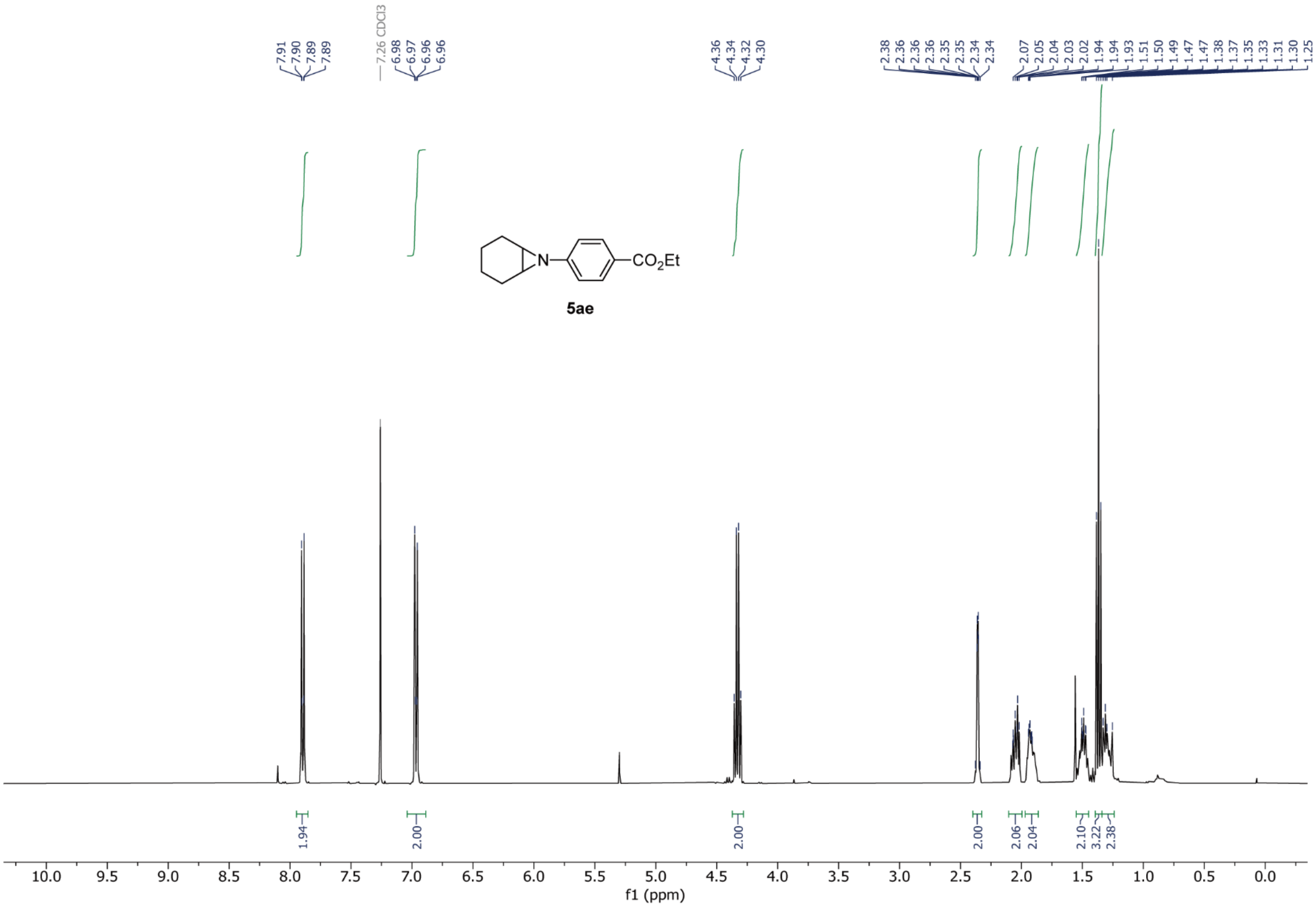

Supplementary Figure 109. ^{1}H NMR spectrum of ethyl 4-(7-azabicyclo[4.1.0]heptan-7-yl)benzoate (**5ae**) in $CDCl_3$ (400 MHz) at 23 °C.

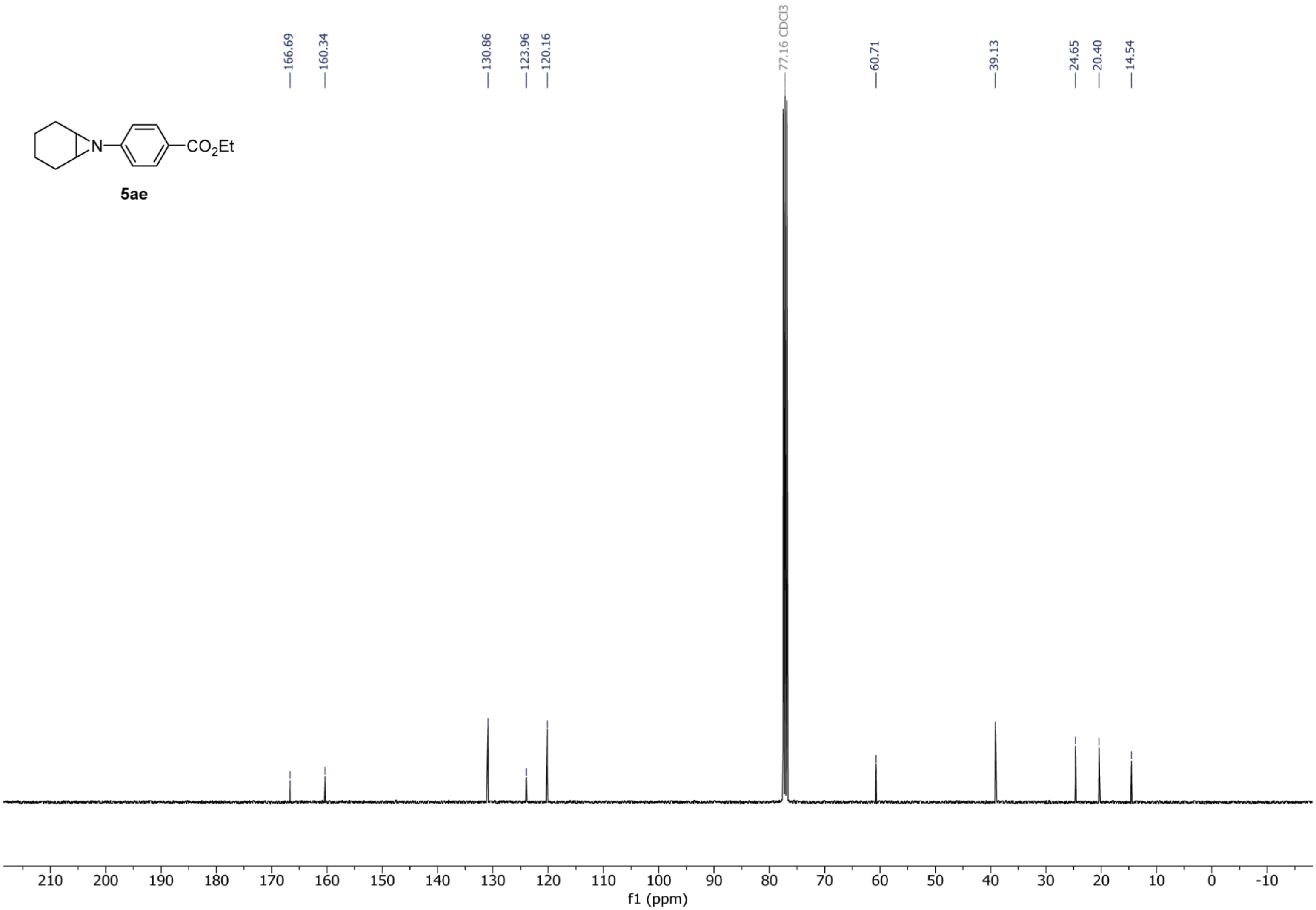

Supplementary Figure 110. ^{13}C NMR spectrum of ethyl 4-(7-azabicyclo[4.1.0]heptan-7-yl)benzoate (**5ae**) in $CDCl_3$ (101 MHz) at 23 °C.

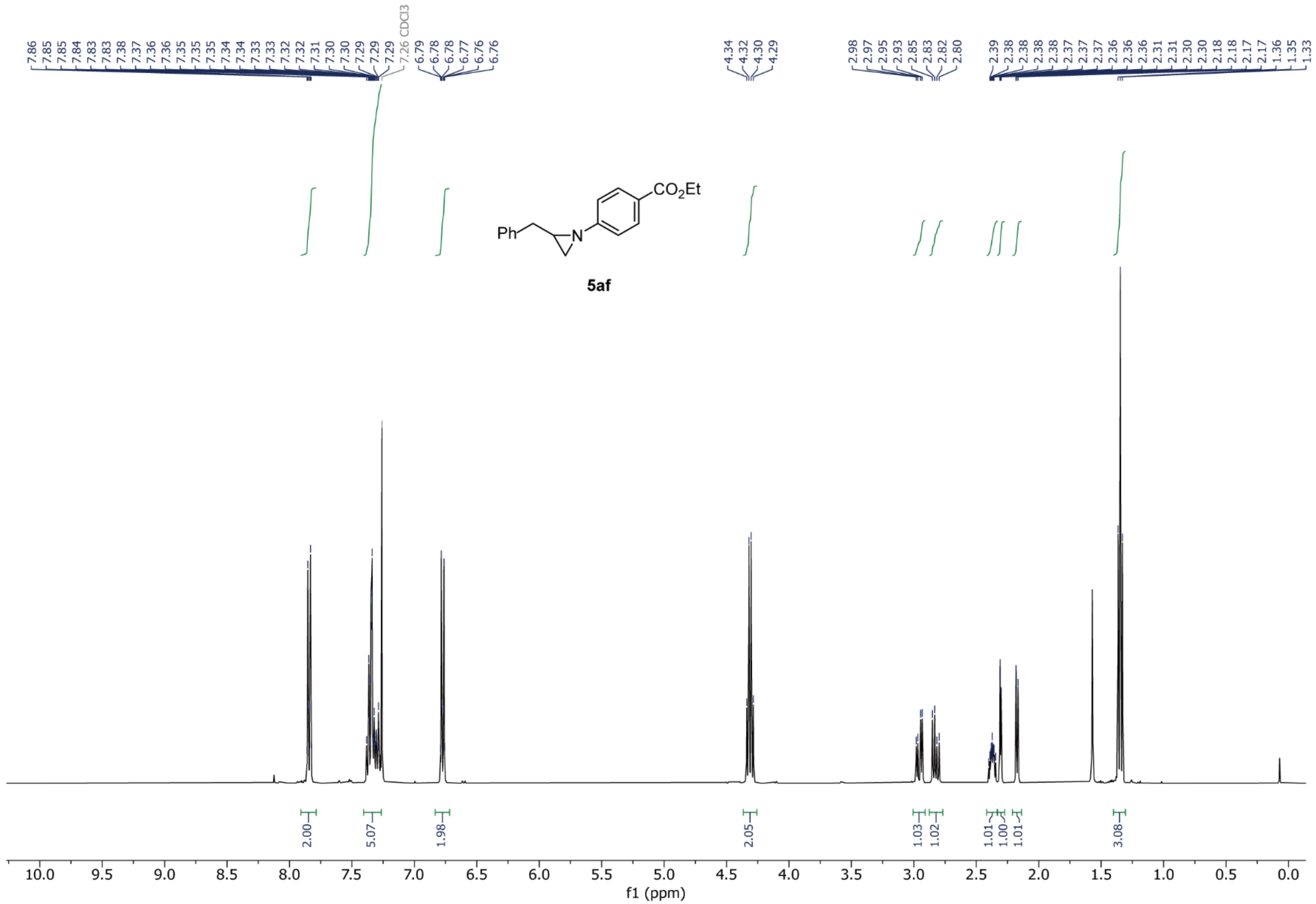

Supplementary Figure 111. ^{1}H NMR spectrum of ethyl 4-(2-benzylaziridin-1-yl)benzoate (**5af**) in $CDCl_3$ (400 MHz) at 23 °C.

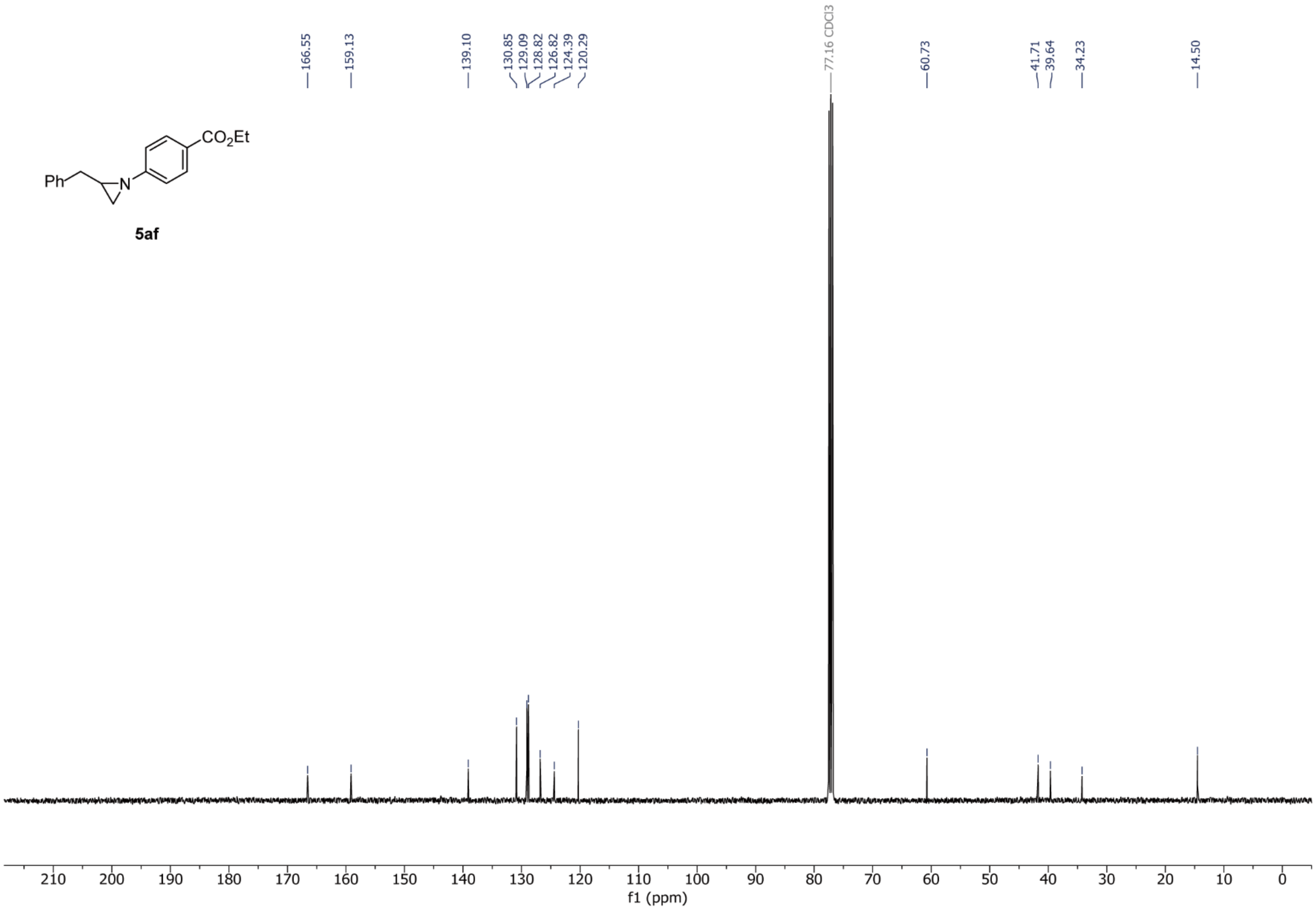

Supplementary Figure 112 ^{13}C NMR spectrum of ethyl 4-(2-benzylaziridin-1-yl)benzoate (**5af**) in $CDCl_3$ (101 MHz) at 23 °C.

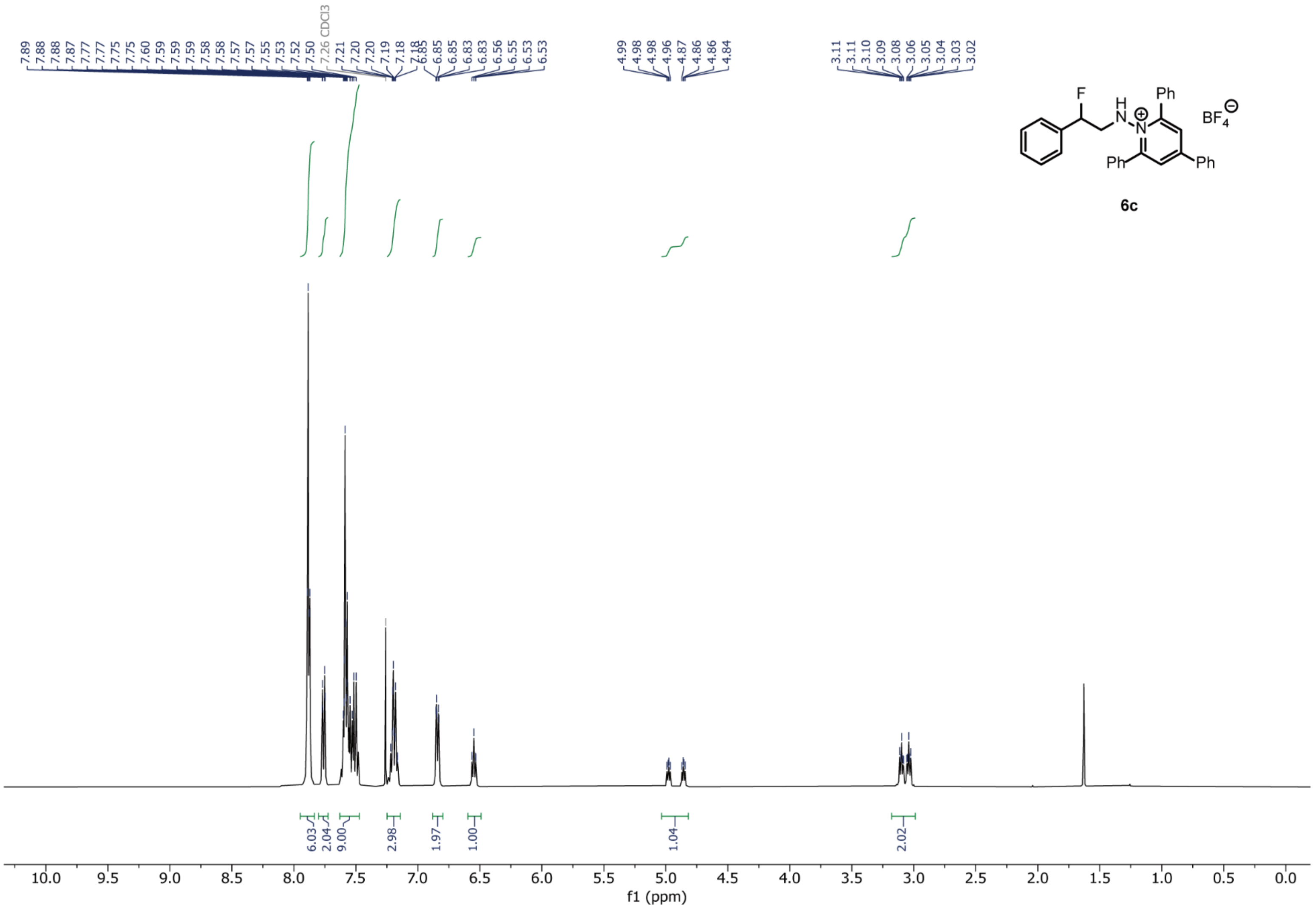

Supplementary Figure 113. ^{1}H NMR spectrum of 1-((2-fluoro-2-phenylethyl)amino)-2,4,6-triphenylpyridin-1-ium tetrafluoroborate (**6c**) in $CDCl_3$ (400 MHz) at 23 °C.

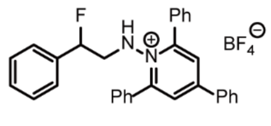

6c

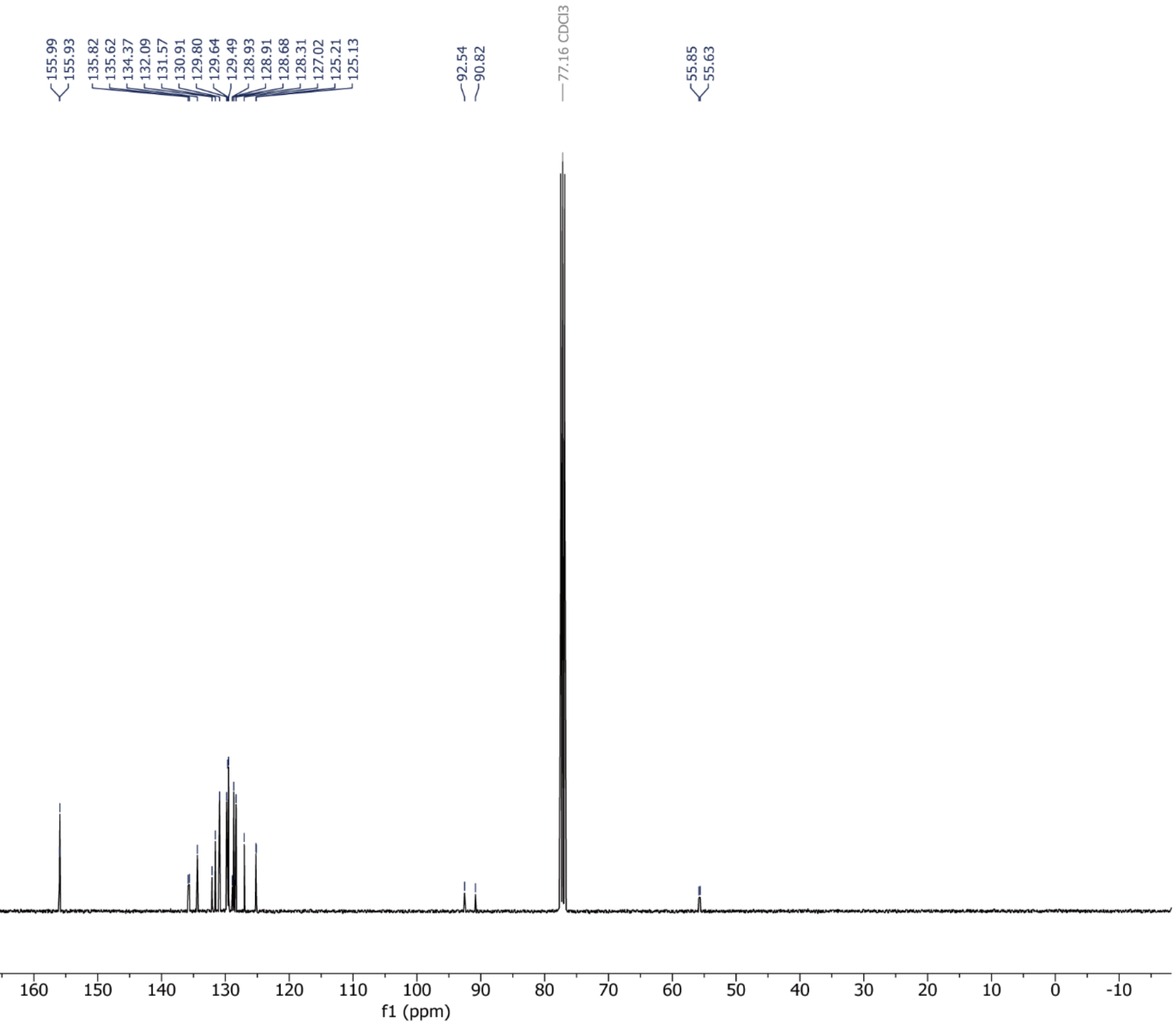

Supplementary Figure 114. ^{13}C NMR spectrum of 1-((2-fluoro-2-phenylethyl)amino)-2,4,6-triphenylpyridin-1-ium tetrafluoroborate (**6c**) in $CDCl_3$ (101 MHz) at 23 °C.

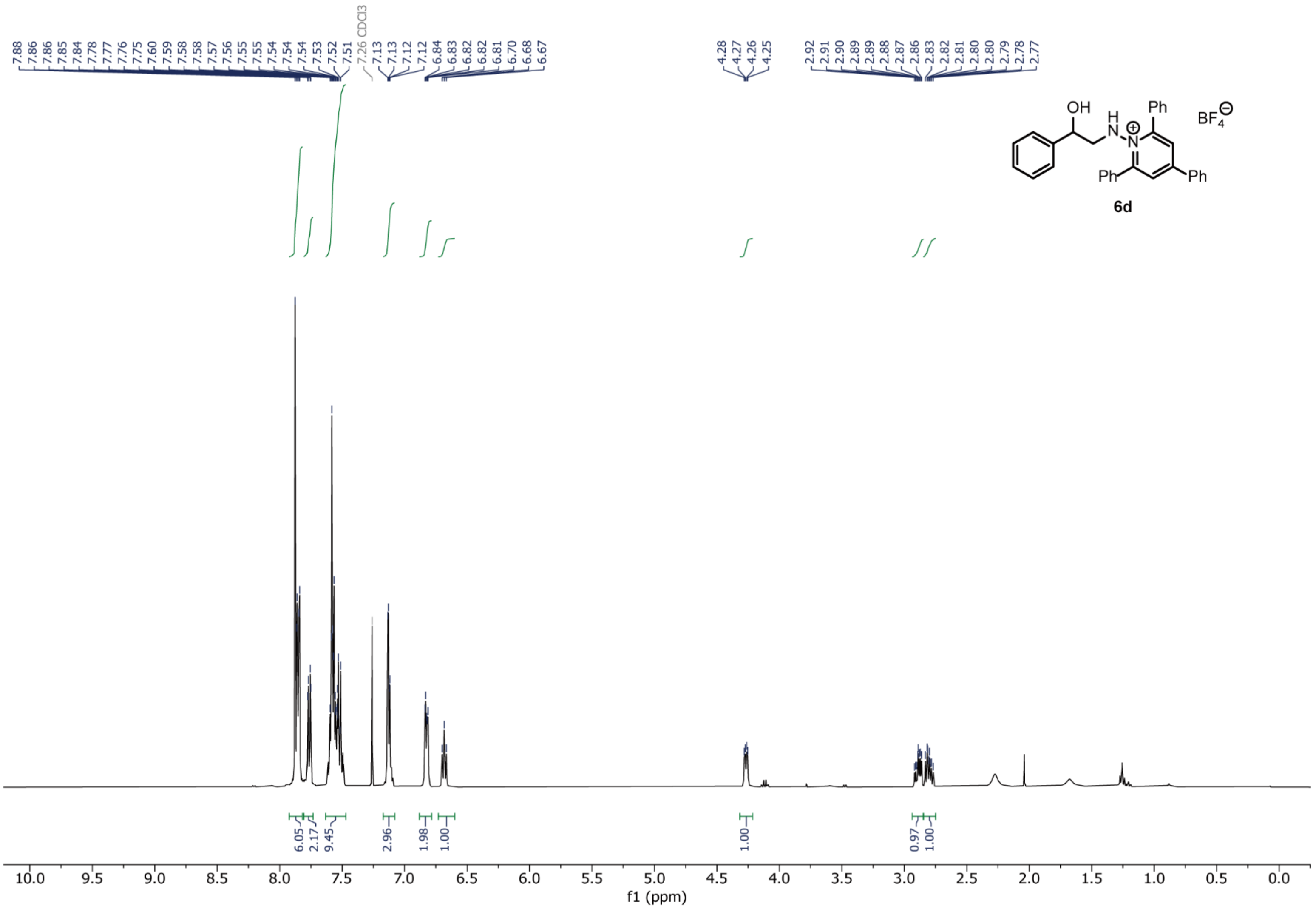

Supplementary Figure 115. ^{1}H NMR spectrum of 1-((2-hydroxy-2-phenylethyl)amino)-2,4,6-triphenylpyridin-1-ium tetrafluoroborate (**6d**) in $CDCl_3$ (400 MHz) at 23 °C.

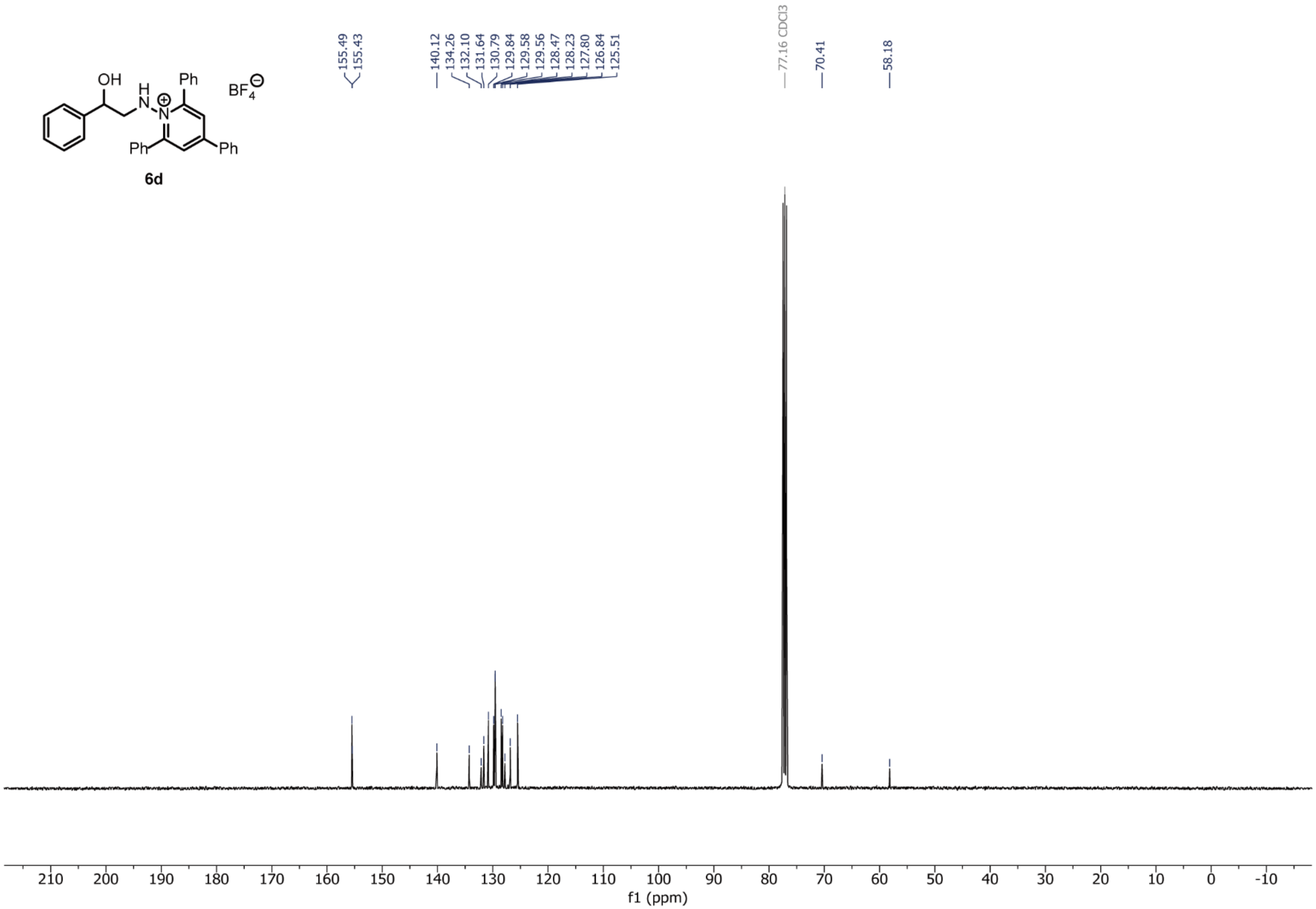

Supplementary Figure 116. ^{13}C NMR spectrum of 1-((2-hydroxy-2-phenylethyl)amino)-2,4,6-triphenylpyridin-1-ium tetrafluoroborate (**6d**) in $CDCl_3$ (101 MHz) at 23 °C.

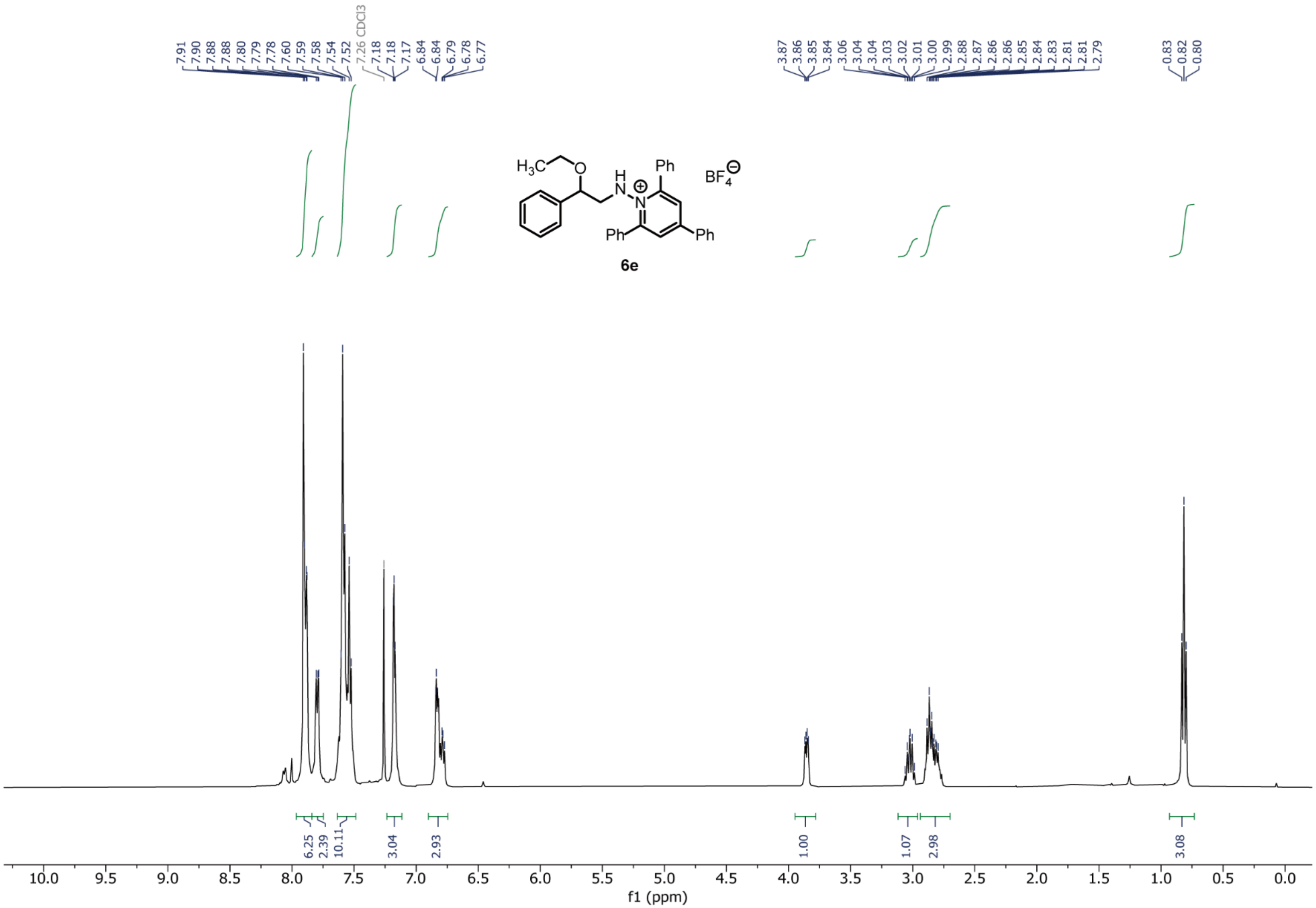

Supplementary Figure 117. ^1H NMR spectrum of 1-((2-ethoxy-2-phenylethyl)amino)-2,4,6-triphenylpyridin-1-ium tetrafluoroborate (**6e**) in $CDCl_3$ (400 MHz) at 23 °C.

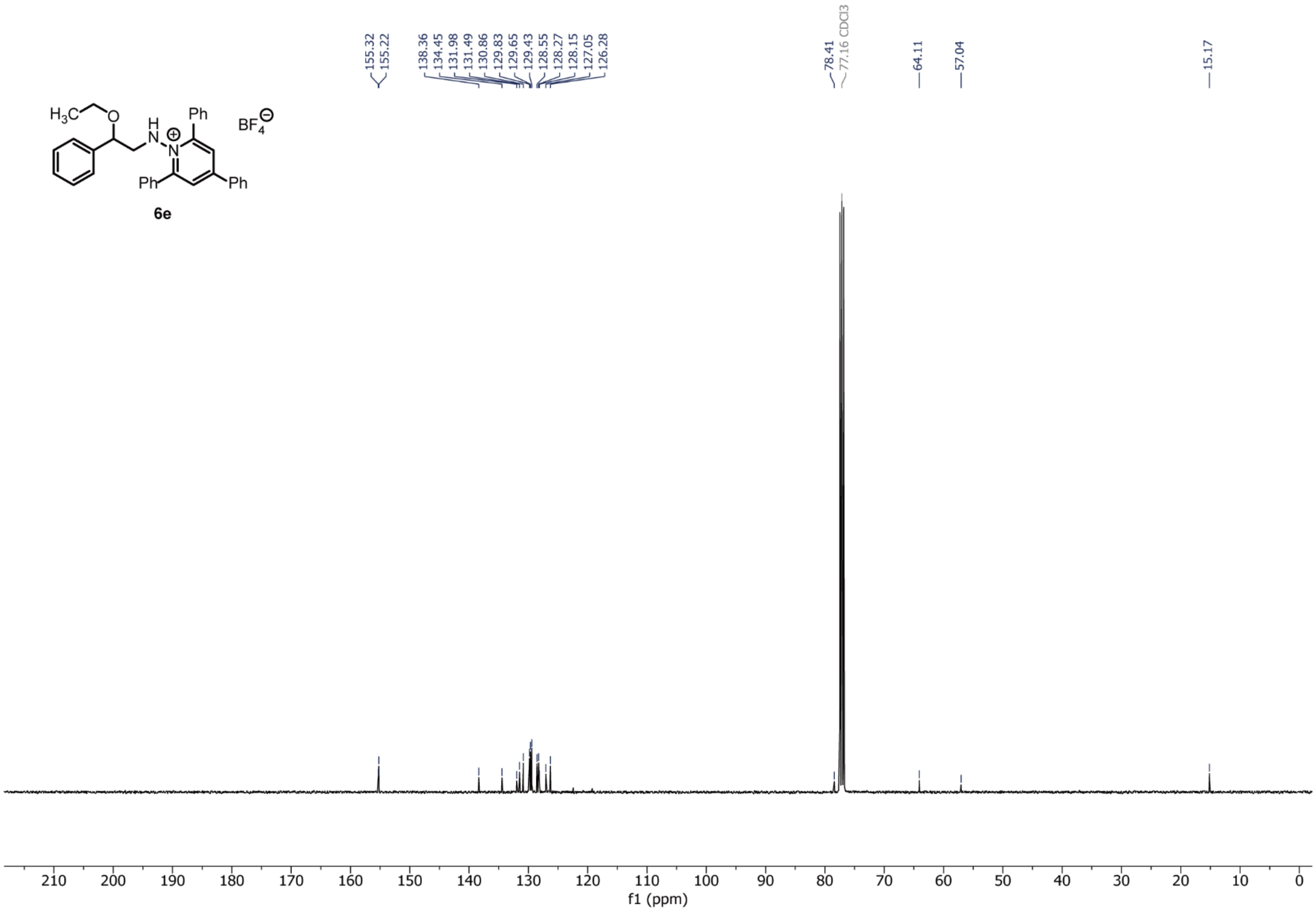

Supplementary Figure 118. ^{13}C NMR spectrum of 1-((2-ethoxy-2-phenylethyl)amino)-2,4,6-triphenylpyridin-1-ium tetrafluoroborate (**6e**) in $CDCl_3$ (101 MHz) at 23 °C.

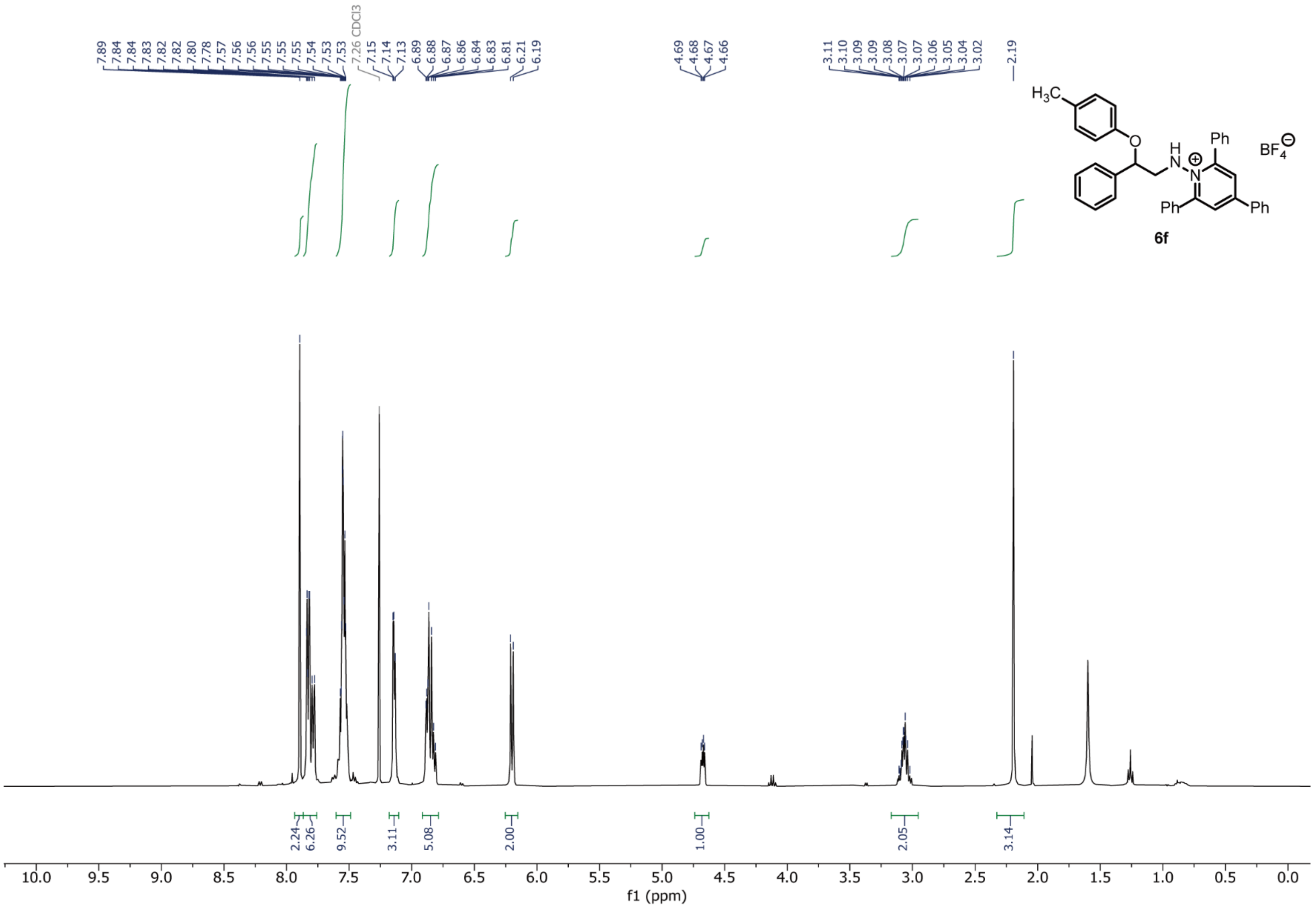

Supplementary Figure 119. ^{1}H NMR spectrum of 2,4,6-triphenyl-1-((2-phenyl-2-(*p*-tolyloxy)ethyl)amino)pyridin-1-ium tetrafluoroborate (**6f**) in $CDCl_3$ (400 MHz) at 23 °C.

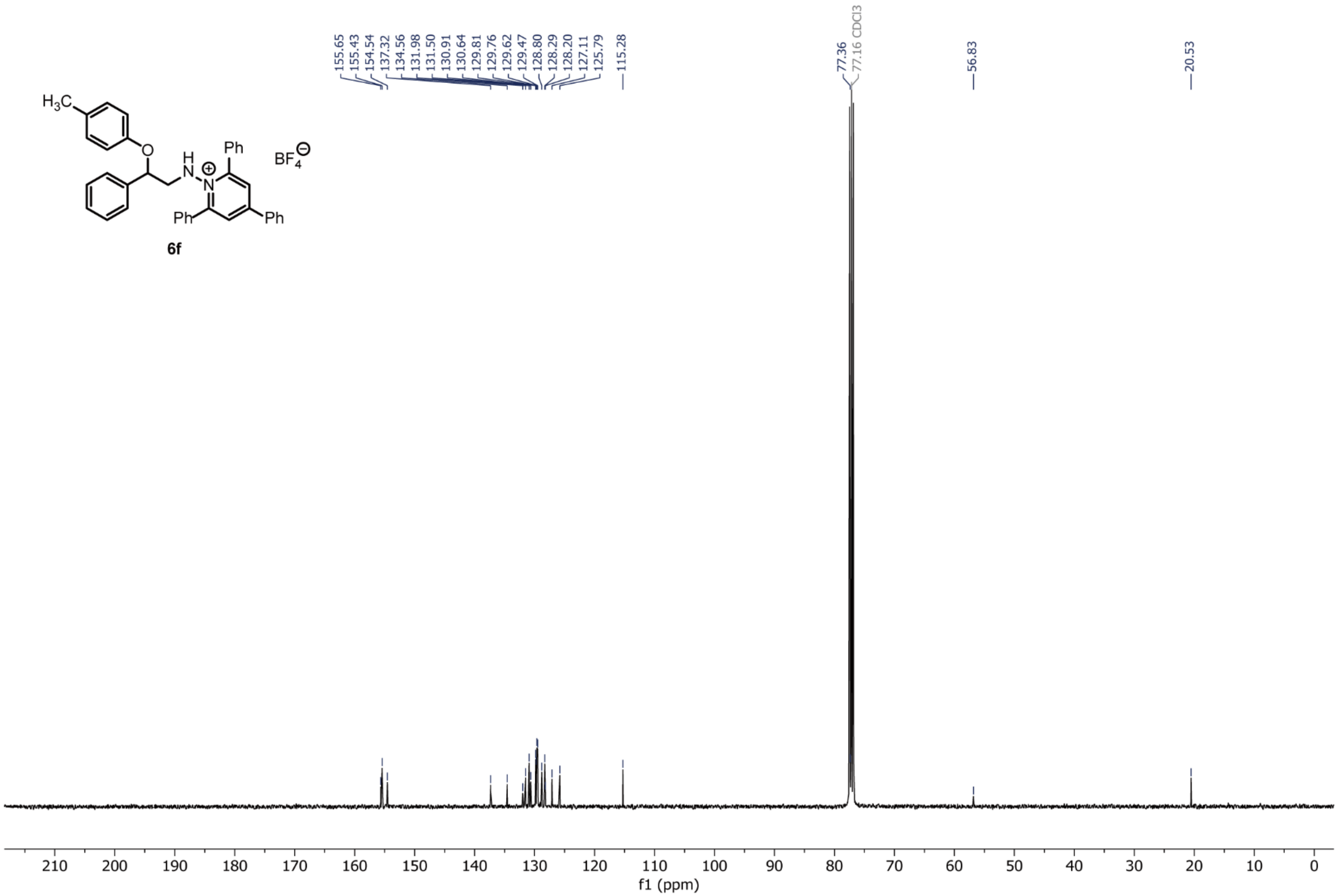

Supplementary Figure 120. ^{13}C NMR spectrum of 2,4,6-triphenyl-1-((2-phenyl-2-(*p*-tolyloxy)ethyl)amino)pyridin-1-ium tetrafluoroborate (**6f**) in $CDCl_3$ (101 MHz) at 23 °C.

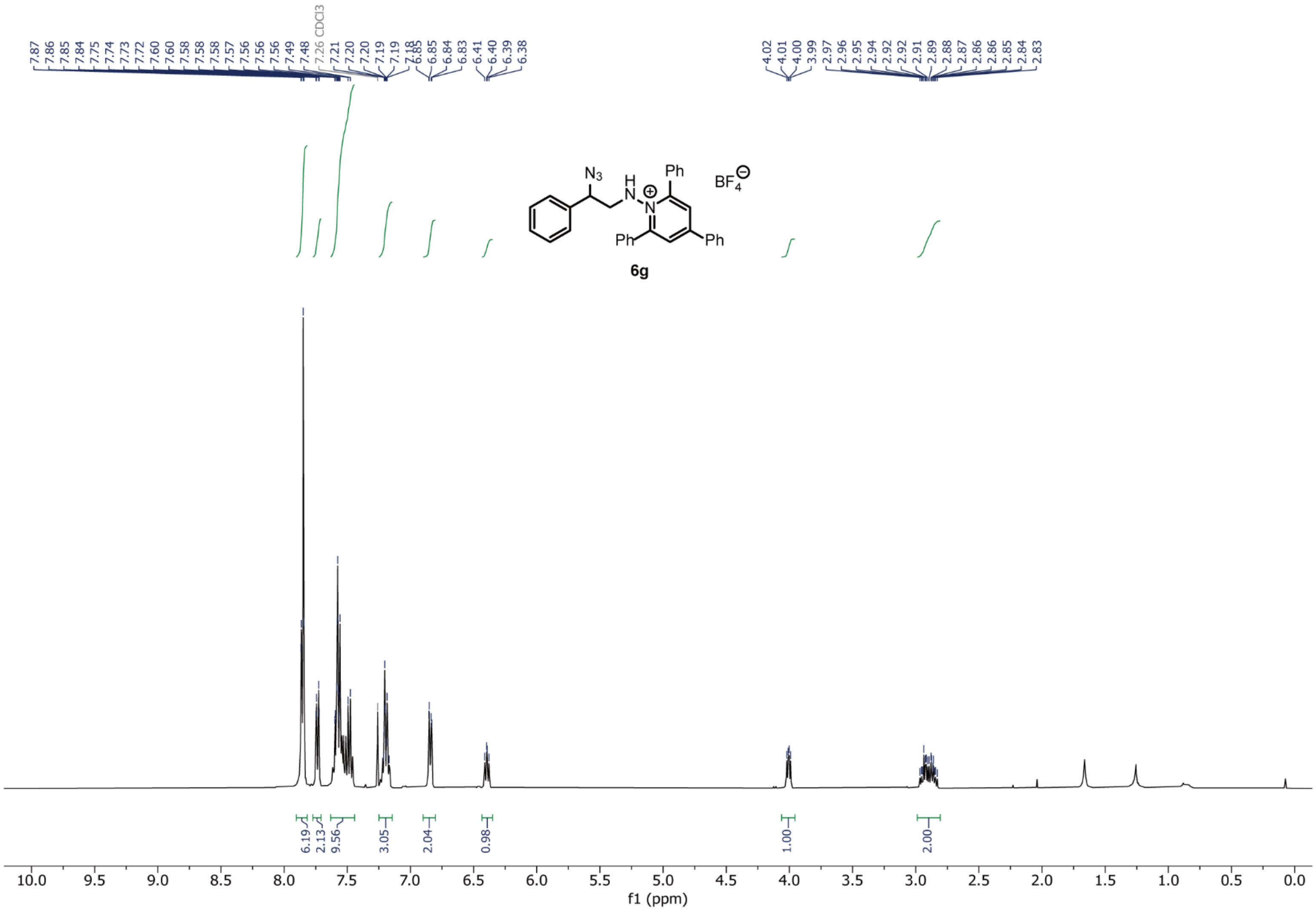

Supplementary Figure 121. ^{1}H NMR spectrum of 1-((2-azido-2-phenylethyl)amino)-2,4,6-triphenylpyridin-1-ium tetrafluoroborate (**6g**) in $CDCl_3$ (400 MHz) at 23 °C.

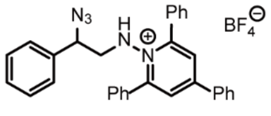

6g

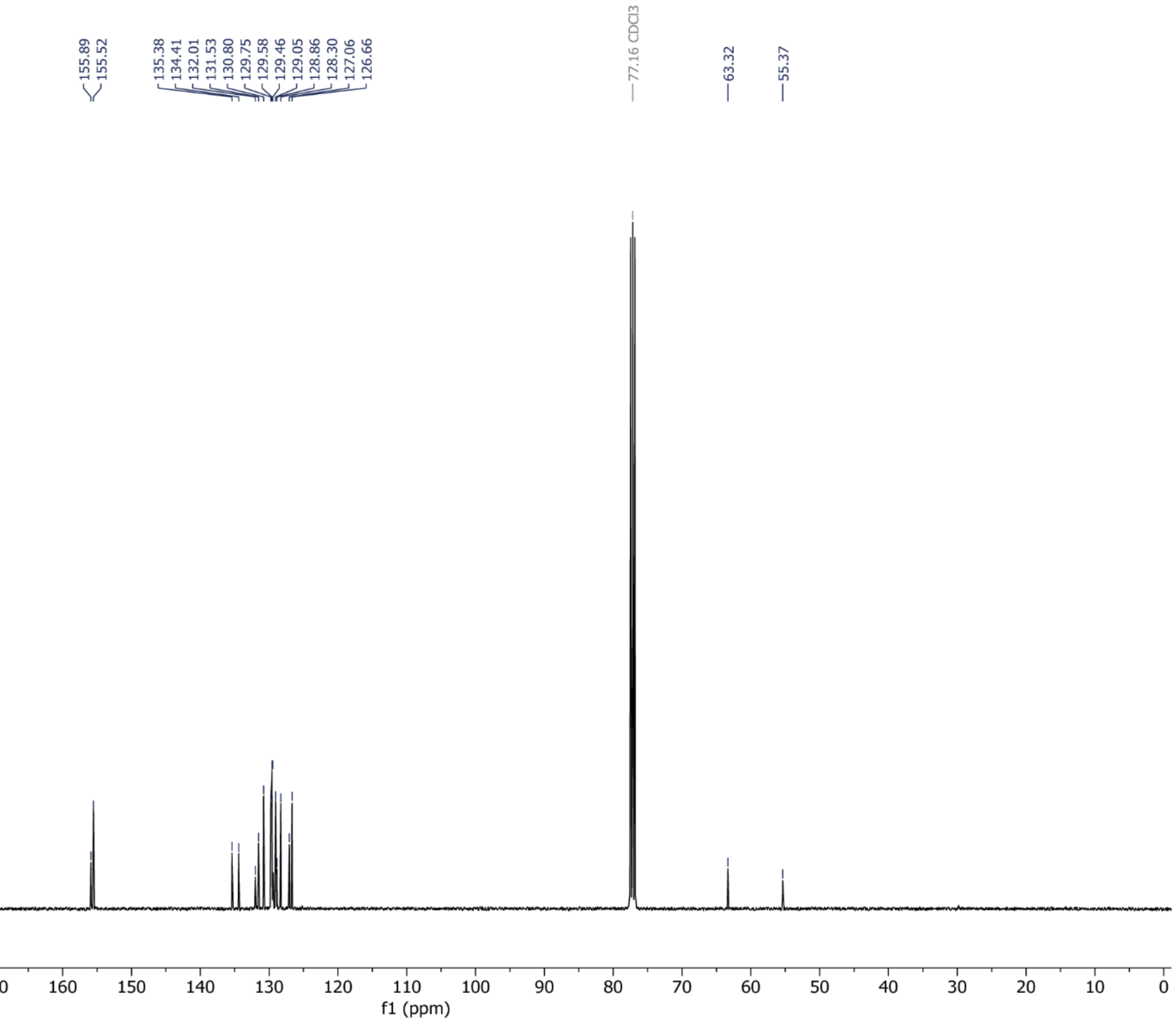

Supplementary Figure 122. ^{13}C NMR spectrum of 1-((2-azido-2-phenylethyl)amino)-2,4,6-triphenylpyridin-1-ium tetrafluoroborate (**6g**) in $CDCl_3$ (101 MHz) at 23 °C.

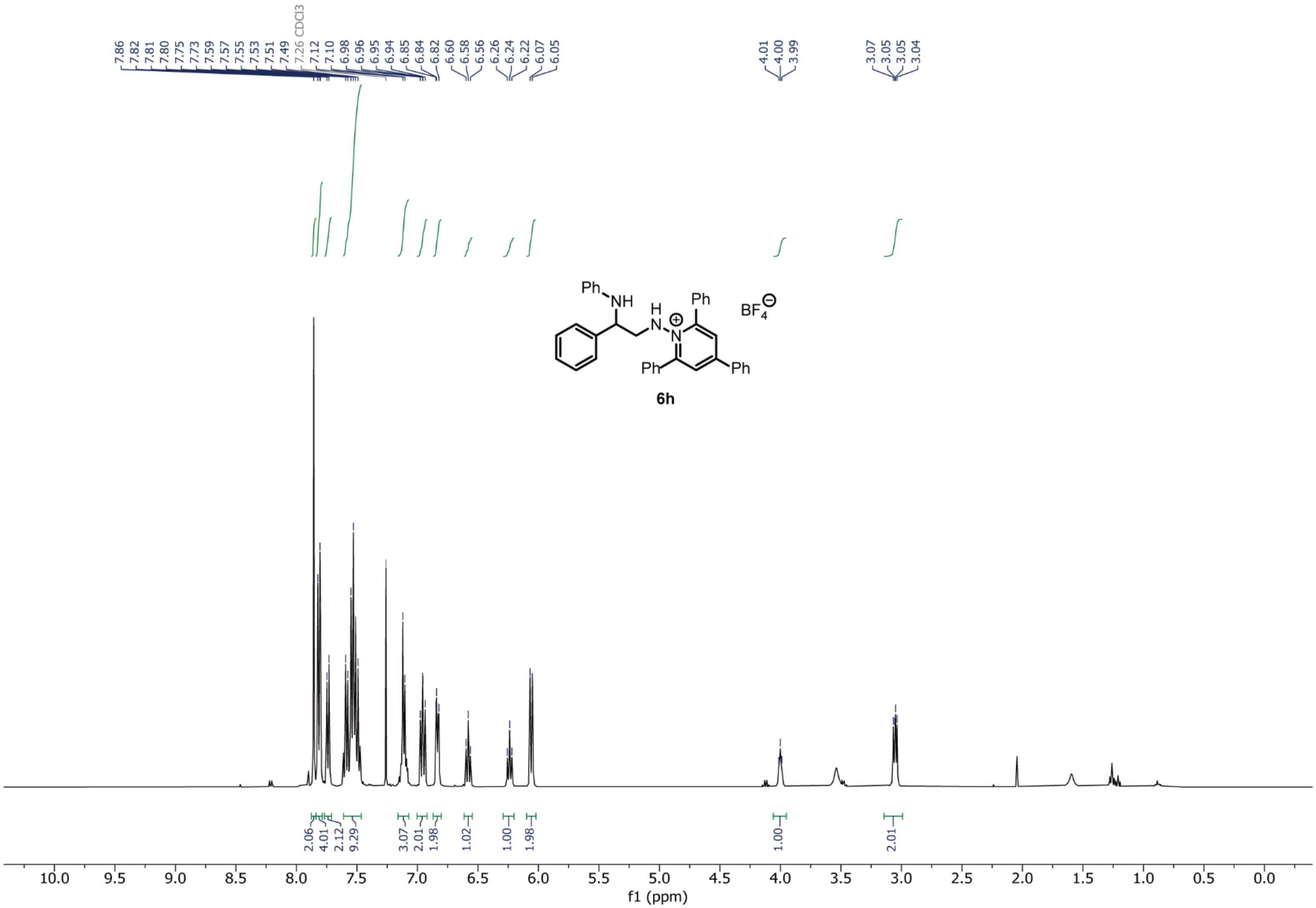

Supplementary Figure 123. ^{1}H NMR spectrum of 2,4,6-triphenyl-1-((2-phenyl-2-(phenylamino)ethyl)amino)pyridin-1-ium tetrafluoroborate (**6h**) in $CDCl_3$ (400 MHz) at 23 °C.

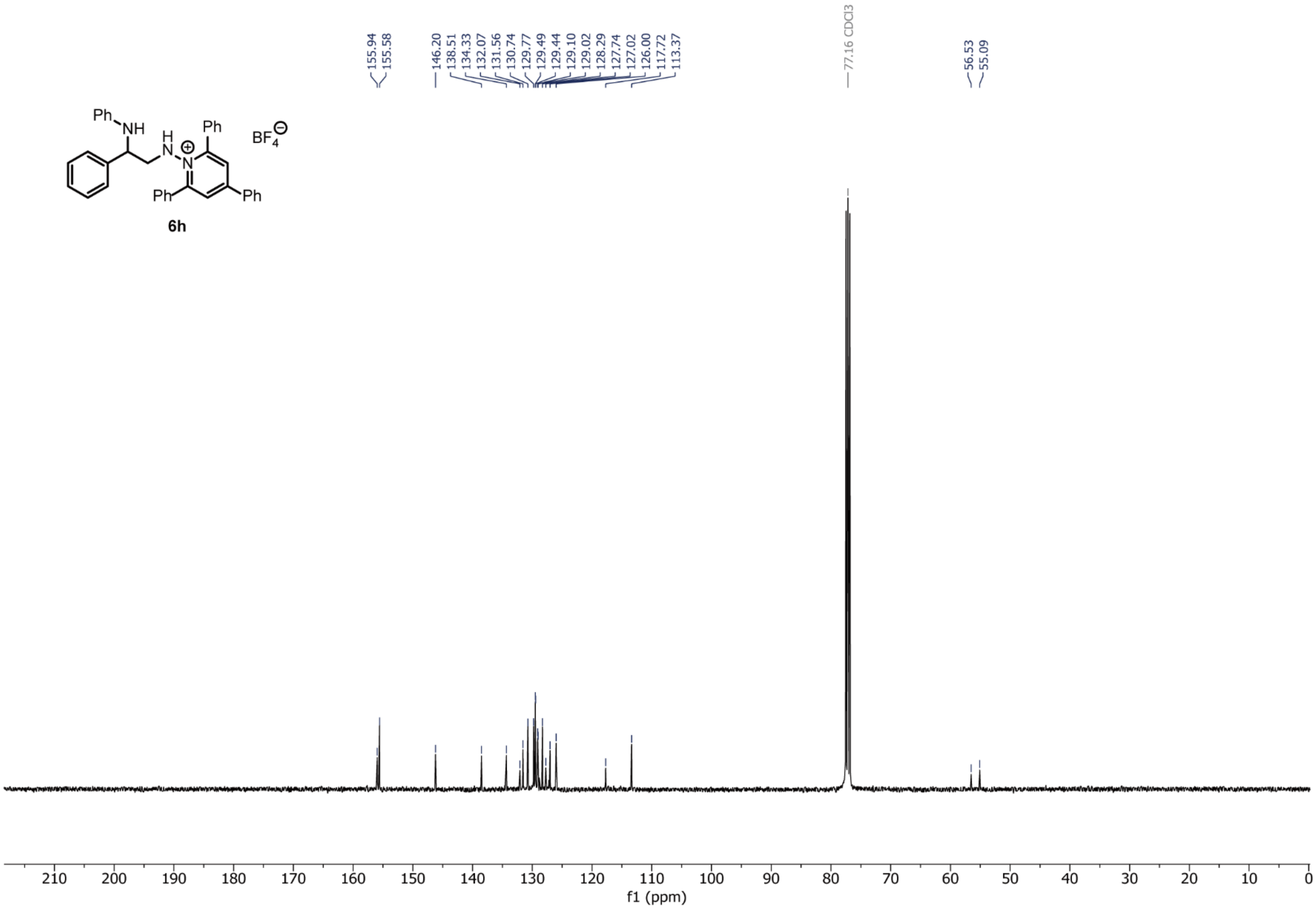

Supplementary Figure 124. ^{13}C NMR spectrum of 2,4,6-triphenyl-1-((2-phenyl-2-(phenylamino)ethyl)amino)pyridin-1-ium tetrafluoroborate (**6h**) in $CDCl_3$ (101 MHz) at 23 °C.

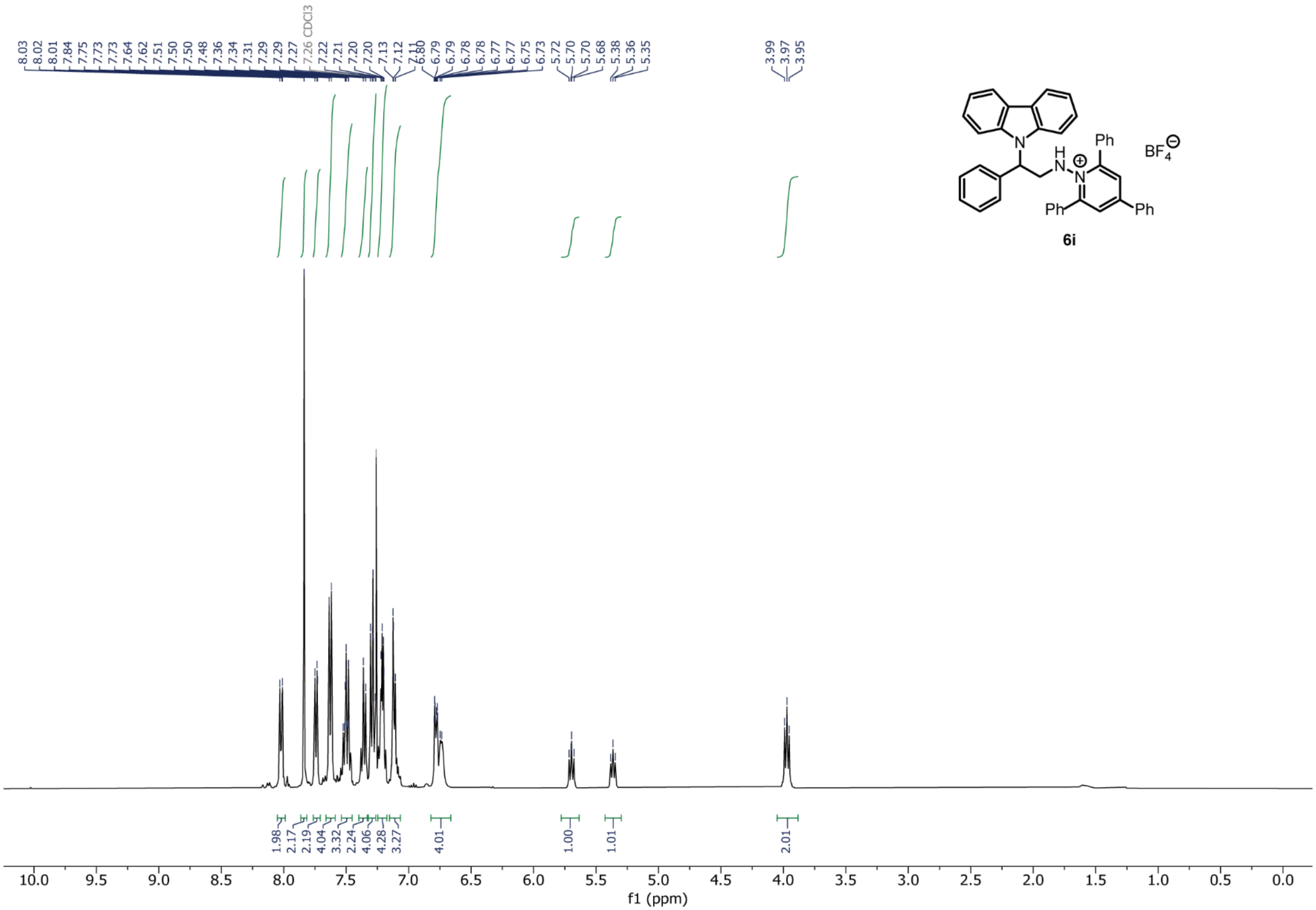

Supplementary Figure 125. ^{1}H NMR spectrum of 1-((2-(9*H*-carbazol-9-yl)-2-phenylethyl)amino)-2,4,6-triphenylpyridin-1-ium tetrafluoroborate (**6i**) in $CDCl_3$ (400 MHz) at 23 °C.

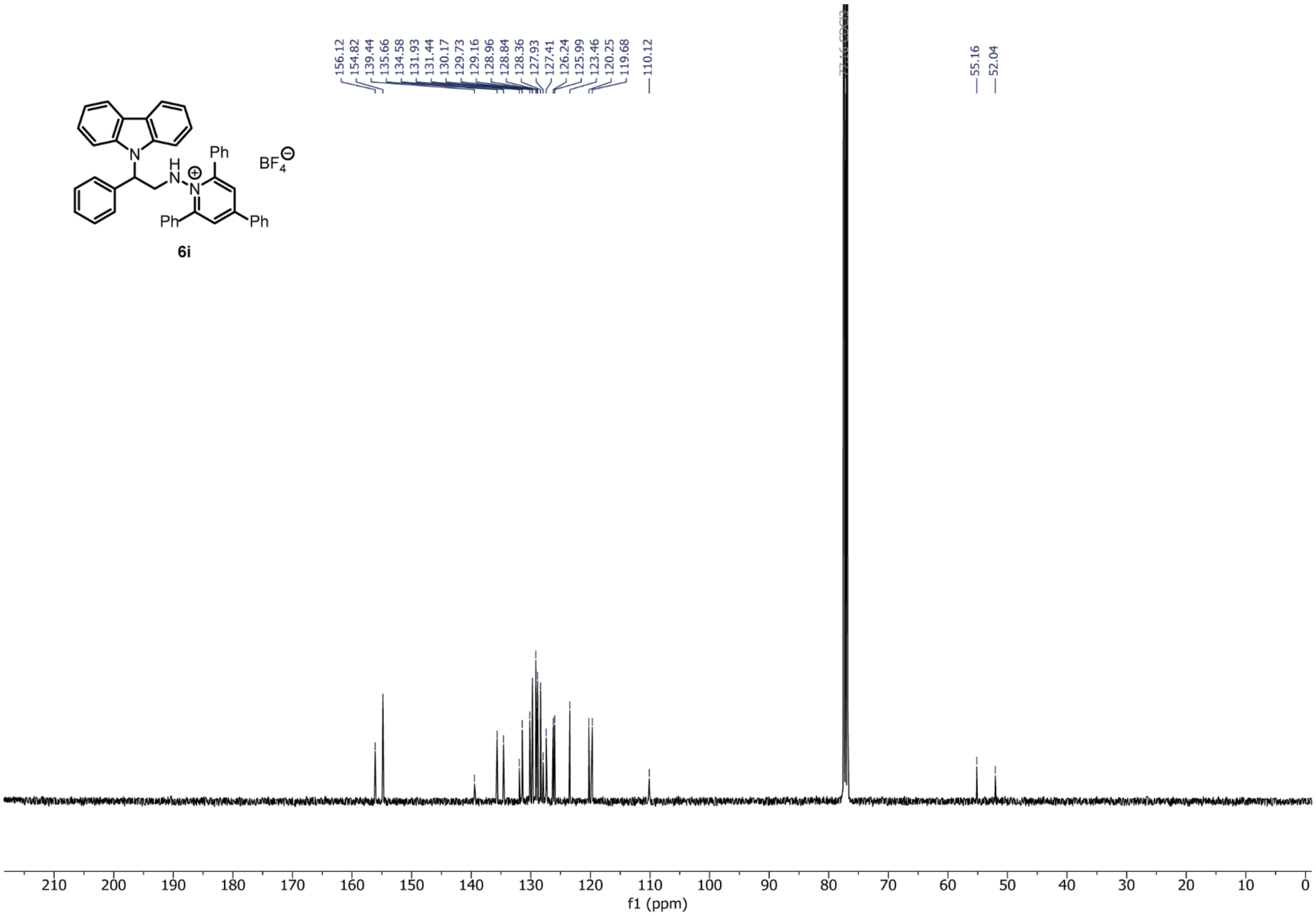

Supplementary Figure 126. ^{13}C NMR spectrum of 1-((2-(9*H*-carbazol-9-yl)-2-phenylethyl)amino)-2,4,6-triphenylpyridin-1-ium tetrafluoroborate (**6i**) in $CDCl_3$ (101 MHz) at 23 °C.

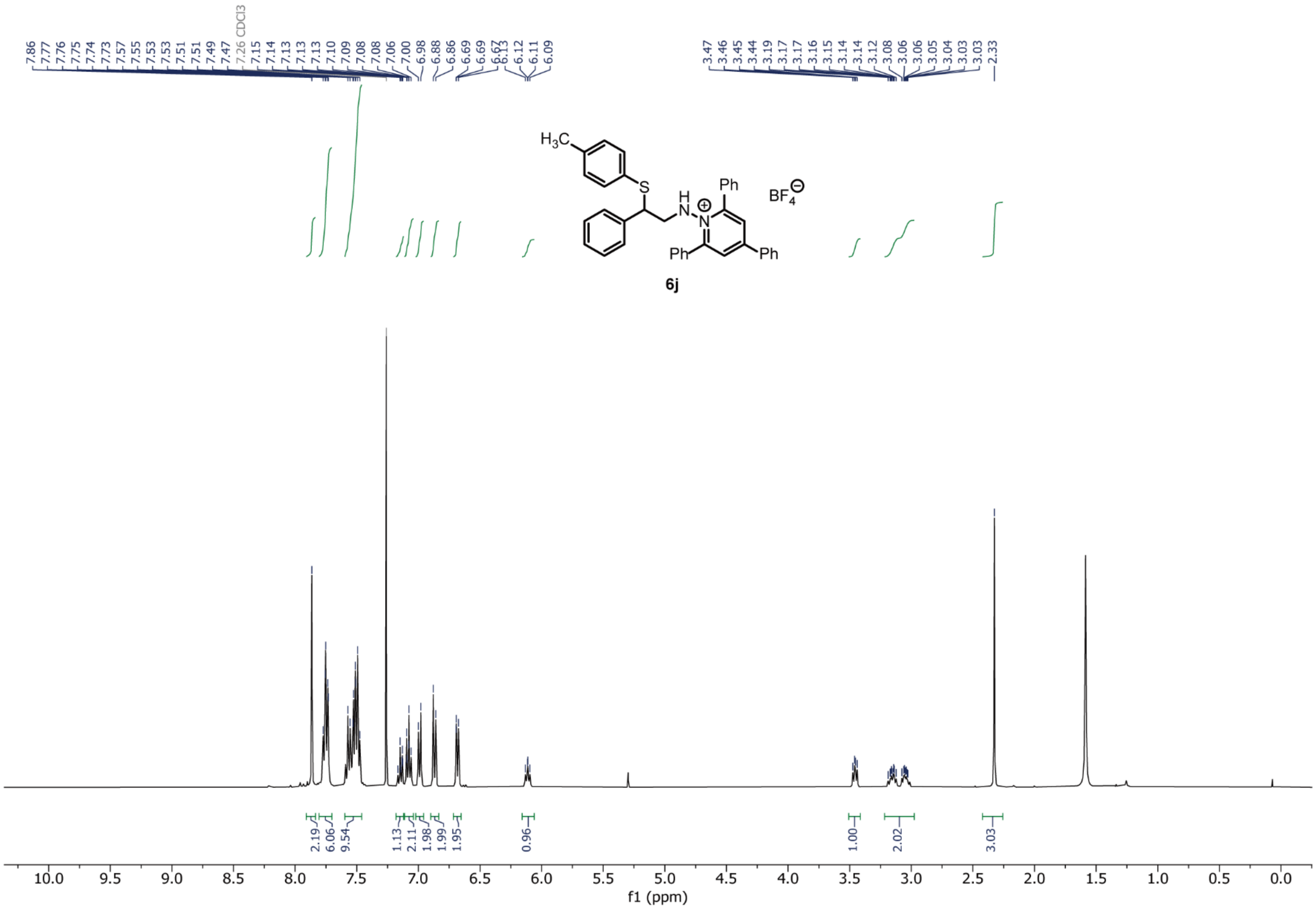

Supplementary Figure 127. ^{1}H NMR spectrum of 2,4,6-triphenyl-1-((2-phenyl-2-(*p*-tolylthio)ethyl)amino)pyridin-1-ium tetrafluoroborate (**6j**) in $CDCl_3$ (400 MHz) at 23 °C.

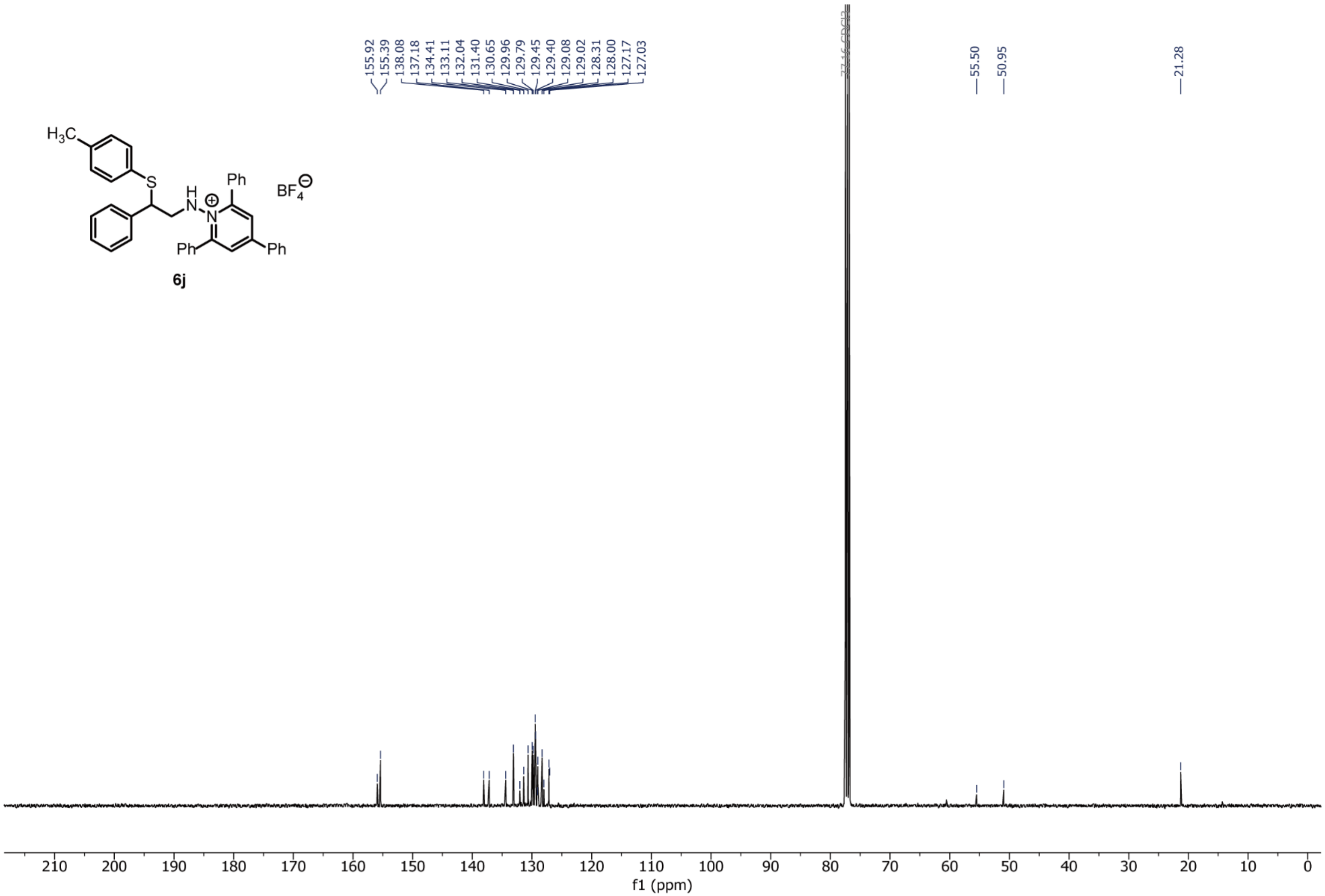

Supplementary Figure 128. ^{13}C NMR spectrum of 2,4,6-triphenyl-1-((2-phenyl-2-(*p*-tolylthio)ethyl)amino)pyridin-1-ium tetrafluoroborate (**6j**) in $CDCl_3$ (101 MHz) at 23 °C.

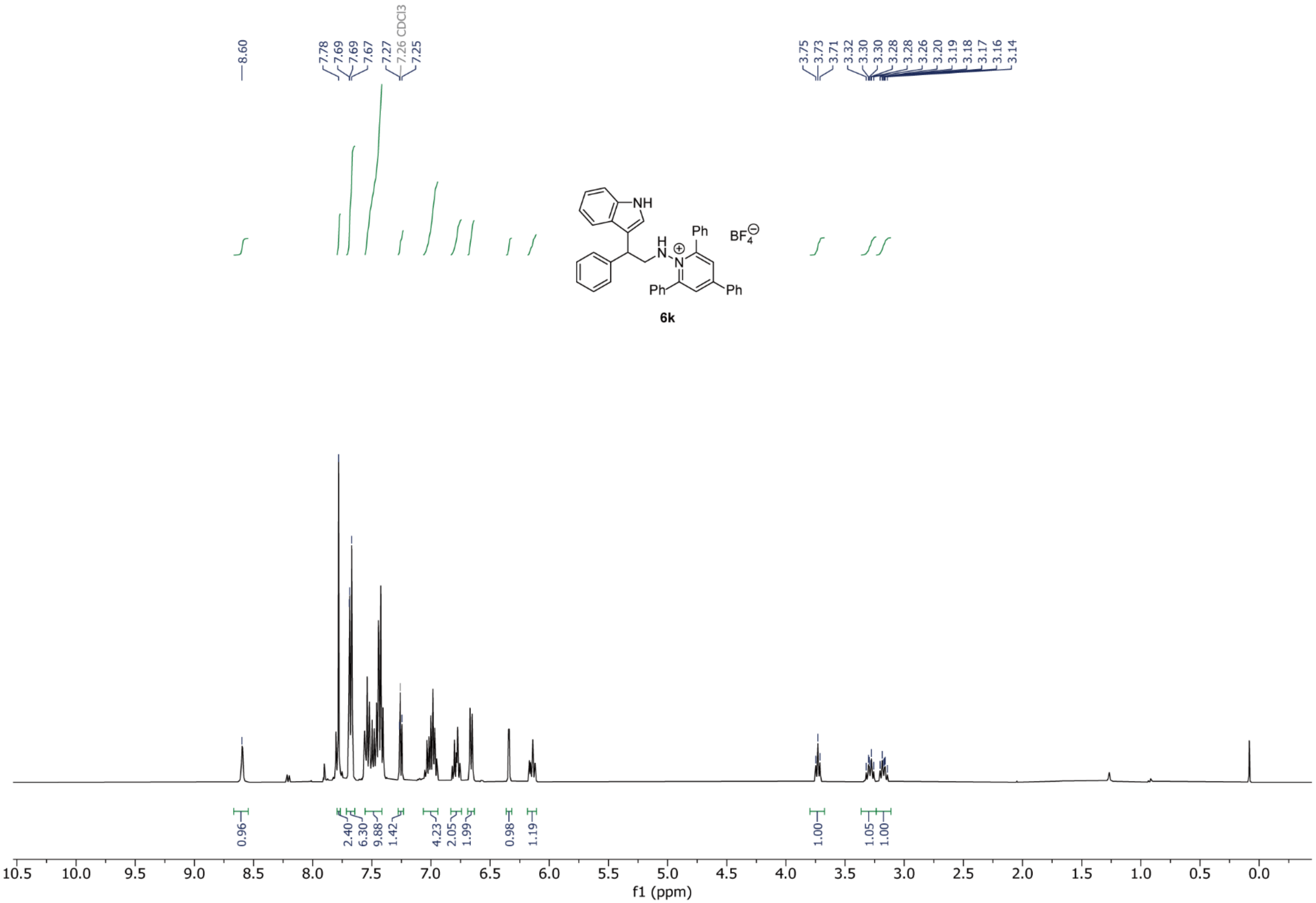

Supplementary Figure 129. ^{1}H NMR spectrum of 1-((2-(1H-indol-3-yl)-2-phenylethyl)amino)-2,4,6-triphenylpyridin-1-ium tetrafluoroborate (**6k**) in $CDCl_3$ (400 MHz) at 23 °C.

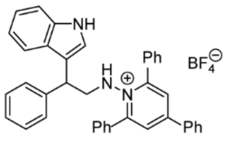

6k

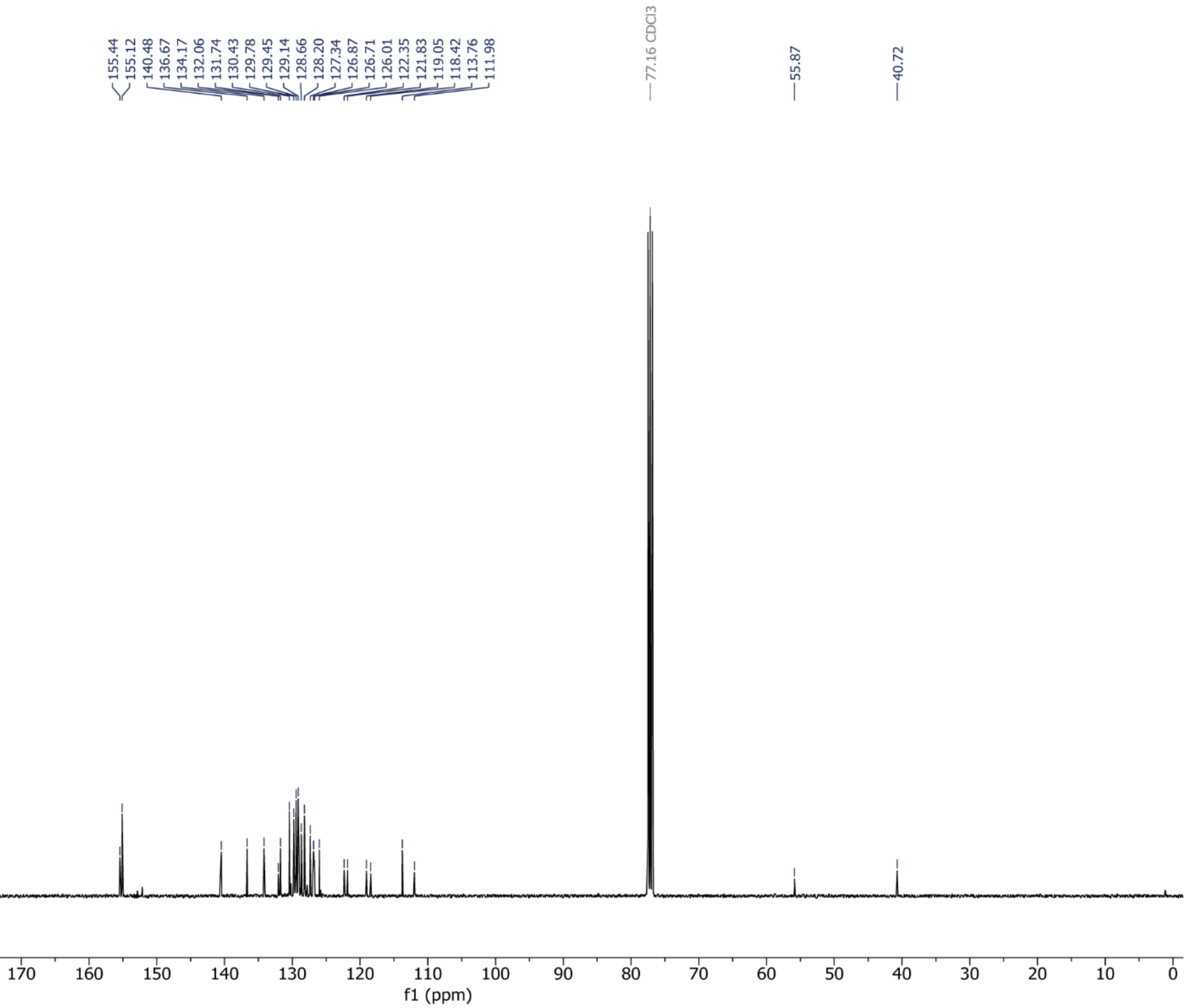

Supplementary Figure 130. ^{13}C NMR spectrum of 1-((2-(1H-indol-3-yl)-2-phenylethyl)amino)-2,4,6-triphenylpyridin-1-ium tetrafluoroborate (**6k**) in $CDCl_3$ (101 MHz) at 23 °C.

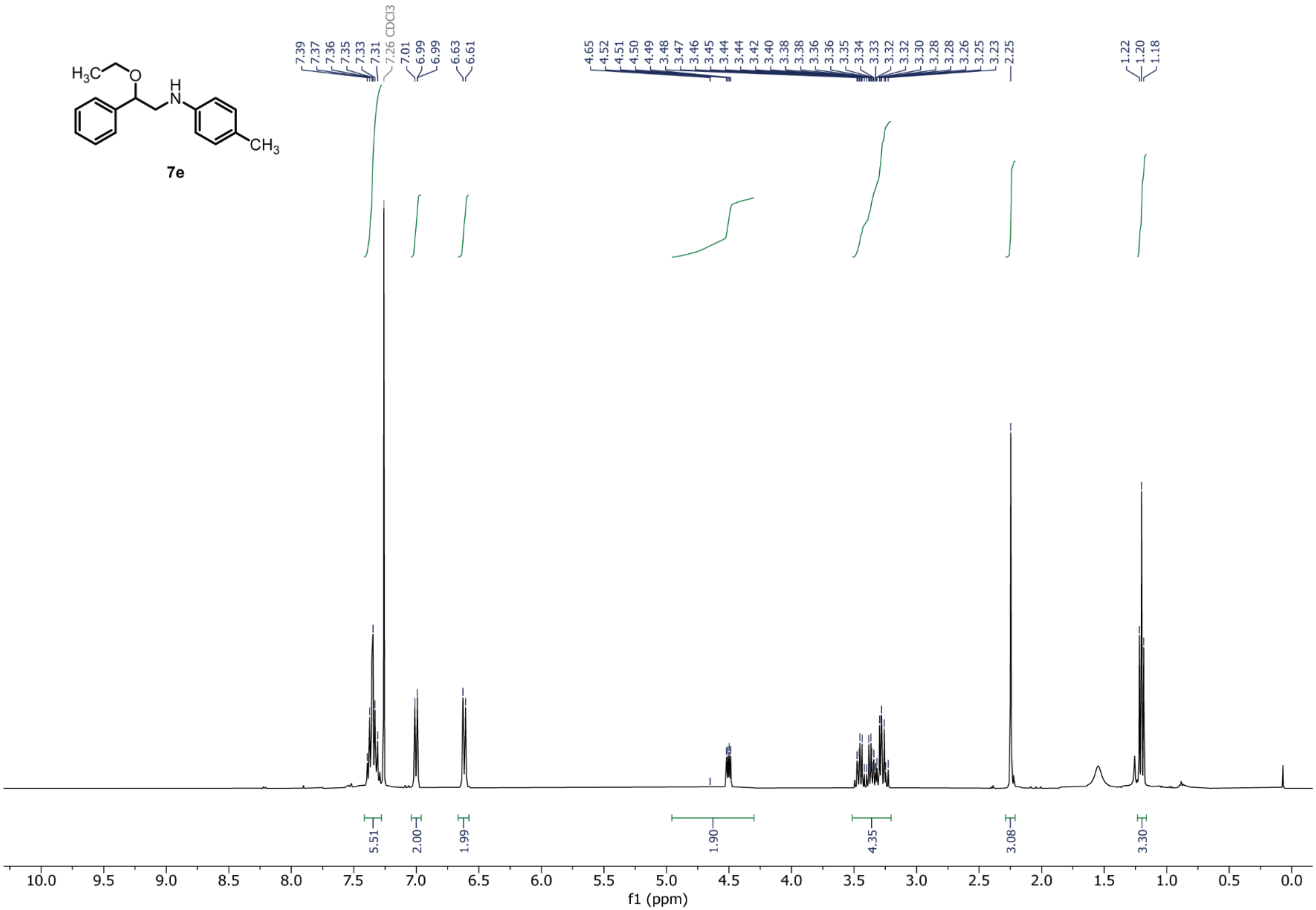

Supplementary Figure 131. ^{1}H NMR spectrum of *N*-(2-ethoxy-2-phenylethyl)-4-methylaniline (**7e**) in $CDCl_3$ (400 MHz) at 23 °C.

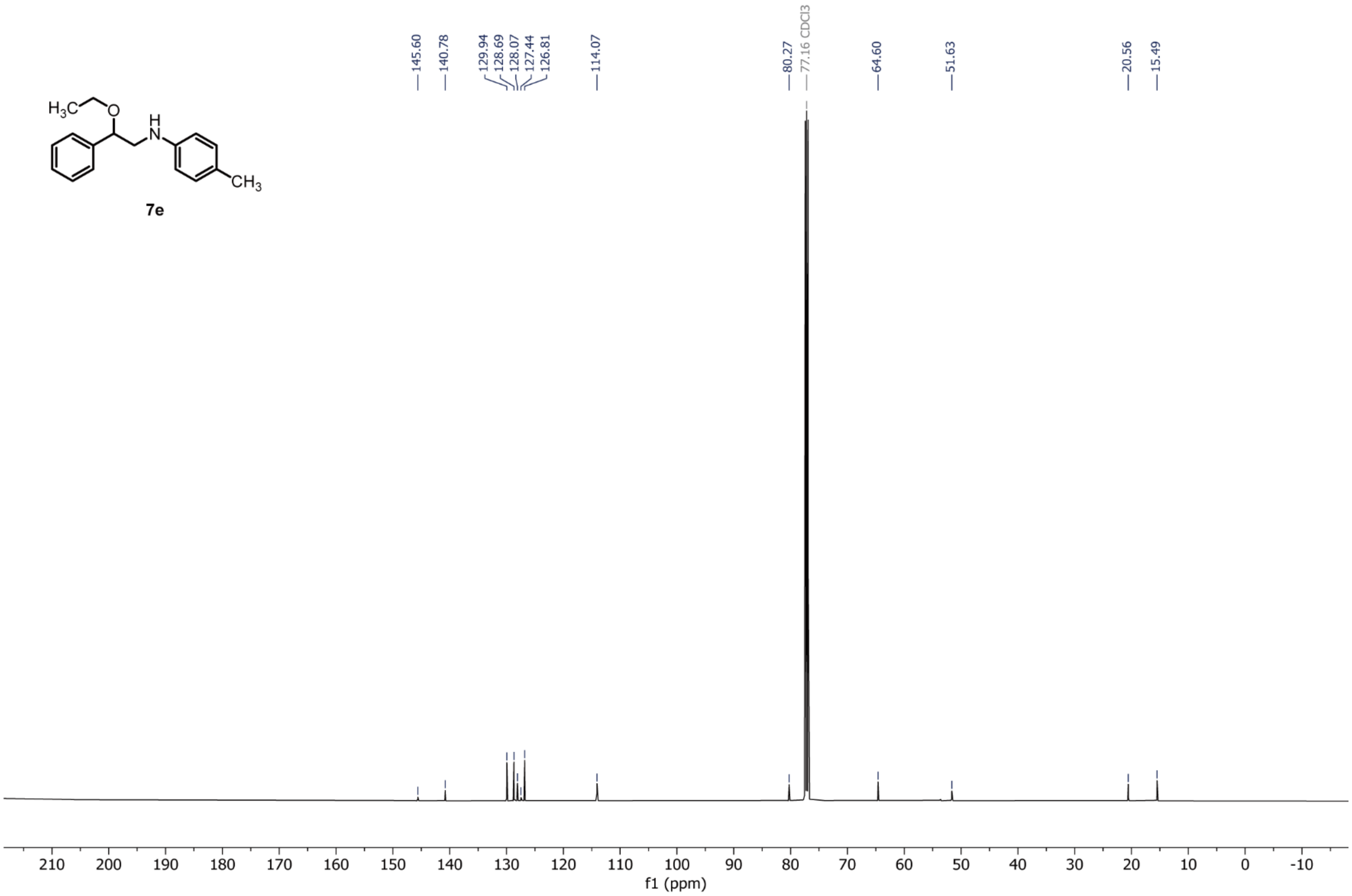

Supplementary Figure 132. ^{13}C NMR spectrum of *N*-(2-ethoxy-2-phenylethyl)-4-methylaniline (**7e**) in $CDCl_3$ (101 MHz) at 23 °C.

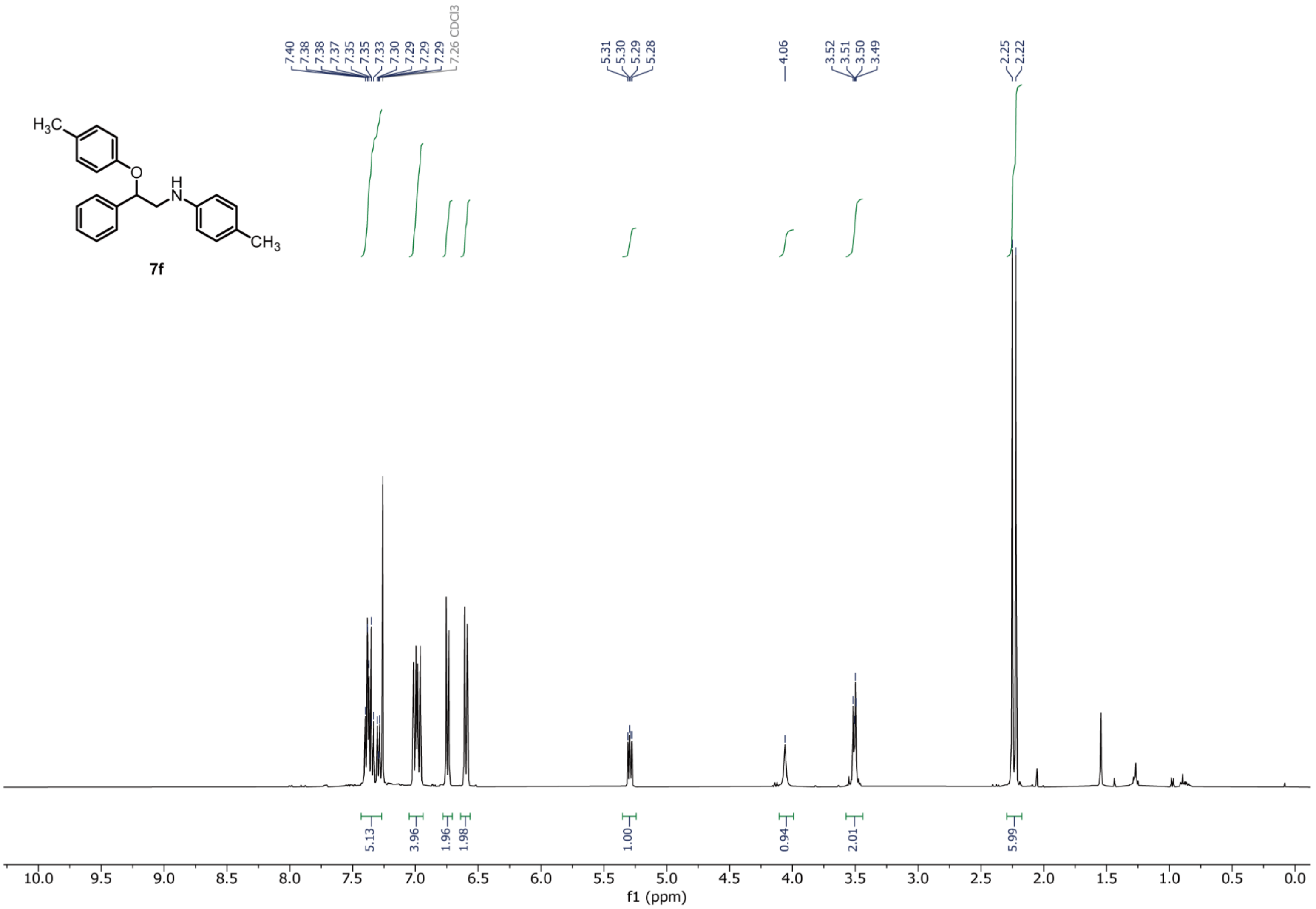

Supplementary Figure 133. ^{1}H NMR spectrum of 4-methyl-*N*-(2-phenyl-2-(*p*-tolyloxy)ethyl)aniline (**7f**) in $CDCl_3$ (400 MHz) at 23 °C.

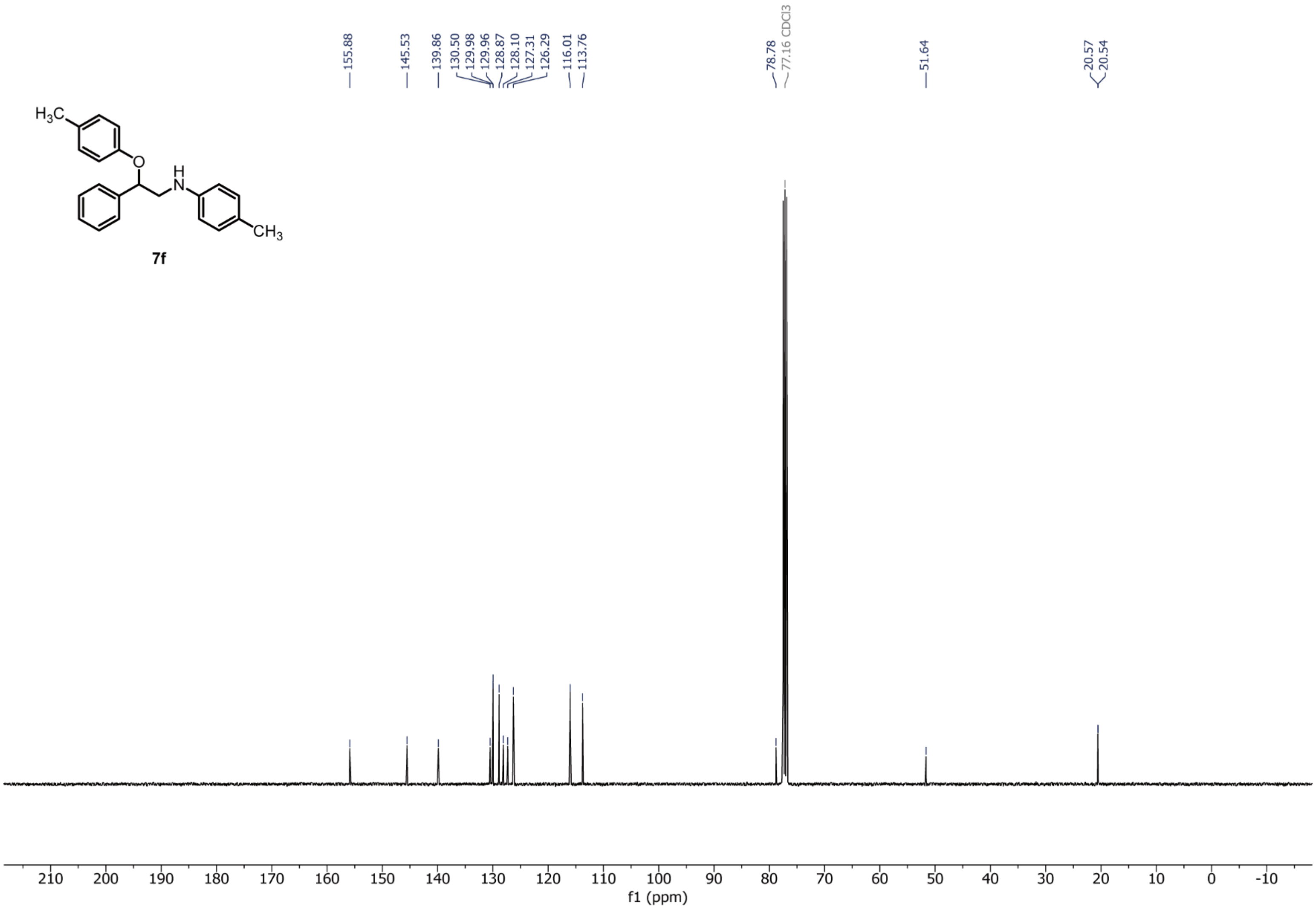

Supplementary Figure 134. ^{13}C NMR spectrum of 4-methyl-*N*-(2-phenyl-2-(*p*-tolyloxy)ethyl)aniline (**7f**) in $CDCl_3$ (101 MHz) at 23 °C.

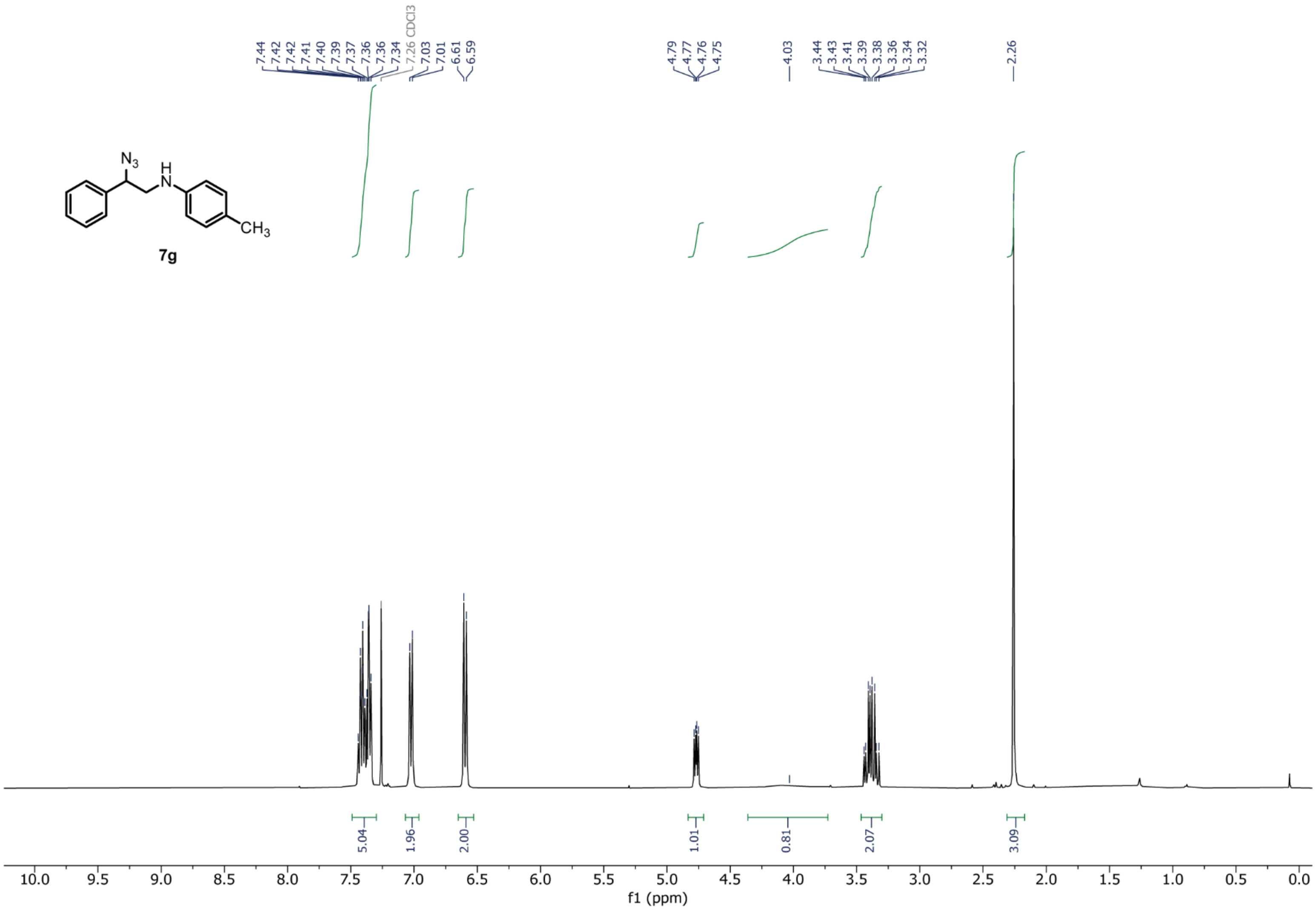

Supplementary Figure 135. ^{1}H NMR spectrum of *N*-(2-azido-2-phenylethyl)-4-methylaniline (**7g**) in $CDCl_3$ (400 MHz) at 23 °C.

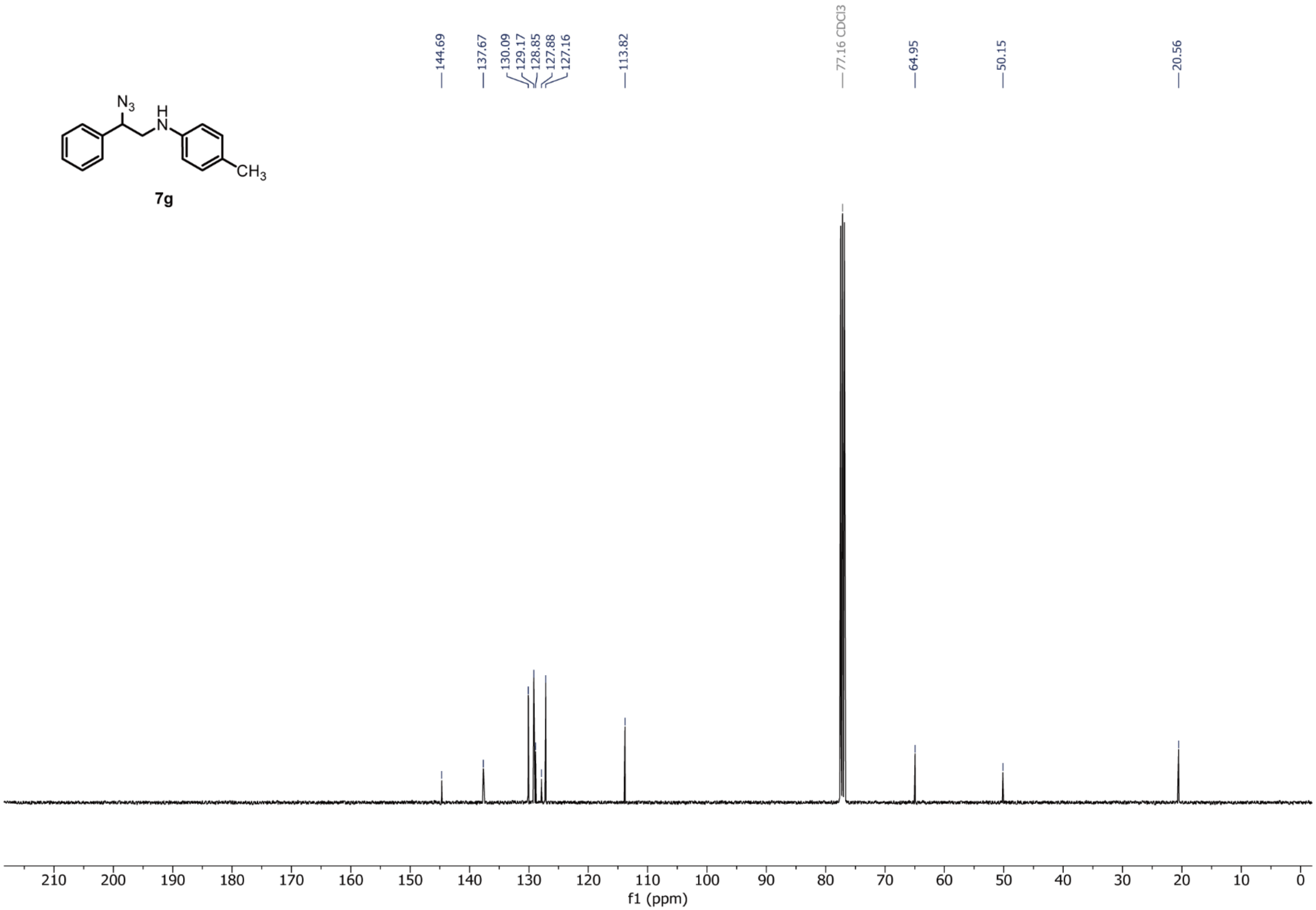

Supplementary Figure 136. ^{13}C NMR spectrum of *N*-(2-azido-2-phenylethyl)-4-methylaniline (**7g**) in $CDCl_3$ (101 MHz) at 23 °C.

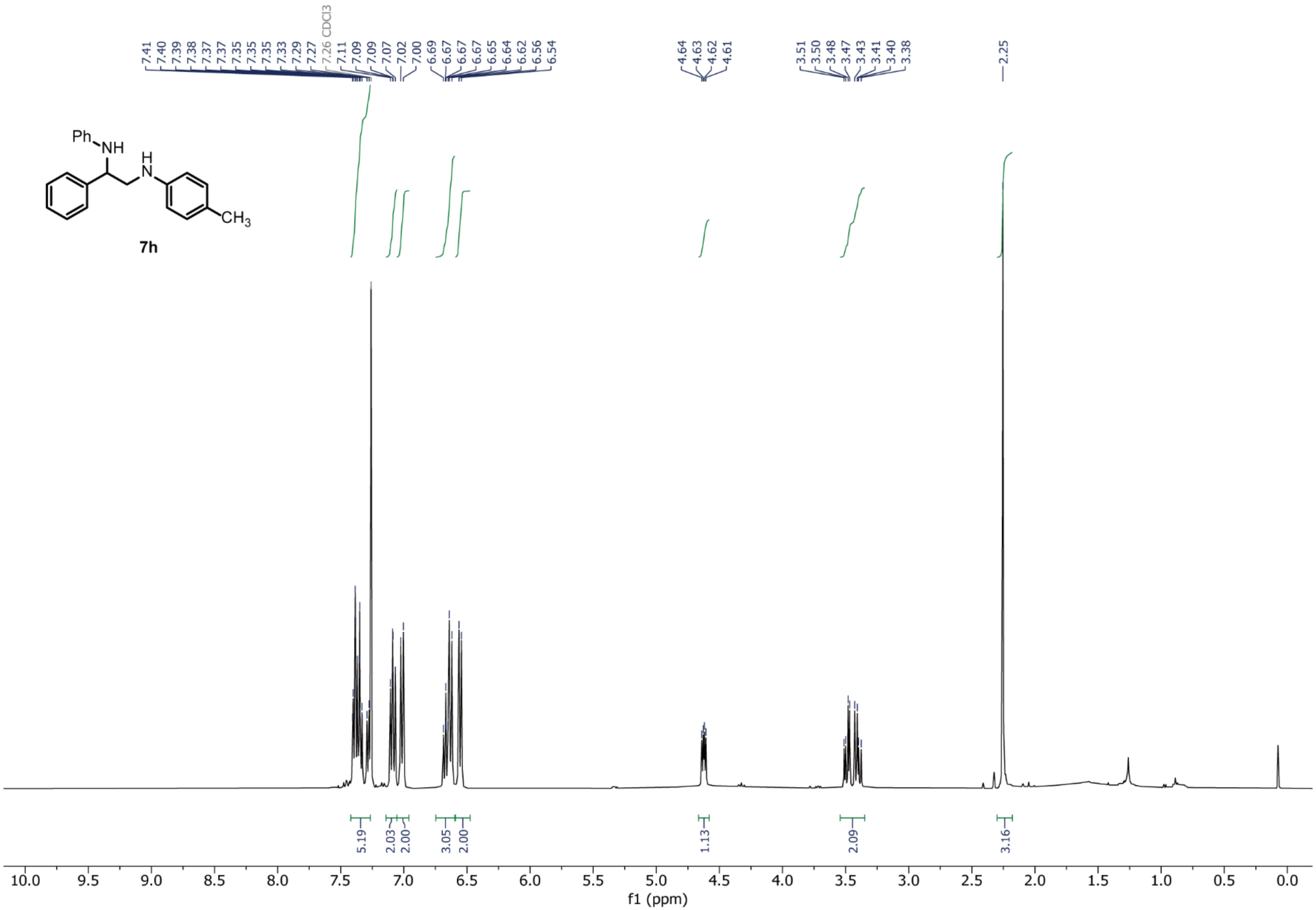

Supplementary Figure 137. ^{1}H NMR spectrum of N^{1},^{1}-diphenyl-N^{2}-(*p*-tolyl)ethane-1,2-diamine (**7h**) in $CDCl_3$ (400 MHz) at 23 °C.

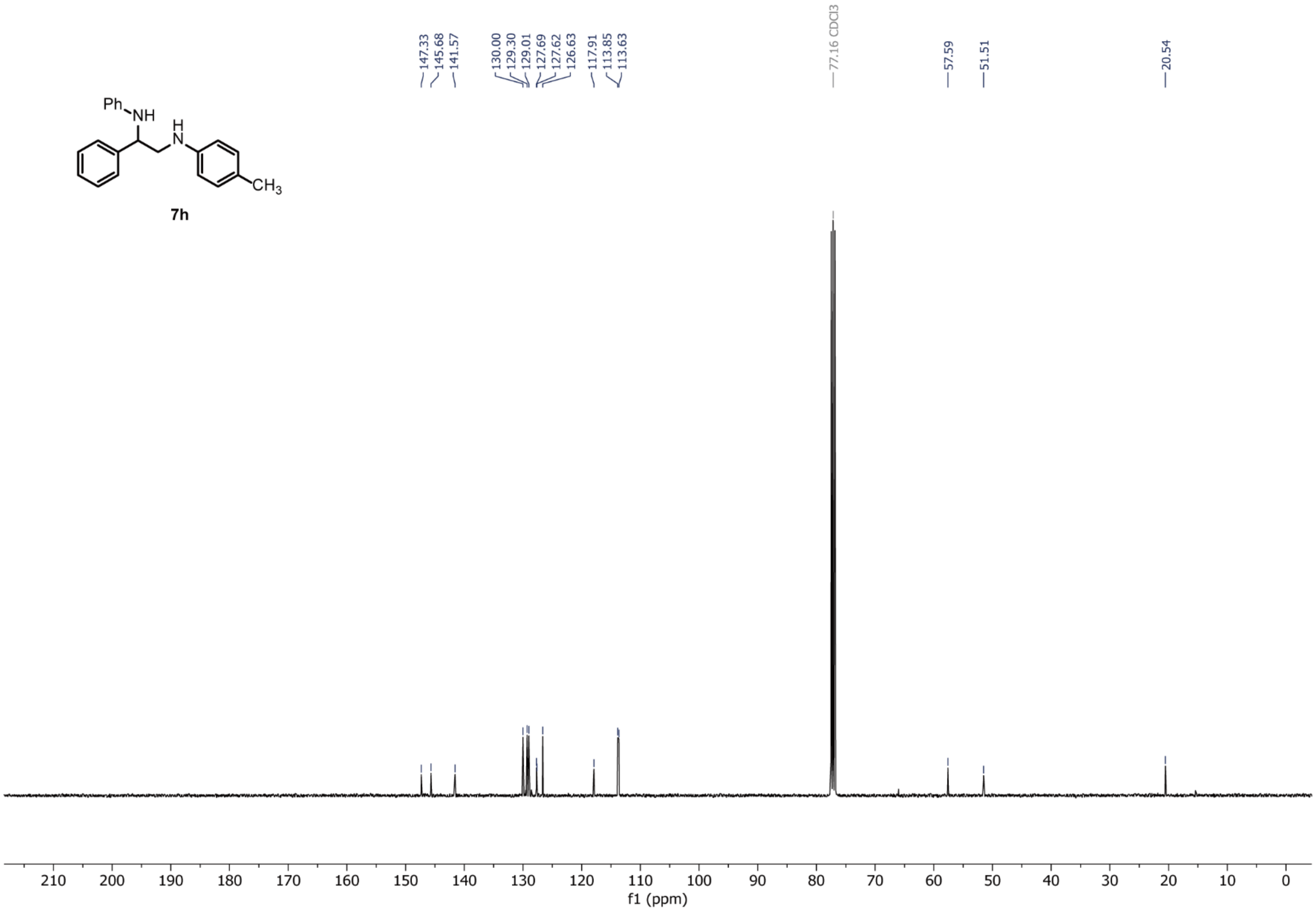

Supplementary Figure 138. ^{13}C NMR spectrum of N^1,1-diphenyl-N^2-(*p*-tolyl)ethane-1,2-diamine (**7h**) in $CDCl_3$ (101 MHz) at 23 °C.

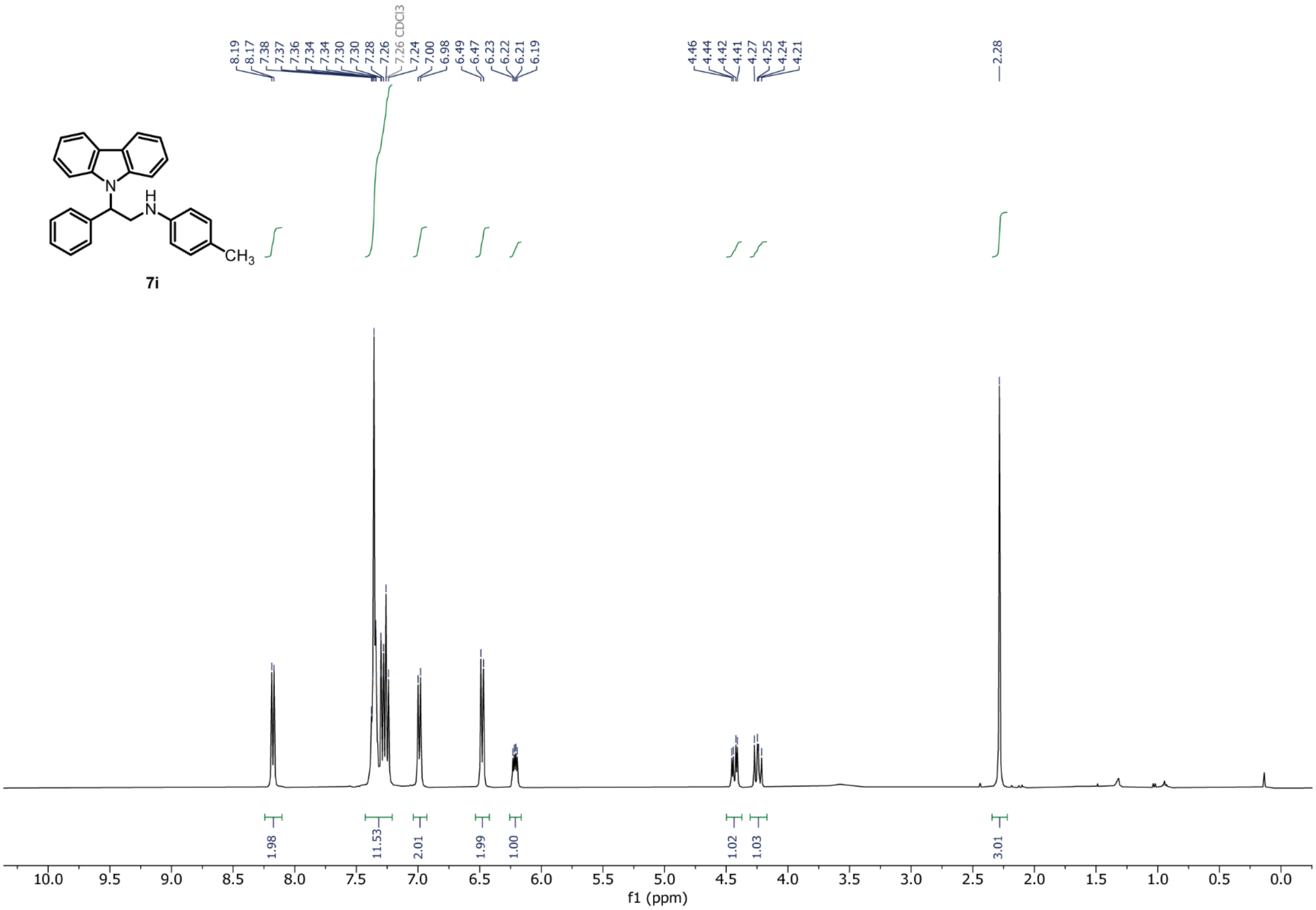

Supplementary Figure 139. ^{1}H NMR spectrum of *N*-(2-(9*H*-carbazol-9-yl)-2-phenylethyl)-4-methylaniline (**7i**) in $CDCl_3$ (400 MHz) at 23 °C.

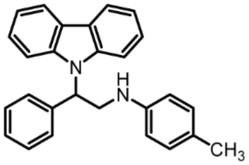

7i

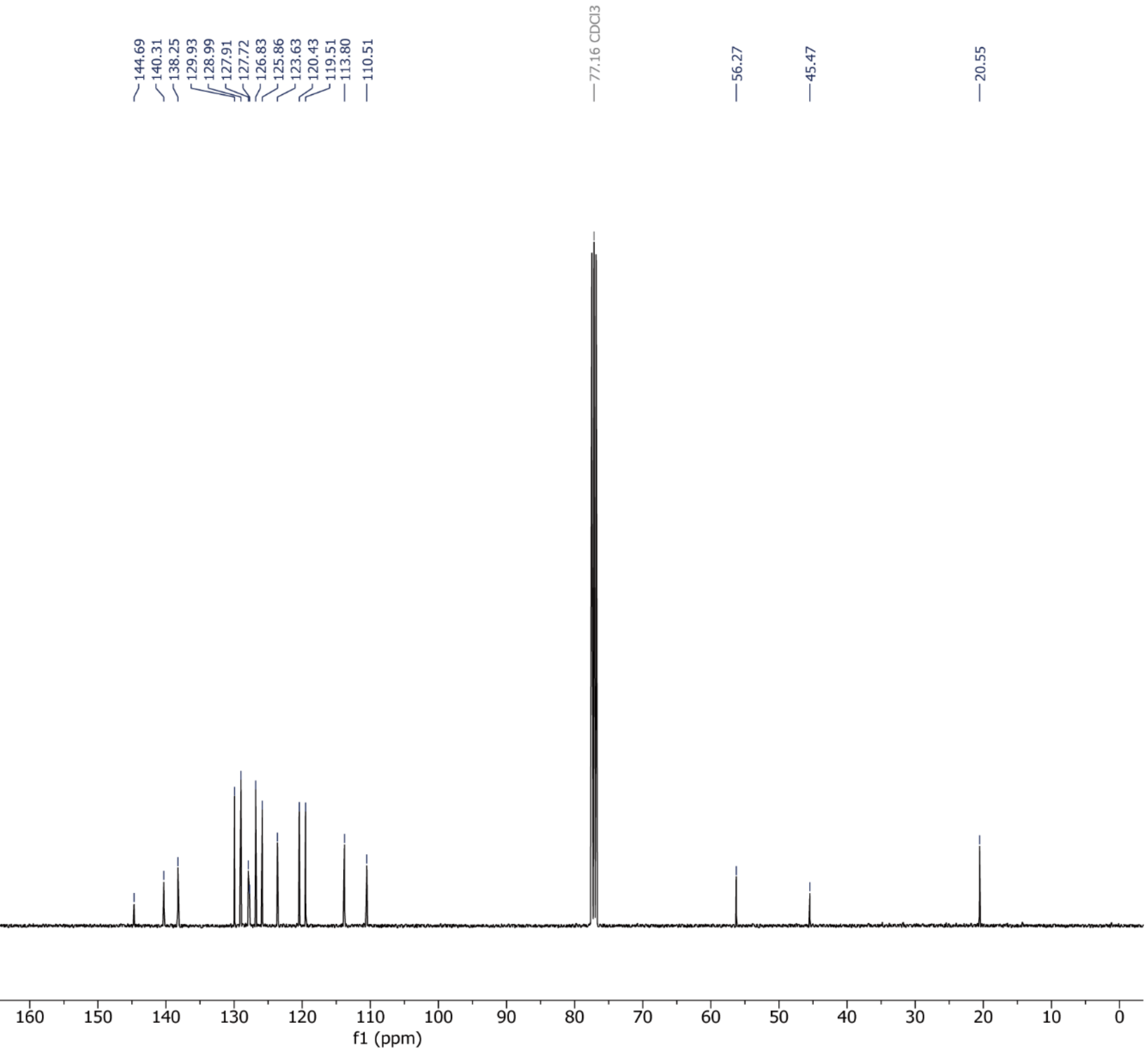

Supplementary Figure 140. ^{13}C NMR spectrum of *N*-(2-(9*H*-carbazol-9-yl)-2-phenylethyl)-4-methylaniline (**7i**) in $CDCl_3$ (101 MHz) at 23 °C.

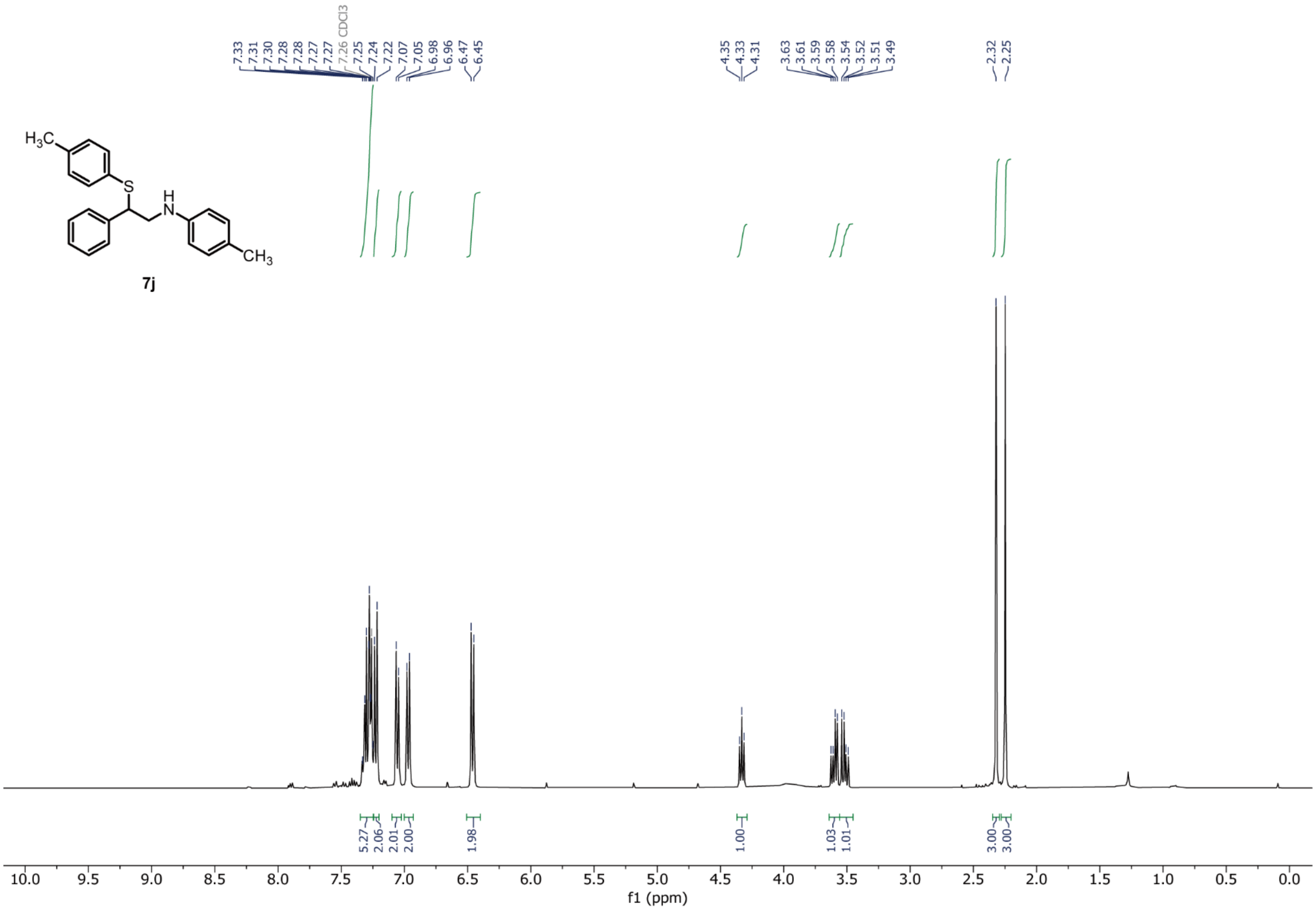

Supplementary Figure 141. ^{1}H NMR spectrum of 4-methyl-*N*-(2-phenyl-2-(*p*-tolylthio)ethyl)aniline (**7j**) in $CDCl_3$ (400 MHz) at 23 °C.

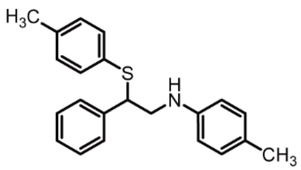

7j

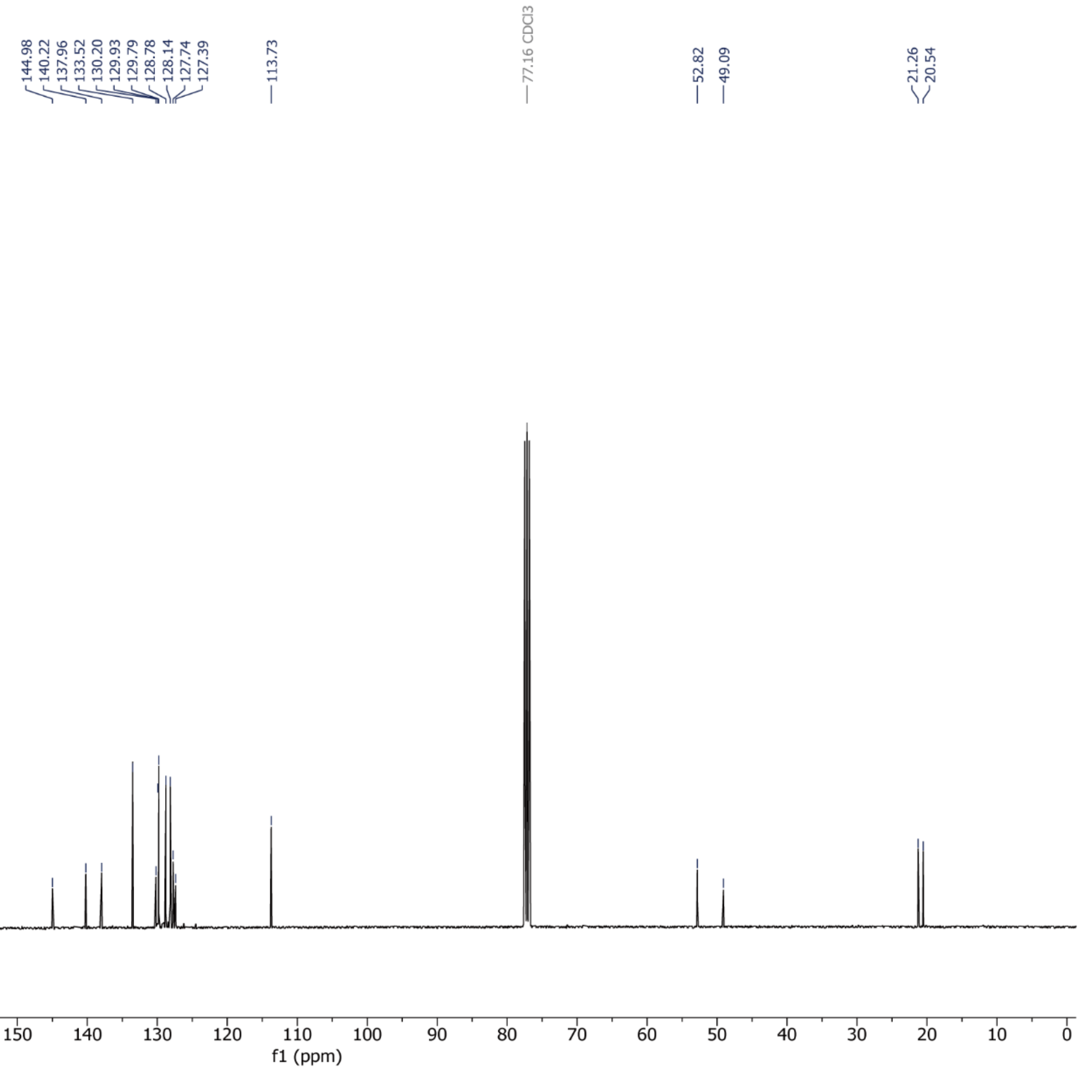

Supplementary Figure 142. ^{13}C NMR spectrum of 4-methyl-*N*-(2-phenyl-2-(*p*-tolylthio)ethyl)aniline (**7j**) in $CDCl_3$ (101 MHz) at 23 °C.

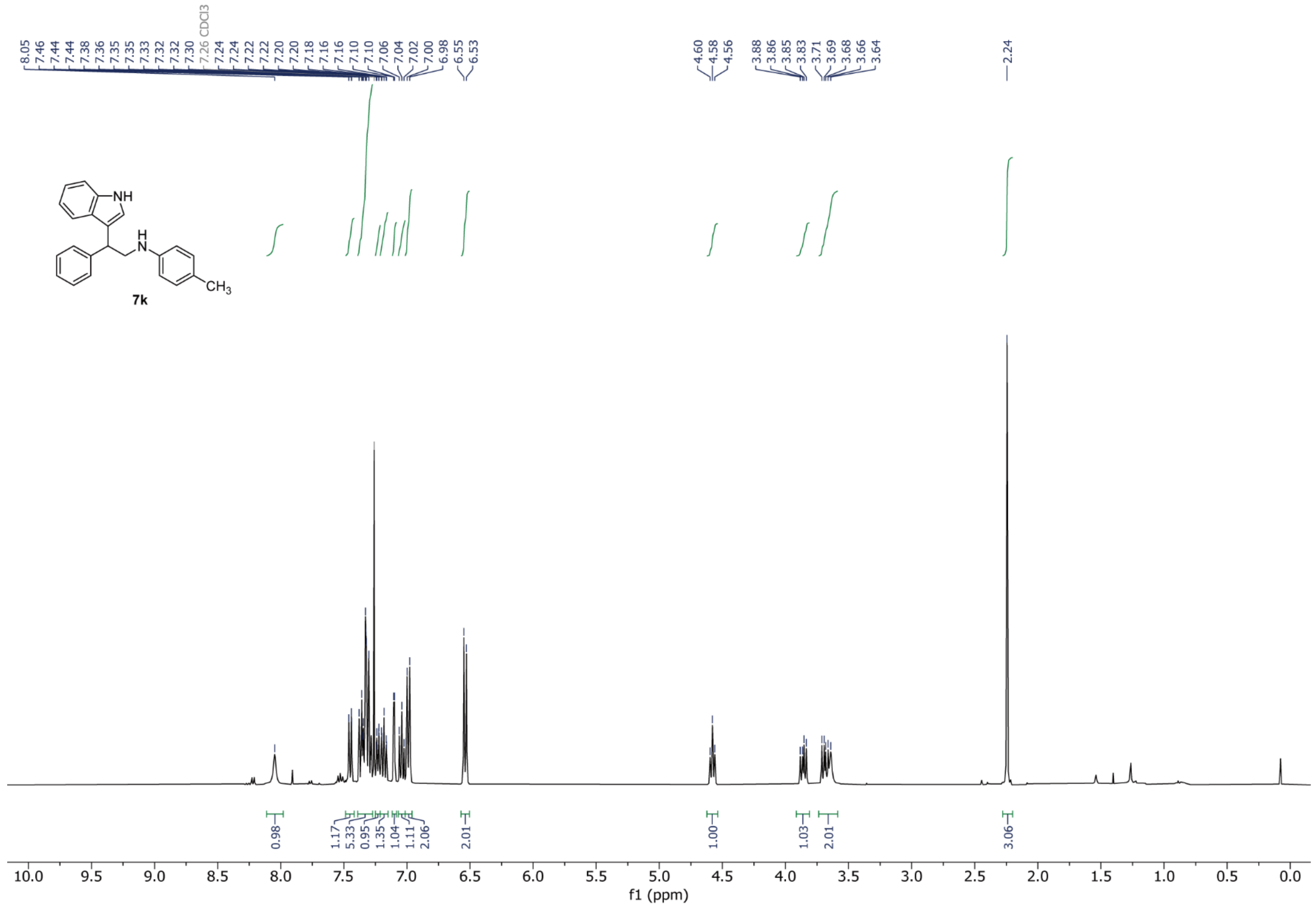

Supplementary Figure 143. ^{1}H NMR spectrum of *N*-(2-(1*H*-indol-3-yl)-2-phenylethyl)-4-methylaniline (**7k**) in $CDCl_3$ (400 MHz) at 23 °C.

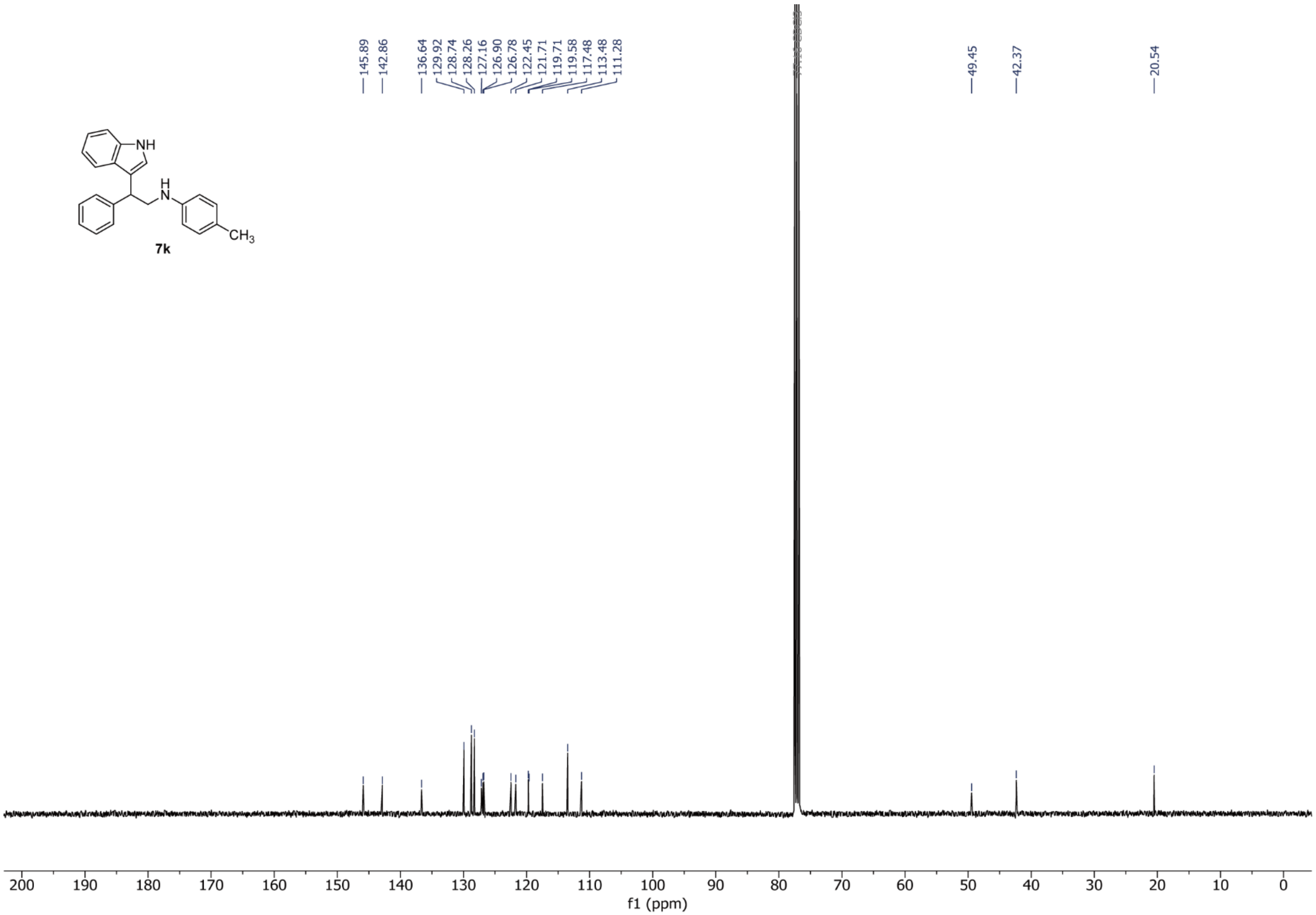

Supplementary Figure 144. ^{13}C NMR spectrum of *N*-(2-(1*H*-indol-3-yl)-2-phenylethyl)-4-methylaniline (**7k**) in $CDCl_3$ (101 MHz) at 23 °C.

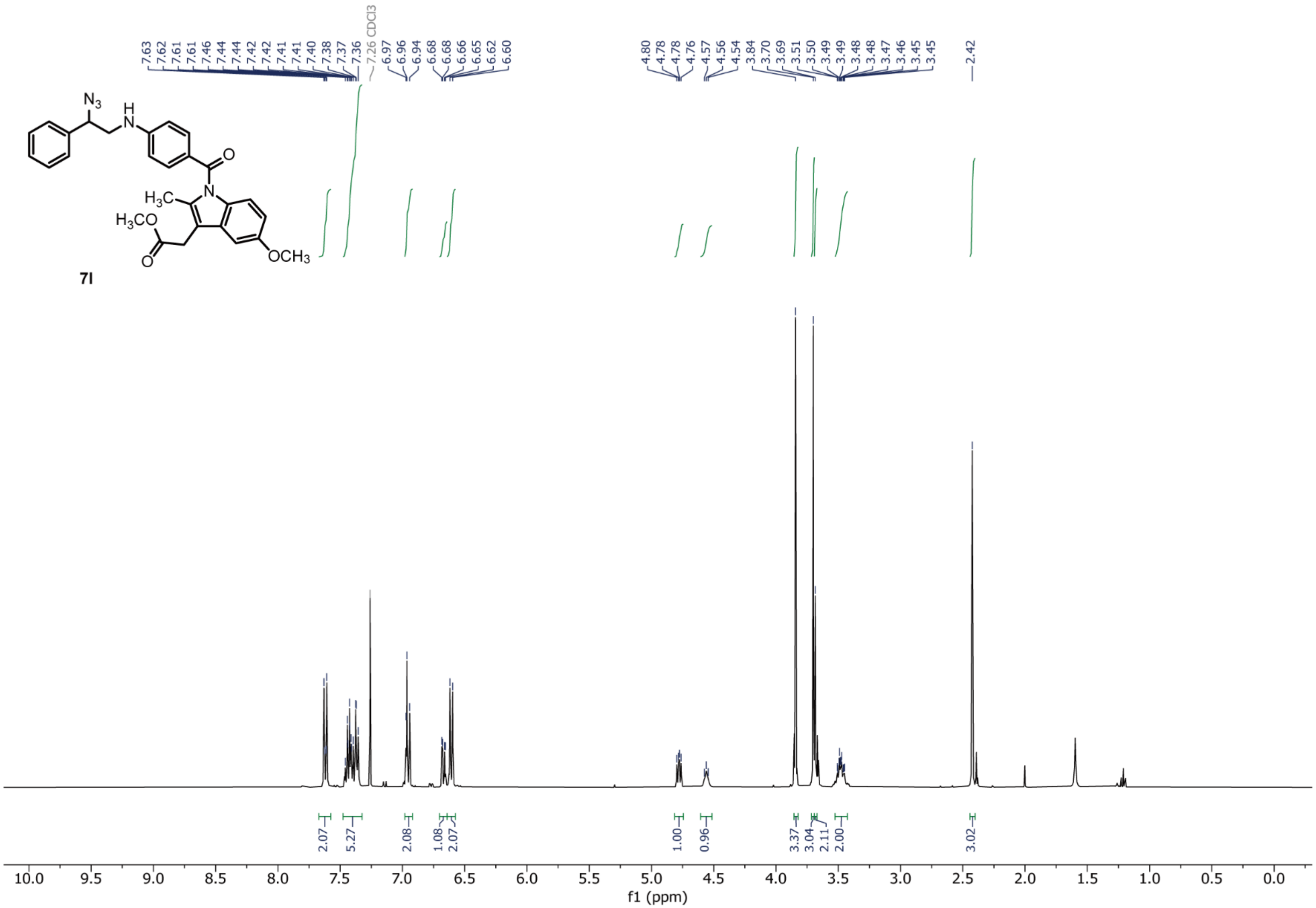

Supplementary Figure 145. ^{1}H NMR spectrum of methyl 2-(1- (4-((2-azido-2-phenylethyl)amino)benzoyl)-5-methoxy-2-methyl-1*H*-indol-3-yl)acetate (**7l**) in $CDCl_3$ (400 MHz) at 23 °C.

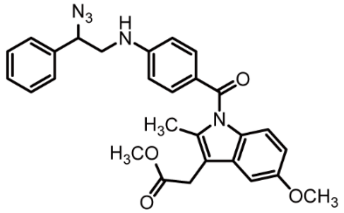

7l

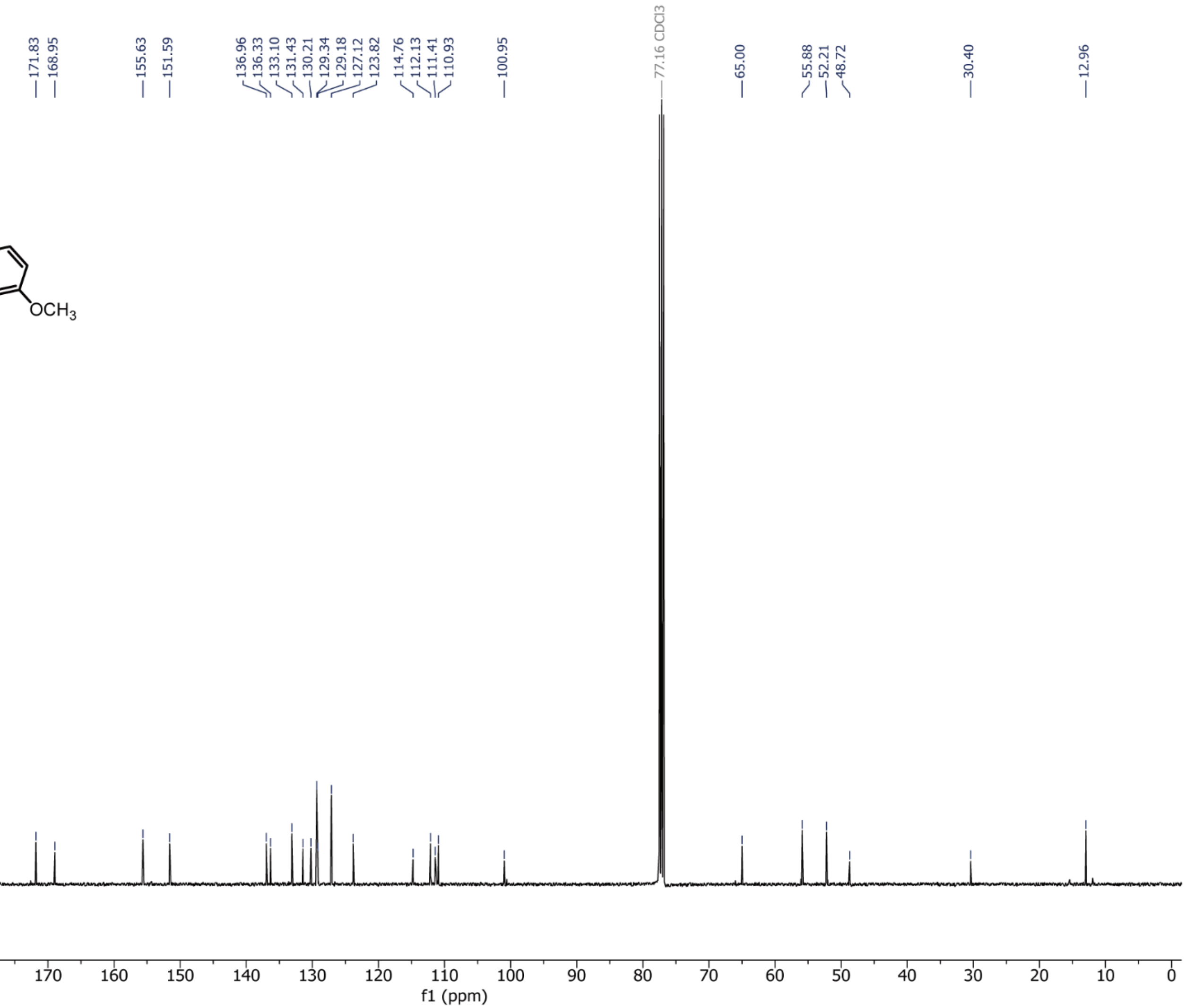

Supplementary Figure 146. ^{13}C NMR spectrum of methyl 2-(1-(4-((2-azido-2-phenylethyl)amino)benzoyl)-5-methoxy-2-methyl-1*H*-indol-3-yl)acetate (**7l**) in $CDCl_3$ (101 MHz) at 23 °C.

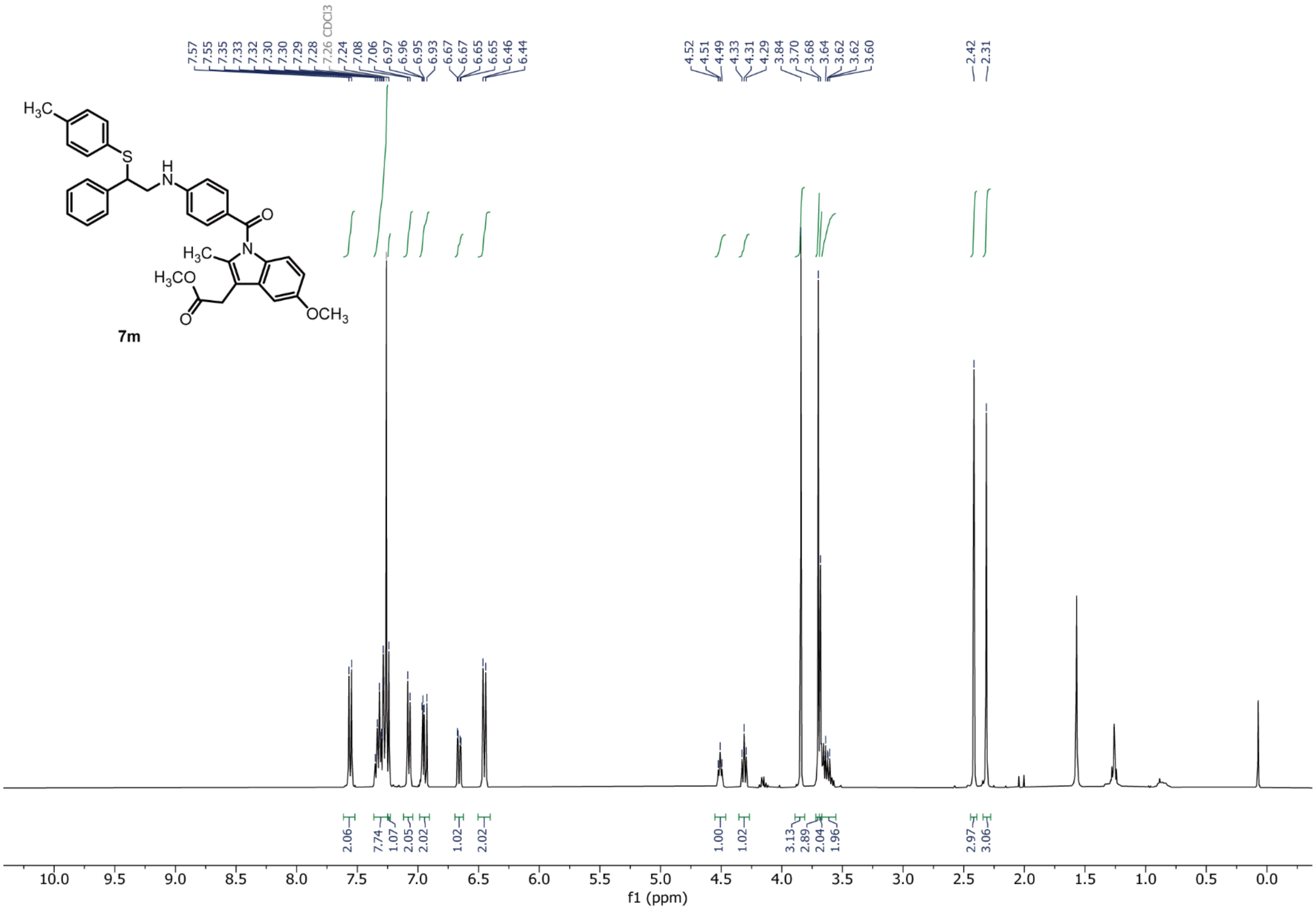

Supplementary Figure 147. ^{1}H NMR spectrum of methyl 2-(5-methoxy-2-methyl-1-(4-((2-phenyl-2-(*p*-tolylthio)ethyl)amino)benzoyl)-1*H*-indol-3-yl)acetate (**7m**) in $CDCl_3$ (400 MHz) at 23 °C.

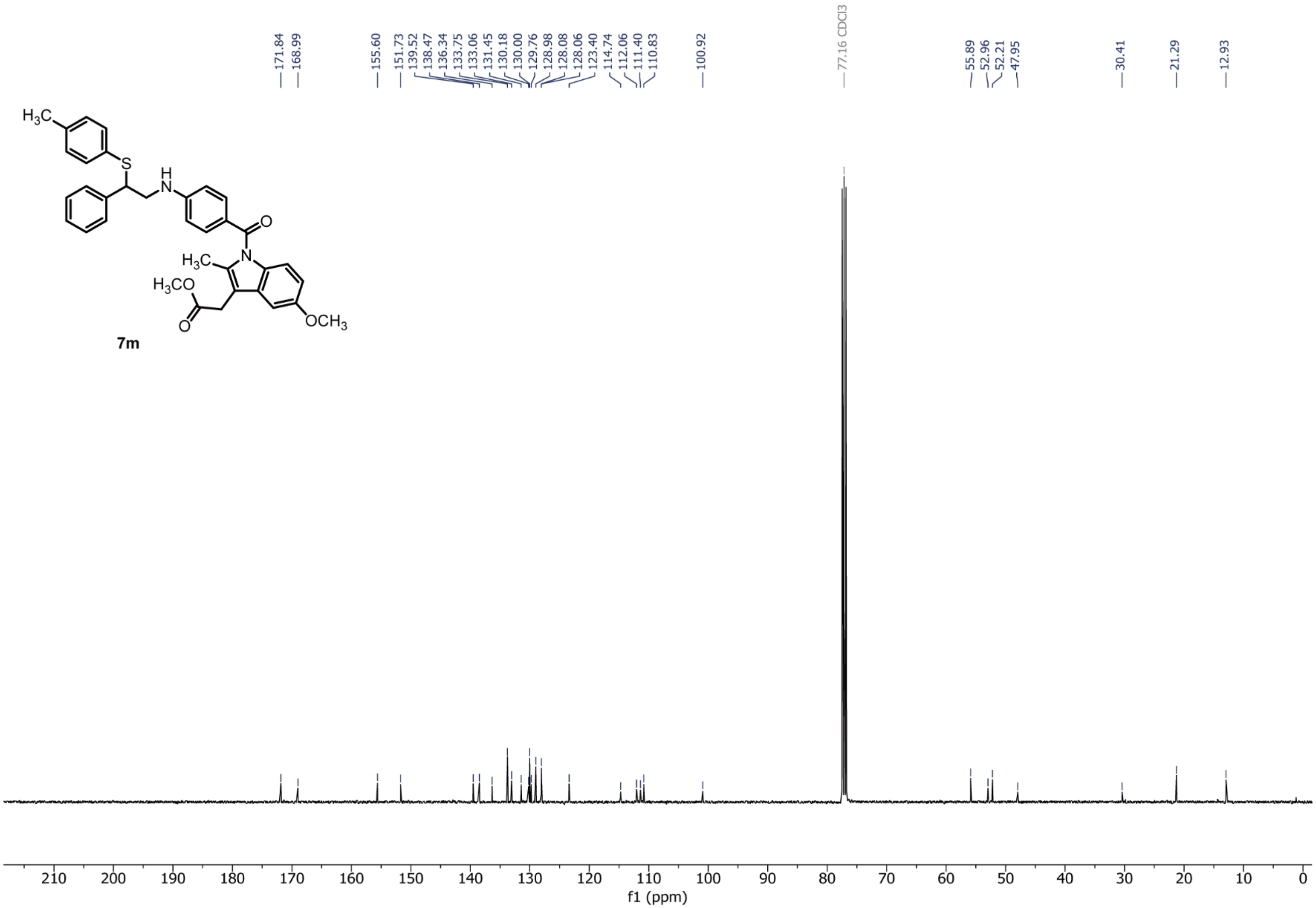

Supplementary Figure 148. ^{13}C NMR spectrum of methyl 2-(1-(4-((2-azido-2-phenylethyl)amino)benzoyl)-5-methoxy-2-methyl-1*H*-indol-3-yl)acetate (**7m**) in $CDCl_3$ (101 MHz) at 23 °C.

III. Supplementary References

1. Pangborn, A. B.; Giardello, M. A.; Grubbs, R. H.; Rosen, R. K.; Timmers, F. J. Safe and Convenient Procedure for Solvent Purification. *Organometallics* **1996**, *15*, 1518–1520.
2. Zha, Z.; Choi, S. R.; Ploessl, K.; Lieberman, B. P.; Qu, W.; Hefti, F.; Mintun, M.; Skovronsky, D.; Kung, H. F. Multidentate ^{18}F-Polypegylated Styrylpyridines As Imaging Agents for Aβ Plaques in Cerebral Amyloid Angiopathy (CAA). *J. Med. Chem.* **2011**, *54*, 8085–8098.
3. Gordillo, A.; Ortuño, M. A.; López-Mardomingo, C.; Lledós, A.; Ujaque, G.; de Jesús, E. Mechanistic Studies on the Pd-Catalyzed Vinylation of Aryl Halides with Vinylalkoxysilanes in Water: The Effect of the Solvent and NaOH Promoter. *J. Am. Chem. Soc.* **2013**, *135*, 13749–13763.
4. Gao, S.; Zhao, L.; Zhao, P.; Huang, Y.; Zhao, H. Synthesis, structure and characterization of a new highly porous zirconium-based metal-organic frameworks. *Inorg. Chim. Acta* **2018**, *480*, 173–176.
5. Rao, D. S.; Reddy, T. R.; Gurawa, A.; Kumar, M.; Kashyap, S. Photoswitchable Regiodivergent Azidation of Olefins with Sulfonium Iodate(I) Reagent. *Org. Lett.* **2019**, *21*, 9990–9994.
6. Chen, F.; Tang, Y.-T.; Li, X.-R.; Duan, Y.-Y.; Chen, C.-X.; Zheng, Y. Oxoammonium Salt-Mediated Vicinal Oxyazidation of Alkenes with NaN_3: Access to β-Aminooxy Azides. *Adv. Synth. Catal.* **2021**, *363*, 5079–5084.
7. Niu, D.; Buchwald, S. L. Design of Modified Amine Transfer Reagents Allows the Synthesis of α-Chiral Secondary Amines via CuH-Catalyzed Hydroamination. *J. Am. Chem. Soc.* **2015**, *137*, 9716–9721.
8. Li, G.; Qin, Z.; Radosevich, A. T. P(III)/P(V)-Catalyzed Methylamination of Arylboronic Acids and Esters: Reductive C–N Coupling with Nitromethane as a Methylamine Surrogate. *J. Am. Chem. Soc.* **2020**, *142*, 16205–16210.
9. Sheldrick, G. M. A Short History of SHELX. *Acta Cryst*. **2008**, *64*, 112–122.
10. Dolomanov, O. V.; Bourhis, L. J.; Gildea, R. J.; Howard, J. A.; Puschmann, H. OLEX2: A Complete Structure Solution, Refinement and Analysis Program. *J. Appl. Cryst*. **2009**, *42*, 339–341.
11. Sheldrick, G. M. Crystal Structure Refinement with SHELXL. *Acta Cryst*. **2015**, *71*, 3– 8.
12. Schönbauer, D.; Sambiago, C.; Noël, T.; Schnürch, M. Photocatalytic Deaminative Benzylation and Alkylation of Tetrahydroisoquinolines with *N*-Alkylpyrydinium Salts. *Beilstein J. Org. Chem.* **2020**, *16*, 809–817.
13. Beltrami, H.; Mallen, J. Reactions of 1-amino-2,4,6-triphenylpyridinium cations with α,β-unsaturated compounds synthesis of highly substituted pyrazolo [1,5-a]pyridine derivatives. *Tetrahedron* **1984**, *40*, 1683–1690.
14. Gösl, R.; Meuwsen, A. 1-AMINOPYRIDINIUM IODIDE. *Org. Synth.* **1963**, *43*, 1–3.
15. Zhang, J.-Z.; Tang, Y. Iron-Catalyzed Regioselective Oxo- and Hydroxy-Phthalimidation of Styrenes: Access to α-Hydroxyphthalimide Ketones. *Adv. Synth. Catal.* **2016**, *358*, 752–764.
16. Song, C.-X.; Chen, P.; Tang, Y. Carboxylation of Styrenes with CBr_4 and DMSO via Cooperative Photoredox and Cobalt Catalysis. *RSC Advances* **2017**, *7*, 11233–11243.
17. Tang, M.; Han, S.; Huang, S.; Huang, S.; Xie, L.-G. Carbosulfenylation of Alkenes with Organozinc Reagents and Dimethyl(methylthio)sulfonium Trifluoromethanesulfonate. *Org. Lett.* **2020**, *22*, 9729–9734.
18. Bialek, M.; Cramail, H.; Deffieux, A.; Guillaume, S. M. Styrene Polymerization using Nickel(II) Complexes as Catalysts. *Eur. Polym. J.* **2005**, *41*, 2678–2684.
19. Lin, Q.; Diao, T. Mechanism of Ni-Catalyzed Reductive 1,2-Dicarbofunctionalization of Alkenes. *J. Am. Chem. Soc.* **2019**, *141*, 17937–17948.
20. Aihara, Y.; Chatani, N. Nickel-Catalyzed Direct Alkylation of C–H Bonds in Benzamides and Acrylamides with Functionalized Alkyl Halides via Bidentate-Chelation Assistance. *J. Am. Chem. Soc.* **2013**, *135*, 5308–5311.

21. Graham, T. J. A.; Doyle, A. G. Nickel-Catalyzed Cross-Coupling of Chromene Acetals and Boronic Acids. *Org. Lett.* **2012**, *14*, 1616–1619.
22. Sebest, F.; Casarrubios, L.; Rzepa, H. S.; White, A. J. P.; Díez-González, S. Thermal azide–alkene cycloaddition reactions: straightforward multi-gram access to Δ2-1,2,3-triazolines in deep eutectic solvents. *Green Chemistry* **2018**, *20*, 4023–4035.
23. Liao, J.; Basch, C. H.; Hoerrner, M. E.; Talley, M. R.; Boscoe, B. P.; Tucker, J. W.; Garnsey, M. R.; Watson, M. P. Deaminative Reductive Cross-Electrophile Couplings of Alkylpyridinium Salts and Aryl Bromides. *Org. Lett.* **2019,** *21*, 2941–2946.
24. Kiyokawa, K.; Kosaka, T.; Minakata, S. Metal-Free Aziridination of Styrene Derivatives with Iminoiodinane Catalyzed by a Combination of Iodine and Ammonium Iodide. *Org. Lett.* **2013**, *15*, 4858–4861.
25. Evans, D. A.; Bilodeau, M. T.; Faul, M. M. Development of the Copper-Catalyzed Olefin Aziridination Reaction. *J. Am. Chem. Soc.* **1994**, *116*, 2742–2753.
26. Hua, Y.-G.; Yang, Q.-Q.; Yang, Y.; Wang, M.-J.; Chu, W.-C.; Bai, P.-Y.; Cui, D.-Y.; Zhang, E.; Liu, H.-M. Metal-free synthesis of 1,2-amino alcohols by one-pot olefin aziridination and acid ring-opening. *Tetrahedron Lett.* **2018,** *59*, 2748–2751.
27. Watile, R. A.; Bagal, D. B.; Deshmukh, K. M.; Dhake, K. P.; Bhanage, B. M. Polymer supported diol functionalized ionic liquids: An efficient, heterogeneous and recyclable catalyst for 5-aryl-2-oxazolidinones synthesis from CO2 and aziridines under mild and solvent free condition. *J. Mol. Catal. A: Chem.* **2011,** *351*, 196–203.
28. Sasaki, M.; Dalili, S.; Yudin, A. K. N-Arylation of Aziridines. *J. Org. Chem.* **2003,** *68*, 2045–2047
